# Tetraspanner‐based nanodomains modulate BAR domain‐induced membrane curvature

**DOI:** 10.15252/embr.202357232

**Published:** 2023-10-30

**Authors:** Daniel Haase, Christiane Rasch, Ulrike Keller, Yaroslav Tsytsyura, Nataliya Glyvuk, Annegret Elting, Julia Wittmar, Annette Janning, Martin Kahms, Noah Wedlich, Christian Schuberth, Andreas Heuer, Jürgen Klingauf, Roland Wedlich‐Söldner

**Affiliations:** ^1^ Institute of Cell Dynamics and Imaging, and Cells‐in‐Motion Interfaculty Center (CiMIC), University of Münster Münster Germany; ^2^ Institute for Medical Physics and Biophysics, and Cells‐in‐Motion Interfaculty Center (CiMIC) Münster Germany; ^3^ Center for Soft Nanoscience Münster Germany; ^4^ Institute for Physical Chemistry Münster Germany

**Keywords:** BAR domain proteins, membrane curvature, plasma membrane, tetraspanners, yeast, Membranes & Trafficking

## Abstract

The topography of biological membranes is critical for formation of protein and lipid microdomains. One prominent example in the yeast plasma membrane (PM) are BAR domain‐induced PM furrows. Here we report a novel function for the Sur7 family of tetraspanner proteins in the regulation of local PM topography. Combining TIRF imaging, STED nanoscopy, freeze–fracture EM and membrane simulations we find that Sur7 tetraspanners form multimeric strands at the edges of PM furrows, where they modulate forces exerted by BAR domain proteins at the furrow base. Loss of Sur7 tetraspanners or Sur7 displacement due to altered PIP2 homeostasis leads to increased PM invagination and a distinct form of membrane tubulation. Physiological defects associated with PM tubulation are rescued by synthetic anchoring of Sur7 to furrows. Our findings suggest a key role for tetraspanner proteins in sculpting local membrane domains. The maintenance of stable PM furrows depends on a balance between negative curvature at the base which is generated by BAR domains and positive curvature at the furrows' edges which is stabilized by strands of Sur7 tetraspanners.

## Graphical abstract

Maintenance of stable PM furrows in budding yeast depends on Sur7 tetraspanner strands at the upper edge that counteract the negative curvature generated by BAR domains at the furrow base.

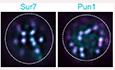

## Introduction

As the primary interface between cells and their environment, the plasma membrane (PM) serves a wide range of biological functions including nutrient uptake, metabolic homeostasis, signal transduction and cell–cell communication. In order to perform these tasks, the PM is laterally segregated into a multitude of nanometer or micrometer‐sized domains. The formation of these domains has been studied extensively, focusing on either lipid‐driven mechanisms such as raft formation (Simons & Ikonen, 1997) and hydrophobic mismatch (Killian, 1998) or protein‐based structures such as cortical actin fences (Kusumi *et al*, 2011) and other multimeric networks (Mueller *et al*, 2012). A common theme in this context is the importance of collective and cooperative interactions between large numbers of individual components.

One particularly relevant class of proteins involved in PM organization is made up of the so‐called tetraspanners, proteins with four transmembrane domains that often form multimeric complexes. Prominent examples of such assemblies are the tetraspanin webs that organize signaling complexes in immune cells (Boucheix & Rubinstein, 2001; Levy & Shoham, 2005) and the claudin and occludin polymers that form the structural basis for tight junctions in higher eukaryotes (Staehelin, 1974; Lal‐Nag & Morin, 2009). Tetraspanner‐mediated PM domains are characterized by high spatiotemporal stability, interaction with defined lipids and partner proteins (Levy & Shoham, 2005; Zuidscherwoude *et al*, 2015), and their association with curved membrane regions (Bari *et al*, 2011; Dharan *et al*, 2022).

The budding yeast *Saccharomyces cerevisiae* has been used as a highly informative model for the study of PM domains (Spira *et al*, 2012; Schuberth & Wedlich‐Söldner, 2015). Two groups of yeast tetraspanner proteins, the Sur7 (Sur7, Pun1, Fmp45, Ynl194C, and Tos7) and Nce102 (Nce102 and Fhn1) families, show similarities to mammalian claudins and occludins, respectively. They are prominent components of a yeast PM domain known as the MCC/eisosome (Malínská *et al*, 2003; Walther *et al*, 2006). MCC/eisosomes are stable furrows, typically 200–400 nm long, 30–50 nm wide, and 50–100 nm deep (Strádalová *et al*, 2009; Douglas & Konopka, 2014; Lee *et al*, 2015). The term “MCC” (Membrane Compartment occupied by Can1) is derived from the association of the arginine permease Can1, whereas “eisosomes” are peripherally associated protein complexes built around two BAR (Bin, Amphiphysin, and Rvs) domain proteins—Pil1 and Lsp1. Together, these proteins assemble into a multimeric coat on the inner surface of the PM in a phosphatidylinositol‐4,5‐bisphosphate (PIP2) dependent manner (Moreira *et al*, 2009; Karotki *et al*, 2011). This coat promotes the negative curvature and inward flexure, which gives rise to the characteristic half‐pipe shaped furrow that marks this domain as a unique topographic environment within the yeast PM.

MCC/eisosomes and their components have been linked to a variety of biological functions, including the protection of nutrient transporters from endocytic internalization (Busto & Wedlich‐Söldner, 2019), lipid homeostasis (Young *et al*, 2002; Fröhlich *et al*, 2014), and cell wall synthesis (Alvarez *et al*, 2008; Wang *et al*, 2016; Lanze *et al*, 2020). Multiple links between the local lipid composition and MCC/eisosome function have been proposed. In addition to the role of PIP2 in eisosome assembly (Fröhlich *et al*, 2014), ergosterol has been shown to be enriched within MCC/eisosomes and several eisosomal components such as Nce102, Pkh1/2, and Slm1/2 have been suggested to regulate sphingolipid homeostasis (Walther *et al*, 2006; Grossmann *et al*, 2007; Luo *et al*, 2008; Fröhlich *et al*, 2009, 2014; Aguilar *et al*, 2010). Like many other BAR domain proteins (Frost *et al*, 2009), purified Pil1 and Lsp1 are known to self‐assemble into helical structures that induce formation of tubes with diameters of 30–40 nm from artificial membranes (Karotki *et al*, 2011). How cells modulate MCC/eisosomal activity to generate stable PM furrows rather than tubes remains unclear (Lanze *et al*, 2020).

In this study, we demonstrate that Sur7 tetraspanner proteins play a key role in modulating the topography of MCC/eisosomes. Using a combination of total internal reflection fluorescence microscopy (TIRFM), stimulated emission depletion (STED) microscopy (Hell & Wichmann, 1994) and freeze–fracture electron microscopy (EM), we show that Sur7 family proteins form multimeric strands along the upper edges of MCC/eisosome furrows. These strands modulate the forces exerted by the BAR domain coat at the furrow base, and thus prevent membrane tubulation. Deletion of Sur7 tetraspanners as well as lateral Sur7‐displacement due to altered cellular PIP2 homeostasis or a reduction of PM tension led to increased PM invaginations and the formation of distinctive half‐toroidal membrane tubes. Importantly, all defects associated with PIP2 overproduction could be rescued by synthetic anchoring of Sur7 to furrows, indicating that Sur7 displacement is a key consequence of PIP2 unbalance. Finally, we explored the requirements for formation of membrane furrows or tubes using simulations of model membranes.

Our findings suggest that the local topography of the yeast PM results from an intricate interplay between cooperatively acting BAR domain‐containing proteins and tetraspanner assemblies.

## Results

### Spatial organization of tetraspanner‐rich domains in the yeast PM

**Figure 1:**
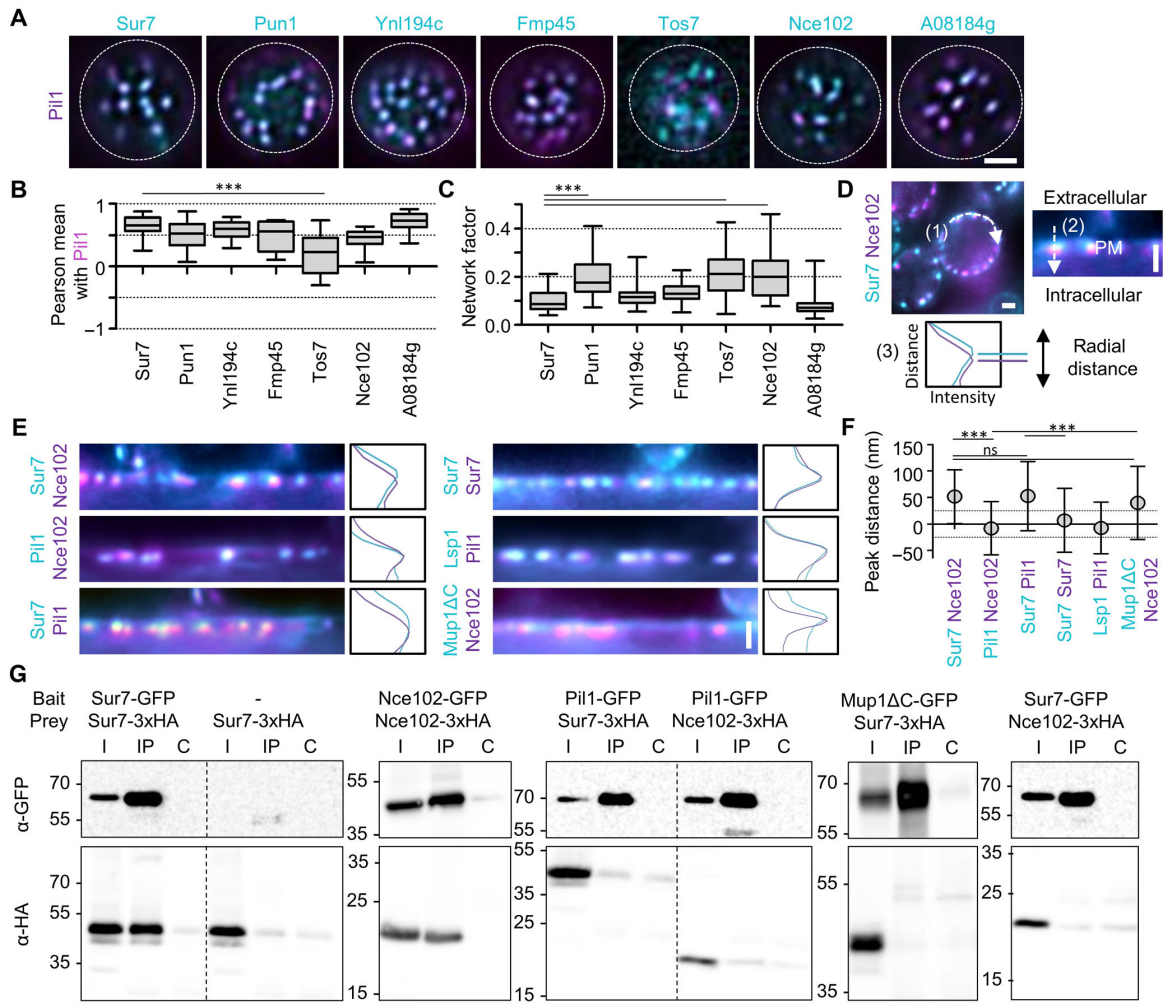
Yeast tetraspanners define subdomains in MCC/eisosomes TIRFM images showing colocalization of the indicated tetraspanners (C‐terminally fused to GFP (cyan) and expressed from the *PMA1* promoter) together with the MCC/eisosomes marker Pil1‐RFP (magenta).Pearson correlation coefficients for colocalization pairs shown in (A).Lateral distribution of tetraspanners from (A) quantified using the network factor.Representative linearized profiles were generated along the cell periphery (1). An exemplary two‐color intensity profile (3) was taken perpendicularly through a single MCC/eisosome, indicated by a dashed arrow (2). Distances between intensity peaks correspond to radial distances between indicated proteins.Radial fluorescence intensity distributions of MCC/eisosomal protein pairs and representative intensity profiles perpendicular to MCC/eisosomes. Proteins were endogenously fused at their C‐termini to mNeGr (cyan) or mRFPruby (magenta). Note that the linearized profile for Sur7‐mNeGr/Nce102‐RFP is taken from the same cell as in (D).Radial distances for indicated protein pairs shown in (E). Dotted lines indicate the 25 nm bracket representing the estimated resolution of peak fitting.Co‐Immunoprecipitation of tetraspanners with various targets. Indicated GFP‐tagged proteins (bait) were pulled down with anti‐GFP. GFP‐tagged bait proteins and HA‐tagged prey proteins were detected by Western blot. I: Input, IP: Co‐IP with anti‐GFP, C: Control IP with unspecific IgG. Data information: (B, C) Boxplots (interquartile range (box), min to max spread (whiskers) and median values (line in boxes)), ANOVA with Dunnett's multiple comparison test, *n* = 15–32 cells from three experiments (B), *n* = 41–88 cells from three experiments (C). (F) Symbol graph, ANOVA with Tukey's multiple comparison test, *n* > 143 MCC/eisosomes from three experiments. *P*‐values: ***P < 0.01, ns: not significant. Scale bars: 1 μm. Source data are available online for this figure.

**Figure EV1:**
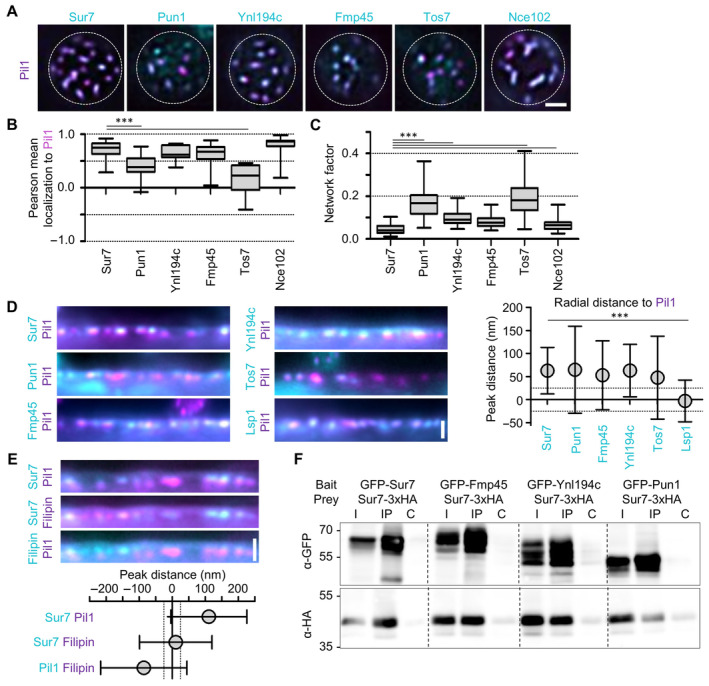
Yeast tetraspanners define subdomains in MCC/eisosomes—Supplement TIRFM images showing colocalization of indicated tetraspanners (C‐terminally endogenously fused to GFP (cyan)) with the MCC/eisosome marker Pil1‐RFP (magenta).Pearson correlation coefficients for colocalization shown in (A).Lateral distribution of tetraspanners from (A) quantified using the network factor.Linearized profiles of WT cells expressing endogenous mNeGr fusions to indicated Sur7 tetraspanners and Lsp1 (cyan) together with Pil1‐RFP (magenta). Symbol graph represents quantification of radial distances between indicated protein pairs. Dotted lines indicate the 25 nm bracket representing the estimated resolution of peak fitting.Linearized profiles of WT cells expressing Sur7‐GFP from the *PMA1* promoter (cyan) and Pil1‐RFP (magenta) stained with 5 μg/ml filipin (magenta/cyan). Symbol graph represents quantification of radial distances between indicated pairs. Dotted lines indicate the 25 nm bracket representing the estimated resolution of peak fitting.Co‐Immunoprecipitation of different Sur7 tetraspanner pairs. The indicated GFP‐tagged bait proteins were overexpressed from the GPD promoter and were pulled down with anti‐GFP antibody. The prey protein Sur7‐3xHA was expressed under the *PMA1* promoter. Western blot probed with antibodies directed against HA and GFP. I: Input, IP: Co‐IP with anti‐GFP, C: Control IP with unspecific IgG. Data information: (B, C) Boxplots (interquartile range (box), min to max spread (whiskers) and median values (line in boxes)), ANOVA with Dunnett's multiple comparison test, *n* = 29–49 cells from three experiments (B), *n* = 59–150 cells from three experiments (C). (D, E) Symbol graph (error bars: SD), ANOVA with Tukey's multiple comparison test, *n* > 119 (D) and *n* > 152 (E) measurements from three experiments each. *P*‐values: ***P < 0.01. Scale bars: 1 μm. Source data are available online for this figure.

To characterize the detailed organization of the MCC/eisosome domains we initially made use of two‐color TIRFM to visualize all seven domain‐resident tetraspanners, and compared their distributions to that of Pil1, the BAR domain protein responsible for the formation of the furrow. The Nce102 paralog Fhn1 was not expressed under our growth conditions and was therefore not included in further comparisons. As an additional control we also determined localization for the Sur7 ortholog A08184g from *Kluyveromyces lactis* in our cells. All tetraspanners co‐localized with Pil1, either when expressed at endogenous levels (Fig EV1A and B) or when expressed from the strong *PMA1* promoter (Fig 1A and B). While most Sur7 tetraspanners were exclusively concentrated in MCC/eisosomes, Pun1, and Tos7 were also detected throughout the PM irrespective of their expression level, as reflected by their higher network factors (Figs 1C and EV1C). This parameter describes the density of signal distribution for PM proteins (Spira *et al*, 2012). In contrast, Nce102 was exclusively concentrated in MCC/eisosomes under normal conditions (Fig EV1C) but its distribution became more dispersed when overexpressed (Fig 1C). Therefore, labeling of Nce102 was performed exclusively on the endogenous level in all further experiments, whereas the expression of Sur7 tetraspanners could be modulated according to the needs to ensure robust comparability of our analysis.

To determine the precise positions of tetraspanners within MCC/eisosome furrows, we performed high‐resolution radial distance measurements of medial cell sections derived from conventional epifluorescence data (Fig 1D). Using this method, we found that all six tested members of the Sur7 family were located approximately 50 nm “outside” of Nce102, Pil1, and Lsp1 (Figs 1E and F, and EV1D). This likely reflected localization at different furrow depths, with Sur7 proteins being closest to the surface. As a representative member of the MCC/eisosome‐associated symporters, the methionine permease Mup1 exhibited a similar radial distribution to that of Sur7 proteins (Fig 1E and F). Note, that we show the distribution of a C‐terminal deletion variant of Mup1, Mup1ΔC, which exhibits improved clustering within MCC/eisosomes (Busto *et al*, 2018). Finally, the sterol marker filipin that was previously reported to concentrate in MCC/eisosomes (Grossmann *et al*, 2007) was also localized at the outer periphery of the furrow, together with Sur7 (Fig EV1E).

To test whether the radial colocalization of the tested markers might reflect actual physical interactions, we next performed co‐immunoprecipitation experiments. We found strong interactions among the Sur7 family members and of Nce102 with itself (Figs 1G and EV1F). Despite their spatial proximity, we found no evidence of strong physical interactions between Nce102 and Pil1 or between Mup1 and Sur7 (Fig 1G).

### Tetraspanners and BAR domain proteins localize to distinct MCC/eisosome subdomains

While we could observe a significant and robust separation of two distinct subdomains in radial representations of MCC/eisosomes, the precise relationship between these domains was obscured by the limited resolution of conventional fluorescence microscopes. To improve the visualization of subdomains we therefore turned to super‐resolution STED microscopy and freeze–fracture EM.

**Figure 2:**
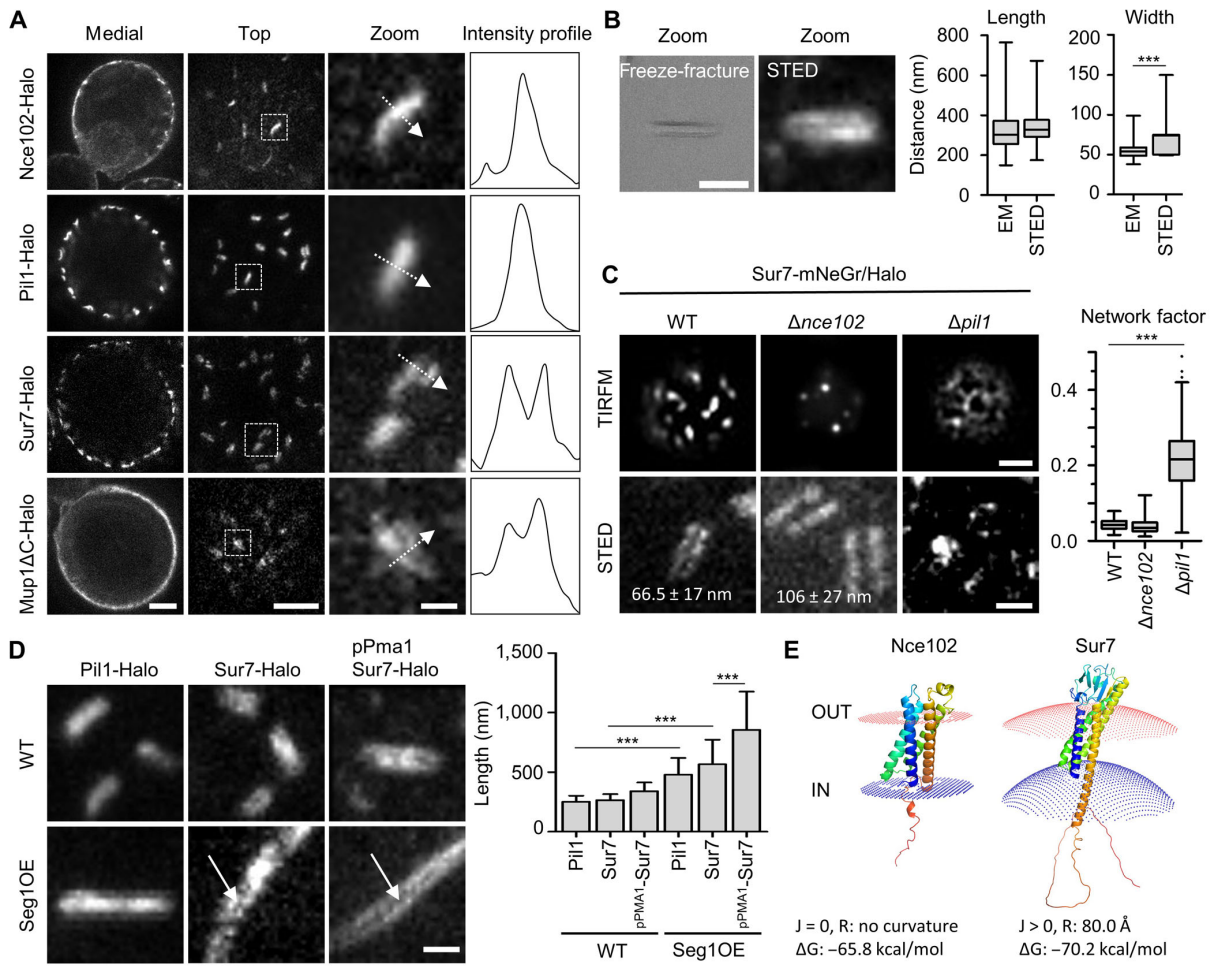
Super‐resolution imaging of MCC/eisosome subdomains STED images of indicated proteins fused to the Halo tag. Intensity profiles correspond to the dotted arrows in the zoomed images.Comparison of MCC/eisosome furrow dimensions obtained from freeze–fracture EM replicas and STED images of a yeast strain expressing Sur7‐Halo under the *PMA1* promoter.Representative STED‐ and TIRFM‐images of yeast WT, Δ*nce102*, and Δ*pil1* cells expressing Sur7‐mNeGr (TIRFM) or Sur7‐Halo (STED); the TIRFM images were used to obtain network factors in the box plot. Numbers in STED images indicate average ± SD gap width between Sur7‐Halo strands in WT and Δ*nce102* cells.Representative STED images of WT and Seg1 overexpressing (OE) yeast cells expressing either endogenous Pil1‐Halo or Sur7‐Halo or overexpressing Sur7‐Halo from the *PMA1* promoter. Arrows highlight double‐strand appearance of Sur7‐tetraspanners. Quantification shows MCC/eisosomal length upon Seg1 overexpression.Positioning of Nce102 and Sur7 in fungal plasma membranes using PPM 3.0. Sign of curvature (J), radius of intrinsic curvature (R) and binding energy to membrane (ΔG) are indicated. Data information: (B, C) Box plots (interquartile range (box), min to max spread (whiskers) and median values (line in boxes)), unpaired *t*‐tests, *n* > 150 MCC/eisosomes from three experiments. (D): Bar graph (error bars: SD), ANOVA with Tukey's multiple comparison test, *n* > 90 MCC/eisosomes from three experiments. *P*‐values: ***P < 0.01. Scale bars: 1 μm, 200 nm (zoom). Source data are available online for this figure.

**Figure EV2:**
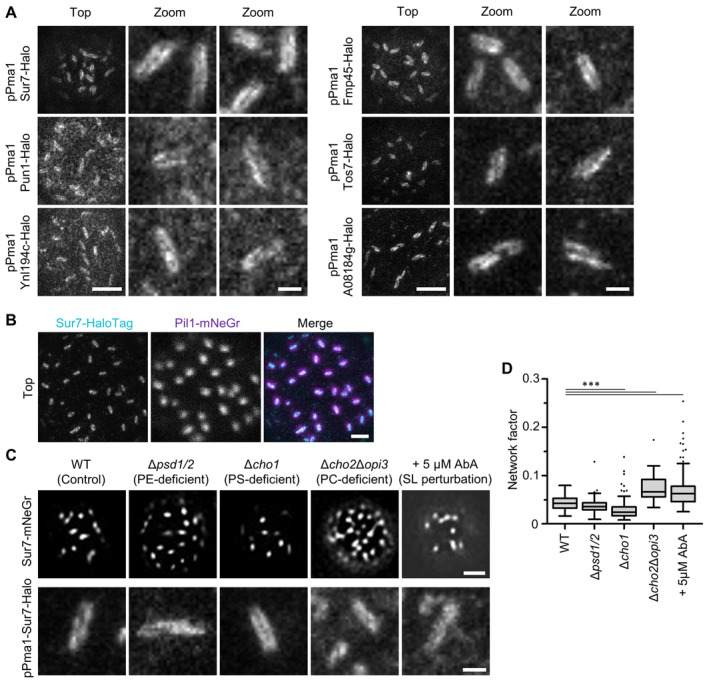
Super‐resolution imaging of Sur7 tetraspanners—Supplement STED images of indicated tetraspanners expressed from the *PMA1* promoter and fused to the Halo tag.Two‐color STED and confocal images of WT cells expressing endogenously tagged Sur7‐Halo (STED, magenta) and Pil1‐mNeGr (confocal, cyan).Effect of indicated lipid perturbations on lateral distribution of Sur7‐mNeGr.Lateral distribution of Sur7‐mNeGr quantified by the network factor in strains shown in (C). Data information: (D) Boxplot (interquartile range (box), min to max spread (whiskers) and median values (line in boxes)), ANOVA with Dunnett's multiple comparison test, *n* = 30–227 cells from three experiments. *P*‐values: ***P < 0.01. Scale bars: 1 μm, 200 nm (zoom). Source data are available online for this figure.

We initially inspected surface sections of cells to determine the lateral localization profiles of key marker proteins for MCC/eisosomes expressed at endogenous levels. The two markers that localized in a more central radial position, Pil1 and Nce102, were found to form single linear strands of around 300 nm in length (Fig 2A). In contrast, the more peripherally located Sur7 and Mup1 exhibited a characteristic double‐strand appearance (Fig 2A) with 300 nm length and intra‐strand gaps of around 60 nm (Fig 2B). These values were strikingly similar to the width and length of MCC/eisosomes observed in EM images of freeze–fracture replicas (Fig 2B). Overexpressed Sur7 family members exhibited identical double‐stranded patterns (Fig EV2A). By combining STED microscopy of Sur7‐HaloTag (Halo) with confocal imaging of Pil1‐mNeonGreen (mNeGr) we confirmed that Sur7 strands laterally overlapped with Pil1‐labeled structures and thus indeed represented the Sur7 distribution in MCC/eisosomes (Fig EV2B).

As shown previously (Walther *et al*, 2006), deletion of Pil1 led to a complete loss of MCC/eisosome‐related structures. In addition to the previously reported eisosomal remnants (Walther *et al*, 2006), Sur7 was distributed over the whole PM and formed small clusters of less than 100 nm diameter (Fig 2C). This was also apparent from an increased network factor in TIRFM images (Fig 2C). Deletion of Nce102 led to the reported reduction of MCC/eisosome number (Walther *et al*, 2007) and to an increased separation for Sur7 strands of over 100 nm in the remaining MCC/eisosomes (Fig 2C). On the other hand, overexpression of the eisosomal regulator Seg1, which is involved in the initiation of eisosome formation (Moreira *et al*, 2012), led to the formation of much longer structures that retained the characteristic appearance of the respective markers (Fig 2D). Elongated Sur7 strands in Seg1 overexpressing cells (Seg1OE) remained continuous along both lateral surfaces (Fig 2D), similar to the appearance in WT MCC/eisosomes (Figs 2A and EV2A). Strand separation was easier to distinguish upon Sur7 overexpression, but also led to an increase in Sur7 strand length (Fig 2D). Finally, removal of abundant lipid classes present in the PM, such as PE, PS, PC, or sphingolipids, only led to minor effects in MCC/eisosome localization of Sur7 or the double‐strand appearance (Fig EV2C and D).

To test whether the strand formation of Sur7 tetraspanners reflected a preference for a particular membrane topography we modeled the curvature preference for different tetraspanners within MCC/eisosome components using the PPM 3.0 web server (Lomize *et al*, 2022). While Nce102 which is located in the lower part of the furrow (Fig 1F) had no curvature preference, Sur7 at the upper furrow edges was predicted to prefer very strong positive curvature with an intrinsic radius of 80 Å (Fig 2E).

Taken together, our analysis combining regular epifluorescence, TIRF, STED, and freeze–fracture electron microscopy demonstrates that MCC/eisosomes exhibit lateral and radial separation of membrane‐integral tetraspanner proteins and peripheral BAR domain proteins into nanometer‐scale domains with distinct curvatures.

### Sur7 tetraspanners modulate BAR domain‐induced PM topography in MCC/eisosomes

**Figure 3:**
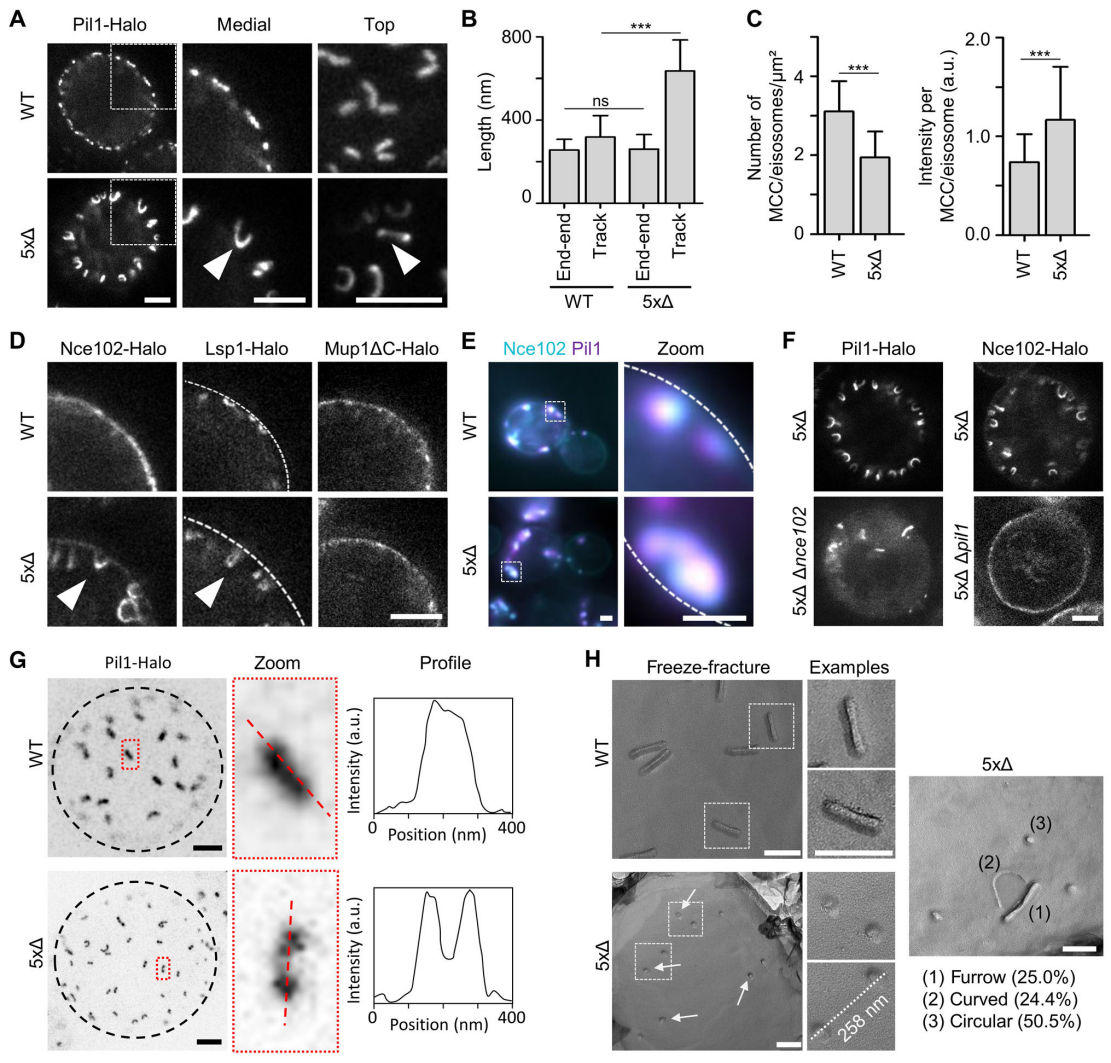
Sur7 tetraspanners prevent closure of MCC/eisosome furrows STED images of yeast cells expressing Pil1‐Halo in control (WT) and in mutant cells depleted of all five Sur7 tetraspanners (5xΔ). Arrowheads indicate tubular invagination.Quantification of end‐to‐end distance (from top views) and track‐length (from medial views) of Pil1‐Halo marked structures in yeast WT or 5xΔ cells.Density (number per area) and intensity of MCC/eisosomes marked by Pil1‐Halo.STED images of yeast WT and 5xΔ cells expressing indicated Halo fusions. Arrowheads highlight tubular structures in 5xΔ cells. Dashed line indicates location of the PM.Colocalization of Pil1‐mRFPruby and Nce102‐mNeGr in tubular structures of 5xΔ cells. Dotted lines indicate the cell edge.STED images of 5xΔ and 6xΔ (5xΔ + Δ*pil1* or Δ*nce102*) cells expressing indicated Halo fusions.Top view STED images of WT or 5xΔ cells expressing Pil1‐Halo. Dotted black line indicates the cell edge. Intensity profiles of linear structures taken along dotted lines in zoomed areas.Freeze–fracture images of the PM in WT and 5xΔ cells. Arrows indicate likely ends of tubular invaginations. The distance between two exemplary circular structures and percentage of identified structural categories (1–3) in 5xΔ cells are shown. Data information: (B, C) Bar graphs (error bars: SD), unpaired *t*‐test, *n* > 44 cells from three experiments. ***P < 0.01, ns: not significant. Scale bars: 1 μm (A, D–F) and 200 nm (G, H). Source data are available online for this figure.

**Figure EV3:**
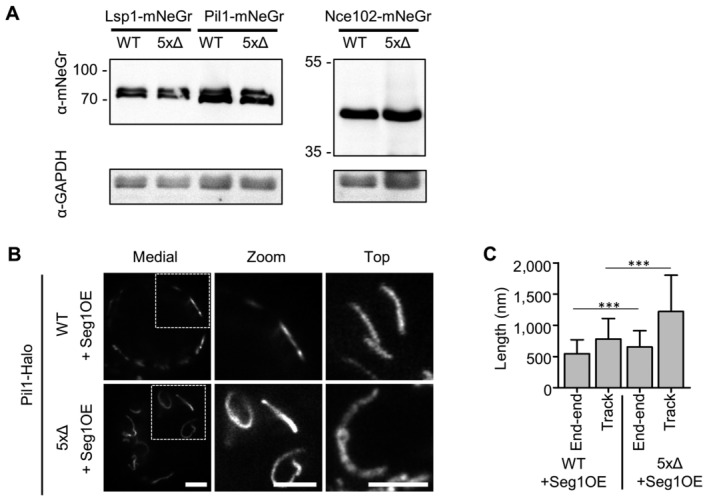
Sur7 tetraspanners prevent closure of MCC/eisosome furrows—Supplement Western blot analysis depicting the expression of endogenous mNeGr fusions to Lsp1, Pil1 and Nce102 in WT and 5xΔ cells.STED microscopy of endogenously tagged Pil1‐Halo in WT and 5xΔ cells that overexpress Seg1 from the GPD promoter (OE).Quantification of track length for Pil1‐Halo positive structures in (B). Data information: (C) Bar graph (error bars: SD), unpaired *t*‐test, *n* = 53–175 tracks from three experiments. *P*‐values: ***P < 0.01. Scale bars: 1 μm. Source data are available online for this figure.

Having established the subdomain organization of MCC/eisosomes, we turned to the role of Sur7 tetraspanners and the parallel Sur7 strands in shaping the local PM topography. We generated a yeast strain lacking all five of the Sur7 family members previously identified in MCC/eisosomes (Fig 1). Strikingly, in STED images of this 5xΔ mutant (Δ*sur7*, Δ*pun1*, Δ*fmp45*, Δ*ynl194c*, and Δ*tos7*), Pil1 localized to distinctive inwardly curved toroidal tubes that were connected with the cell perimeter at both ends (Fig 3A). Importantly, while individual tubes had track lengths of around 600 nm, their end‐to‐end distance of around 250 nm was comparable to the length of normal MCC/eisosome furrows (Fig 3B). The density of tubes was lower than for MCC/eisosomes in control strains but individual tubes exhibited higher Pil1 fluorescence intensities (Fig 3C), indicating that Pil1 molecules were redistributed into fewer but longer structures. An overall conservation of Pil1, Lsp1, and Nce102 levels was confirmed by comparison of protein expression levels (Fig EV3A). We next verified the localization of other MCC/eisosomal markers. Lsp1 and Nce102 were detected within the tube structures, while Mup1 was largely excluded (Fig 3D). We confirmed co‐localization of Nce102 and Pil1 within tubular structures by epifluorescence imaging (Fig 3E) and found that tube formation was largely abolished in the absence of either component (Fig 3F).

To further verify if the Pil1‐positive tubes have the same origin as MCC/eisosomes we overexpressed the proposed scaffold protein Seg1 responsible for MCC/eisosome length (Moreira *et al*, 2012) in the 5xΔ background. We again observed pronounced tube‐like structures that despite their often distorted shape mostly remained connected to the PM at their ends (Fig EV3B). Importantly, the end‐to‐end distance of these structures was comparable to the increased furrow‐length of MCC/eisosomes in WT cells overexpressing Seg1 (Fig EV3C). These results indicate that MCC/eisosomes and the toroidal tubes in 5xΔ cells both originate from the same Seg1‐scaffolded structure.

**Figure EV4:**
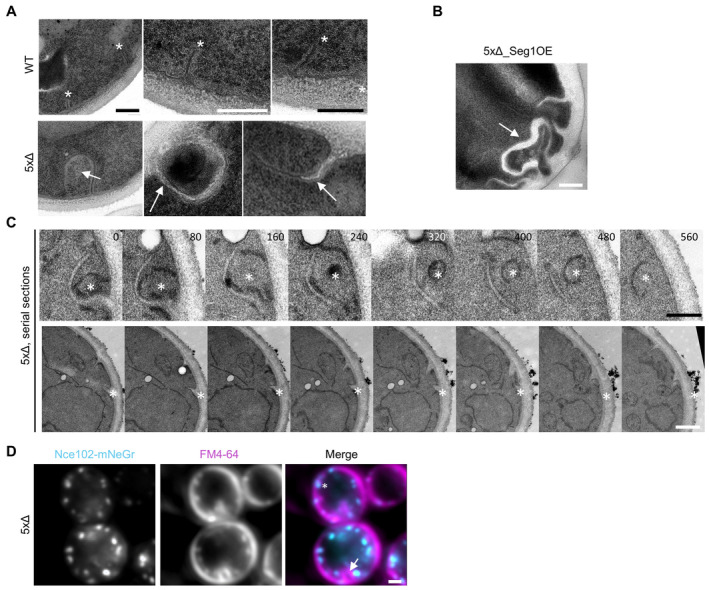
Sur7 tetraspanners prevent closure of MCC/eisosome furrows – Supplement TEM micrographs of ultrathin sections of WT and 5xΔ. Asterisks indicate perpendicular sections through MCC/eisosome furrows. Arrows indicate abnormal PM invaginations.TEM micrograph of ultrathin sections of a 5xΔ cell overexpressing Seg1 from the GPD promoter (OE). The arrow indicates an abnormal PM invagination.Exemplary serial TEM sections of 5xΔ cells illustrating the sheet‐like invaginations filled with electron translucent cell wall material. Asterisks indicate positions of invaginations. Sample depth is indicated in nm.5xΔ cells expressing Nce102‐mNeGr (cyan) and labeled with the membrane dye FM4‐64 (magenta). Asterisk indicates a Nce102‐positive tube that is labeled by FM4‐64. Arrow indicates a large FM4‐64 positive invagination that does not contain Nce102. Data information: Scale bars: 200 nm (A–C), 1 μm (D). Source data are available online for this figure.

To verify whether the observed structures indeed corresponded to half‐toroidal membrane tubes, we compared their nano‐architecture in STED microscopy and freeze–fracture EM. While top view STED images of WT MCC/eisosomes show a continuous labeling with Pil1 along their long axes (Fig 3G), linear structures in 5xΔ cells exhibited much stronger intensities at their ends, consistent with inwardly bent tubes that are covered by a Pil1 coat (Fig 3G). Freeze–fracture replicas showed the typical 300 nm long furrows of MCC/eisosomes in the WT PM with consistently higher curvature at the lateral edges versus furrow tips (Fig 3H). In the PM of 5xΔ cells, we observed a smaller amount of straight furrows (~25%), an equal number of curved invaginations (~25%) and a majority of smaller circular structures (~50%) (Fig 3H). The distance of 200–300 nm between these circles fits well to the length of MCC/eisosomes (Strádalová *et al*, 2009) and to our STED images (Fig 3B and G). The circle diameter of around 40 nm also fits the expected range seen for Pil1‐derived membrane tubes *in vitro* (Karotki *et al*, 2011). Finally, we attempted to confirm the formation of membrane tubes in 5xΔ cells using transmission EM of ultrathin sections. We could identify the expected MCC/eisosome invaginations in WT cells (Fig EV4A, asterisks) and found distinctive membrane tubes that extended far into the cell (Fig EV4A, arrows). Many of these tubes were filled with electron translucent material that was reminiscent of cell wall material (Fig 3H) as also seen in Δ*sur7* cells of *C. albicans* (Alvarez *et al*, 2008). This phenotype was especially apparent in Seg1 overexpression conditions where tubes were much longer and convoluted (Fig EV4B). However, in serial sections we found that all visible invaginations presumably filled with cell wall material extended over several 100 nm and represented sheets or cup‐like invaginations rather than closed tubes (Fig EV4C). In epifluorescence images, we also found that 5xΔ cells exhibited membrane staining in Ne102‐positive tubes (Fig EV4D, asterisk) but also large PM invaginations that were labeled with FM4‐64 but not Nce102 (Fig EV4D, arrow). We suggest that the half‐toroidal tubes formed by Pil1 in 5xΔ cells were not detected in our transmission EM preparations because of sub‐optimal contrasting of the sample due to the cell wall (note that lipid bilayers cannot be well distinguished) and that in addition, large cell wall‐filled invaginations form in the tetraspanner mutant that are presumably not directly related to MCC/eisosomes.

Our results indicate that Sur7 tetraspanners play a major role in modulating PM topography by opposing Pil1‐mediated PM curvature and preventing the formation of toroidal membrane tubes.

### The function of Sur7 in shaping MCC/eisosomes requires its C‐terminal region

**Figure 4:**
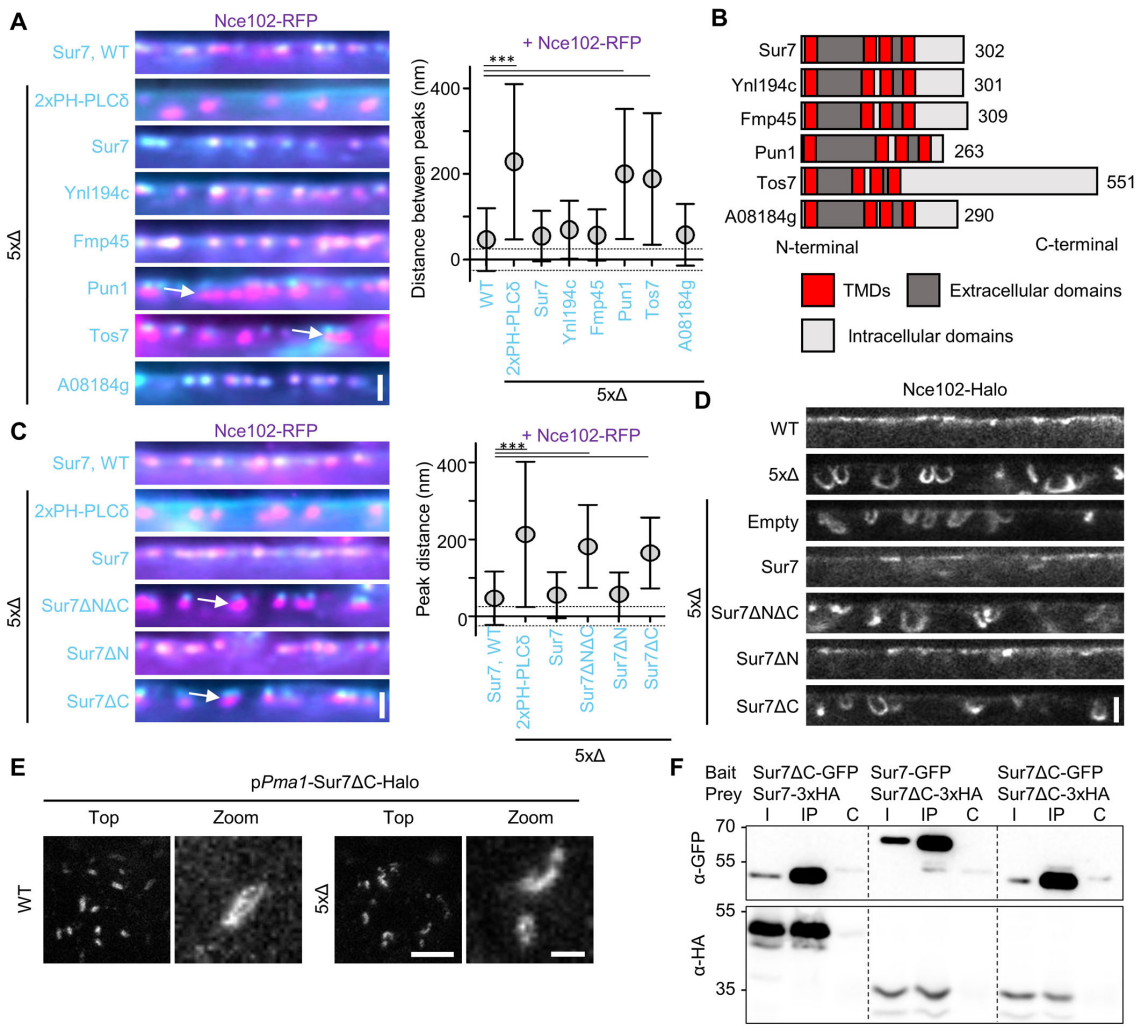
The C‐terminus of Sur7 is required for regulating PM domain topography Radial fluorescence intensity distribution of indicated protein pairs in linearized profiles of WT and 5xΔ cells expressing Nce102‐RFP (magenta) and GFP‐fused Sur7‐tetraspanners (cyan) from the *PMA1* promoter. 2xPH‐PLCδ was used as an empty control and marks the PM. Symbol graph indicates distances between radial profile peaks. Arrows indicate separation of tetraspanners. Dotted lines indicate the 25‐nm bracket representing the estimated resolution of peak fitting.Topology of different Sur7‐tetraspanner family members with transmembrane (TMDs, red), extracellular (dark gray), and intracellular (light gray) segments indicated.Radial fluorescence intensity distributions of protein pairs in WT and 5xΔ cells expressing Nce102‐RFP (magenta) and GFP‐fused Sur7‐truncations (cyan) from the *PMA1* promoter. 2xPH‐PLCδ was used as control. Symbol graph indicates distances between radial profile peaks. Sur7 = 1–302 aa; Sur7ΔC = 1–210 aa; Sur7ΔN = 7–302 aa; Sur7ΔNΔC = 7–210 aa. Arrows indicate separation of tetraspanners. Dotted lines indicate the 25 nm bracket representing the estimated resolution of peak fitting.Linearized STED profiles from 5xΔ cells expressing Nce102‐Halo and Sur7 truncations.STED images of WT and 5xΔ cells expressing Sur7ΔC‐Halo from the *PMA1* promoter.Co‐Immunoprecipitation of Sur7 truncations. Indicated GFP‐tagged proteins (bait) were pulled down with anti‐GFP. GFP‐tagged prey proteins and HA‐tagged prey proteins were detected by Western blot. I: Input, IP: Co‐IP with anti‐GFP, C: Control IP with unspecific IgG. Data information: (A, C) Symbol graphs (error bars: SD), ANOVA with Dunnett's multiple comparison test, *n* > 67 MCC/eisosomes from three experiments (A), *n* > 139 MCC/eisosomes from three experiments (C). *P*‐values: ***P < 0.01. Scale bars: 1 μm (A, C, E‐top), 500 nm (D), 200 nm (E‐zoom). Source data are available online for this figure.

To identify specific structural features in Sur7 tetraspanners that mediate their function in PM domain formation, we attempted to rescue the tubulation phenotype by overexpressing individual Sur7 family proteins in the 5xΔ strain (Fig 4A). The radial distance between Nce102 in tubes and the cell surface was greatly increased to over 200 nm in 5xΔ cells (Fig 4A, in the absence of Sur7 proteins Nce102 was compared to the PIP2‐binding PH domain of PLCδ: “2xPH”). We found that three members—Sur7, Fmp45, and Ynl194c—fully rescued the radial separation between Nce102 and the respective Sur7 tetraspanner (Fig 4A) to the typical 50 nm seen in control cells (Fig 4A). The single *K. lactis* Sur7 homolog A08184g was also able to fully rescue MCC/eisosome tubulation (Fig 4A). In contrast, the remaining members, Pun1 and Tos7 could not rescue PM topography (Fig 4A). Notably, these two proteins were the only two Sur7 variants that also localized to PM regions outside of MCC/eisosomes (Figs 1A and C, and EV1A and C). The largest differences between sequences of rescuing and non‐rescuing Sur7 family members are found in their C‐terminal regions (Fig 4B). Therefore, we repeated our rescue experiments with Sur7 variants lacking N‐ and/or C‐terminal regions. While deletion of the N‐terminus had no obvious effect on the rescue of tubulation, removal of the C‐terminus led to a complete loss of rescue activity (Fig 4C). STED microscopy confirmed that expression of Sur7 lacking the C‐terminus could not prevent the formation of tubes in the 5xΔ mutant (Fig 4D). These results are consistent with recent findings in *C. albicans*, which assigned a key function in regulating morphogenesis and stress responses to the Sur7 C‐terminus (Lanze *et al*, 2021). Importantly, despite its role in sculpting membranes, the Sur7 C‐terminus was not required for recruitment to MCC/eisosomes in WT cells and was still able to assemble into elongated strands in 5xΔ cells (Fig 4E). Consistently, Co‐IP analysis showed that (like Sur7) Sur7ΔC was able to interact with itself (Fig 4F).

In summary, the C‐terminal cytosolic segment of Sur7 plays a key part in the function of this tetraspanner in modulating membrane tubulation via the BAR domains of Pil1 and Lsp1.

### The role of PIP2 in PM organization

**Figure 5:**
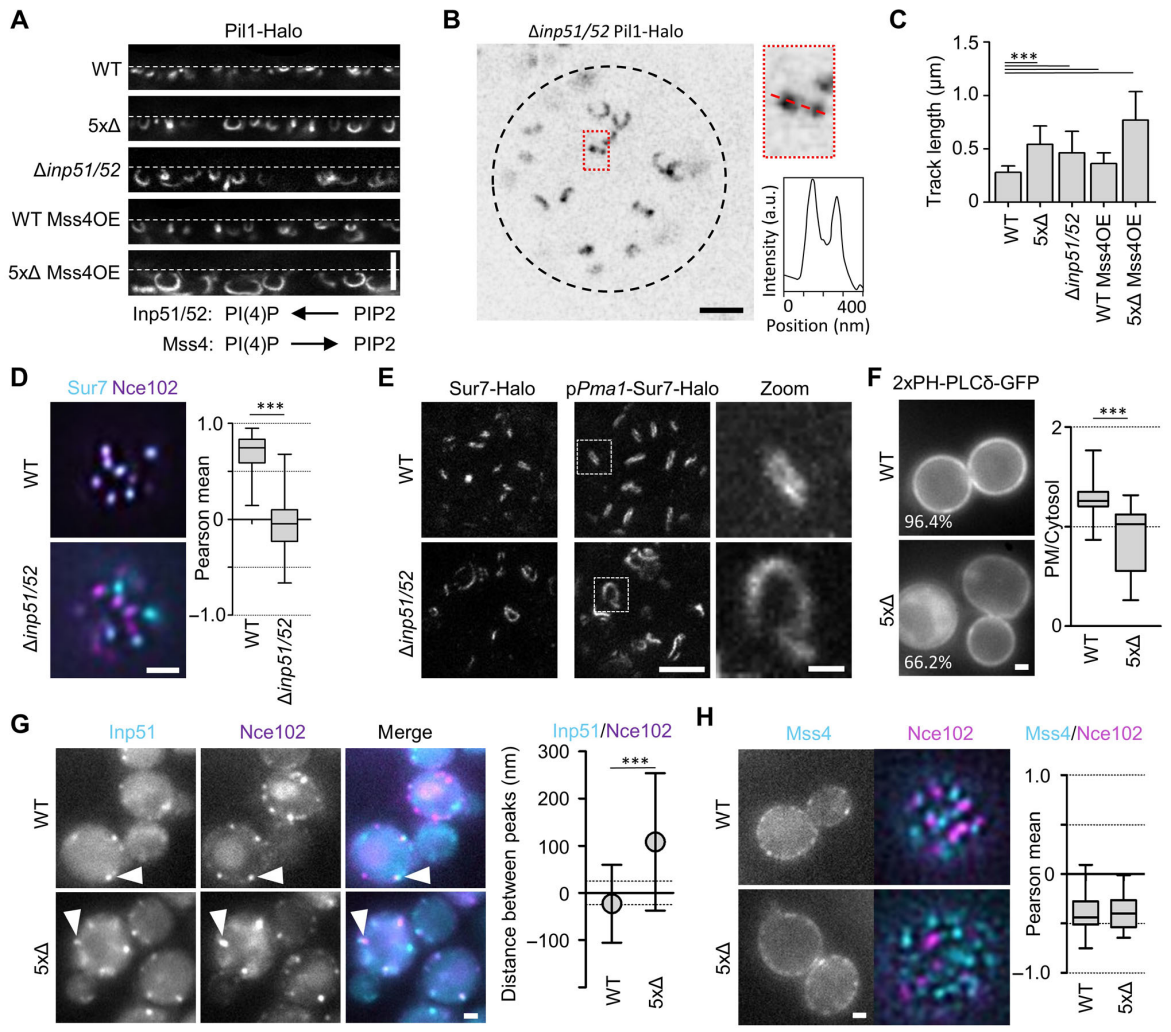
The role of PIP2 in the regulation of MCC/eisosome topography Linearized STED profiles of indicated mutants expressing Pil1‐Halo. Mss4 was constitutively overexpressed from the GPD promoter. Dotted lines indicate PM. Function of indicated enzymes in PI(4)P‐ and PIP2‐biosythesis is displayed schematically.Top view STED image of a Δ*inp51/52* cell expressing Pil1‐Halo. Dotted black line indicates the cell edge. Intensity profile of linear structure taken along dotted line in zoomed area.Bar graphs indicate track lengths of tubular invaginations formed under the conditions shown in (A).Colocalization of Sur7‐mNeGr (cyan) and Nce102‐RFP (magenta) in MCC/eisosomes from WT and Δ*inp51/52* cells. Box plots indicate the respective Pearson mean coefficients.STED images of Sur7‐Halo expressed endogenously or from the *PMA1* promoter in WT and Δ*inp51/52* cells. Zoomed images show details of Sur7 strand organization.Distribution of the PIP2 reporter 2xPH‐PLCδ‐GFP in WT and 5xΔ cells. Percentage of cells that showed PM staining is indicated. Box plots show PM to cytosol ratios of GFP signal.Colocalization of Inp51‐mNeGr (cyan) and Nce102‐RFP (magenta) in MCC/eisosomes (arrowheads) of WT and 5xΔ cells. Symbol graph indicates distances between radial profile peaks.Localization of endogenously tagged GFP‐Mss4 (cyan) in WT and 5xΔ cells expressing Nce102‐RFP (magenta). Box plots indicate the respective Pearson mean coefficients. Data information: (C) Bar graph (error bars: SD), ANOVA with Dunnett's multiple comparison test, *n* = 139–292 tracks from three experiments. (D) Boxplot (interquartile range (box), min to max spread (whiskers) and median values (line in boxes)), unpaired *t*‐test, *n* = 82–93 cells from three experiments. (F) Boxplot, unpaired *t*‐test, *n* > 62 cells from three experiments. (G) Symbol graph (error bars: SD), unpaired *t*‐test, *n* > 104 measurements from three experiments. (H) Boxplot, unpaired *t*‐test, *n* > 26 cells from three experiments. *P*‐values: ***P < 0.01. Scale bars: 1 μm and 200 nm (zoom). Source data are available online for this figure.

As shown above, the major phospholipids and sphingolipids present in the yeast PM were not directly involved in the assembly of Sur7 strands at the upper edge of MCC/eisosomes (Fig EV2C and D). However, we did not yet test one specific type of lipid that has previously been implicated in MCC/eisosome biogenesis (Fröhlich *et al*, 2014). Since PIP2 is essential for yeast cells, we tested how increased PIP2 levels affect subdomain organization of MCC/eisosomes. In agreement with previous reports (Singer‐Krüger *et al*, 1998; Stefan *et al*, 2002; Karotki *et al*, 2011), we found that deletion of the major PIP2 phosphatases Inp51 and Inp52 induced the formation of aberrant PM invaginations. Using the improved resolution provided by STED microscopy, we found Pil1‐positive tubes that closely resembled those observed in the 5xΔ strain in appearance (Fig 5A and B) and size (Fig 5C). To a lesser degree, tubes were also formed when increasing PIP2 levels by overexpressing the yeast PI(4)P‐5 kinase Mss4 (Fig 5A and C, Mss4OE). Strikingly, increased PIP2 levels and deletion of all five Sur7 family members exhibited synergistic effects on tube length (Fig 5A and C). These results suggest a close link between cellular PIP2 levels and PM domain topography driven by Sur7 tetraspanners. We therefore tested, whether PIP2 perturbations had a direct effect on the localization of Sur7. Using TIRFM we found that Sur7 was in fact displaced from Nce102‐labeled MCC/eisosomes under conditions in which PIP2 levels were increased (Fig 5D). More specifically, upon deletion of INP51 and INP52, Sur7 parallel strands imaged by STED microscopy were absent and the tetraspanners instead formed abnormally shaped linear structures within the yeast PM—irrespective of Sur7 expression levels (Fig 5E).

We also wanted to observe whether deletion of Sur7 tetraspanners in turn affected PIP2 levels at the PM. Indeed, 5xΔ cells exhibited strongly reduced PM recruitment of the PIP2 probe 2xPH(PLCδ)/GFP with cells instead accumulating the probe in the cytosol (Fig 5F).

One molecular mechanism linking MCC/eisosomes and PIP2 homeostasis involves the direct interaction of the major PIP2 phosphatase Inp51 with Pil1 (Fröhlich *et al*, 2014). Consistent with a direct Pil1‐Inp51 interaction radial profiles of dynamic Inp51 patches closely matched those of Nce102, characteristic for the bottom of MCC/eisosome furrows (Fig 5G). Interestingly, this association was lost in 5xΔ cells, where Inp51 was found at the cell periphery, clearly separate from Nce102 (Fig 5G). In contrast, the PI(4)P‐5 kinase Mss4 did not localize to MCC/eisosomes and its distribution was not altered in 5xΔ cells (Fig 5H).

Our results indicate close links between PIP2 homeostasis, Sur7 localization and local PM topography in MCC/eisosomes.

### Mechanistic basis of Sur7 function in subdomain organization

**Figure 6:**
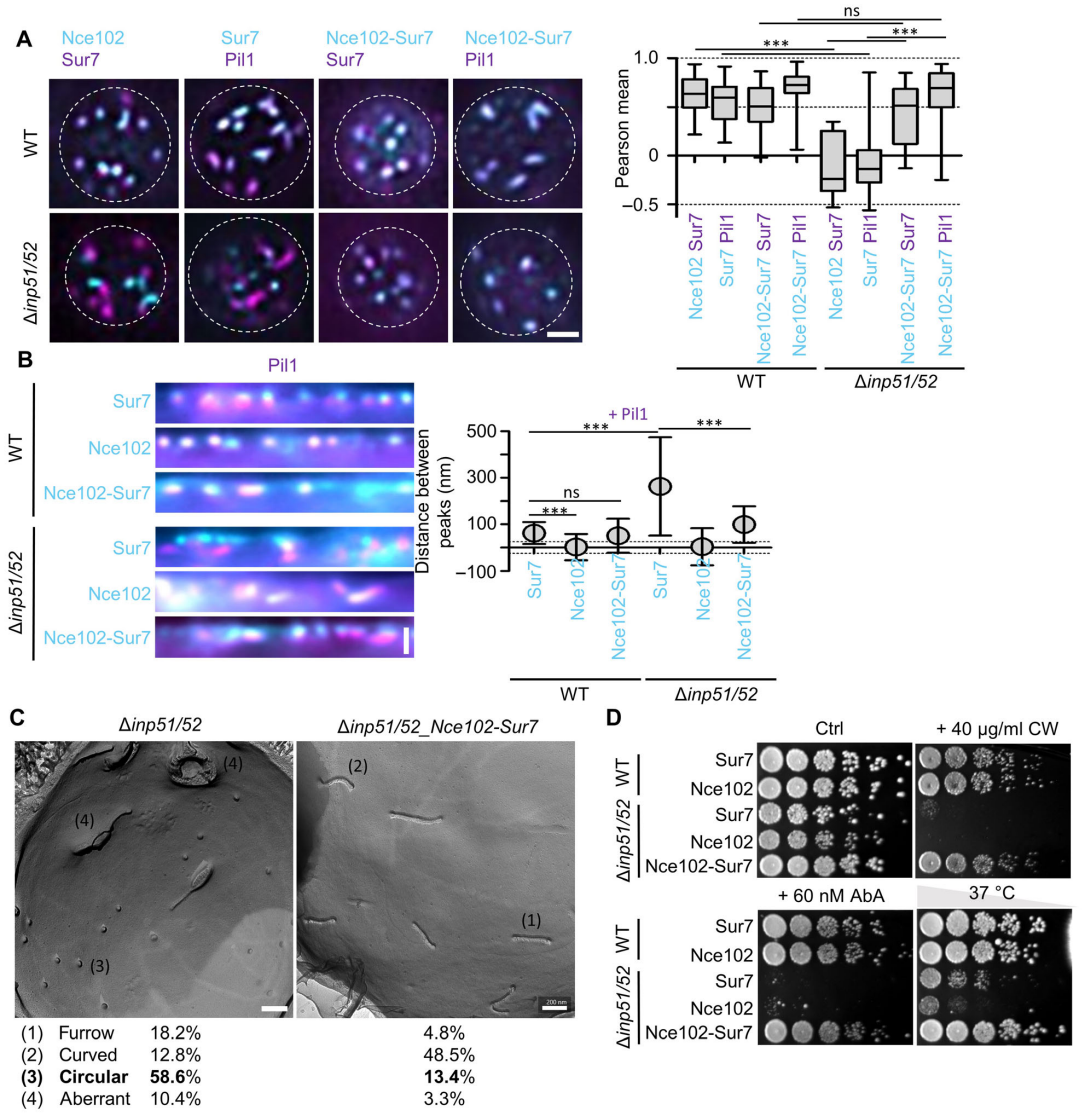
Synthetic recruitment of Sur7 controls the topography and function of MCC/eisosomes Colocalization of mNeGr fusions to Sur7, Nce102 or the Nce102‐Sur7‐chimera (cyan) with Sur7‐RFP or Pil1‐RFP (magenta) in WT and Δ*inp51/52* cells. Box plot shows Pearson correlation coefficients for the respective protein pairs.Linearized profiles of WT and Δ*inp51/52* expressing mNeGr fused to Sur7, Nce102 or the Nce102‐Sur7‐chimera (cyan). MCC/eisosomes are marked with Pil1‐RFP (magenta). Symbol graph of distances between radial profile peaks of Pil1‐RFP and shown proteins. Dotted lines indicate the 25 nm bracket representing the estimated resolution of peak fitting.Freeze–fracture EM micrographs of the PM in a Δ*inp51/52* cell and a Δ*inp51/52* cell expressing the Nce102‐Sur7‐mNeGr‐chimera. Percentages for identified structural categories (1–4) are shown.Growth assay for WT or Δ*inp51/52* cells expressing mNeGr fusions to Sur7, Nce102 or the Nce102‐Sur7‐chimera. Dots represent a five‐fold serial dilution series on YPD plates containing 40 μg/ml Calcofluor White (CW), 60 nM AureobasidinA (AbA) or grown at 37°C for 48 h. Data information: (A) Boxplot (interquartile range (box), min to max spread (whiskers) and median values (line in boxes)), ANOVA with Dunnett's multiple comparison test, *n* = 14–55 cells from three experiments. (B) Symbol graph (error bars: SD), ANOVA with Dunnett's multiple comparison test, *n* > 123 MCC/eisosomes from three experiments. (C) *n* = 26–45 cells. *P*‐values: ***P < 0.01, ns: not significant. Scale bars: 1 μm (A, B), 200 nm (C). Source data are available online for this figure.

**Figure EV5:**
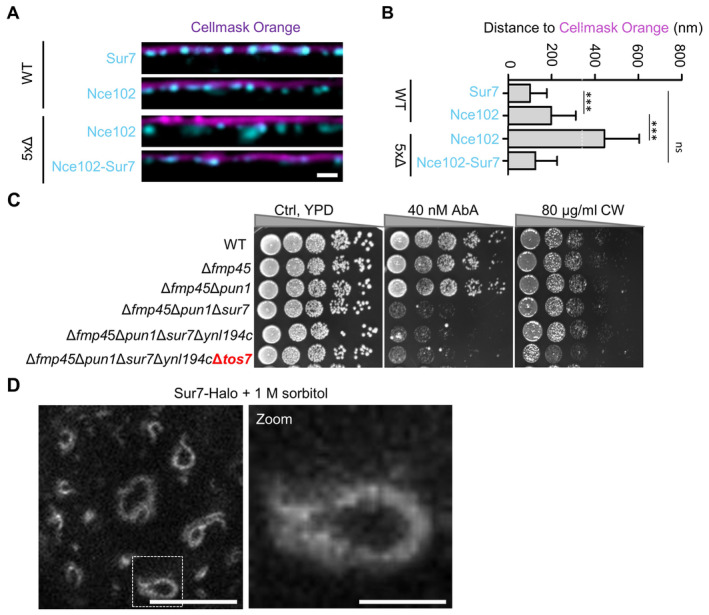
Mechanistic basis of Sur7 function in subdomain organization—Supplement Linearized profiles of WT cells expressing indicated GFP fusions (cyan) stained with the PM dye Cellmask Orange (magenta).Bar graph representing quantification of the radial distances between indicated protein and Cellmask Orange in (A).Growth assays with successive deletions of Sur7 tetraspanners. Cells were grown on YPD plates containing 40 nM AbA or 40 μg/ml calcofluor white (CW) at 30°C for 48 h.STED image of WT cells expressing Sur7‐Halo from the *PMA1* promoter after addition of 1 M sorbitol for 5 min. Data information: (B) Bar graph (error bars: SD), ANOVA with Tukey's multiple comparison test, *n* > 133 measurements from three experiments. *P*‐values: ***P < 0.01, ns: not significant. Scale bars: 1 μm, 200 nm (zoom). Source data are available online for this figure.

We found that increased PIP2 levels lead to lateral displacement of Sur7 away from MCC/eisosomes but also saw altered PIP2 distribution in Sur7‐depleted cells. To establish whether Sur7 or PIP2 unbalance was the driving force behind the conversion of PM furrows into half‐toroidal tubes we therefore wanted to test whether synthetic tethering of Sur7 to MCC/eisosomes could overcome the phenotypic changes induced by PIP2 overproduction. To this end, we fused Sur7 directly to Nce102, the only membrane‐resident component that we could identify inside the MCC/eisosome furrow. The Nce102‐Sur7 chimera was correctly localized to MCC/eisosomes (Fig 6A) and retained Sur7 functionality as it rescued the tubulation phenotype of 5xΔ cells (Fig EV5A and B). The lateral segregation of endogenous Sur7 from MCC/eisosomes in Δ*inp51*/*inp52* cells was also completely restored by expression of the chimera (Fig 6A), consistent with a retained capacity for self‐interaction and oligomerization. Remarkably, the tubulation phenotype of Δ*inp51*/*inp52* cells was also rescued by expression of the chimera as seen in radial profiles (Figs 6B and EV5A and B) and freeze–fracture EM images (Fig 6C). While the occurrence of circular structures that likely represent the ends of half‐toroidal tubes was strongly reduced, PM furrows in rescued cells often exhibited a wavy/curved appearance, indicating that the chimera was not able to fully restore normal PM topography.

To explore the physiological relevance for yeast cells of either having furrows or tubular invaginations, we monitored growth under a variety of stress conditions. We found a strong sensitivity of Inp51/52‐deficient strains to cell wall stress induced by calcofluor white (CW), to a block in synthesis of complex sphingolipids using Aureobasidin A (AbA) or to increased temperatures (Fig 6D). Remarkably, all hypersensitivities could be fully rescued by expression of the Nce102‐Sur7 chimera (Fig 6D). A link between MCC/eisosome topography and stress resistance of yeast cells was further underscored by the hypersensitivity of cells with various members of the Sur7 family deleted to AbA and CW (Fig EV5C). Interestingly, while the sensitivity to altered sphingolipids was seen in all Sur7 mutants forming PM tubes instead of furrows (at least 3xΔ), the CW sensitivity was specifically linked to an absence of Tos7, indicating further functional specialization of Sur7 members (Fig EV5C).

Taken together, we found that synthetic anchoring of Sur7 to MCC/eisosomes can restore normal PM topography and stress resistance in cells with increased PIP2 levels. This suggests a central function for Sur7 in regulating MCC/eisosome topography and function—and that a major consequence of altered PIP2 homeostasis is the displacement of Sur7.

### Acute perturbation of PM organization

**Figure 7:**
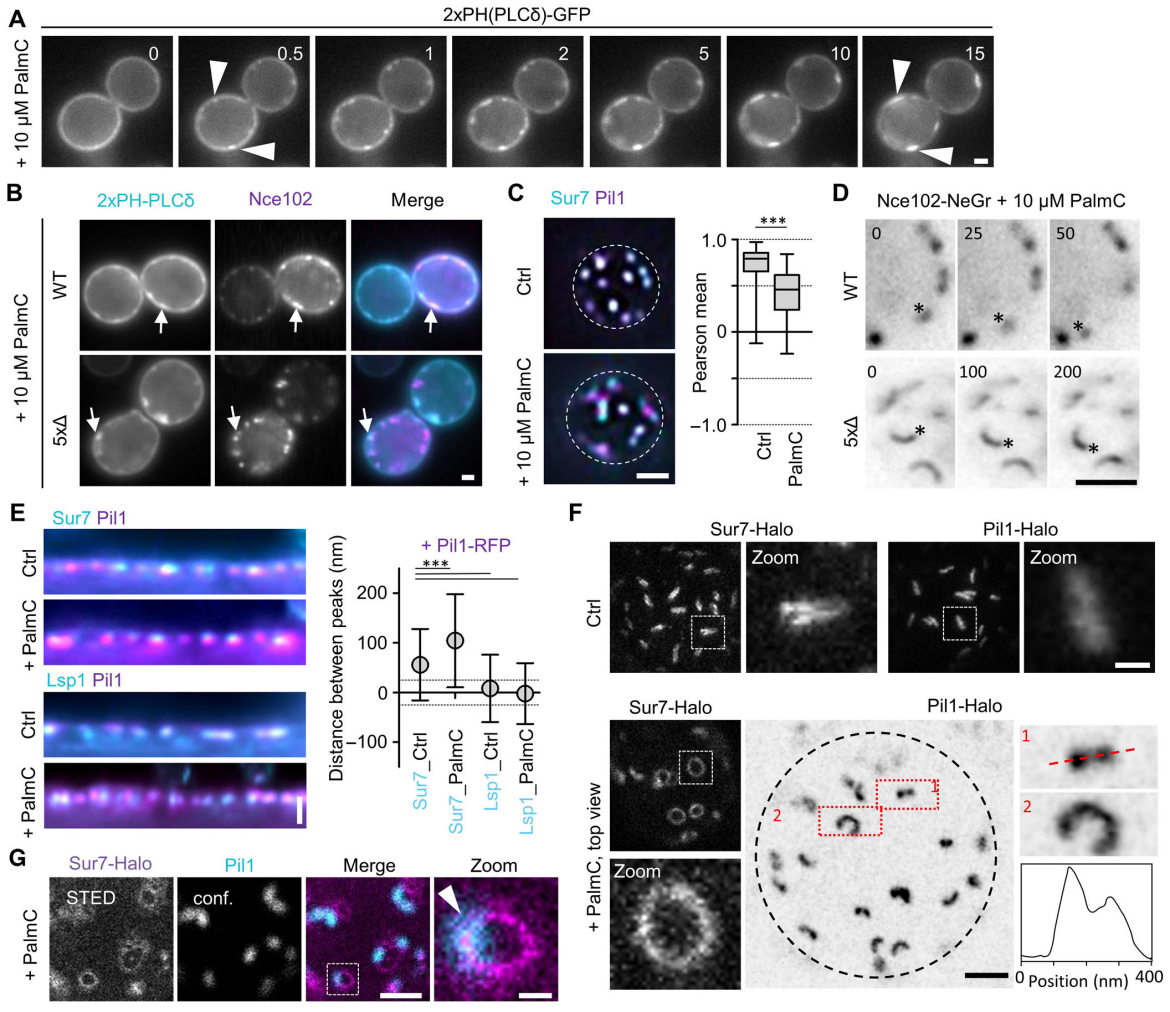
Effects of acute perturbation of MCC/eisosome topography Effect of addition of 10 μM palmitoylcarnitine (PalmC) on PM distribution of PIP2, marked by 2xPH(PLCδ)‐GFP. Time in min after PalmC treatment. Arrowheads indicate PIP2 clusters.Colocalization of Nce102‐RFP (magenta) with PIP2 marked by 2xPH(PLCδ)‐GFP (cyan) in WT and 5xΔ cells, 10 min after addition of 10 μM PalmC. Arrows indicate colocalization in clusters.Colocalization of Sur7‐mNeGr (cyan) and Pil1‐RFP (magenta) in WT cells before and after treatment with PalmC. Box plots indicate Pearson correlation coefficients in respective conditions.Exemplary time series of WT or 5xΔ cells expressing Nce102‐mNeGr after treatment with 10 μM PalmC. Asterisks indicate laterally translating structures. Time in seconds. See Movie EV1 for more examples.Linearized profiles and distances of radial profile peaks between Pil1‐RFP (magenta) and Sur7‐mNeGr (cyan) before and after PalmC treatment. The distance between Lsp1‐mNeGr and Pil1‐RFP was used as a control. Dotted lines indicate the 25 nm bracket representing the estimated resolution of peak fitting.STED images of WT cells expressing Sur7‐Halo from the *PMA1* promoter or endogenous Pil1‐Halo before and after PalmC treatment. Zoom shows curved structures observed under PalmC treatment. Black dotted line indicates the cell edge. Intensity profile was taken along the red dotted line along structure (1).Colocalization of WT cell expressing Sur7‐Halo from the *PMA1* promoter (magenta, STED) and Pil1‐mNeGr (cyan, confocal) after 10 μM PalmC treatment. Arrow head indicates the area of overlap. Data information: (C) Box plot (interquartile range (box), min to max spread (whiskers) and median values (line in boxes)), unpaired *t*‐test, *n* = 37–53 cells from three experiments. (E) Symbol graph (error bars: SD), ANOVA with Dunnett's multiple comparison test, *n* > 128 MCC/eisosomes from three experiments. *P*‐values: ***P < 0.01. Scale bars: 1 μm, 200 nm (zoom). Source data are available online for this figure.

So far, all mutants and treatments that we used led to permanent or long‐term changes in PM lipid and protein composition. The observed phenotypes could therefore be due to secondary adaptations of yeast cells. We wanted to also test our interpretation of the Sur7 function by using acute changes in MCC/eisosome composition. A previous study reported that PM tension of yeast cells could be rapidly altered by treatment with the fatty acid derivative palmitoylcarnitine (PalmC; Riggi *et al*, 2018). As shown in this report we observed the formation of PIP2 clusters within a few minutes of exposure to 10 μM PalmC (Fig 7A). Interestingly, we found that these clusters often were closely associated with MCC/eisosomes (Fig 7B) and reasoned that Sur7 localization might be directly affected by this. Indeed, we found that after only 10 min PalmC treatment, Sur7 became laterally displaced from Pil1‐marked MCC/eisosomes (Fig 7C). We next followed the effects of PalmC on Nce102‐marked MCC/eisosome furrows in WT cells or tubes in 5xΔ cells by life‐cell microscopy. To our great surprise, we found that within seconds of PalmC addition individual MCC/eisosomes or tubes started to move along the cell periphery, sometimes across the whole cell (Fig 7D, Movie EV1). The tubular nature of PM invaginations becomes very apparent in the shown sequences of 5xΔ cells. These results indicated that PalmC treatment leads to large‐scale reorganization of the yeast PM and that as a consequence Sur7 is displaced from furrow edges. We predicted that such an acute displacement should then allow the BAR domains within MCC/eisosomes to further bend the membrane and potentially form closed tubes. Consistent with this, we found that the radial distance between Pil1 and Sur7 was significantly increased, while Lsp1 and Pil1 remained closely associated (Fig 7E), indicative of deeper furrows or tubulation. We next used STED microscopy to examine the PalmC effects at a higher resolution. We found that after 10 min of treatment, Sur7 strands exhibited strong curvature, leading to circular or ovoid patterns (Fig 7F). In addition, Pil1 labeled structures often exhibited increased curvature and the characteristic double‐peak profiles seen for toroidal tubes in 5xΔ and Δ*inp51*/*inp52* cells (Fig 7F). Two color imaging of Sur7 (STED) and Pil1 (confocal) confirmed the lateral segregation of the two markers (Fig 7G). Finally, displacement of Sur7 was also observed when PM tension was reduced by exposure of cells to hyperosmotic conditions with 1 M sorbitol (10 min; Fig EV5D), indicating that the observed effects were not specific to PalmC‐treatment.

In summary, we could show that an acute reduction in PM tension and concomitantly altered PIP2 distribution leads to displacement of Sur7 from MCC/eisosomes and to a simultaneous increase of MCC/eisosome furrow depth and possibly tubulation.

## Discussion

In this study, we have uncovered a novel function for the Sur7 family of tetraspanners in modulating the topography of the yeast PM. This role is critical in protecting yeast cells from a variety of environmental stress conditions.

**Figure 8:**
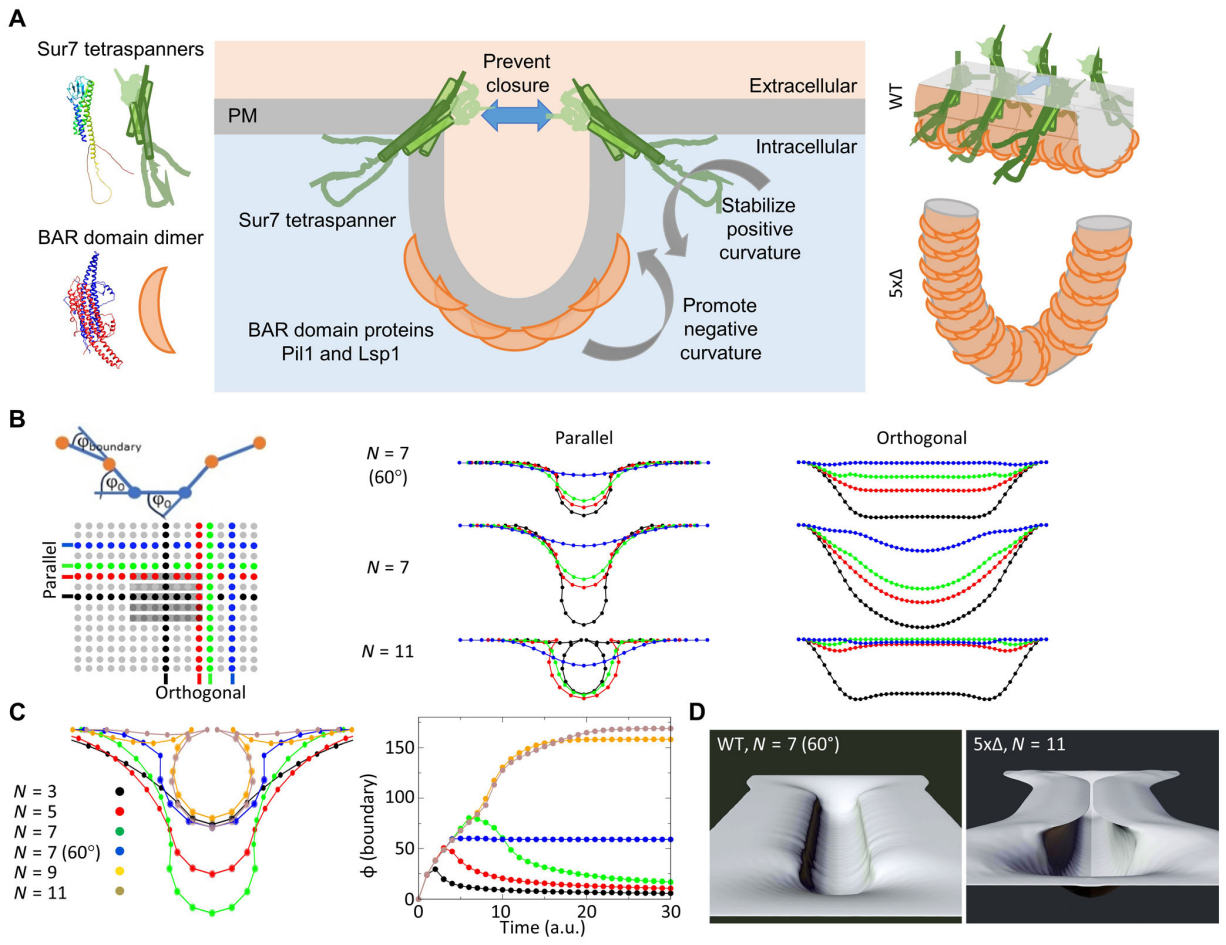
Conceptual model for tetraspanner functions in MCC/eisosome organization Mechanistic model depicting the balance of curvature generation between different MCC/eisosome subdomains. Negative curvature is induced by the binding of Pil1 and Lsp1 to PIP2‐enriched PM domains. Positive curvature at the upper edge of the formed furrows is stabilized by Sur7 parallel strands. Gray arrows indicate direction of curvature. Blue arrow illustrates the inhibition of furrow closure. Schematic protein structures are based on AlphaFold predictions and are not to scale. Right: 3D illustration of Sur7 and BAR domain distribution within MCC/eisosome furrows of WT cells and half‐toroidal tubes of 5xΔ cells.Schematic representation of the boundary angle and the central part of the simulated 25 × 49 array with indicated BAR domains (gray bars, 29 in actual simulation) and positions of line profiles parallel and orthogonal to the BAR domains (color‐coded). Profiles show bending of the membrane for different number of BAR domains (*N*) and in the presence of tetraspanners that stabilize the positive curvature at the boundary (*φ* here fixed at 60°).Cross sections parallel through the curved membrane area (equivalent to black parallel profile) with different numbers of BAR domains (*N*) and in the presence of tetraspanners that stabilize the positive curvature at the boundary *N* = 7 (60°). Note the development of omega‐shaped profiles for *N* ≥ 9 that mimic conformations where opposing parts of the membranes could fuse. In addition, the time evolution of boundary angles (see B) for different *N* and with/without stabilization of curvature at 60° is shown (right, color code is indicated).Rendered 3D views of the 2D array at equilibrium for simulations *N* = 7 (60°) that corresponds to WT MCC/eisosome furrows (left) and *N* = 11 corresponding to the 5xΔ strain (right). Also see corresponding tracking shots in Movies EV2 and EV3.

We found that the maintenance of stable PM furrows requires a balance between two force‐generating complexes: BAR domain proteins at the base of MCC/eisosome furrows establish negative curvature. Unchecked, this invagination would lead to the formation of half‐toroidal membrane tubes. To prevent this, Sur7 family tetraspanners assemble into multimeric strands at the upper longitudinal edges of membrane furrows. In effect, they stabilize the local positive curvature and counter further invagination (Fig 8A).

Using quantitative epifluorescence microscopy and STED nanoscopy, we were able to pinpoint the different components of MCC/eisosomes at the base and edge of furrows with great precision. The basal furrow region was defined by the presence of Nce102 tetraspanners and the cytosolic BAR domain proteins Pil1 and Lsp1 (Fig 8A). In contrast, we found that Sur7 proteins form parallel strands along the longitudinal axis at the upper edges of MCC/eisosome furrows (Fig 8A). We observed physical interactions between various members of the Sur7‐family, suggesting that the strands correspond to hetero‐oligomeric protein complexes. Neither the N‐ nor C‐terminal cytosolic extensions of Sur7 were necessary for these interactions, indicating that strands were formed via transmembrane regions or extracellular loops in Sur7. This is reminiscent of oligomeric structures formed by other tetraspanner families such as the tetraspanins, claudins and occludins found in mammalian cells (Lal‐Nag & Morin, 2009; Charrin *et al*, 2014). The methionine permease Mup1 and the sterol dye filipin were closely associated with the Sur7 strands at the upper edge of MCC/eisosome furrows suggesting specific lipid composition and biological functions of this region. Importantly, our observation of distinct subdomains within MCC/eisosomes is consistent with previous measurements using single molecule localization (Appadurai *et al*, 2020) and immunogold labeling (Strádalová *et al*, 2009).

A key finding of our study is that the establishment and maintenance of MCC/eisosomes depend on the balance of two forces with one acting at the base and the other on the edges of furrows. This is based on our observation that in the absence of Sur7 tetraspanners the characteristic halfpipe‐shaped MCC/eisosome furrows are replaced by half‐toroidal tubes that are labeled by Nce102 and Pil1 and remain attached to the PM at both ends (Fig 8A).

This highly unusual topography requires local membrane fission. At first glance, it is difficult to rationalize how BAR domain‐mediated bending can lead to such drastic changes in PM conformation. We see several unique components and biophysical factors of the yeast system that might contribute to this phenomenon:

- Tube formation is driven by pre‐assembled BAR domain proteins. We could show that tube formation depends on the presence of Pil1 and the end diameter of tubes in freeze–fracture EM was consistent with membrane tubes formed by purified Pil1 (Karotki *et al*, 2011) or Lsp1 (Zhao *et al*, 2013). We also found that the distance of tube ends was determined by the level of the scaffold protein Seg1, which recruits Pil1 and Lsp1 into linear structures at the PM and provides a polarized orientation for their assembly and forces (Moreira *et al*, 2012). The large PM area (over 200 nm in length) covered by the Seg1‐BAR domain scaffold might increase the chance for spontaneous membrane fission once the membranes are closely apposed, which could then rapidly expand to form the observed half‐toroidal tubes.
- The yeast PM has gel‐like properties (Aresta‐Branco *et al*, 2011), likely allowing it to withstand the considerable lateral forces that are expected to arise from the cooperative forces exerted by BAR domain coats and tetraspanner strands.
- The high turgor pressure of yeast cells is expected to further compress invaginations with boundary angles (upper edge of furrows) beyond 90°.
- The strong preference of Sur7 for positively curved membranes is expected to facilitate recruitment and possibly strand assembly at the edge of developing furrows.

To further explore our hypothesis we compared the membrane deformation by BAR domains with and without stabilizing tetraspanners using Monte Carlo simulations of 2D particle arrays with interaction terms that represented a gel‐like membrane and enabled in‐plane and out‐of‐plane spontaneous fluctuations (see Materials and Methods).

At first, we implemented BAR domains (gray bars in Fig 8B) as a bias of these fluctuations within the membrane towards a negative angle of −28° (*φ*_0_ in Fig 8B) and fixed the number of biased rows and orientation of the bias (parallel) to approximate the effect of the Seg1 scaffold. We then ran simulations with varying numbers (*N*) of sequential BAR domain elements (number of successive *φ*_0_ around center), effectively controlling the degree of membrane deformation.

Radial profiles parallel or orthogonal to the BAR domain‐induced curvature (Fig 8B) demonstrate that for low number of BAR domains (*N* ≤ 7) invaginations follow a non‐monotonous growth process and reach far into the cell (Fig 8C). Outside the region covered by BAR domains the invaginations gradually approach the flat membrane resulting in small positive boundary angles < 20° (Fig 8B and C). Such broad invaginations could easily attract additional BAR domains to the edge of the curved region in a positive feedback. Note that due to the selected *φ*_0_ of −28° the invagination in the BAR domain‐covered area is basically a half circle for *N* = 7. If *N* is further increased, the qualitative features of invaginations change. They adopt an omega‐shape close to the surface, with boundary angles >90° (Fig 8C and D). The complex spatial behavior of the membrane at the edge of the BAR domain reflects the interplay between the pre‐patterned BAR domains and the overall tendency of the membrane to remain flat. Such omega‐structures with high boundary angles have a significant energy penalty, which may be resolved by localized membrane fission and the formation of half‐toroidal tubes.

We next implemented tetraspanners in our simulations by fixing the boundary angle at 60°, once this curvature was reached. This angle is reached for *N* ≥ 7 and reflects the predicted preference of Sur7 for high positive curvature (Fig 2E). The restriction of boundary angles led to the formation of stable furrows instead of omega structures (Fig 8C and D), since it stops further recruitment of BAR domains (increase of *N*). Examples of structures formed by simulations with only BAR domains or with additionally implemented tetraspanners (60° limit for boundary angle) can be seen in Fig 8D and Movies EV2/EV3. Note the areas with lower curvature at the tip areas not covered by BAR domains, which adopt a conformation resembling the circular ends of tubes in 5xΔ cells.

In summary, our simulations illustrate a possible mechanism for the formation of half‐toroidal membrane tubes, as well as a possible suppression of this mechanism by the presence of tetraspanners.

It is important to note that in both the 5xΔ (Fig EV4) and Δ*inp51/52* mutants (Singer‐Krüger *et al*, 1998; Stefan *et al*, 2002) additional membrane invaginations occur that often are more extensive (vacuole‐ or sheet‐like, extending of several 100 nm in serial EM sections), are likely filled with cell wall material and presumably do not contain Pil1 or Nce102 (Fig EV4D). We currently do not understand the molecular nature of these larger invaginations but think that they are not directly related to Pil1 function or Sur7 tetraspanners. Future studies will further explore this issue.

As to the exact mechanism of Sur7 function, we found that the regulation of membrane topography depended on the tetraspanners cytosolic C‐terminal extension. Removal of the tail did not affect localization at WT MCC/eisosome edges and did not block self‐association in Co‐IP experiments. Nonetheless the C‐terminal truncation of Sur7 failed to prevent the formation of membrane tubes.

The C‐terminal tails of claudins are known to be important sites of interaction with partner proteins and thereby promote tight junction formation in mammalian cells (Lal‐Nag & Morin, 2009). Similarly, the C‐terminal region of Sur7 has been shown to play a key functional role in *C. albicans* morphogenesis under various stress conditions (Lanze *et al*, 2021). Sur7 has been proposed to interact with the yeast cell wall through its extracellular domains (Young *et al*, 2002). While the orientation of the C‐terminus towards the cytosol makes a direct interaction with the cell wall unlikely, indirect effects through conformational changes are definitely possible. Further experiments will be required to identify the relevant interaction partners of the C‐terminal segment that mediate its function in regulating membrane topography.

MCC/eisosomes have been previously linked to lipid homeostasis, including regulation of PIP2 levels through Pil1‐associated PIP2 phosphatase Inp51 (Fröhlich *et al*, 2014) and the control of sphingolipid metabolism via the MCC/eisosome‐associated sensors Slm1/2 (Berchtold *et al*, 2012) and Nce102 (Fröhlich *et al*, 2009; Zahumenský *et al*, 2022). Interestingly, previous studies have indicated that an increase in PIP2 levels leads to the formation of tubular invaginations that strongly resemble those found in 5xΔ cells (Stefan *et al*, 2002; Karotki *et al*, 2011). We have now shown that these effects on MCC/eisosome topography are accompanied by a displacement of Sur7. This altered distribution occurs in the Δ*inp51*/*52* mutant, in which PIP2 levels are increased. In addition, acute treatment with PalmC was associated with both, formation of PIP2 clusters in the PM (Fig 7A, Riggi *et al*, 2018) and relocation of Sur7. Strikingly, the PM topography defects of the Δ*inp51*/*52* mutant could be rescued by synthetic tethering of Sur7 to MCC/eisosomes.

While PIP2 levels very likely regulate Sur7 localization and function, we also found evidence for the inverse relation: The Inp51 phosphatase has been shown to directly interact with Pil1 (Fröhlich *et al*, 2014) and was localized at the base of MCC/eisosome furrows in WT cells (Fig 5G). However, in 5xΔ cells Inp51 was found at the PM outside of half‐toroidal tubes (Fig 5G). This displaced Inp51 would be expected to have two distinct consequences that are consistent with our experimental findings: (i) An increase of PIP2 levels within tubes that is expected to recruit additional BAR domains that mediate tubulation and (ii) phosphatase outside tubes that is no longer restricted by binding to Pil1 and therefore lowers the overall level of PIP2 in the PM (Fig 5F). Taken together, our results suggest that Sur7 strands on the one hand restrict localization and function of Inp51 to MCC/eisosomes and on the other hand are themselves sensitive to local PIP2 levels.

Importantly, the rescue of the Δ*inp51*/*52* mutant by the Nce102‐Sur7 chimera did not only restore topographic changes in the PM, but also abolished the increased sensitivity to various cellular stresses, including cell wall disruption, inhibition of sphingolipid synthesis and increased temperature. This indicates that correct PM topography is critical for many physiological functions of the PM and explains the intricate mechanisms that have evolved to maintain a tight balance between opposing forces and curvatures.

## Materials and Methods

### Yeast strains and plasmids

All strains in this study were derived from the *S. cerevisiae* BY4741 (Euroscarf). Genomic tagging and deletions were performed by direct integration of PCR products as described previously (Janke *et al*, 2004). The five‐fold tetraspanner mutant was created by a variant of the *delitto perfetto* marker‐less approach using the counter‐selectable *Kl*URA3 marker (Stuckey & Storici, 2013). Counter‐selection was performed on SCD plates containing 1 mg/ml 5‐fluoroorotic acid. After loss of the *Kl*URA3 marker, the following remnant sequence was left at each integration site: CGT ACG CTG CAG GTC GAC AAC CCT TAA TAT AAC TTT ATA ATG TAT GTA TAG AAG TTA TTA GGT GAT ATC AGA TCC ACT AGT GGC CTA TGC. PCR products for direct integration were generated through overlapping PCR using standard methods. All plasmids were constructed using standard molecular biology techniques. Transformation into yeast cells was performed using the LiOAc method (Janke *et al*, 2004). All plasmid sequences were verified. PCR‐derived endogenous integrations were verified via colony‐PCR and additional sequencing for point mutations. All strains, plasmids, oligonucleotides and linker sequences used in this study are listed in Dataset EV1.

### Media and growth conditions

If not otherwise indicated, all yeast strains were grown overnight in standard Yeast extract Peptone Dextrose medium (YPD) or in synthetic complete media with 2% glucose (SCD) at 30°C. Before imaging, cells were washed (1 min at 1,000 × *g*) in H_2_O and diluted 1:20 in appropriate SCD medium, and grown to logarithmic phase for a further 2–4 h. The Δ*cho1*‐mutant was grown in medium supplemented with 1 mM ethanolamine, and the Δ*psd1/2*‐mutant was supplemented with 1 mM choline. For perturbation of complex SL‐synthesis, the SCD medium was supplemented with 5 μM AureobasidinA (Clontech), diluted from a 5 mM stock solution (in ethanol) in SCD medium and cells were incubated for 1 h at 30°C. For acute modulation of the lipid composition of the PM, palmitoyl‐DL‐carnitine chloride (PalmC, Sigma‐Aldrich) was added from a 10 mM stock (in DMSO) to a final concentration of 10 μM, and cells were incubated for the indicated time at 30°C. Hyperosmotic shock was applied by addition of 1 M sorbitol for 5–10 min before imaging.

### Plate growth assays

Indicated yeast strains were grown overnight in YPD at 30°C. Cells were washed (1 min at 1,000 × *g*) once in H_2_O and diluted 1:10 into fresh medium for further incubation over 2–3 h at 30°C. Logarithmically growing cells were spotted in a five‐fold serial dilution, starting at OD_600_ of 0.05, on agar plates containing indicated concentration of AureobasidinA (Clontech), Calcofluor White (Sigma‐Aldrich), or phytosphingosine (ChemCruz). Plates were incubated at 30°C and imaged after 48 h.

### Filipin staining

One milliliter aliquots of logarithmic grown yeast cells were washed (1 min at 1,000 × *g*) once in PBS and resuspended in 1 ml PBS containing 5 μg/ml filipin (Sigma‐Aldrich, Stock: 5 mg/ml in DMSO). Cells were incubated for 5 min at room temperature in the dark, washed once with PBS and imaged with 355 nm excitation.

### FM4‐64 staining

One milliliter aliquots of logarithmic grown yeast cells were washed (1 min at 1,000 × *g*) once in PBS and resuspended in 1 ml PBS containing 1 μg/ml FM™ 4–64 (stock: 1 mg/ml in DMSO, stored at −20°C). Samples were incubated for 1 min at room temperature and subsequently washed once with PBS. Cells were imaged at 561 nm excitation.

### CellMask Orange staining

One milliliter aliquots of logarithmically grown yeast cells were washed (1 min at 1,000 × *g*) in PBS and resuspended in 1 ml PBS with 0.5 μg/ml CellMask™ Orange (stock 5 mg/ml in DMSO, ThermoFisher Scientific). Samples were incubated for 5 min at room temperature and subsequently washed three times in PBS. Cells were imaged at 561 nm excitation.

### Co‐Immunoprecipitation (Co‐IP)

Co‐IP experiments were performed as described previously (Bonifacino *et al*, 2001). In brief, 30 μl Protein G Sepharose® (#GE17‐0618‐01, Merck) was coupled to 1 μg monoclonal mouse anti‐GFP antibody (#11814460001, Roche) and incubated with protein extracts. Crude extracts were obtained by crushing 50 OD_600_ cells with glass beads in non‐denaturing lysis buffer (50 mM Tris/HCl pH 7.4, 300 mM NaCl, 10% Glycerin, 1% Triton X‐100, 100 mM PMSF, 2 μg/ml leupeptin, 1× complete EDTA‐free inhibitor cocktail). After centrifugation at 16,000 × *g* for 15 min at 4°C, the supernatant was pre‐cleared with uncoupled Protein G Sepharose for 1 h at 4°C. An aliquot of the supernatant was diluted 1:1 with HU‐Buffer + 1.5% DTT (0.2 M Tris/HCl pH 6.8, 8 M Urea, 5% SDS, 1 mM EDTA, 0.1% bromophenol blue) and used as the input‐sample (I‐sample). The pre‐cleared supernatant was split into two parts that were either incubated overnight at 4°C with Protein G Sepharose conjugated to unspecific‐IgG (Control, C‐sample) or to anti‐GFP antibody (Co‐IP, IP‐Sample). The antibody‐conjugated beads were washed three times with washing buffer (0.1% (w/v) Triton X‐100, 50 mM Tris/HCl pH 7.4, 300 mM NaCl, 5 mM EDTA) on ice, diluted 1:1 with HU‐buffer + 1.5% DTT, heated at 65°C for 30 min and analyzed via SDS–PAGE and Western blot. Membranes were probed with primary polyclonal rabbit anti‐HA and anti‐GFP antibodies (#51064‐2‐AP, #50430‐2‐AP, Proteintech) used in Figs EV1F and 1 or mouse anti‐GFP antibody (#11814460001, Roche). Signals were detected by chemiluminescence following incubation with AffiniPure Goat Anti‐Rabbit or AffiniPure Goat Anti‐Mouse IgG conjugated to horseradish peroxidase, respectively (#111‐035‐003, #115‐035‐003, Jackson Immuno Research).

### Protein extraction

One milliliter of yeast cells (OD_600_ = 1) from logarithmic growth phase were harvested by centrifugation (1 min at 1,000 × *g*). Cell pellets were suspended in 100 μl ice‐cold H_2_O. Cell lysis and protein precipitation were performed by sequential addition of 50 μl of 2 M NaOH solution and 50 μl of a 50% TCA solution for 10 min on ice each. Samples were centrifuged for 5 min at 18,000 × *g* (4°C) and the pellet was resuspended in 100 μl HU‐Buffer + 1.5% DTT. Samples were further processed by SDS‐PAGE and immunoblotting. Samples were probed with monoclonal mouse anti‐mNeonGreen (mNeGr) IgGs (#32F6, Chromotek) and monoclonal mouse anti‐GAPDH IgGs (#ab125247, Abcam). Primary antibodies were detected and quantified by Western blotting, as described in the previous section.

### Fluorescence microscopy

Coverslips were cleaned by sequential sonication in absolute Ethanol, Acetone, 1 M NaOH and H_2_O for 30 min each. Before imaging, coverslips were coated with 12 μl of 1 mg/ml Concanavalin A (Sigma‐Aldrich) and air‐dried. Epifluorescence (medial view) and Total Internal Reflection Fluorescence Microscopy (TIRFM, top view) was performed on iMIC‐based microscopes (FEI/Till Photonics) equipped with Olympus 100×/1.45 NA oil immersion objectives and DPSS lasers at 488 nm (Cobolt Calypso, 75 mW) and 561 nm (Cobolt Jive, 150 mW). For filipin imaging a polychrome at 355 nm excitation was used. A two‐axis galvanometer‐driven scanning head was used to adjust TIRFM‐angles individually for each color. Two separate dichroic filter cubes were used for detection of GFP and RFP signals. For filipin imaging a dichromatic quadband dichroic filter cube (zt405/488/561/640 RPC) was used. Images were acquired on an Andor iXON DU‐897 EMCCD or IMAGO‐QE camera controlled by the LiveAcquisition software (FEI/Till Photonics).

### STED super‐resolution microscopy

For STED imaging proteins of interest were genetically fused to the HaloTag (Halo) and subsequently labeled with Janelia Fluor® 646 HaloTag ligand (#GA1121, Promega) by incubating 200 nM ligand (from 200 μM stock in DMSO) with cells in SCD medium at 30°C for 30–120 min. STED super‐resolution imaging was performed on a STEDYCON scanner with pulsed 450 nm (confocal), 640 nm excitation lasers and a pulsed 775 nm depletion laser (Abberior Instruments GmbH, Göttingen, Germany). The STEDYCON was attached to a Nikon Eclipse Ti‐E microscope with a 100×/1.45 NA oil immersion objective. Depletion laser power was set to obtain a pixel size of 25 nm, the pinhole was fixed at 1.1 Airy units and the pixel dwell time was set to 10 μs with five line accumulations. The STED signal was collected by an avalanche photo diode after passing a 675/25 nm bandpass filter with a gating of 1–7 ns. The STEDYCON was controlled via the STEDYCON Smart Control software. Samples in Fig EV2B and the Δ*inp51/52* cells in Fig 7A were analyzed on a Leica TCS SP8 STED3x microscope equipped with a 100×/1.40 oil immersion objective and a pulsed (80 MHz) white light excitation laser. Excitation was performed using 488/561/633 nm laser lines and for depletion a pulsed laser at 775 nm was used. The depletion laser power was set to achieve a pixel size of 25 nm with eight line accumulations, the pinhole was fixed at 1.0 Airy units and the pixel dwell time was set to 8.6 μs. The STED signal was collected at 648–701 nm using a hybrid detector with a gating time of 0.6–6 ns. The microscope was controlled by LAS X software.

### Freeze–fracture transmission electron microscopy

Five milliliter of yeast cells from logarithmic growth phase (4 h) were harvested by centrifugation (1 min at 1,000 × *g*) and washed in KPi‐buffer (50 mM potassium phosphate buffer, pH 5.5). Cells were fixed for 30 min in 1% (final v/V) glutaraldehyde and subsequently washed three times in KPi‐buffer. Fixed samples were stored overnight in KPi‐buffer with 20% BSA at 4°C. A 2 μl aliquot was loaded into a gold‐coated copper carrier (3 mm ø) with a dimple, frozen in liquid ethane (−170°C) and transferred into liquid nitrogen. The sample was channeled into a Leica ACE900 and adjusted at −130°C. The sample was cut with the fracturing knife at −110°C and immediately coated with 2.5 nm Pt/C (45°, without rotation). The replica was stabilized with 30 nm C‐coating (90°, 120 rpm). The replica was cleaned three times in _dd_H_2_O, in 48% H_2_SO_4_ for ~16 h (overnight), in 75% H_2_SO_4_ for 3 h and in _dd_H_2_O (5x). The sample was loaded onto a pioloform‐coated copper grid and images were acquired on a Phillips CM 10 transmission electron microscope and a TEMCam F‐416 camera from TVIPS (Gauting, Germany).

### Ultrathin section transmission electron microscopy

Five milliliter of yeast cells from logarithmic growth phase (4 h) were harvested by centrifugation (1 min at 1,000 × *g*) and washed in phosphate buffer (PBS, pH 7.3). Cells were fixed in 2.5% (v/V) glutaraldehyde in phosphate buffer for ~16 h at 4°C. After washing three times with Sörensen phosphate buffer (pH 7.3), yeast cells were post‐fixed in Sörensen phosphate buffer (pH 7.3) containing 1% osmium tetroxide (OsO4) for 1 h at room temperature.

The samples were dehydrated at room temperature by passage through a graded ethanol series (30, 50, 70% for 10 min each and 90% and 2× 100% absolute ethanol for 15 min each), and further dehydrated in propyleneoxide for 20 min. Subsequently, the samples were infiltrated in a 2:1 (v/V) mixture of propyleneoxide: epon (overnight, 4°C), 1:1 mixture (3 h, room temperature), and 1:2 (overnigth, 4°C). Finally, the samples were infiltrated with pure epon for 3 h and then again overnight. Infiltrated samples were polymerized in epon‐filled molds at 60°C for 36 h. Ultrathin sections (~60 nm) were cut on an ultramicrotome (Reichert Ultracut S) with a diamond knife. Sections were placed on pioloform‐coated TEM copper grids and contrasted with uranyl acetate (20 min) and lead‐citrate (70–90 s.).

For serial sectioning, yeast cells were washed with double‐distilled water (DDW) after glutaraldehyde fixation and post‐fixed in 1% aqueous KMnO_4_ for 1 h at room temperature (Baharaeen & Vishniac, 1982) followed by a thorough wash with DDW until the sample was colorless. Then the samples were additionally stained en‐bloc with 4% aqueous neodymium acetate for 1 h at room temperature (Kuipers & Giepmans, 2020). Dehydration and embedding in resin were performed as described above. Ultrathin serial sections (~60 nm) were cut on an ultramicrotome (Leica UC7) with a diamond knife. Sections were placed on formvar‐coated TEM aperture copper grids; no post‐contrasting was used. All Images were acquired on a Phillips CM 10 transmission electron microscope with a TEMCam F‐416 camera from TVIPS (Gauting, Germany).

### Image processing and visualization

TIRFM images were processed using Fiji and MATLAB. Raw TIRFM images were deconvolved (deconvlucy) using the Lucy‐Richardson algorithm with 20 iterations and a PSF function obtained from 100 nm tetraspec microspheres in MATLAB. Protein colocalization (Pearson mean) and fluorescence intensity distribution (Network factor) were calculated from two‐color TIRFM images using a customized script in MATLAB. In brief, cells were automatically detected, deconvolved and thresholded. For colocalization between GFP and RFP signals, a mask was generated for each channel and combined by AND‐function. The colocalization between both channels was calculated using the Pearson correlation coefficient.

The normalized intensity distribution (i.e., the network factor) (Spira *et al*, 2012; Busto *et al*, 2018) was calculated from deconvolved TIRFM images. A rolling ball filter with 25 pixel diameter was used for background equalization. Number of iterations for deconvolution was set to 20. Low values (≤ 0.15) indicate clustered and patchy structures, whereas higher values (> 0.15) represent a more disperse and network‐like distribution.

Equatorial epifluorescence images and STED images are shown as raw images. All images were contrast‐adjusted and zoomed for presentation purposes only. Additionally, samples treated with CellMask™ Orange were denoised, using the “remove background” algorithm (radius 10 pixel) in Fiji.

Linearized profiles of single yeast cells were obtained from circular segmented ROIs along the cell periphery. The ROIs were processed with the “straightener” function in Fiji. For visualization, images were scaled with bilinear interpolation and X, Y scaling‐factor of 4. Linearized profiles were used exclusively for visualization and not for quantification of intensity profiles.

The radial distance between two signal peaks was measured using a semi‐automated Fiji macro. Two‐color images were merged and shift‐corrected. Subsequently, three linear ROIs perpendicular to individual MCC/eisosomes were drawn per mother cell at intervals of approximately 120 degrees. ROIs were further processed by using the “multichannel plot profile” function in Fiji. Distances were transferred and visualized in Prism 5.0 (GraphPad).

To calculate the PM/cytosol ratio, two circular ROIs were drawn around the cell periphery enclosing the PM. The PM intensity was obtained by subtracting inner from outer ROI using a custom Fiji macro. Background values were subtracted before calculating the ratios.

### Protein structure prediction

Protein topology was predicted by TMHMM (Krogh *et al*, 2001). Protein structures were obtained from the Alphafold structure database (Jumper *et al*, 2021; Varadi *et al*, 2022). The spatial arrangement of Nce102 (UniProt: Q12207) and Sur7 (UniProt: P54003) in planar and curved membranes (fungal PM) was predicted by PPM 3.0 (PPM 3.0 Web Sever_cgopm) (Lomize *et al*, 2022).

### Membrane simulations

To represent a simplified membrane we considered a 2D lattice of size $L_{x}\times L_{y}$. Specifically, we chose $L_{x}=25$ (corresponding to points *i* = 0, 1, …, $L_{x}-1$) and $L_{y}=49$ (corresponding to points *j* = 0, 1, …, $L_{y}-1$). To reflect the orientation and length bias of the Seg1 scaffold for BAR domain assembly we aligned a variable number of BAR domains (*N* points where *N* is chosen to be an odd number) along the *x*‐axis from $i=i_{\min}\equiv L_{x}-1-\frac{N}{2}$ to $i=i_{\max}\equiv L_{x}-1+\frac{N}{2}$ and defined a fixed number of 29 sets of BAR domains, ranging from $j=j_{\min}\equiv L_{y}-15$ to $j=j_{\max}\equiv L_{y}+13$.

In equilibrium each point corresponds to one region of the membrane with positions $x\left(i,j\right)=i$ and $y\left(i,j\right)=j$. Thus the distance of adjacent points is unity. Furthermore, each region is characterized by a variable, non‐positive height $z\left(i,j\right)$ to reflect fluctuations and the restriction of the cell wall. At the boundaries, i.e. for $i=0,1,L_{x}-2,L_{x}-1$, as well as for $j=0,1,L_{y}-2,L_{y}-1$ the height is fixed to 0, reflecting the boundary conditions when incorporating the simulated part of the membrane into a larger array. Values for $y\left(i,j\right)=j$ were kept constant, while the values $x\left(i,j\right)$ varied to allow the interaction with the BAR domains. The array was then modeled as a network of harmonic springs (in *x*‐ and *y*‐direction) between nearest neighbors to take the forces within the 2D plane of the membrane into account. Distances were constrained between *r* = 0.5 and *r* = 2 and the harmonic spring potential is defined as $V_{\text{harm}}\left(r\right)=k_{\text{harm}}\left(r\right){\left(r^{2}-1\right)}^{2}$. To model the drag force of the surrounding membrane we introduced the drag potential $V_{\text{drag}}\left(x\left(i=0\right),y(j\right))=+k_{\text{drag}}x$, $V_{\text{drag}}\left(x\left(i=L_{x}-1\right),y(j\right))=-k_{\text{drag}}x$. Specially we chose the following parameters (units are expressed in terms of $k_{B}T$): $k_{\text{harm}}\left(0.5<r\leq 1\right)=100,000$, $k_{\text{harm}}\left(1<r\leq 2\right)=20,000$, $k_{\text{drag}}=800$. These parameters ensured that without any additional interactions equilibrium distances of array points were close to unity and that fluctuations were of the order 0.01.

The fluctuations in *z*‐direction, reflecting the elastic properties of the membrane, were modeled with the standard Cahn–Hilliard approach (Cahn & Hilliard, 1958) via the elastic potential $V_{\text{elast}}\left(i,j\right)=k_{\text{elast}}\;\varphi_{x}{\left(i,j\right)}^{2}+k_{\text{elast}}\;\varphi_{y}{\left(i,j\right)}^{2}$. Here$\;\varphi_{x}\left(i,j\right)$ denotes the angle formed by the membrane elements at positions (i−1, j), (i, j) and (i + 1, j). $\;\varphi_{y}\left(i,j\right)$ is defined analogously along the *j*‐direction. Using a value of $\;k_{\text{elast}}=80,000$ the typical fluctuations in *z*‐direction without external potentials were kept close to 0.01. Finally, the BAR domains were modeled by providing an optimum angle for $\;\varphi_{x}\left(i,j\right)$ defined as $\varphi_{\text{curv}}$. Thus, the potential of membrane elements bound by a BAR domain is given as $V_{\text{elast}}\left(i,j\right)=k_{\text{curv}}{\left[\;\varphi_{x}\left(i,j\right)-\varphi_{\text{curv}}\right]}^{2}+k_{\text{elast}}\;\varphi_{y}{\left(i,j\right)}^{2}$. The anisotropy reflects the properties of the BAR domains. We chose $\;k_{\text{curv}}=1,200,000$ empirically so that in the presence of BAR domains the system can balance the forces within the 2D plane (reflected by deviations of adjacent lattice points from the optimum distance 1) as well as the forces exerted by the BAR domains (reflected by deviations from $\varphi_{\text{curv}}$, here chosen as 28°).

For time evolution of this model we used Monte Carlo simulations. We randomly chose one membrane element and displace its *x*‐ and *z*‐component with a random number between −0.001 and 0.001. The new configuration was accepted with the standard Metropolis criterion. Two additional technical points have been included. First, it turns out that the system may find local minima of the free energy which correspond to highly asymmetric configurations and possess much higher energies than the finally converged symmetric states. To avoid this artifact, we apply an additional symmetry criterion, namely $\left|\;\varphi_{x}\left(i_{\min}-1,j\right)-\varphi_{x}\left(i_{\max}+1,j\right)\right|<10{}^{\circ}$. The precise value does not matter because the final configurations turn out to be highly symmetric. Second, for *N* = 7 we perform a second simulation to take into account the additional attraction of a tetra‐spanner under appropriate conditions of the curvature close to the BAR domain. Specifically, we consider (for $j\in \left[j_{\min},j_{\max}\right]$) the angles $\varphi_{x}\left(i_{\min}-1,j\right)$ and $\varphi_{x}\left(i_{\max}+1,j\right)$. Once, these angles are larger than 60° (reflecting a positive curvature as opposed to the negative curvature below the BAR domains) the angle is fixed with a maximum deviation of 5°. In this way subsequent attraction of additional BAR domains would be avoided and the system can relax.

We used the open‐source python‐library “Open3D” to generate a surface from the simulated point‐cloud using the ball‐pivoting function. We then used the open source 3D‐graphics suite Blender to smooth and render the surface mesh. We also used Blender to generate tracking shots for structures generated with *N* = 7 (60°) and *N* = 11.

All programs and code for the model and surface rendering are available upon request.

#### Data information and statistics

Boxplots show interquartile range (box), min to max spread (whiskers) and median values (line in boxes). Symbol graphs and bar graphs depict mean ± standard deviation (SD). Number of measurements (*n*) is indicated in each figure legend. Statistical analysis was carried out with Prism 5.0 (GraphPad). For statistical comparison between two samples an unpaired *t*‐test (***P < 0.001) was used. For comparison of multiple samples with a single control, one‐way ANOVA with Dunnett's multiple comparison test (***P < 0.01) was used. For comparison of multiple columns, one‐way ANOVA with Tukey's multiple comparison test (***P < 0.01) was performed. All graphs were generated in Prism 5.0 (GraphPad).

## Author contributions

**Daniel Haase:** Conceptualization; formal analysis; validation; investigation; visualization; methodology; writing – original draft; project administration; writing – review and editing. **Christiane Rasch:** Investigation; methodology. **Ulrike Keller:** Investigation; methodology. **Yaroslav Tsytsyura:** Investigation; methodology. **Nataliya Glyvuk:** Investigation; methodology. **Annegret Elting:** Investigation; visualization; methodology. **Julia Wittmar:** Investigation; methodology. **Annette Janning:** Investigation; methodology. **Martin Kahms:** Methodology. **Noah Wedlich:** Software; visualization; methodology. **Christian Schuberth:** Conceptualization; formal analysis; supervision; investigation; methodology. **Andreas Heuer:** Software; formal analysis; validation; visualization; writing – review and editing. **Jürgen Klingauf:** Supervision; funding acquisition; methodology. **Roland Wedlich‐Söldner:** Conceptualization; data curation; formal analysis; supervision; funding acquisition; validation; investigation; visualization; methodology; writing – original draft; project administration; writing – review and editing.

## Disclosure and competing interests statement

The authors declare that they have no conflict of interest.

## Supporting information



Expanded View Figures PDF

Movie EV1

Movie EV2

Movie EV3

Dataset EV1

PDF+

Source Data for Expanded View

Source Data for Figure 1

Source Data for Figure 2

Source Data for Figure 3

Source Data for Figure 4

Source Data for Figure 5

Source Data for Figure 6

Source Data for Figure 7

## Data Availability

No data were deposited in databases.
