# Supplementary material for: Tetraspanner‐based nanodomains modulate BAR domain‐induced membrane curvature
Source: EMBO Rep. 2023 Oct 30;24(12):e57232. doi: 10.15252/embr.202357232 (PMC10702824; doi:10.15252/embr.202357232)
Supplement: Supplementary file 1 — Expanded View Figures PDF [file EMBR-24-e57232-s013.pdf]

Expanded View Figures

Figure EV1. Yeast tetraspanners define subdomains in MCC/eisosomes—Supplement.

A TIRFM images showing colocalization of indicated tetraspanners (C-terminally endogenously fused to GFP (cyan)) with the MCC/eisosome marker Pil1-RFP (magenta).
B Pearson correlation coefficients for colocalization shown in (A).
C Lateral distribution of tetraspanners from (A) quantified using the network factor.
D Linearized profiles of WT cells expressing endogenous mNeGr fusions to indicated Sur7 tetraspanners and Lsp1 (cyan) together with Pil1-RFP (magenta). Symbol graph represents quantification of radial distances between indicated protein pairs. Dotted lines indicate the 25 nm bracket representing the estimated resolution of peak fitting.
E Linearized profiles of WT cells expressing Sur7-GFP from the *PMA1* promoter (cyan) and Pil1-RFP (magenta) stained with 5 μg/ml filipin (magenta/cyan). Symbol graph represents quantification of radial distances between indicated pairs. Dotted lines indicate the 25 nm bracket representing the estimated resolution of peak fitting.
F Co-Immunoprecipitation of different Sur7 tetraspanner pairs. The indicated GFP-tagged bait proteins were overexpressed from the GPD promoter and were pulled down with anti-GFP antibody. The prey protein Sur7-3xHA was expressed under the *PMA1* promoter. Western blot probed with antibodies directed against HA and GFP. I: Input, IP: Co-IP with anti-GFP, C: Control IP with unspecific IgG.

Data information: (B, C) Boxplots (interquartile range (box), min to max spread (whiskers) and median values (line in boxes)), ANOVA with Dunnett's multiple comparison test, $n = 29–49$ cells from three experiments (B), $n = 59–150$ cells from three experiments (C). (D, E) Symbol graph (error bars: SD), ANOVA with Tukey's multiple comparison test, $n > 119$ (D) and $n > 152$ (E) measurements from three experiments each. P-values: ***$P < 0.01$. Scale bars: 1 μm.
Source data are available online for this figure.

 © 2023 The Authors

20 of 19 EMBO reports e57232 | 2023 © 2023 The Authors

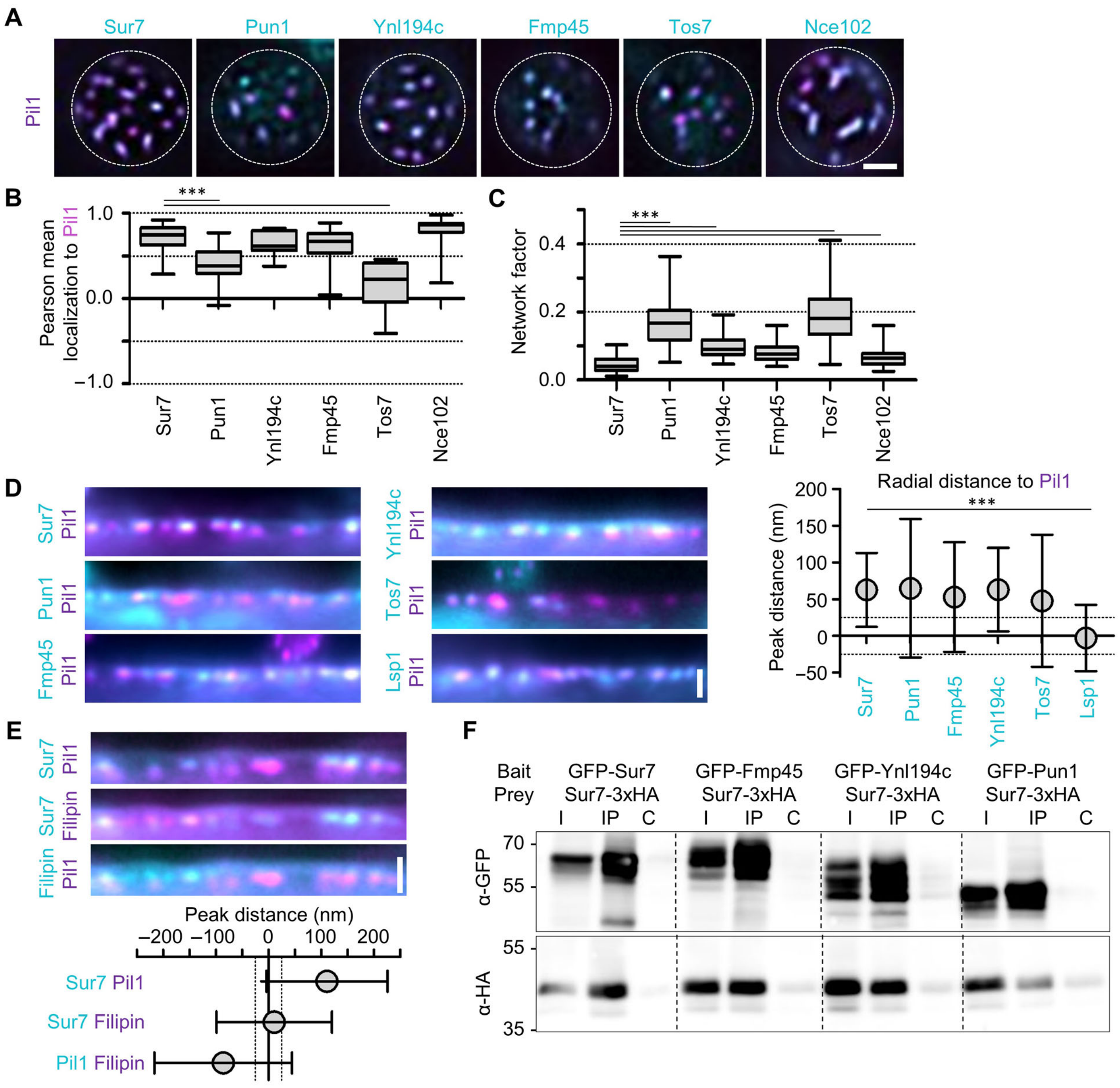

Figure EV1.

Figure EV2. Super-resolution imaging of Sur7 tetraspanners—Supplement.

A STED images of indicated tetraspanners expressed from the *PMA1* promoter and fused to the Halo tag.

B Two-color STED and confocal images of WT cells expressing endogenously tagged Sur7-Halo (STED, magenta) and Pil1-mNeGr (confocal, cyan).

C Effect of indicated lipid perturbations on lateral distribution of Sur7-mNeGr.

D Lateral distribution of Sur7-mNeGr quantified by the network factor in strains shown in (C).

Data information: (D) Boxplot (interquartile range (box), min to max spread (whiskers) and median values (line in boxes)), ANOVA with Dunnett's multiple comparison test, $n = 30$–227 cells from three experiments. P-values: ***$P < 0.01$. Scale bars: 1 µm, 200 nm (zoom).
Source data are available online for this figure.

▶

© 2023 The Authors

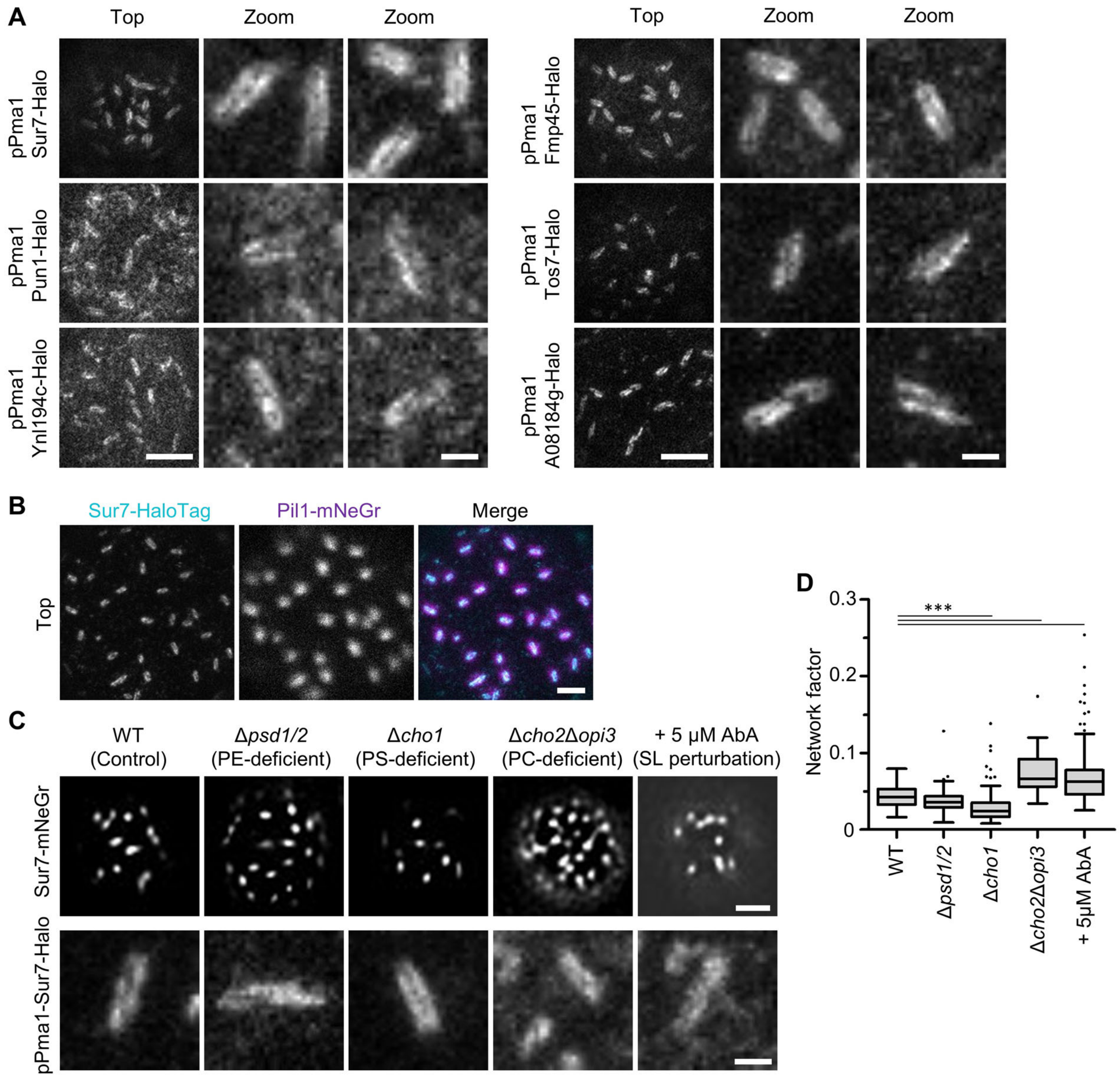

Figure EV2.

 © 2023 The Authors

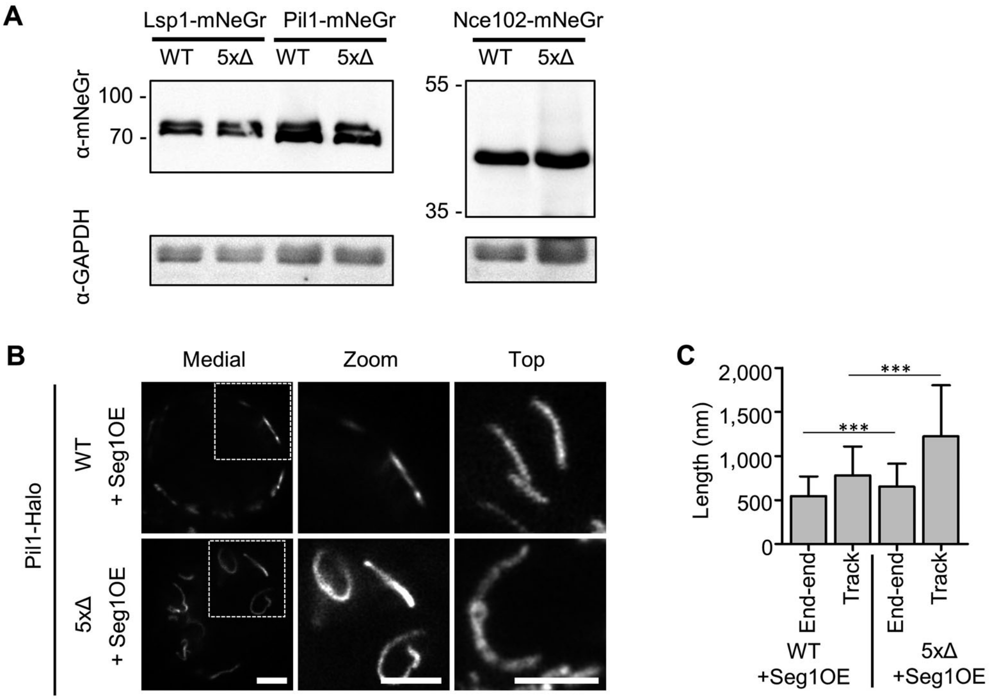

Figure EV3. Sur7 tetraspanners prevent closure of MCC/eisosome furrows—Supplement.

A Western blot analysis depicting the expression of endogenous mNeGr fusions to Lsp1, Pil1 and Nce102 in WT and 5xΔ cells.
B STED microscopy of endogenously tagged Pil1-Halo in WT and 5xΔ cells that overexpress Seg1 from the GPD promoter (OE).
C Quantification of track length for Pil1-Halo positive structures in (B).

Data information: (C) Bar graph (error bars: SD), unpaired *t*-test, $n = 53$–175 tracks from three experiments. *P*-values: ***$P < 0.01$. Scale bars: 1 μm.
Source data are available online for this figure.

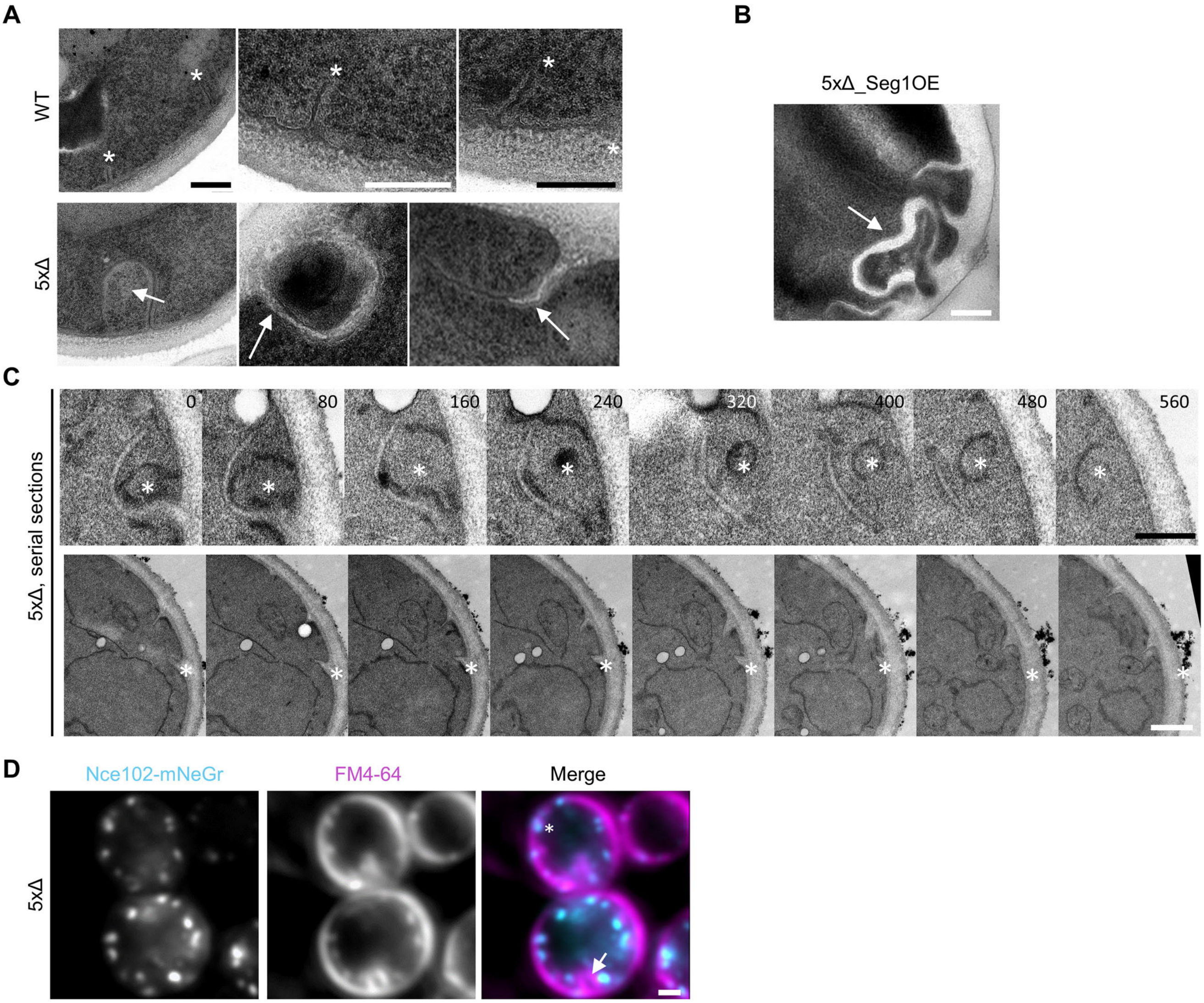

Figure EV4. Sur7 tetraspanners prevent closure of MCC/eisosome furrows – Supplement.

A TEM micrographs of ultrathin sections of WT and 5xΔ. Asterisks indicate perpendicular sections through MCC/eisosome furrows. Arrows indicate abnormal PM invaginations.

B TEM micrograph of ultrathin sections of a 5xΔ cell overexpressing Seg1 from the GPD promoter (OE). The arrow indicates an abnormal PM invagination.

C Exemplary serial TEM sections of 5xΔ cells illustrating the sheet-like invaginations filled with electron translucent cell wall material. Asterisks indicate positions of invaginations. Sample depth is indicated in nm.

D 5xΔ cells expressing Nce102-mNeGr (cyan) and labeled with the membrane dye FM4-64 (magenta). Asterisk indicates a Nce102-positive tube that is labeled by FM4-64. Arrow indicates a large FM4-64 positive invagination that does not contain Nce102.

Data information: Scale bars: 200 nm (A–C), 1 μm (D).
Source data are available online for this figure.

 © 2023 The Authors

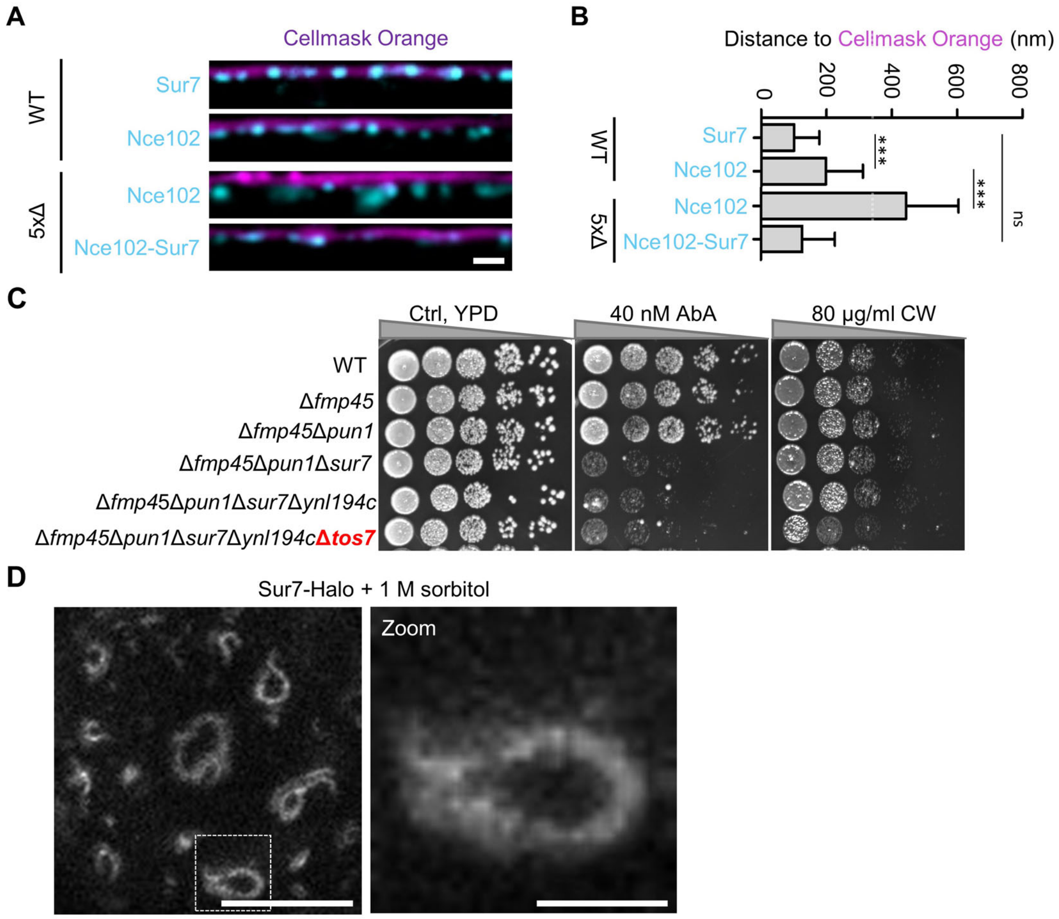

Figure EV5. Mechanistic basis of Sur7 function in subdomain organization—Supplement.

A Linearized profiles of WT cells expressing indicated GFP fusions (cyan) stained with the PM dye Cellmask Orange (magenta).

B Bar graph representing quantification of the radial distances between indicated protein and Cellmask Orange in (A).

C Growth assays with successive deletions of Sur7 tetraspanners. Cells were grown on YPD plates containing 40 nM AbA or 40 µg/ml calcofluor white (CW) at 30°C for 48 h.

D STED image of WT cells expressing Sur7-Halo from the *PMA1* promoter after addition of 1 M sorbitol for 5 min.

Data information: (B) Bar graph (error bars: SD), ANOVA with Tukey's multiple comparison test, $n > 133$ measurements from three experiments. *P*-values: ***$P < 0.01$, ns: not significant. Scale bars: 1 µm, 200 nm (zoom).
Source data are available online for this figure.

© 2023 The Authors
