# Supplementary figures and images for: Tetraspanner‐based nanodomains modulate BAR domain‐induced membrane curvature (part 1 of 3)
Source: EMBO Rep. 2023 Oct 30;24(12):e57232. doi: 10.15252/embr.202357232 (PMC10702824; doi:10.15252/embr.202357232)

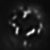

Supplement: Supplementary file 7 — Source Data for Expanded View [file EMBR-24-e57232-s004.zip › Figure EV1/EV1A/Nce102GFP-Pil1RFP.tif]

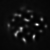

Supplement: Supplementary file 7 — Source Data for Expanded View [file EMBR-24-e57232-s004.zip › Figure EV1/EV1A/Sur7GFP-Pil1RFP.tif]

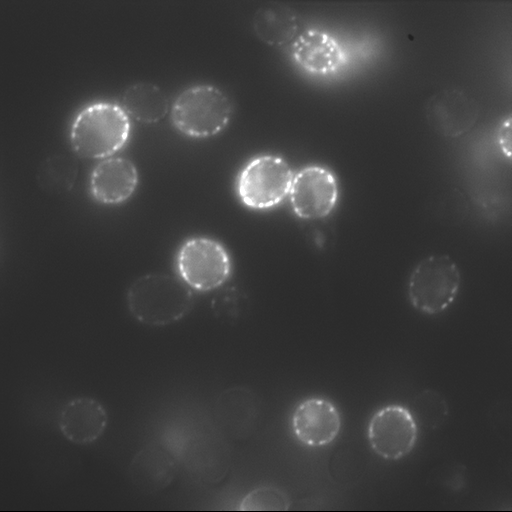

Supplement: Supplementary file 7 — Source Data for Expanded View [file EMBR-24-e57232-s004.zip › Figure EV1/EV1D/Fmp45mNeGr_Pil1RFP_cell.tif]

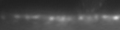

Supplement: Supplementary file 7 — Source Data for Expanded View [file EMBR-24-e57232-s004.zip › Figure EV1/EV1D/Fmp45mNeGr_Pil1RFP_linear.tif]

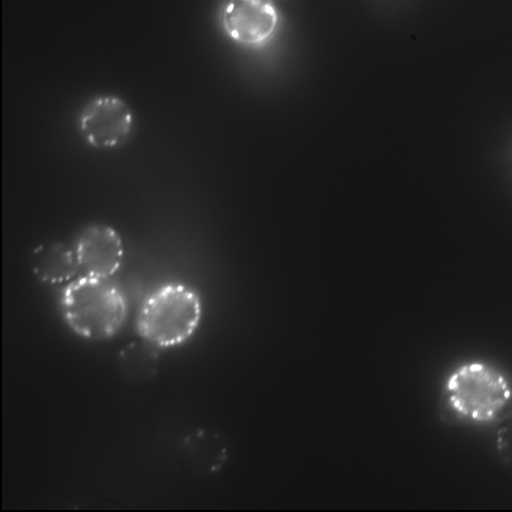

Supplement: Supplementary file 7 — Source Data for Expanded View [file EMBR-24-e57232-s004.zip › Figure EV1/EV1D/Lsp1mNeGr_Pil1RFP_cell.tif]

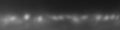

Supplement: Supplementary file 7 — Source Data for Expanded View [file EMBR-24-e57232-s004.zip › Figure EV1/EV1D/Lsp1mNeGr_Pil1RFP_linear.tif]

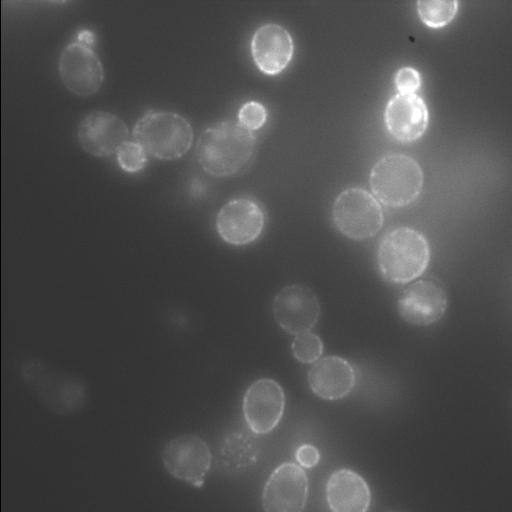

Supplement: Supplementary file 7 — Source Data for Expanded View [file EMBR-24-e57232-s004.zip › Figure EV1/EV1D/Pun1mNeGr_Pil1RFP_cell.tif]

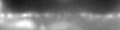

Supplement: Supplementary file 7 — Source Data for Expanded View [file EMBR-24-e57232-s004.zip › Figure EV1/EV1D/Pun1mNeGr_Pil1RFP_linear.tif]

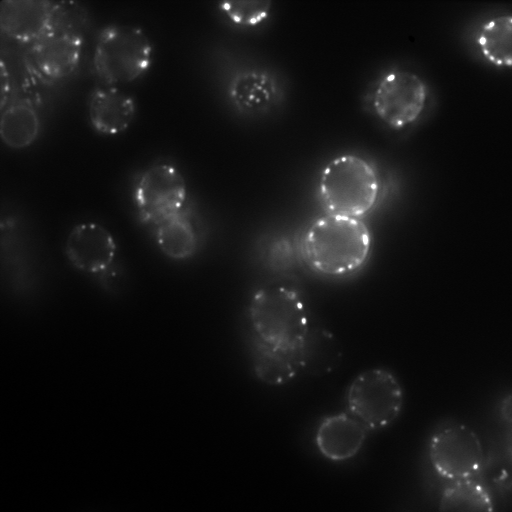

Supplement: Supplementary file 7 — Source Data for Expanded View [file EMBR-24-e57232-s004.zip › Figure EV1/EV1D/Sur7mNeGr_Pil1RFP_cell.tif]

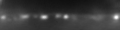

Supplement: Supplementary file 7 — Source Data for Expanded View [file EMBR-24-e57232-s004.zip › Figure EV1/EV1D/Sur7mNeGr_Pil1RFP_linear.tif]

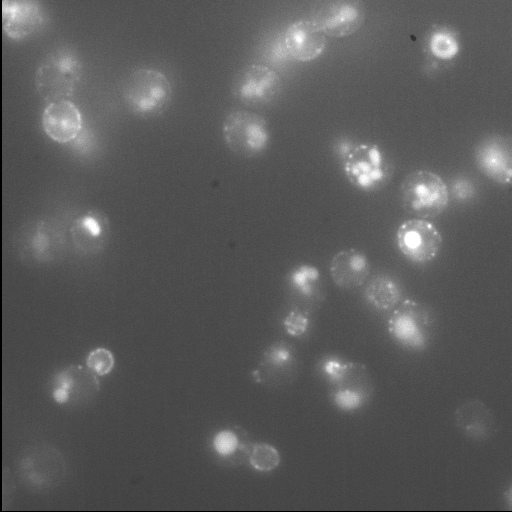

Supplement: Supplementary file 7 — Source Data for Expanded View [file EMBR-24-e57232-s004.zip › Figure EV1/EV1D/Tos7mNeGr_Pil1RFP_cell.tif]

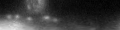

Supplement: Supplementary file 7 — Source Data for Expanded View [file EMBR-24-e57232-s004.zip › Figure EV1/EV1D/Tos7mNeGr_Pil1RFP_linear.tif]

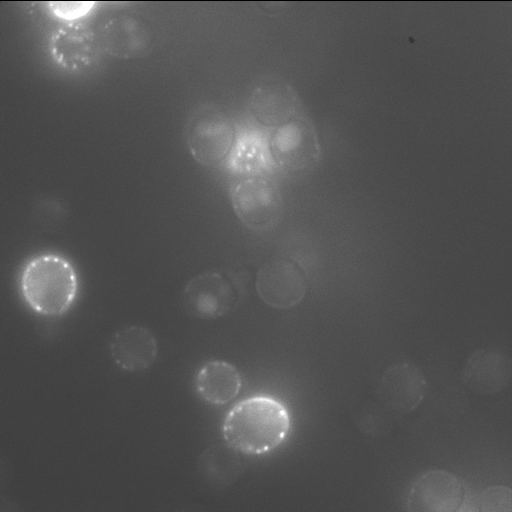

Supplement: Supplementary file 7 — Source Data for Expanded View [file EMBR-24-e57232-s004.zip › Figure EV1/EV1D/Ynl194cmNeGr_Pil1RFP_cell.tif]

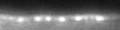

Supplement: Supplementary file 7 — Source Data for Expanded View [file EMBR-24-e57232-s004.zip › Figure EV1/EV1D/Ynl194cmNeGr_Pil1RFP_linear.tif]

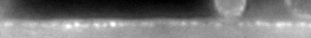

Supplement: Supplementary file 7 — Source Data for Expanded View [file EMBR-24-e57232-s004.zip › Figure EV1/EV1E/Filipin_Pil1RFP_linear.tif]

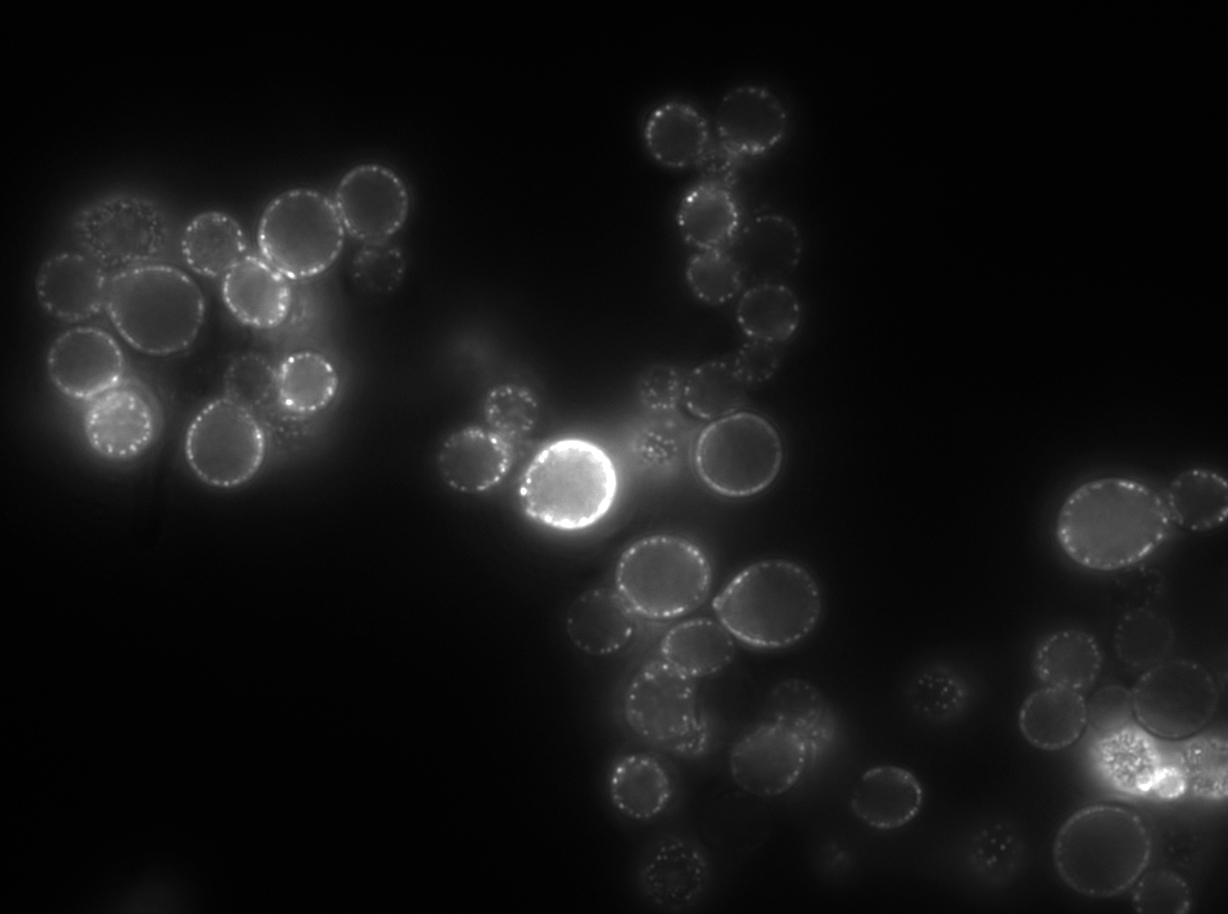

Supplement: Supplementary file 7 — Source Data for Expanded View [file EMBR-24-e57232-s004.zip › Figure EV1/EV1E/Pil1RFP_Sur7GFP_Filipin_cell.tif]

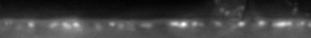

Supplement: Supplementary file 7 — Source Data for Expanded View [file EMBR-24-e57232-s004.zip › Figure EV1/EV1E/Sur7GFP_Filipin_linear.tif]

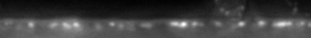

Supplement: Supplementary file 7 — Source Data for Expanded View [file EMBR-24-e57232-s004.zip › Figure EV1/EV1E/Sur7GFP_Pil1RFP_linear.tif]

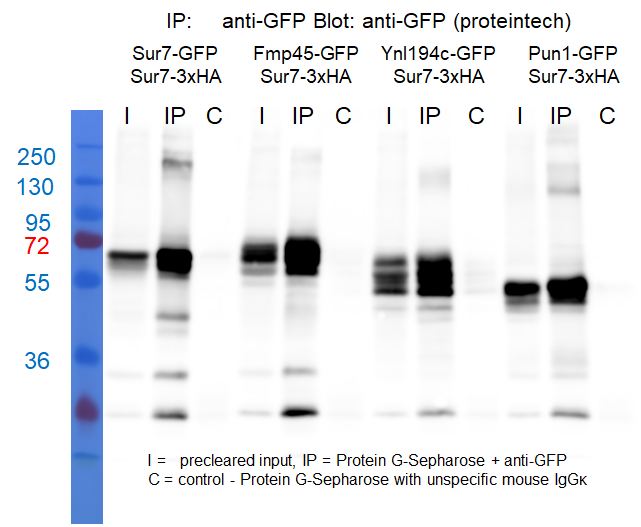

Supplement: Supplementary file 7 — Source Data for Expanded View [file EMBR-24-e57232-s004.zip › Figure EV1/EV1F/EV1F_GFP.jpg]

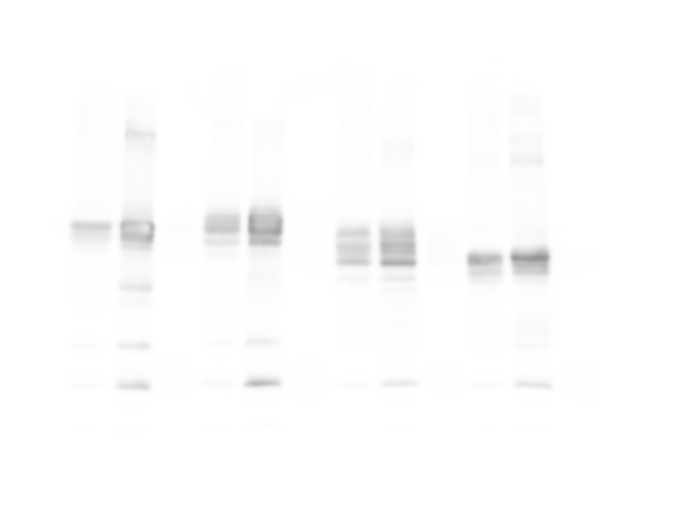

Supplement: Supplementary file 7 — Source Data for Expanded View [file EMBR-24-e57232-s004.zip › Figure EV1/EV1F/EV1F_GFP.tif]

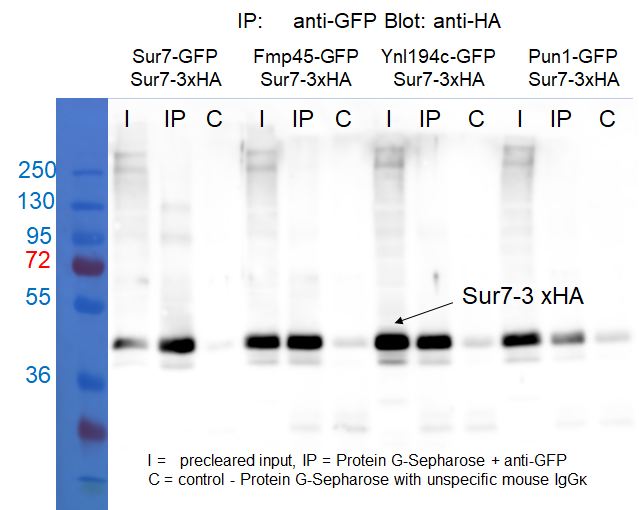

Supplement: Supplementary file 7 — Source Data for Expanded View [file EMBR-24-e57232-s004.zip › Figure EV1/EV1F/EV1F_HA.jpg]

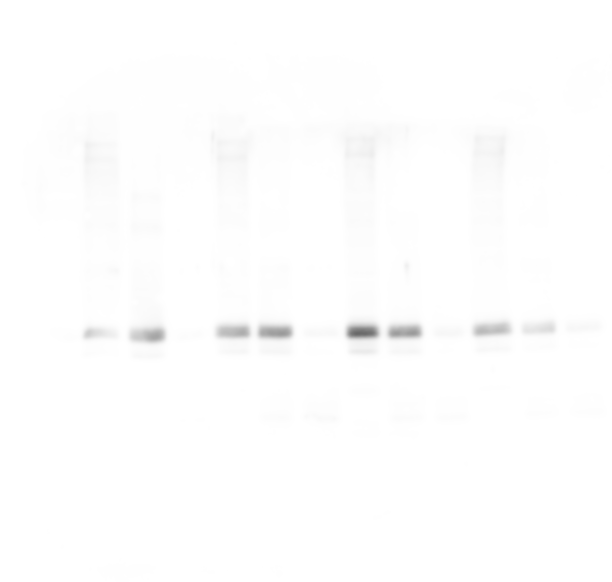

Supplement: Supplementary file 7 — Source Data for Expanded View [file EMBR-24-e57232-s004.zip › Figure EV1/EV1F/EV1F_HA.tif]

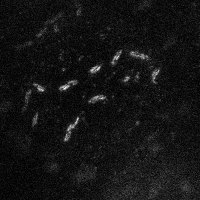

Supplement: Supplementary file 7 — Source Data for Expanded View [file EMBR-24-e57232-s004.zip › Figure EV2/EV2A/A08184gHalo_cell_zoom1.tif]

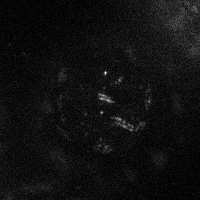

Supplement: Supplementary file 7 — Source Data for Expanded View [file EMBR-24-e57232-s004.zip › Figure EV2/EV2A/A08184gHalo_zoom2.tif]

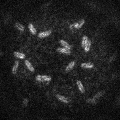

Supplement: Supplementary file 7 — Source Data for Expanded View [file EMBR-24-e57232-s004.zip › Figure EV2/EV2A/Fmp45Halo_cell_zoom12.tif]

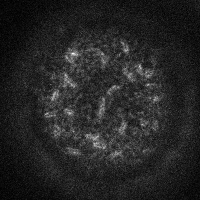

Supplement: Supplementary file 7 — Source Data for Expanded View [file EMBR-24-e57232-s004.zip › Figure EV2/EV2A/Pun1Halo_cell_zoom2.tif]

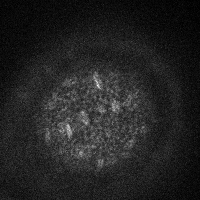

Supplement: Supplementary file 7 — Source Data for Expanded View [file EMBR-24-e57232-s004.zip › Figure EV2/EV2A/Pun1Halo_zoom1.tif]

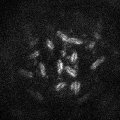

Supplement: Supplementary file 7 — Source Data for Expanded View [file EMBR-24-e57232-s004.zip › Figure EV2/EV2A/Sur7Halo_cell.tif]

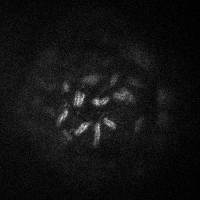

Supplement: Supplementary file 7 — Source Data for Expanded View [file EMBR-24-e57232-s004.zip › Figure EV2/EV2A/Sur7Halo_Zoom1.tif]

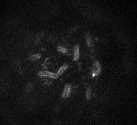

Supplement: Supplementary file 7 — Source Data for Expanded View [file EMBR-24-e57232-s004.zip › Figure EV2/EV2A/Sur7Halo_Zoom2.tif]

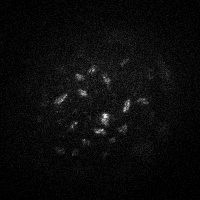

Supplement: Supplementary file 7 — Source Data for Expanded View [file EMBR-24-e57232-s004.zip › Figure EV2/EV2A/Tos7Halo_cell.tif]

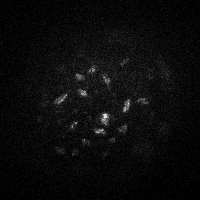

Supplement: Supplementary file 7 — Source Data for Expanded View [file EMBR-24-e57232-s004.zip › Figure EV2/EV2A/Tos7Halo_cell_zoom12.tif]

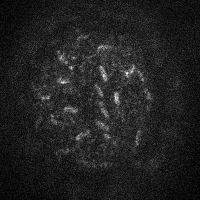

Supplement: Supplementary file 7 — Source Data for Expanded View [file EMBR-24-e57232-s004.zip › Figure EV2/EV2A/Ynl194cHalo_cell_zoom12.tif]

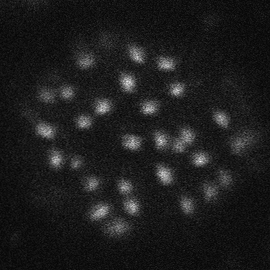

Supplement: Supplementary file 7 — Source Data for Expanded View [file EMBR-24-e57232-s004.zip › Figure EV2/EV2B/top_merge.tif]

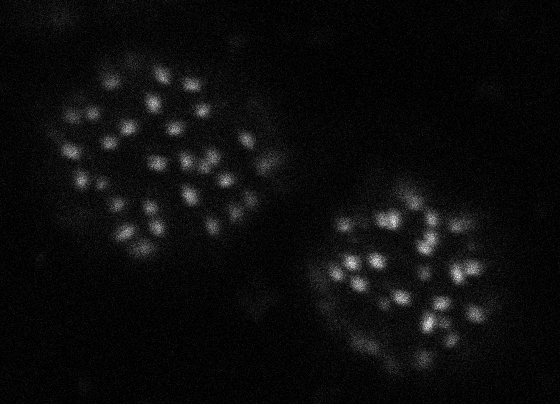

Supplement: Supplementary file 7 — Source Data for Expanded View [file EMBR-24-e57232-s004.zip › Figure EV2/EV2B/top_Pil1mNeGr.tif]

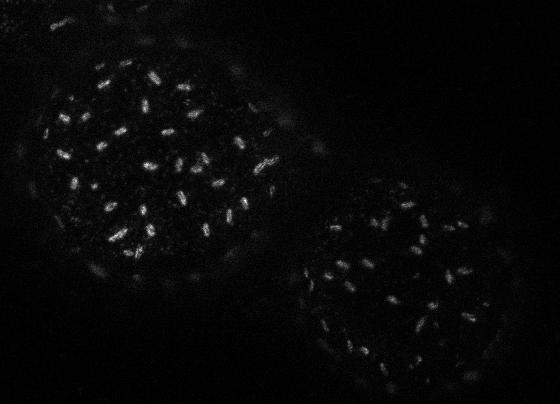

Supplement: Supplementary file 7 — Source Data for Expanded View [file EMBR-24-e57232-s004.zip › Figure EV2/EV2B/top_Sur7Halo.tif]

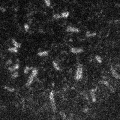

Supplement: Supplementary file 7 — Source Data for Expanded View [file EMBR-24-e57232-s004.zip › Figure EV2/EV2C/Sur7Halo_AbA_STED.tif]

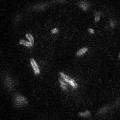

Supplement: Supplementary file 7 — Source Data for Expanded View [file EMBR-24-e57232-s004.zip › Figure EV2/EV2C/Sur7Halo_Dcho1_STED.tif]

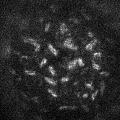

Supplement: Supplementary file 7 — Source Data for Expanded View [file EMBR-24-e57232-s004.zip › Figure EV2/EV2C/Sur7Halo_Dcho2Dopi3_STED.tif]

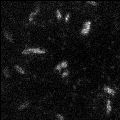

Supplement: Supplementary file 7 — Source Data for Expanded View [file EMBR-24-e57232-s004.zip › Figure EV2/EV2C/Sur7Halo_Dpsd1Dpsd2_STED.tif]

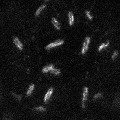

Supplement: Supplementary file 7 — Source Data for Expanded View [file EMBR-24-e57232-s004.zip › Figure EV2/EV2C/Sur7Halo_WT_STED.tif]

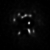

Supplement: Supplementary file 7 — Source Data for Expanded View [file EMBR-24-e57232-s004.zip › Figure EV2/EV2C/Sur7mNeGr_AbA_TIRF.tif]

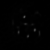

Supplement: Supplementary file 7 — Source Data for Expanded View [file EMBR-24-e57232-s004.zip › Figure EV2/EV2C/Sur7mNeGr_Dcho1_TIRF.tif]

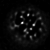

Supplement: Supplementary file 7 — Source Data for Expanded View [file EMBR-24-e57232-s004.zip › Figure EV2/EV2C/Sur7mNeGr_Dcho2Dopi3_TIRF.tif]

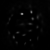

Supplement: Supplementary file 7 — Source Data for Expanded View [file EMBR-24-e57232-s004.zip › Figure EV2/EV2C/Sur7mNeGr_Dpsd1Dpsd2_TIRF.tif]

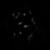

Supplement: Supplementary file 7 — Source Data for Expanded View [file EMBR-24-e57232-s004.zip › Figure EV2/EV2C/Sur7mNeGr_WT_TIRF.tif]

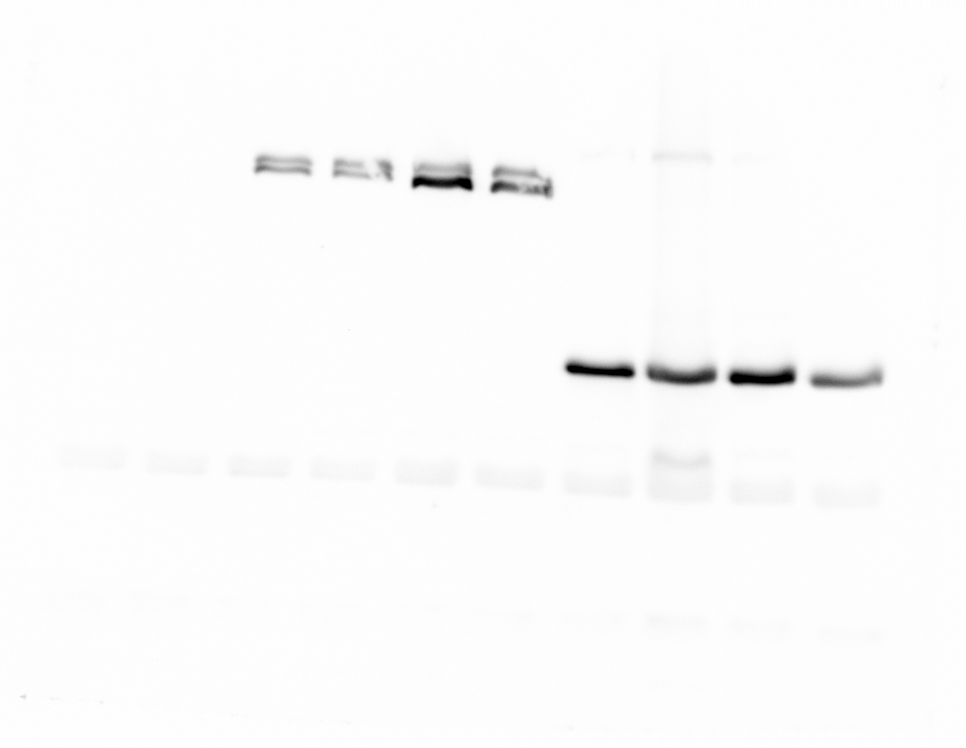

Supplement: Supplementary file 7 — Source Data for Expanded View [file EMBR-24-e57232-s004.zip › Figure EV3/EV3 A/blot.tif]

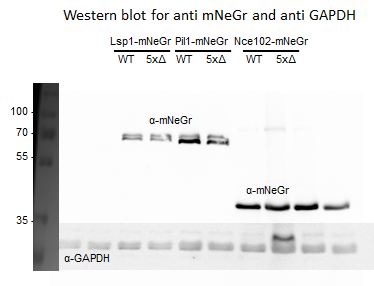

Supplement: Supplementary file 7 — Source Data for Expanded View [file EMBR-24-e57232-s004.zip › Figure EV3/EV3 A/Blot_annotated.jpg]

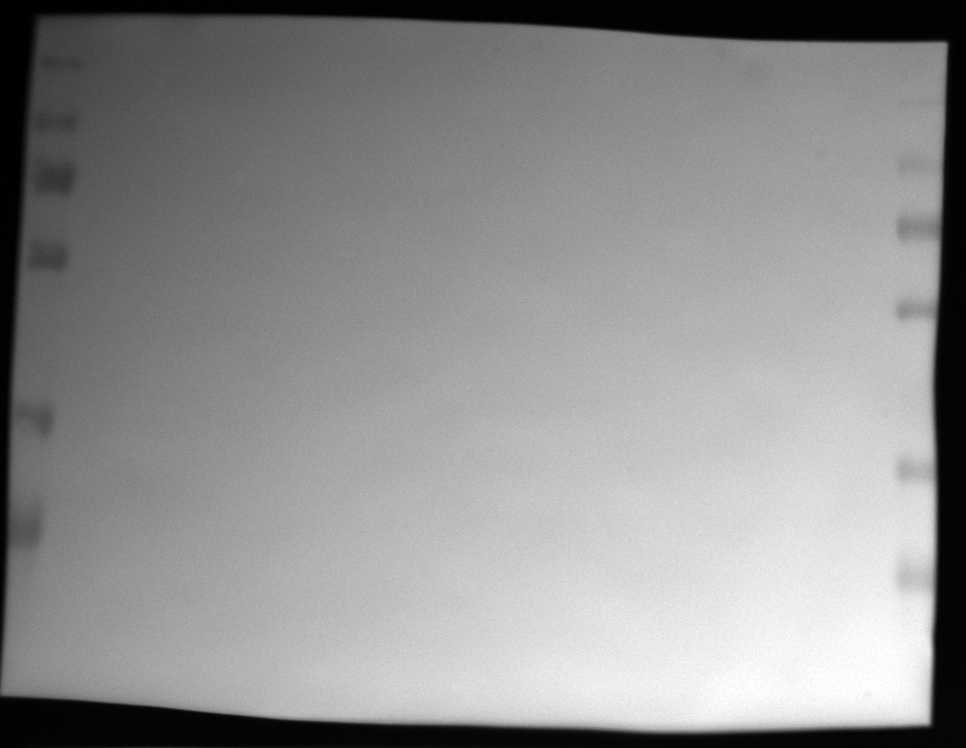

Supplement: Supplementary file 7 — Source Data for Expanded View [file EMBR-24-e57232-s004.zip › Figure EV3/EV3 A/marker.tif]

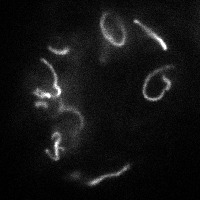

Supplement: Supplementary file 7 — Source Data for Expanded View [file EMBR-24-e57232-s004.zip › Figure EV3/EV3 B/Seg1OE_5xKO_medial_zoom.tif]

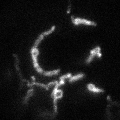

Supplement: Supplementary file 7 — Source Data for Expanded View [file EMBR-24-e57232-s004.zip › Figure EV3/EV3 B/Seg1OE_5xKO_top.tif]

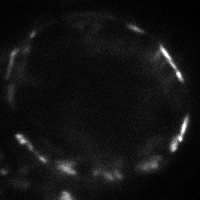

Supplement: Supplementary file 7 — Source Data for Expanded View [file EMBR-24-e57232-s004.zip › Figure EV3/EV3 B/Seg1OE_WT_medial_zoom.tif]

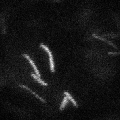

Supplement: Supplementary file 7 — Source Data for Expanded View [file EMBR-24-e57232-s004.zip › Figure EV3/EV3 B/Seg1OE_WT_top.tif]

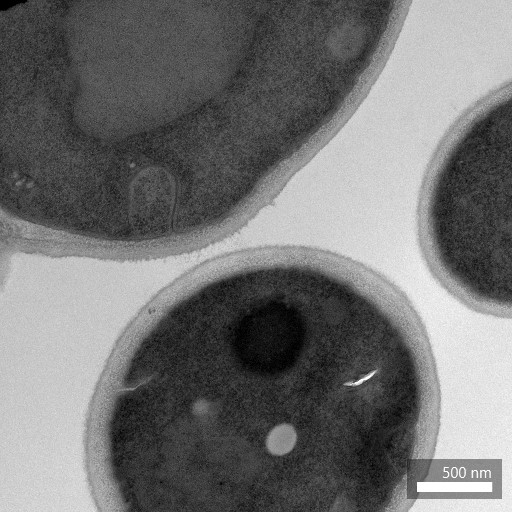

Supplement: Supplementary file 7 — Source Data for Expanded View [file EMBR-24-e57232-s004.zip › Figure EV4/EV4A/TEM_Detail_5xKO_1.tif]

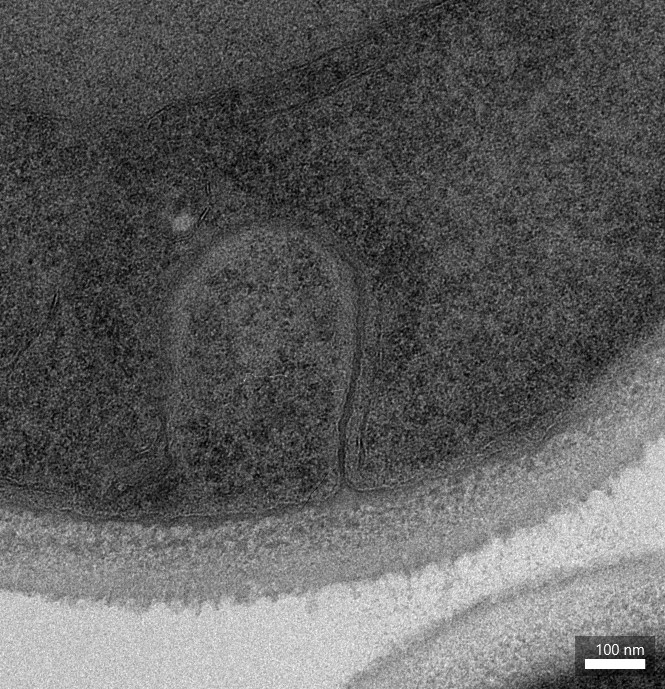

Supplement: Supplementary file 7 — Source Data for Expanded View [file EMBR-24-e57232-s004.zip › Figure EV4/EV4A/TEM_Detail_5xKO_1b.tif]

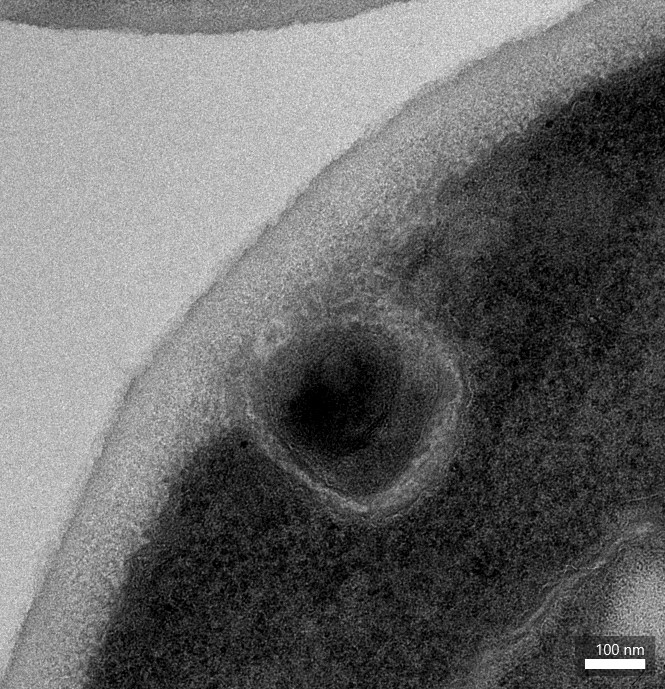

Supplement: Supplementary file 7 — Source Data for Expanded View [file EMBR-24-e57232-s004.zip › Figure EV4/EV4A/TEM_Detail_5xKO_2.tif]

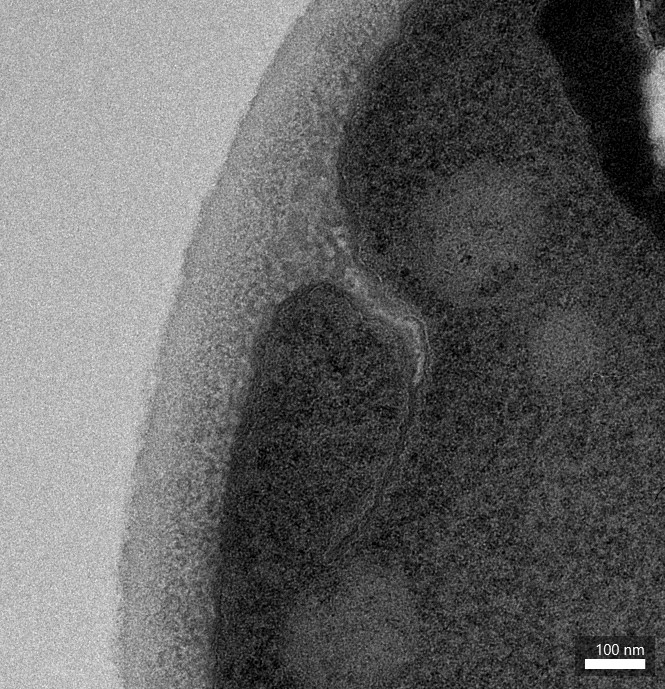

Supplement: Supplementary file 7 — Source Data for Expanded View [file EMBR-24-e57232-s004.zip › Figure EV4/EV4A/TEM_Detail_5xKO_3.tif]

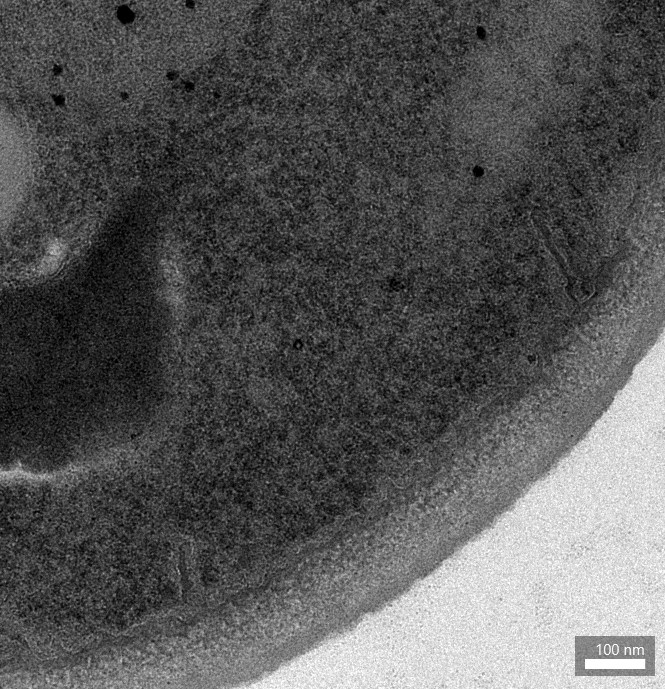

Supplement: Supplementary file 7 — Source Data for Expanded View [file EMBR-24-e57232-s004.zip › Figure EV4/EV4A/TEM_Detail_WT_1.tif]

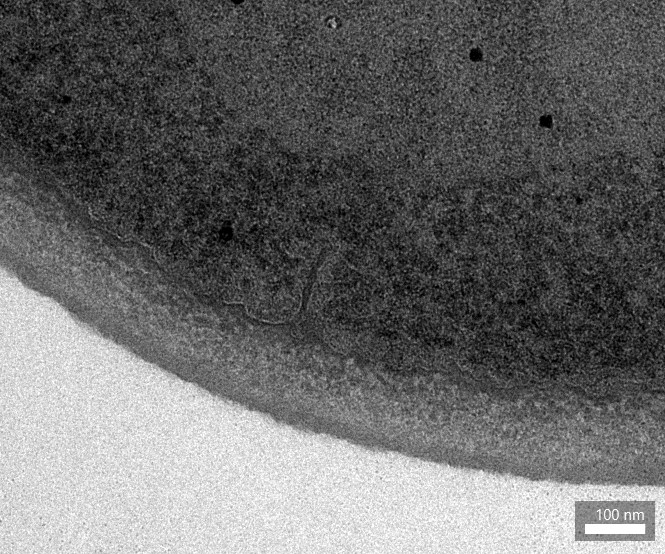

Supplement: Supplementary file 7 — Source Data for Expanded View [file EMBR-24-e57232-s004.zip › Figure EV4/EV4A/TEM_Detail_WT_2.tif]

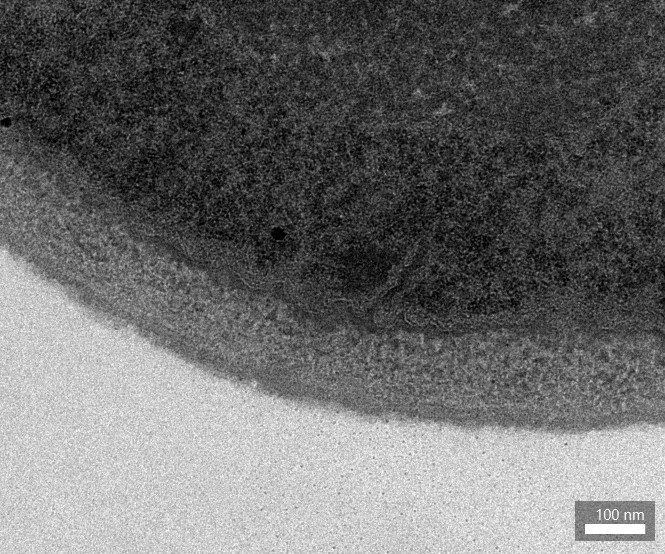

Supplement: Supplementary file 7 — Source Data for Expanded View [file EMBR-24-e57232-s004.zip › Figure EV4/EV4A/TEM_Detail_WT_3.tif]

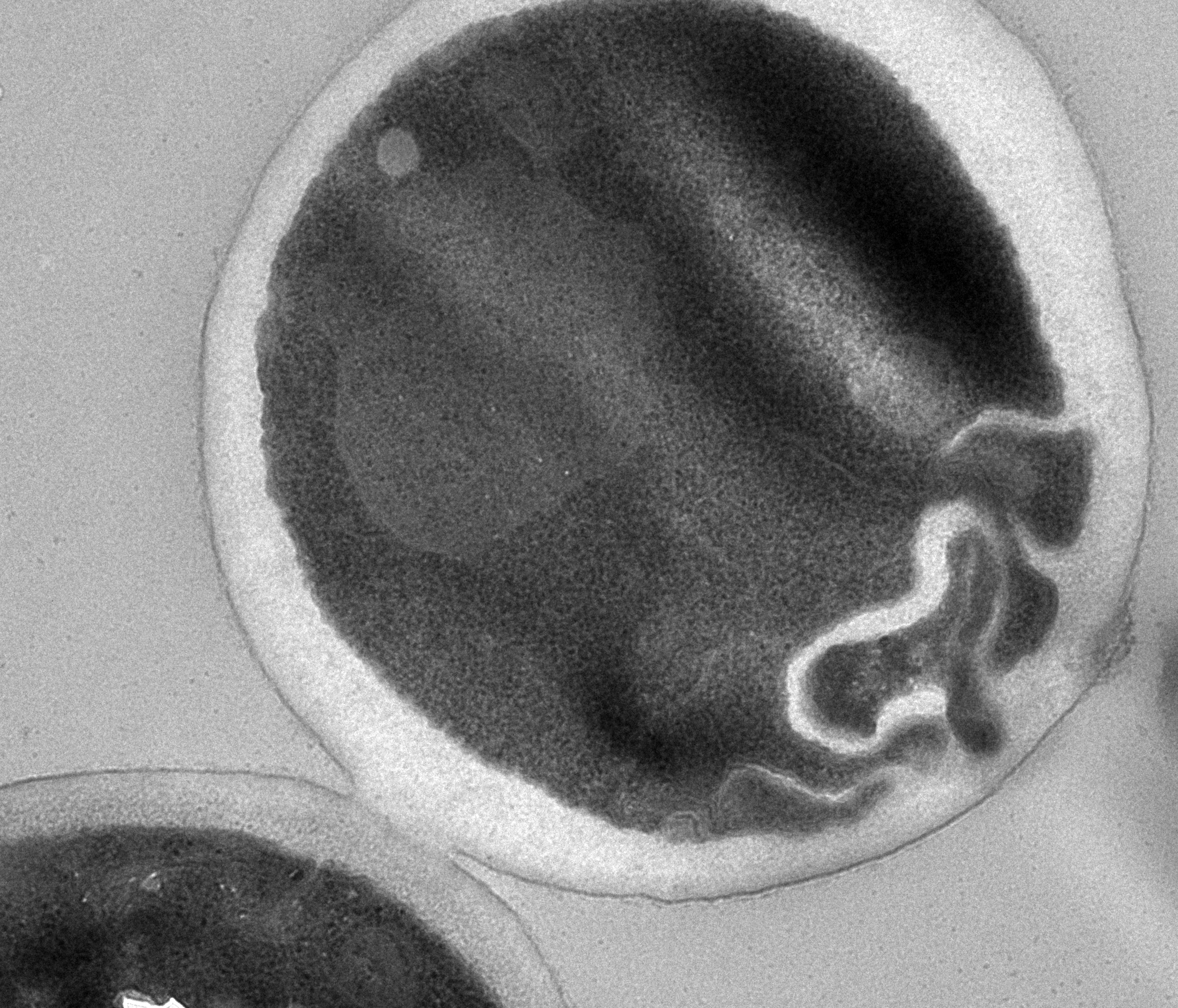

Supplement: Supplementary file 7 — Source Data for Expanded View [file EMBR-24-e57232-s004.zip › Figure EV4/EV4B/TEM_5xKO_Seg1OE.tif]

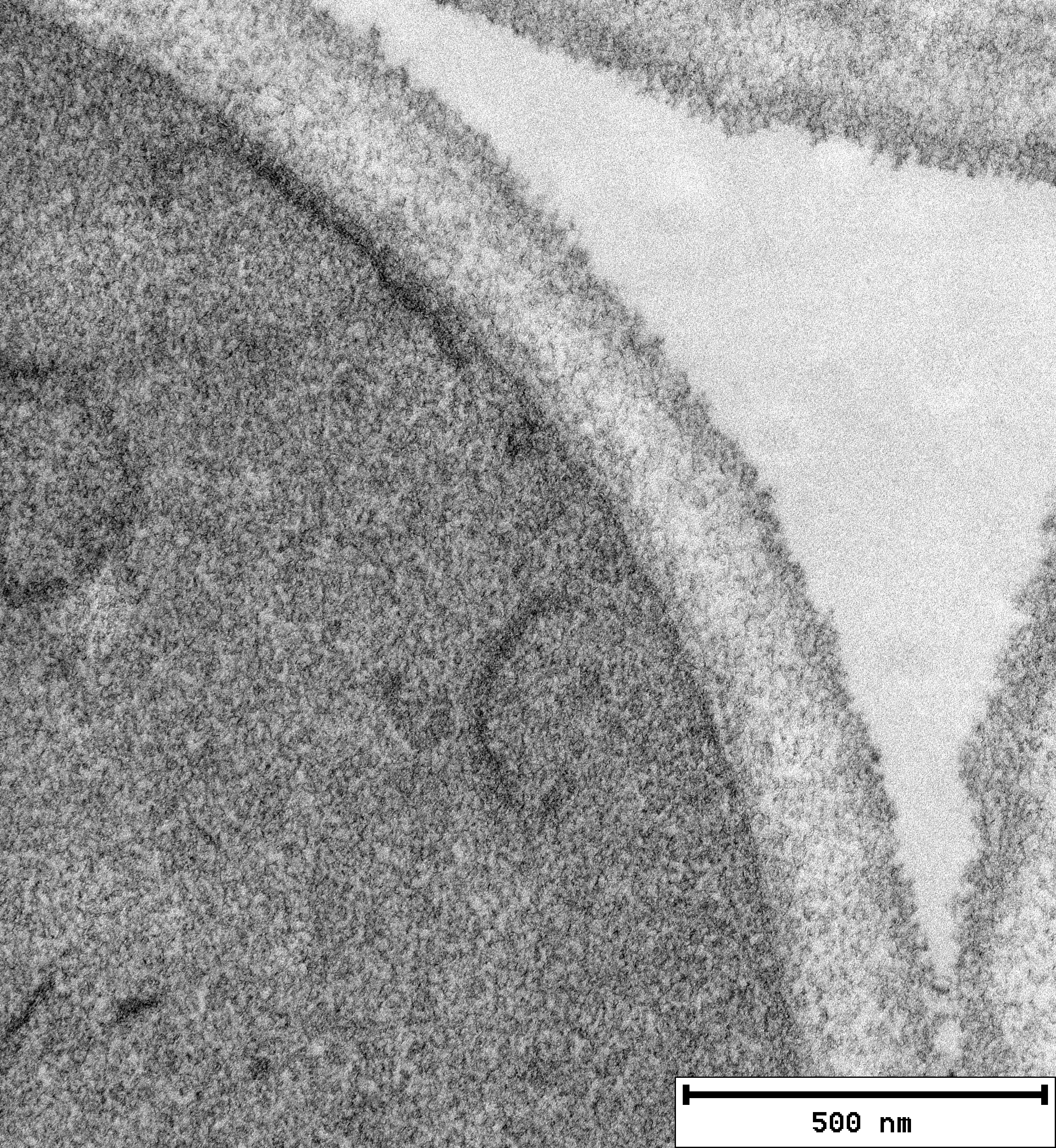

Supplement: Supplementary file 7 — Source Data for Expanded View [file EMBR-24-e57232-s004.zip › Figure EV4/EV4C/5xKO_serial_1_9x.tif]

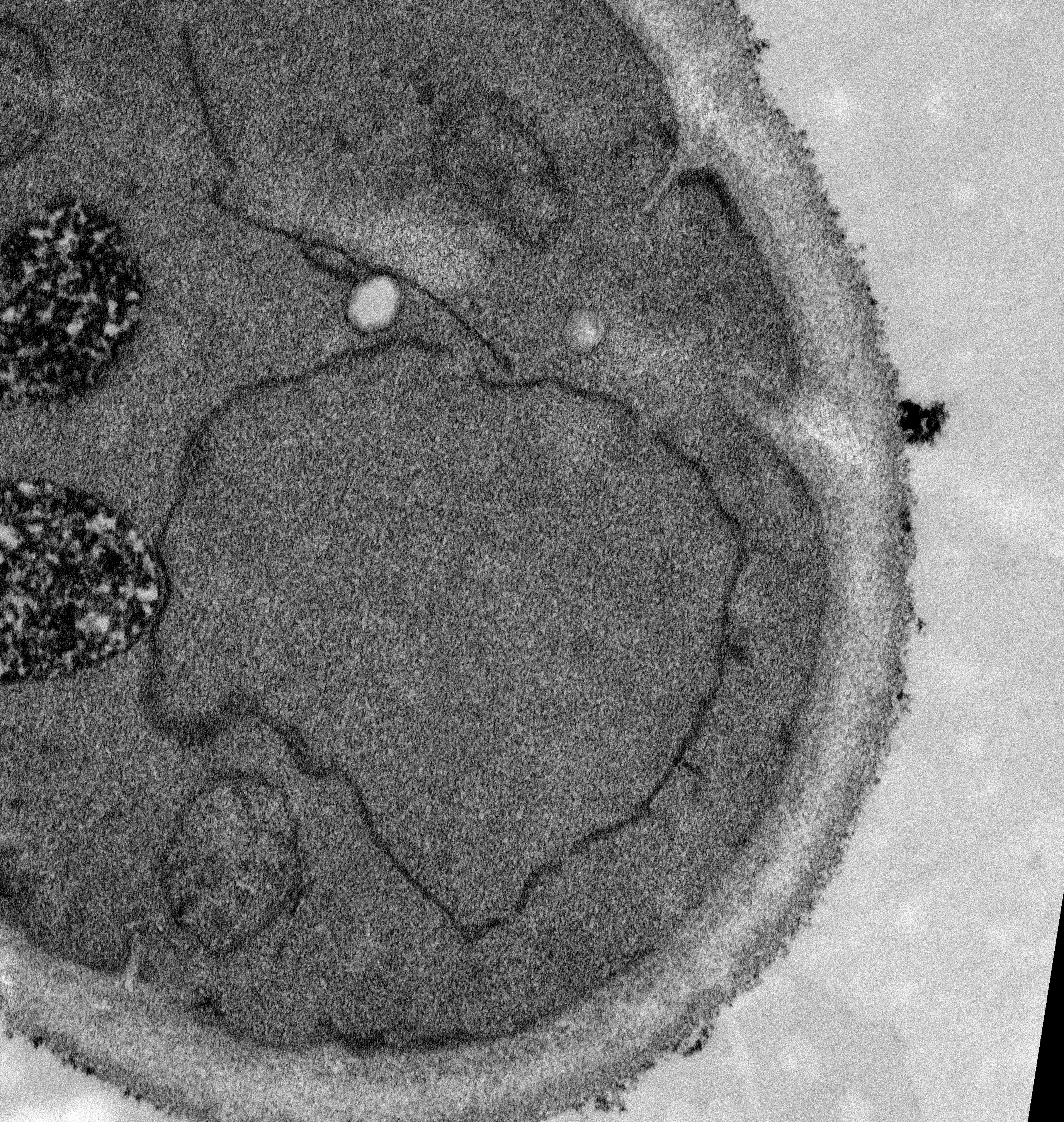

Supplement: Supplementary file 7 — Source Data for Expanded View [file EMBR-24-e57232-s004.zip › Figure EV4/EV4C/5xKO_serial_2_8x.tif]

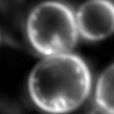

Supplement: Supplementary file 7 — Source Data for Expanded View [file EMBR-24-e57232-s004.zip › Figure EV4/EV4D/Nce102mNeGr_FM464.tif]

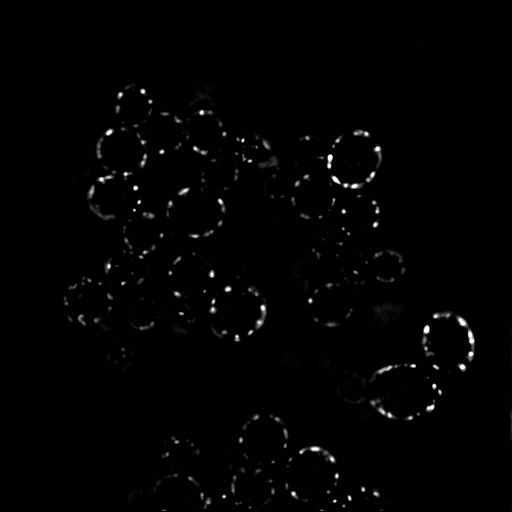

Supplement: Supplementary file 7 — Source Data for Expanded View [file EMBR-24-e57232-s004.zip › Figure EV5/EV5A/KO_Nce102-Sur7mNeGr_CMO_cell.tif]

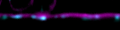

Supplement: Supplementary file 7 — Source Data for Expanded View [file EMBR-24-e57232-s004.zip › Figure EV5/EV5A/KO_Nce102-Sur7mNeGr_CMO_linear.tif]

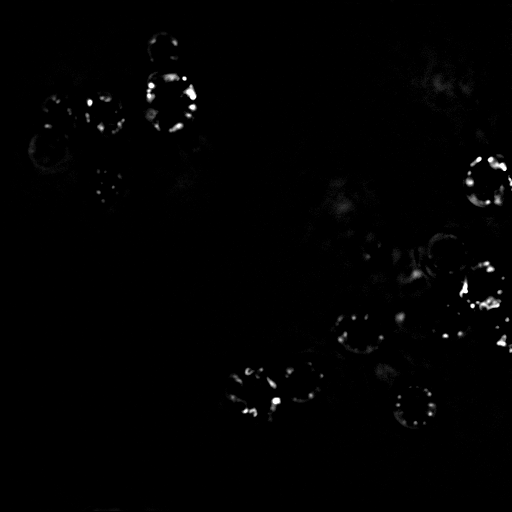

Supplement: Supplementary file 7 — Source Data for Expanded View [file EMBR-24-e57232-s004.zip › Figure EV5/EV5A/KO_Nce102mNeGr_CMO_cell.tif]

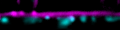

Supplement: Supplementary file 7 — Source Data for Expanded View [file EMBR-24-e57232-s004.zip › Figure EV5/EV5A/KO_Nce102mNeGr_CMO_linear.tif]

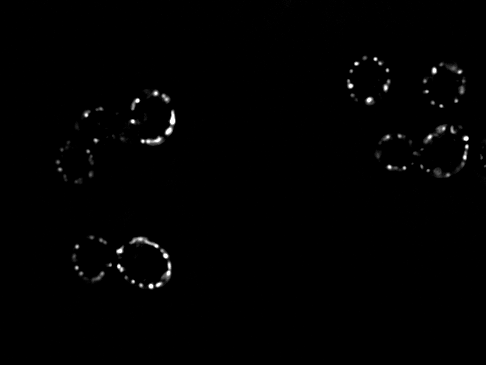

Supplement: Supplementary file 7 — Source Data for Expanded View [file EMBR-24-e57232-s004.zip › Figure EV5/EV5A/WT_Nce102mNeGr_CMO_cell.tif]

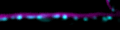

Supplement: Supplementary file 7 — Source Data for Expanded View [file EMBR-24-e57232-s004.zip › Figure EV5/EV5A/WT_Nce102mNeGr_CMO_linear.tif]

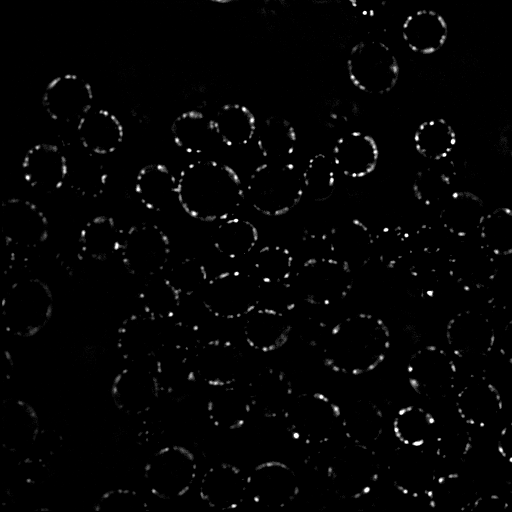

Supplement: Supplementary file 7 — Source Data for Expanded View [file EMBR-24-e57232-s004.zip › Figure EV5/EV5A/WT_Sur7mNeGr_CMO_cell.tif]

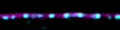

Supplement: Supplementary file 7 — Source Data for Expanded View [file EMBR-24-e57232-s004.zip › Figure EV5/EV5A/WT_Sur7mNeGr_CMO_linear.tif]

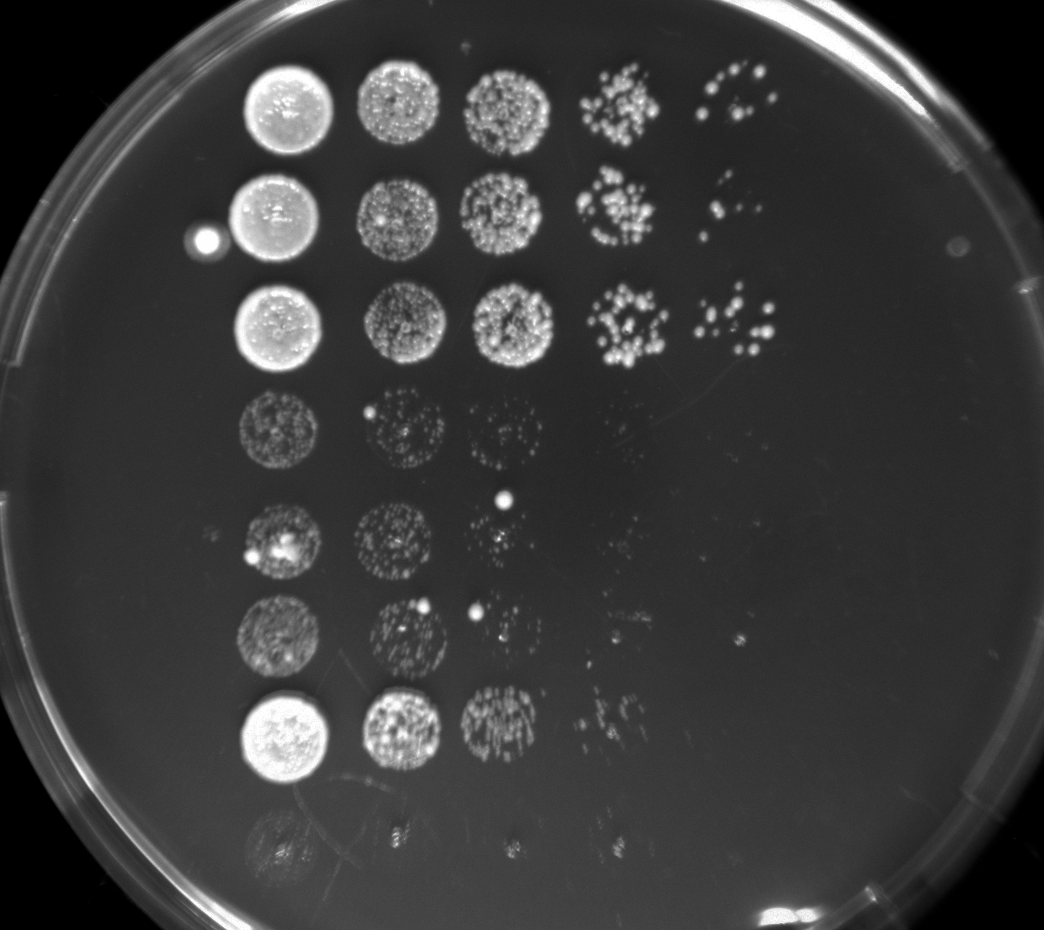

Supplement: Supplementary file 7 — Source Data for Expanded View [file EMBR-24-e57232-s004.zip › Figure EV5/EV5C/GrowthAssay_AbA.tif]

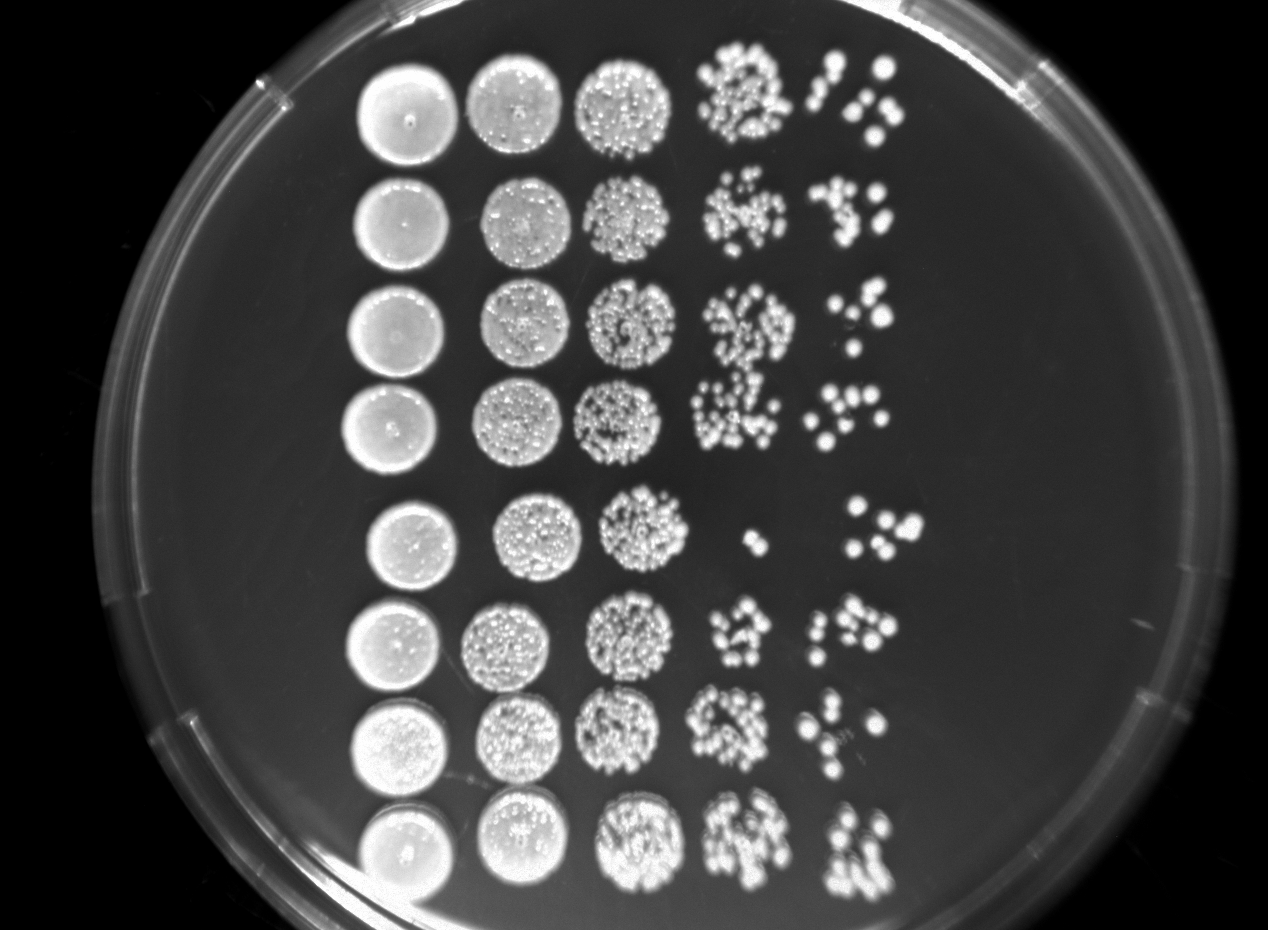

Supplement: Supplementary file 7 — Source Data for Expanded View [file EMBR-24-e57232-s004.zip › Figure EV5/EV5C/GrowthAssay_Ctrl.tif]

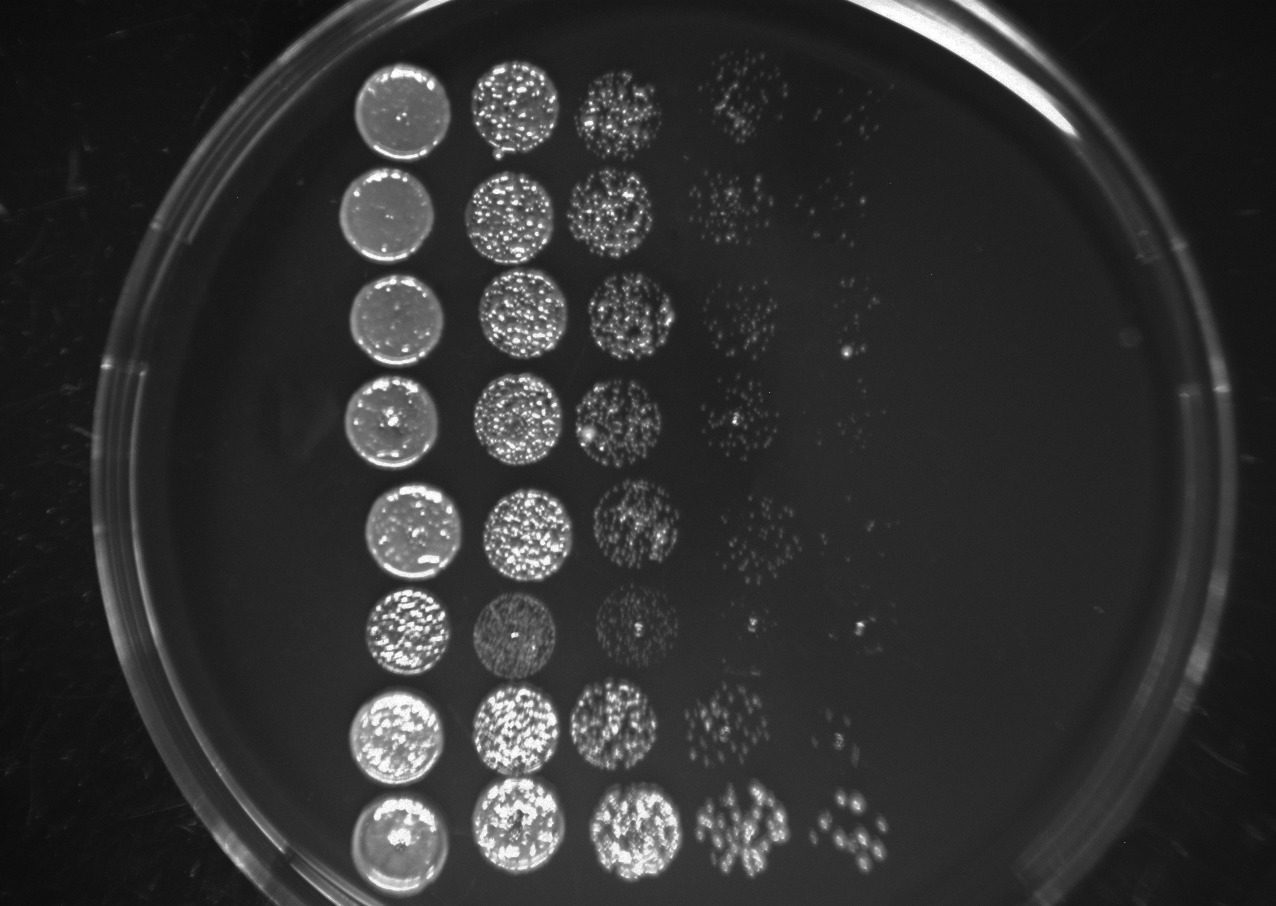

Supplement: Supplementary file 7 — Source Data for Expanded View [file EMBR-24-e57232-s004.zip › Figure EV5/EV5C/GrowthAssay_CW.tif]

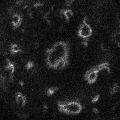

Supplement: Supplementary file 7 — Source Data for Expanded View [file EMBR-24-e57232-s004.zip › Figure EV5/EV5D/Sur7Halo_1Msorb_cell_zoom.tif]

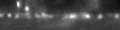

Supplement: Supplementary file 8 — Source Data for Figure 1 [file EMBR-24-e57232-s006.zip › Figure 1/1E/Sur7Nce102_straigthen.tif]

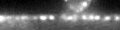

Supplement: Supplementary file 8 — Source Data for Figure 1 [file EMBR-24-e57232-s006.zip › Figure 1/1E/Sur7Sur7_straighten.tif]

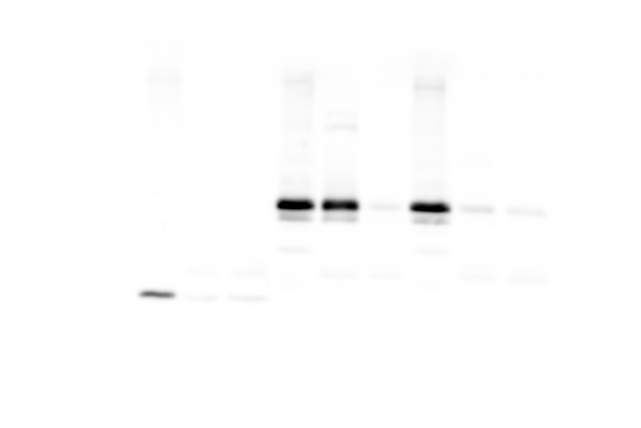

Supplement: Supplementary file 8 — Source Data for Figure 1 [file EMBR-24-e57232-s006.zip › Figure 1/1G/1+5bottom_Sur7GFP_Sur7HA_aHA.tif]

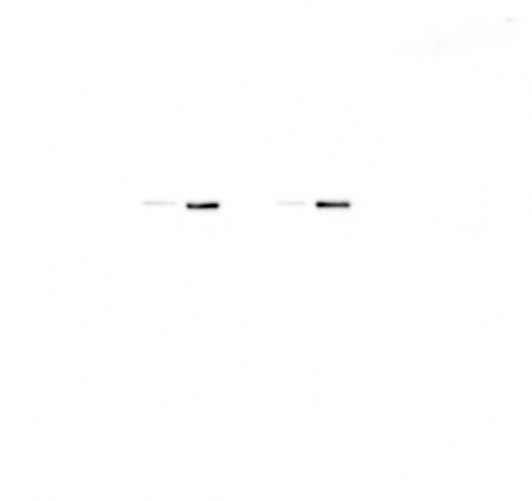

Supplement: Supplementary file 8 — Source Data for Figure 1 [file EMBR-24-e57232-s006.zip › Figure 1/1G/1+5top_Sur7GFP_Sur7HA_aGFP.tif]

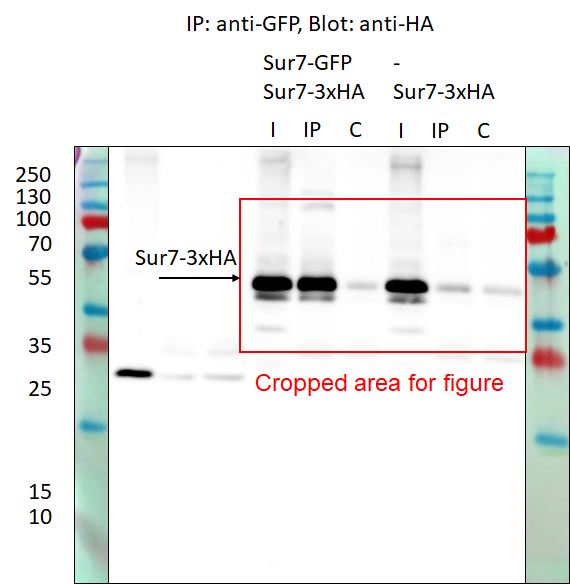

Supplement: Supplementary file 8 — Source Data for Figure 1 [file EMBR-24-e57232-s006.zip › Figure 1/1G/1bottom_Sur7GFP_Sur7HA_aHA.jpg]

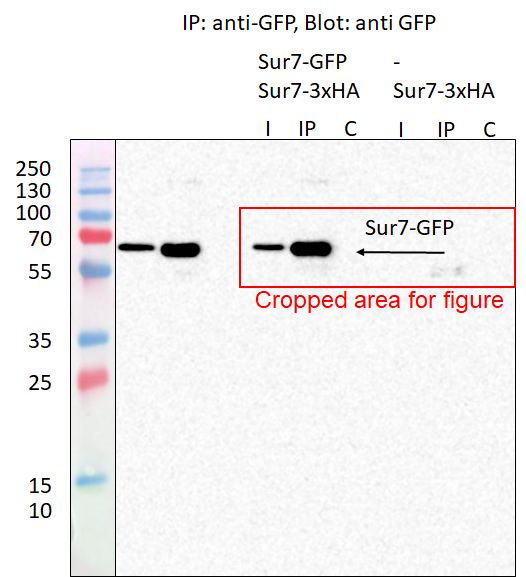

Supplement: Supplementary file 8 — Source Data for Figure 1 [file EMBR-24-e57232-s006.zip › Figure 1/1G/1top_Sur7GFP_Sur7HA_aGFP.jpg]

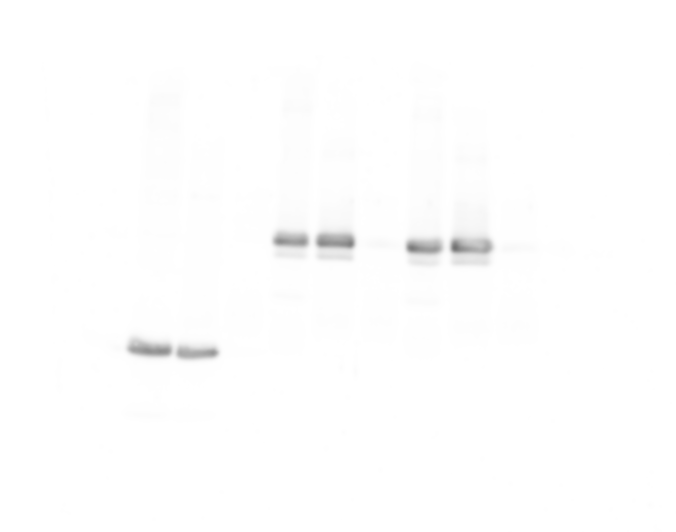

Supplement: Supplementary file 8 — Source Data for Figure 1 [file EMBR-24-e57232-s006.zip › Figure 1/1G/2bottom_Nc102GFP_Nce102HA_aHA.tif]

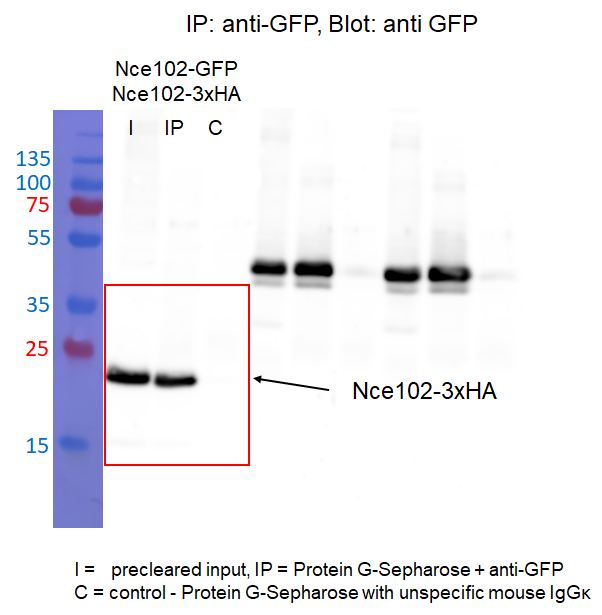

Supplement: Supplementary file 8 — Source Data for Figure 1 [file EMBR-24-e57232-s006.zip › Figure 1/1G/2bottom_Nce102GFP_Nce102HA_aHA.jpg]

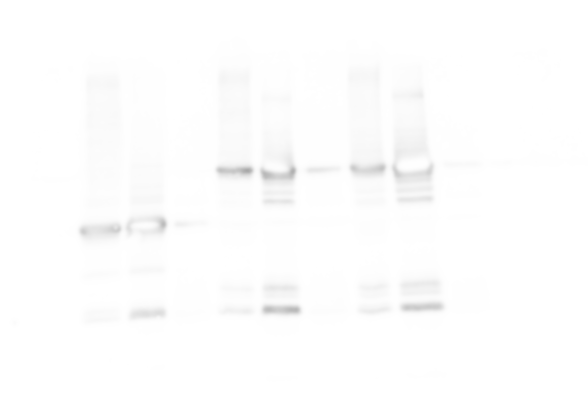

Supplement: Supplementary file 8 — Source Data for Figure 1 [file EMBR-24-e57232-s006.zip › Figure 1/1G/2top_Nc102GFP_Nce102HA_aGFP.tif]

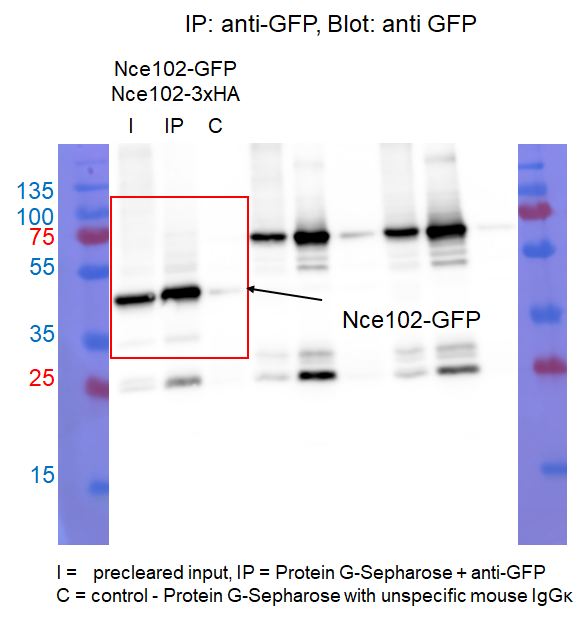

Supplement: Supplementary file 8 — Source Data for Figure 1 [file EMBR-24-e57232-s006.zip › Figure 1/1G/2top_Nce102GFP_Nce102HA_aGFP.jpg]

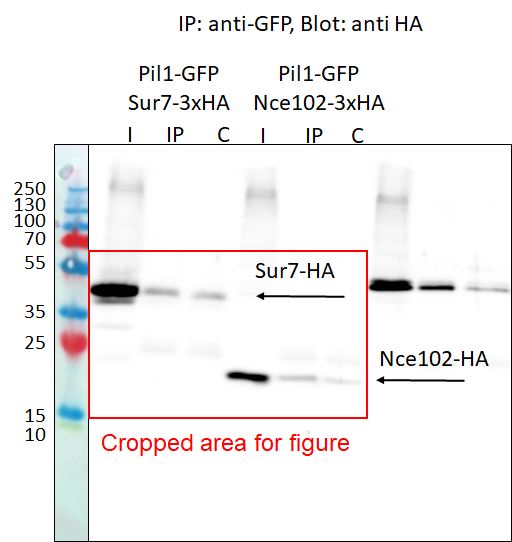

Supplement: Supplementary file 8 — Source Data for Figure 1 [file EMBR-24-e57232-s006.zip › Figure 1/1G/3bottom_Pil1GFP_Sur7HA_Nce102HA_aHA.jpg]

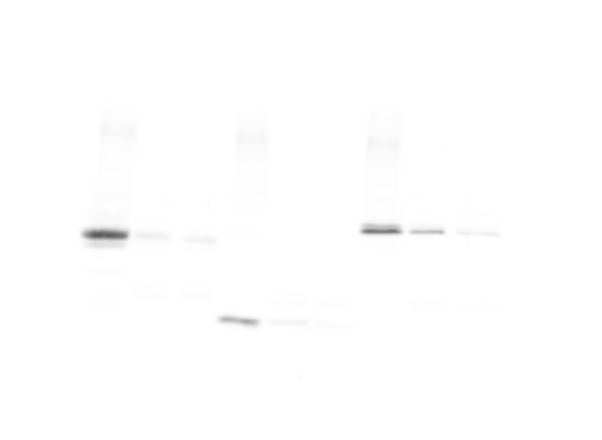

Supplement: Supplementary file 8 — Source Data for Figure 1 [file EMBR-24-e57232-s006.zip › Figure 1/1G/3bottom_Pil1GFP_Sur7HA_Nce102HA_aHA.tif]

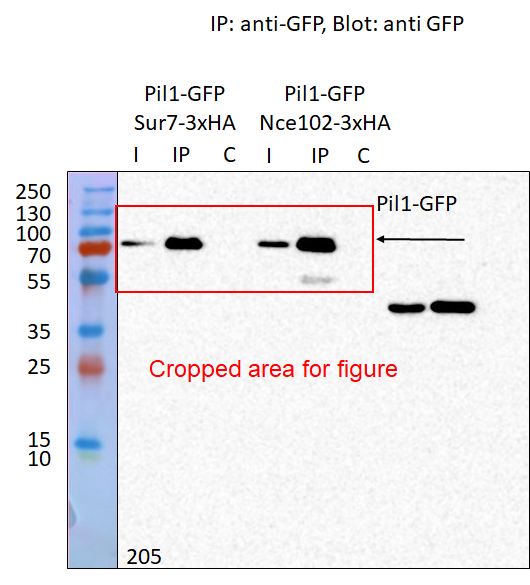

Supplement: Supplementary file 8 — Source Data for Figure 1 [file EMBR-24-e57232-s006.zip › Figure 1/1G/3top_Pil1GFP_Sur7HA_Nce102HA_aGFP.jpg]

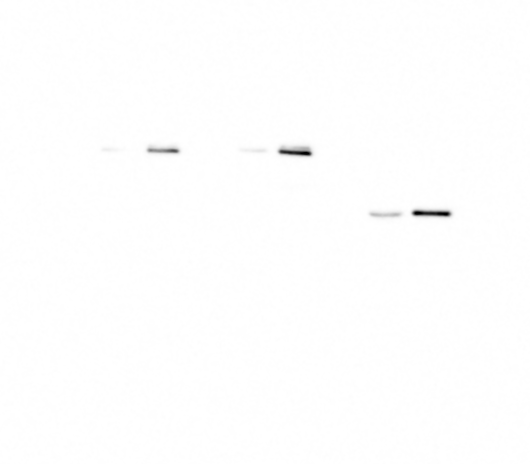

Supplement: Supplementary file 8 — Source Data for Figure 1 [file EMBR-24-e57232-s006.zip › Figure 1/1G/3top_Pil1GFP_Sur7HA_Nce102HA_aGFP.tif]

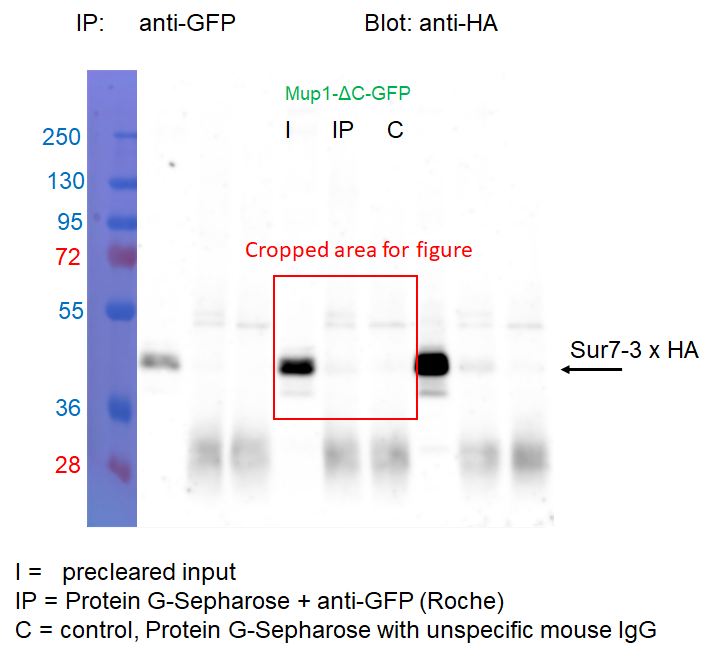

Supplement: Supplementary file 8 — Source Data for Figure 1 [file EMBR-24-e57232-s006.zip › Figure 1/1G/4bottom_Mup1GFP_Sur7HA_aHA.jpg]

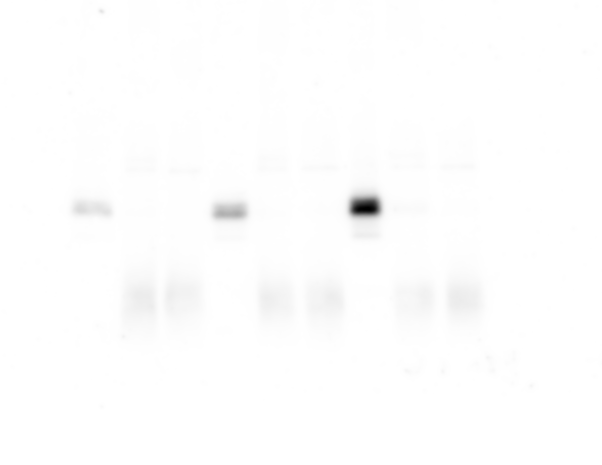

Supplement: Supplementary file 8 — Source Data for Figure 1 [file EMBR-24-e57232-s006.zip › Figure 1/1G/4bottom_Mup1GFP_Sur7HA_aHA.tif]

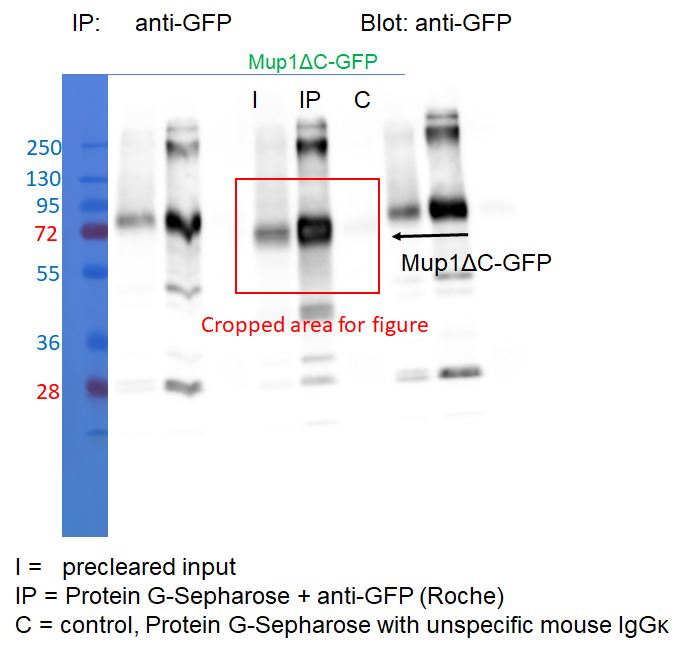

Supplement: Supplementary file 8 — Source Data for Figure 1 [file EMBR-24-e57232-s006.zip › Figure 1/1G/4top_Mup1GFP_Sur7HA_aGFP.jpg]

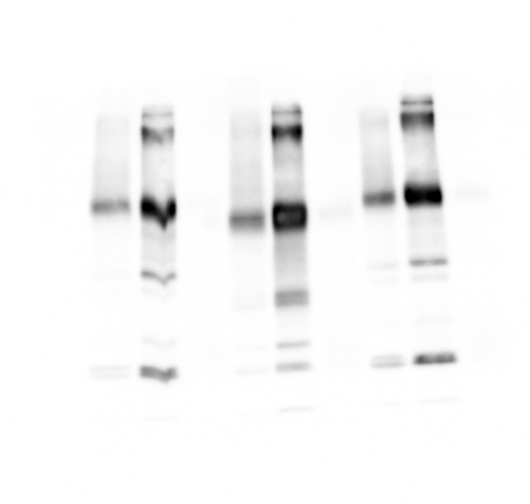

Supplement: Supplementary file 8 — Source Data for Figure 1 [file EMBR-24-e57232-s006.zip › Figure 1/1G/4top_Mup1GFP_Sur7HA_aGFP.tif]

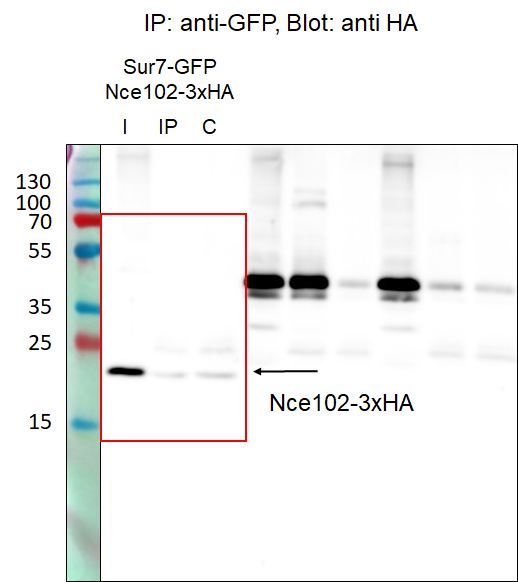

Supplement: Supplementary file 8 — Source Data for Figure 1 [file EMBR-24-e57232-s006.zip › Figure 1/1G/5bottom_Sur7GFP_Nce102HA_aHA.jpg]

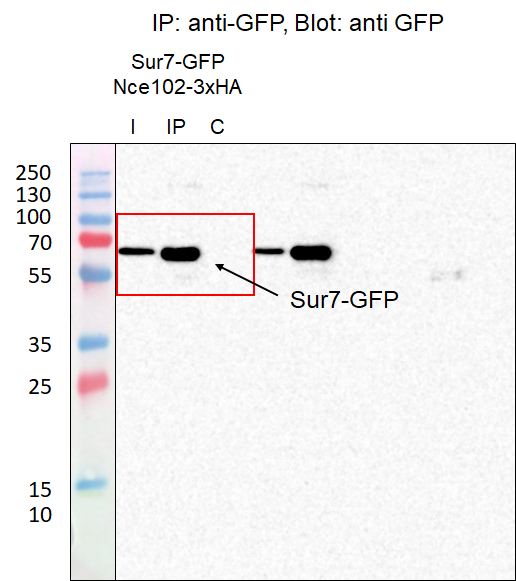

Supplement: Supplementary file 8 — Source Data for Figure 1 [file EMBR-24-e57232-s006.zip › Figure 1/1G/5top_Sur7GFP_Nce102HA_aGFP.jpg]

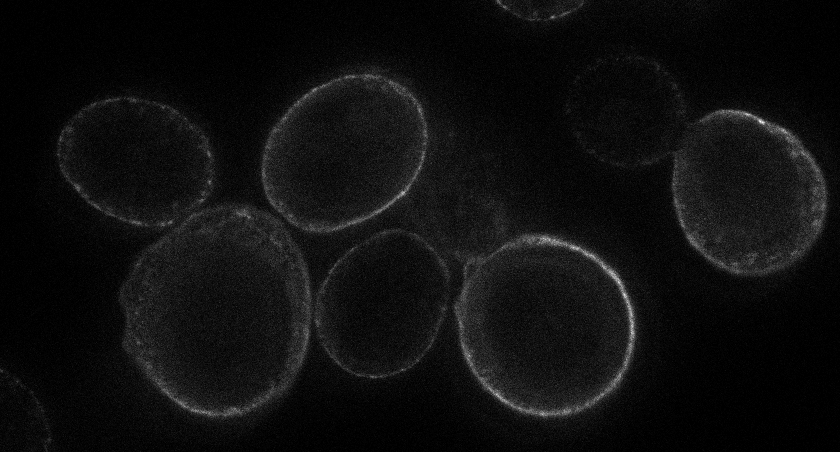

Supplement: Supplementary file 9 — Source Data for Figure 2 [file EMBR-24-e57232-s005.zip › Figure 2/2A/Mup1_medial.tif]

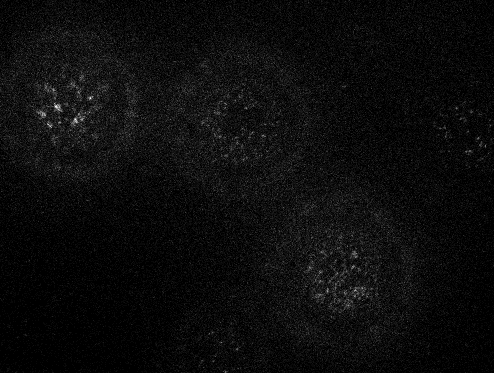

Supplement: Supplementary file 9 — Source Data for Figure 2 [file EMBR-24-e57232-s005.zip › Figure 2/2A/Mup1_top.tif]

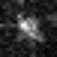

Supplement: Supplementary file 9 — Source Data for Figure 2 [file EMBR-24-e57232-s005.zip › Figure 2/2A/Mup1_top_detail_3x.tif]

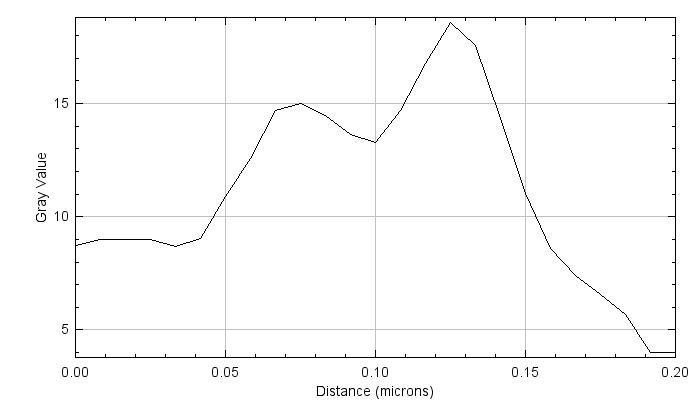

Supplement: Supplementary file 9 — Source Data for Figure 2 [file EMBR-24-e57232-s005.zip › Figure 2/2A/Mup1_top_profile.tif]
