# Supplementary figures and images for: Tetraspanner‐based nanodomains modulate BAR domain‐induced membrane curvature (part 2 of 3)
Source: EMBO Rep. 2023 Oct 30;24(12):e57232. doi: 10.15252/embr.202357232 (PMC10702824; doi:10.15252/embr.202357232)

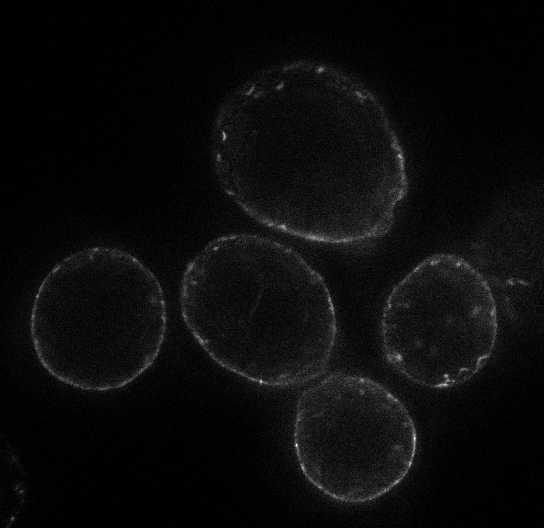

Supplement: Supplementary file 9 — Source Data for Figure 2 [file EMBR-24-e57232-s005.zip › Figure 2/2A/Nce102_medial.tif]

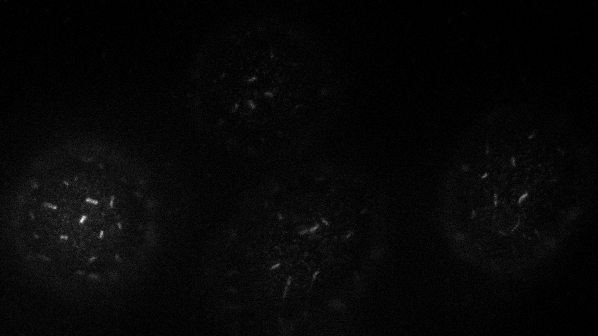

Supplement: Supplementary file 9 — Source Data for Figure 2 [file EMBR-24-e57232-s005.zip › Figure 2/2A/Nce102_top.tif]

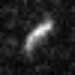

Supplement: Supplementary file 9 — Source Data for Figure 2 [file EMBR-24-e57232-s005.zip › Figure 2/2A/Nce102_top_detail_3x.tif]

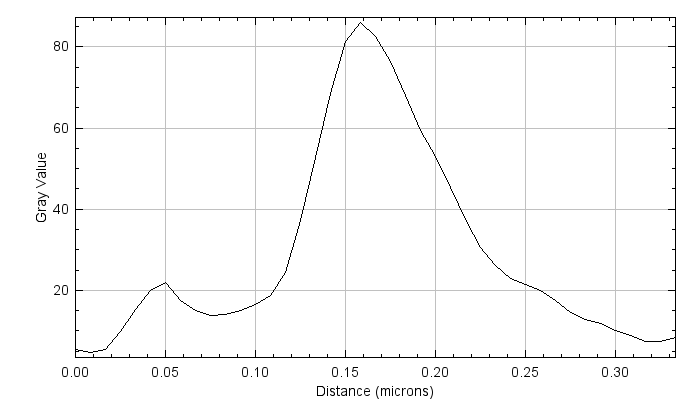

Supplement: Supplementary file 9 — Source Data for Figure 2 [file EMBR-24-e57232-s005.zip › Figure 2/2A/Nce102_top_profile.tif]

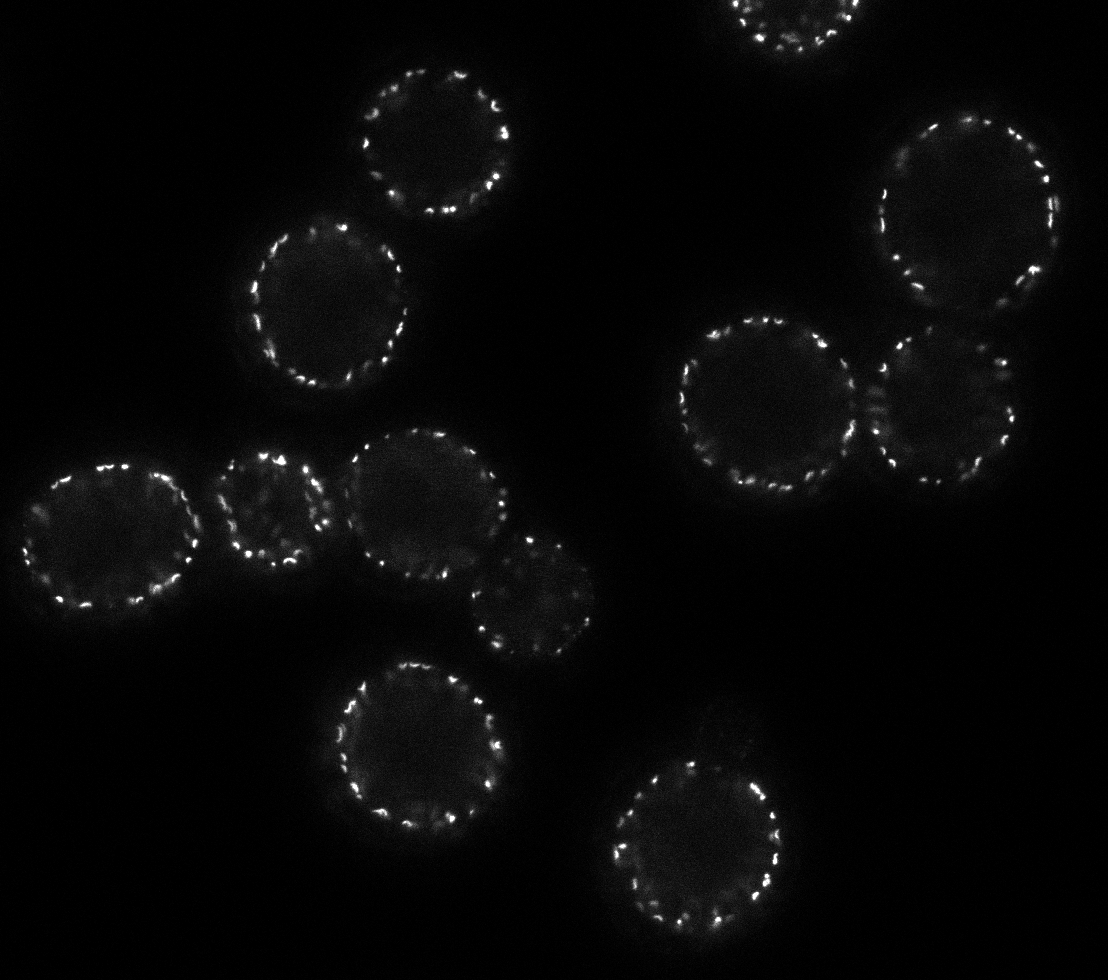

Supplement: Supplementary file 9 — Source Data for Figure 2 [file EMBR-24-e57232-s005.zip › Figure 2/2A/Pil1_medial.tif]

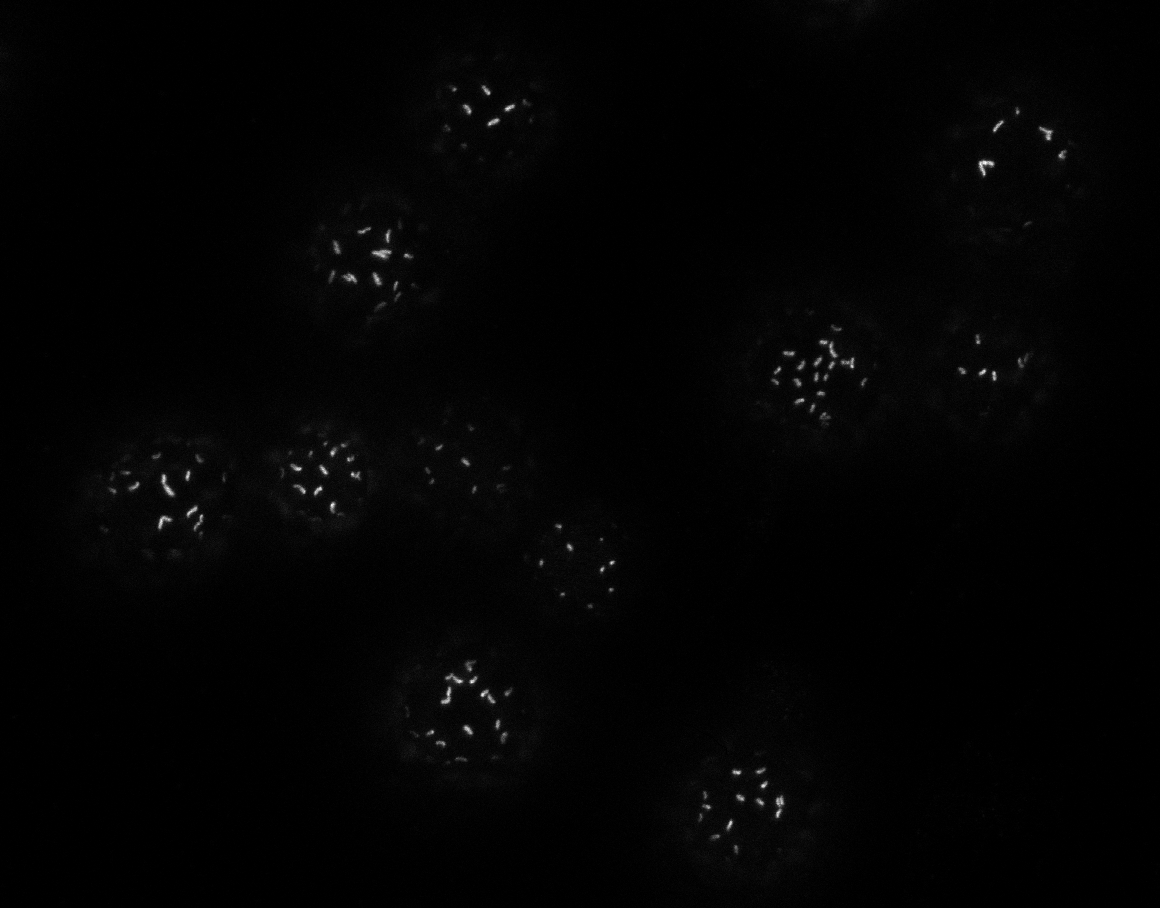

Supplement: Supplementary file 9 — Source Data for Figure 2 [file EMBR-24-e57232-s005.zip › Figure 2/2A/Pil1_top.tif]

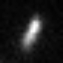

Supplement: Supplementary file 9 — Source Data for Figure 2 [file EMBR-24-e57232-s005.zip › Figure 2/2A/Pil1_top_detail_3x.tif]

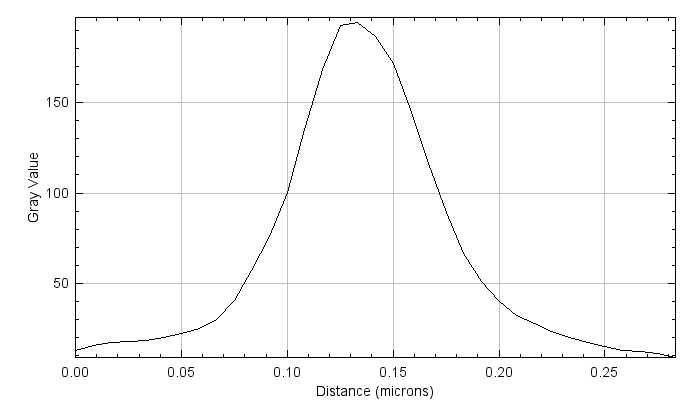

Supplement: Supplementary file 9 — Source Data for Figure 2 [file EMBR-24-e57232-s005.zip › Figure 2/2A/Pil1_top_profile.tif]

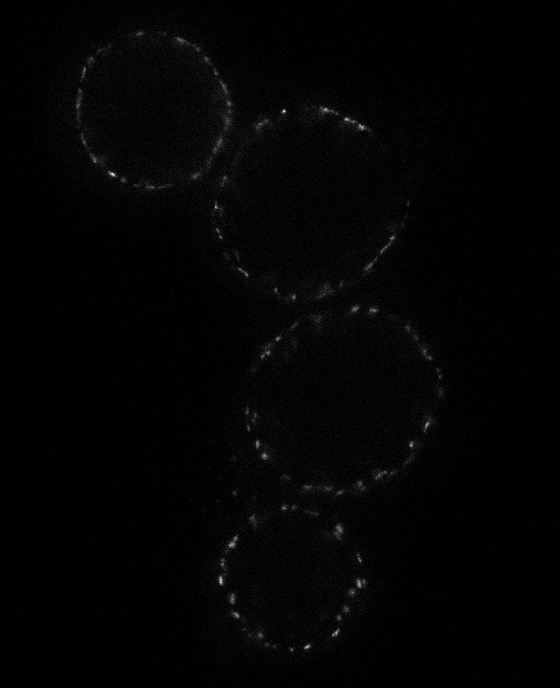

Supplement: Supplementary file 9 — Source Data for Figure 2 [file EMBR-24-e57232-s005.zip › Figure 2/2A/Sur7_medial.tif]

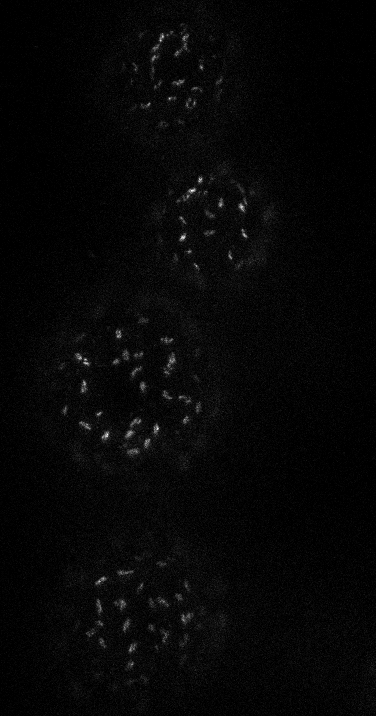

Supplement: Supplementary file 9 — Source Data for Figure 2 [file EMBR-24-e57232-s005.zip › Figure 2/2A/Sur7_top.tif]

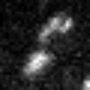

Supplement: Supplementary file 9 — Source Data for Figure 2 [file EMBR-24-e57232-s005.zip › Figure 2/2A/Sur7_top_detail_3x.tif]

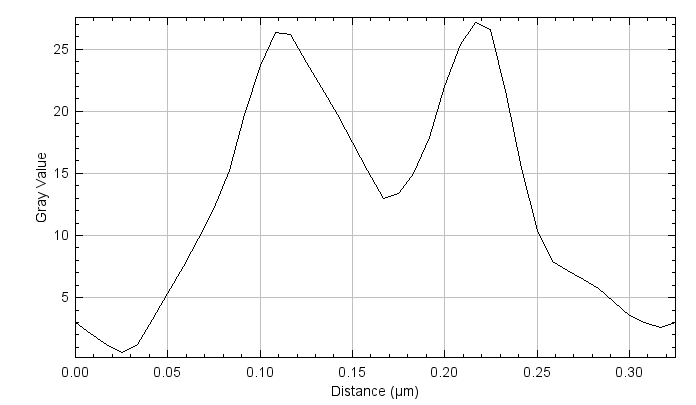

Supplement: Supplementary file 9 — Source Data for Figure 2 [file EMBR-24-e57232-s005.zip › Figure 2/2A/Sur7_top_profile.tif]

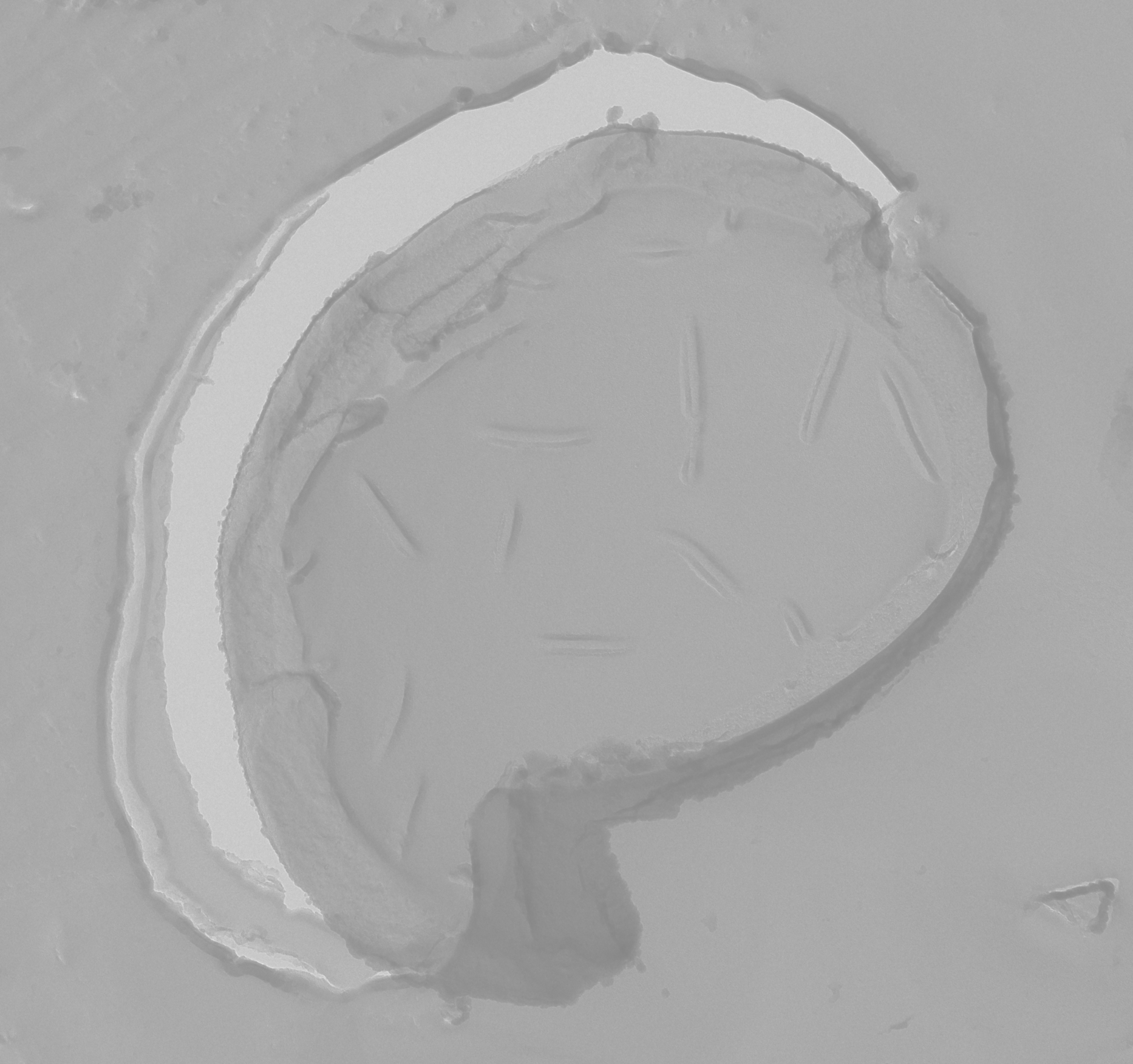

Supplement: Supplementary file 9 — Source Data for Figure 2 [file EMBR-24-e57232-s005.zip › Figure 2/2B/FreezeFracture.tif]

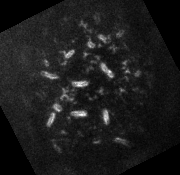

Supplement: Supplementary file 9 — Source Data for Figure 2 [file EMBR-24-e57232-s005.zip › Figure 2/2B/STED.tif]

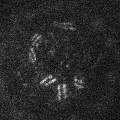

Supplement: Supplementary file 9 — Source Data for Figure 2 [file EMBR-24-e57232-s005.zip › Figure 2/2C/Dnce102_STED.tif]

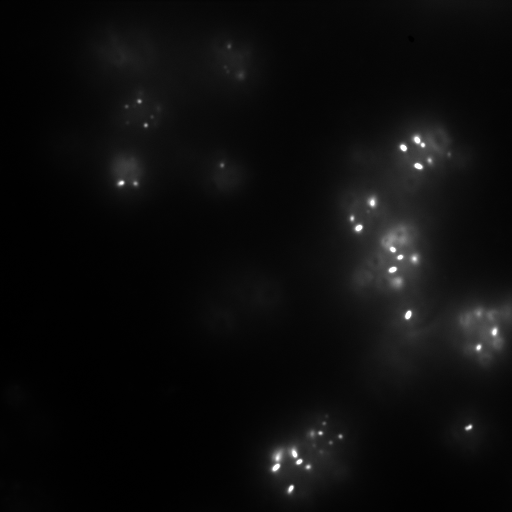

Supplement: Supplementary file 9 — Source Data for Figure 2 [file EMBR-24-e57232-s005.zip › Figure 2/2C/Dnce102_TIRFM.tif]

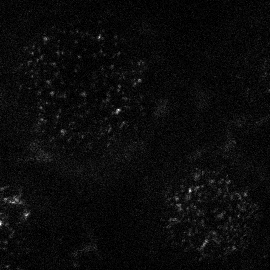

Supplement: Supplementary file 9 — Source Data for Figure 2 [file EMBR-24-e57232-s005.zip › Figure 2/2C/Dpil1_STED.tif]

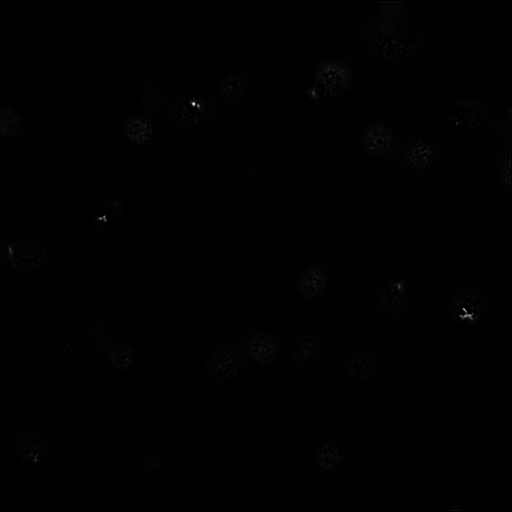

Supplement: Supplementary file 9 — Source Data for Figure 2 [file EMBR-24-e57232-s005.zip › Figure 2/2C/Dpil1_TIRFM.tif]

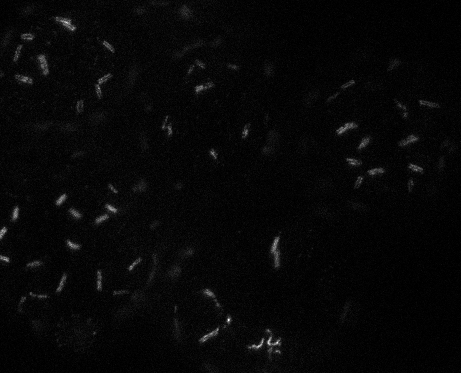

Supplement: Supplementary file 9 — Source Data for Figure 2 [file EMBR-24-e57232-s005.zip › Figure 2/2C/WT_STED.tif]

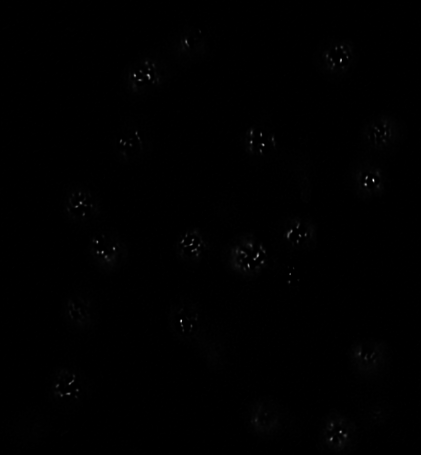

Supplement: Supplementary file 9 — Source Data for Figure 2 [file EMBR-24-e57232-s005.zip › Figure 2/2C/WT_TIRFM.tif]

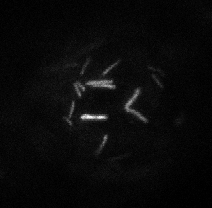

Supplement: Supplementary file 9 — Source Data for Figure 2 [file EMBR-24-e57232-s005.zip › Figure 2/2D/Seg1OE_Pil1.tif]

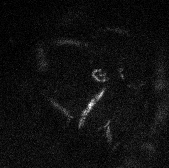

Supplement: Supplementary file 9 — Source Data for Figure 2 [file EMBR-24-e57232-s005.zip › Figure 2/2D/Seg1OE_Sur7.tif]

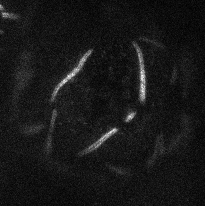

Supplement: Supplementary file 9 — Source Data for Figure 2 [file EMBR-24-e57232-s005.zip › Figure 2/2D/Seg1OE_Sur7OE.tif]

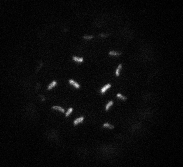

Supplement: Supplementary file 9 — Source Data for Figure 2 [file EMBR-24-e57232-s005.zip › Figure 2/2D/WT_Pil1.tif]

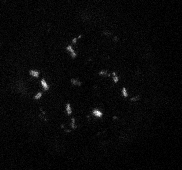

Supplement: Supplementary file 9 — Source Data for Figure 2 [file EMBR-24-e57232-s005.zip › Figure 2/2D/WT_Sur7.tif]

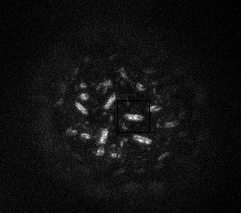

Supplement: Supplementary file 9 — Source Data for Figure 2 [file EMBR-24-e57232-s005.zip › Figure 2/2D/WT_Sur7OE.tif]

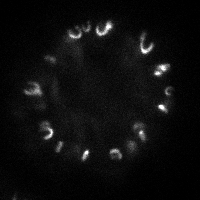

Supplement: Supplementary file 10 — Source Data for Figure 3 [file EMBR-24-e57232-s001.zip › Figure 3/3A/5xKO_Pil1Halo_medial.tif]

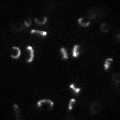

Supplement: Supplementary file 10 — Source Data for Figure 3 [file EMBR-24-e57232-s001.zip › Figure 3/3A/5xKO_Pil1Halo_top.tif]

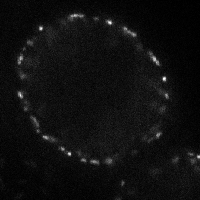

Supplement: Supplementary file 10 — Source Data for Figure 3 [file EMBR-24-e57232-s001.zip › Figure 3/3A/WT_Pil1Halo_medial.tif]

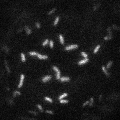

Supplement: Supplementary file 10 — Source Data for Figure 3 [file EMBR-24-e57232-s001.zip › Figure 3/3A/WT_Pil1Halo_top.tif]

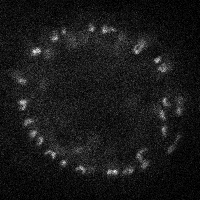

Supplement: Supplementary file 10 — Source Data for Figure 3 [file EMBR-24-e57232-s001.zip › Figure 3/3D/5xKO_Lsp1Halo_medial.tif]

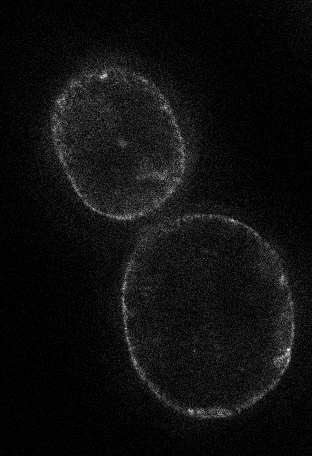

Supplement: Supplementary file 10 — Source Data for Figure 3 [file EMBR-24-e57232-s001.zip › Figure 3/3D/5xKO_Mup1DCHalo_medial.tif]

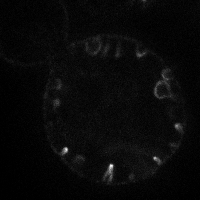

Supplement: Supplementary file 10 — Source Data for Figure 3 [file EMBR-24-e57232-s001.zip › Figure 3/3D/5xKO_Nce102Halo_medial.tif]

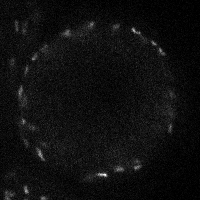

Supplement: Supplementary file 10 — Source Data for Figure 3 [file EMBR-24-e57232-s001.zip › Figure 3/3D/WT_Lsp1Halo_medial.tif]

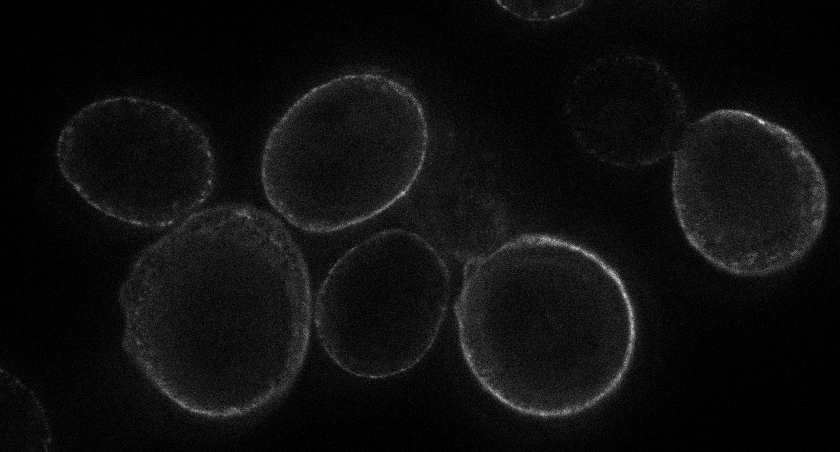

Supplement: Supplementary file 10 — Source Data for Figure 3 [file EMBR-24-e57232-s001.zip › Figure 3/3D/WT_Mup1DCHalo_medial.tif]

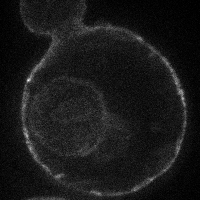

Supplement: Supplementary file 10 — Source Data for Figure 3 [file EMBR-24-e57232-s001.zip › Figure 3/3D/WT_Nce102Halo_medial.tif]

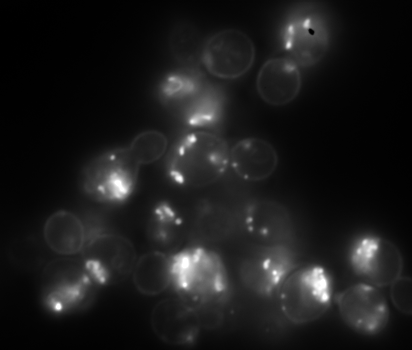

Supplement: Supplementary file 10 — Source Data for Figure 3 [file EMBR-24-e57232-s001.zip › Figure 3/3E/5xKO_Nce102mNeGr_Pil1mRFPruby_medial.tif]

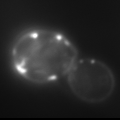

Supplement: Supplementary file 10 — Source Data for Figure 3 [file EMBR-24-e57232-s001.zip › Figure 3/3E/WT_Nce102mNeGr_Pil1mRFPruby_medial.tif]

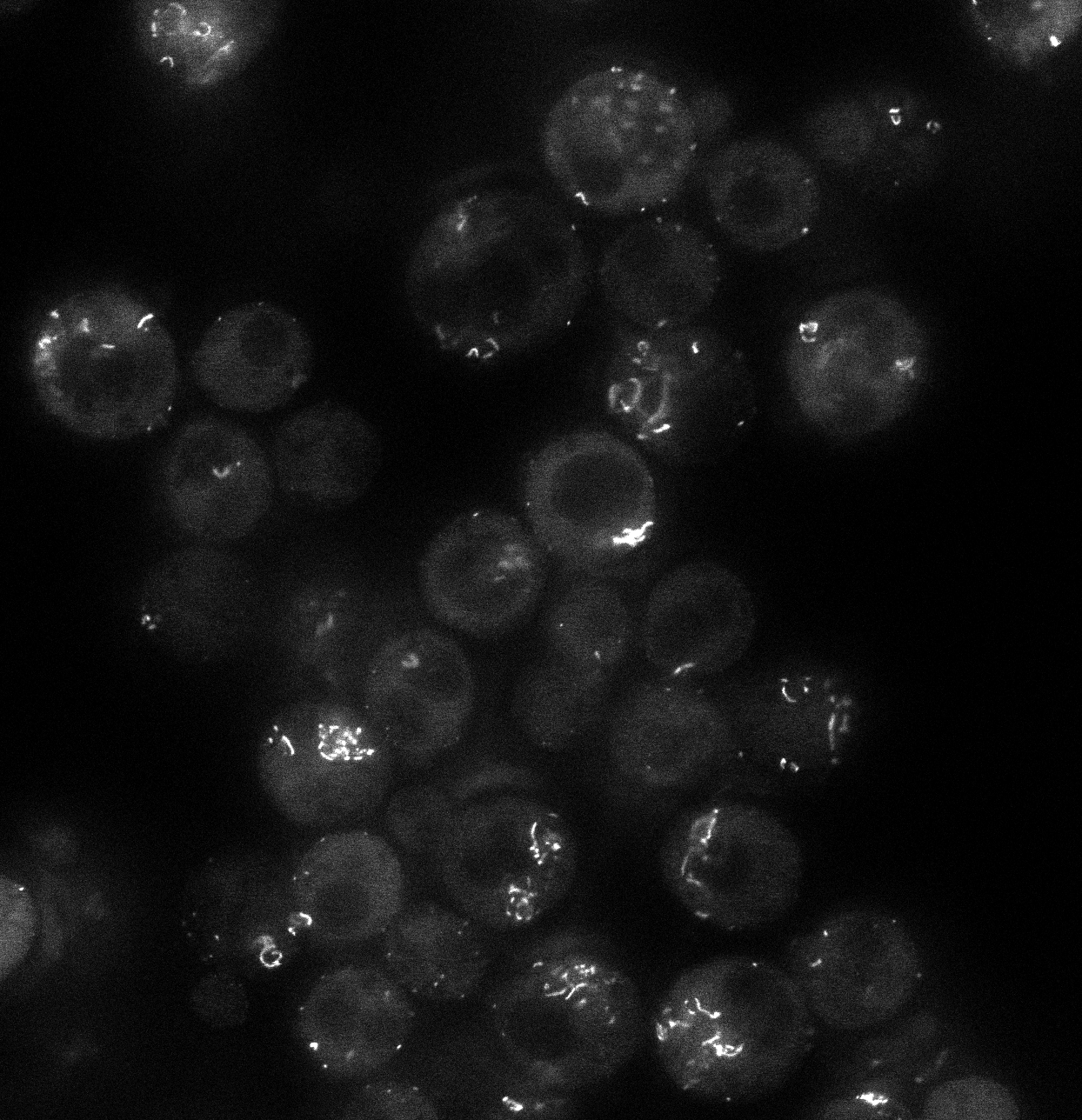

Supplement: Supplementary file 10 — Source Data for Figure 3 [file EMBR-24-e57232-s001.zip › Figure 3/3F/5xKO_dNce102_Pil1Halo_medial.tif]

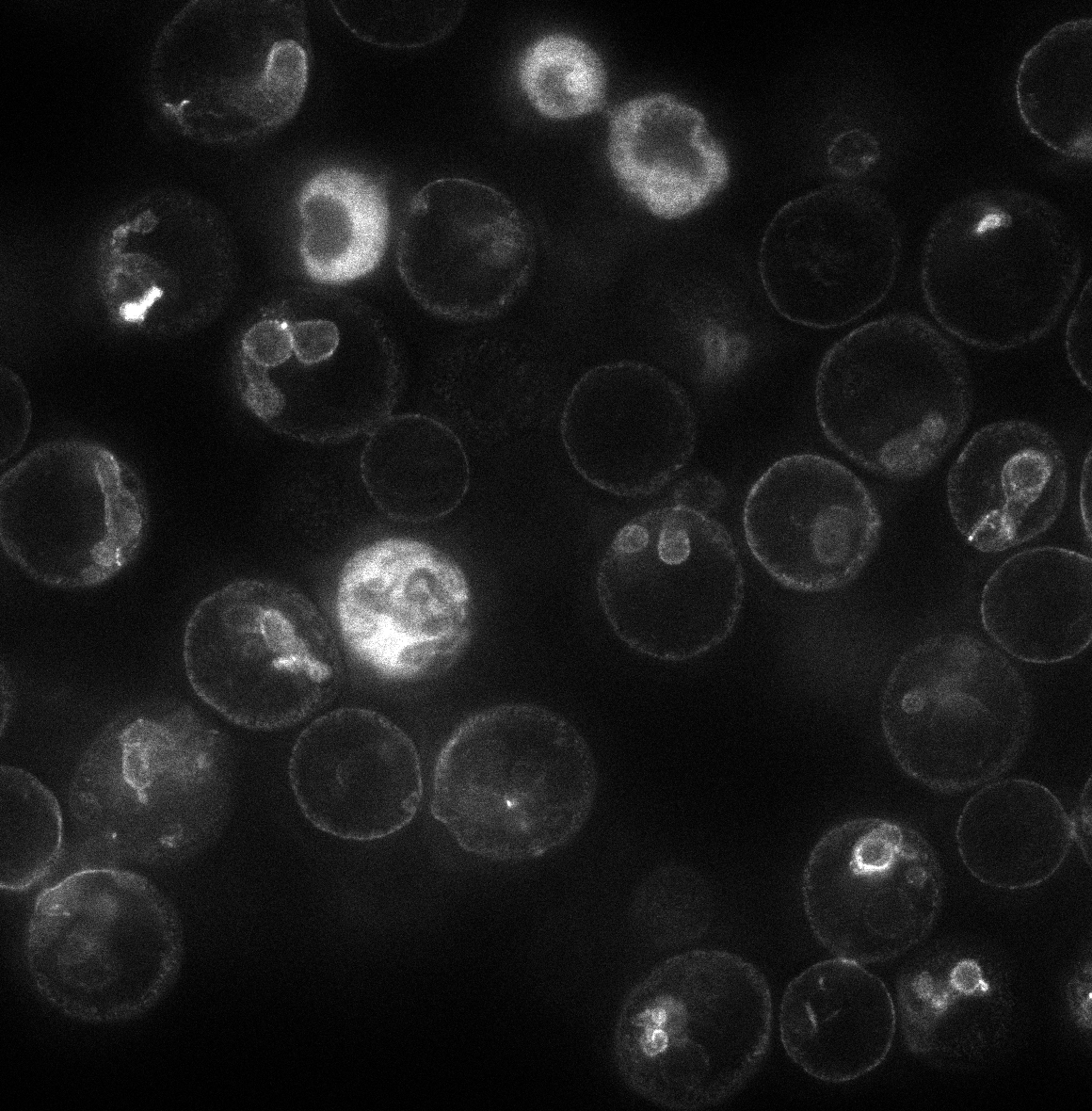

Supplement: Supplementary file 10 — Source Data for Figure 3 [file EMBR-24-e57232-s001.zip › Figure 3/3F/5xKO_dPil1_Nce102Halo_medial.tif]

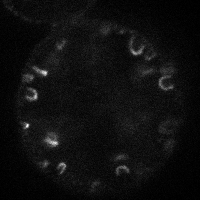

Supplement: Supplementary file 10 — Source Data for Figure 3 [file EMBR-24-e57232-s001.zip › Figure 3/3F/5xKO_Nce102Halo_medial.tif]

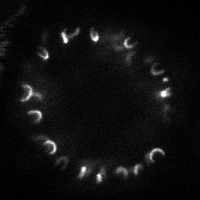

Supplement: Supplementary file 10 — Source Data for Figure 3 [file EMBR-24-e57232-s001.zip › Figure 3/3F/5xKO_Pil1Halo_medial.tif]

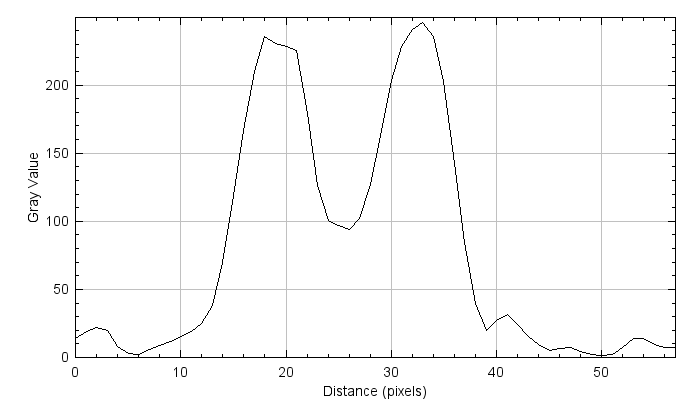

Supplement: Supplementary file 10 — Source Data for Figure 3 [file EMBR-24-e57232-s001.zip › Figure 3/3G/5xKO_Pil1Halo_profile.tif]

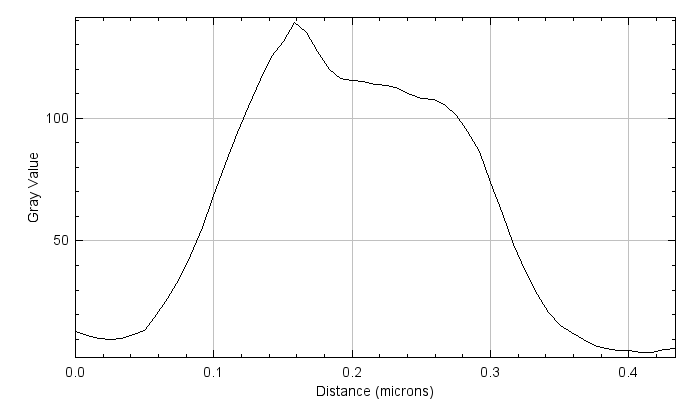

Supplement: Supplementary file 10 — Source Data for Figure 3 [file EMBR-24-e57232-s001.zip › Figure 3/3G/WT_Pil1Halo_profile.tif]

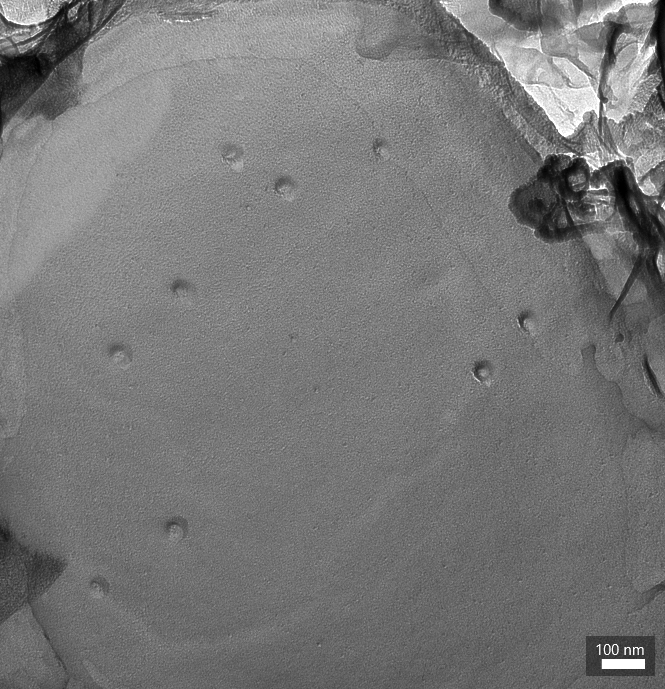

Supplement: Supplementary file 10 — Source Data for Figure 3 [file EMBR-24-e57232-s001.zip › Figure 3/3H/5xKO_FreezeFracture1.tif]

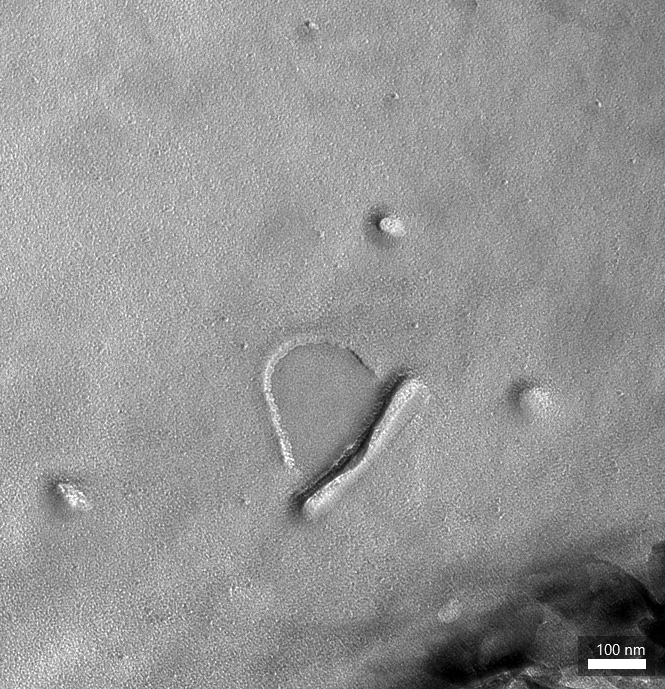

Supplement: Supplementary file 10 — Source Data for Figure 3 [file EMBR-24-e57232-s001.zip › Figure 3/3H/5xKO_FreezeFracture2.tif]

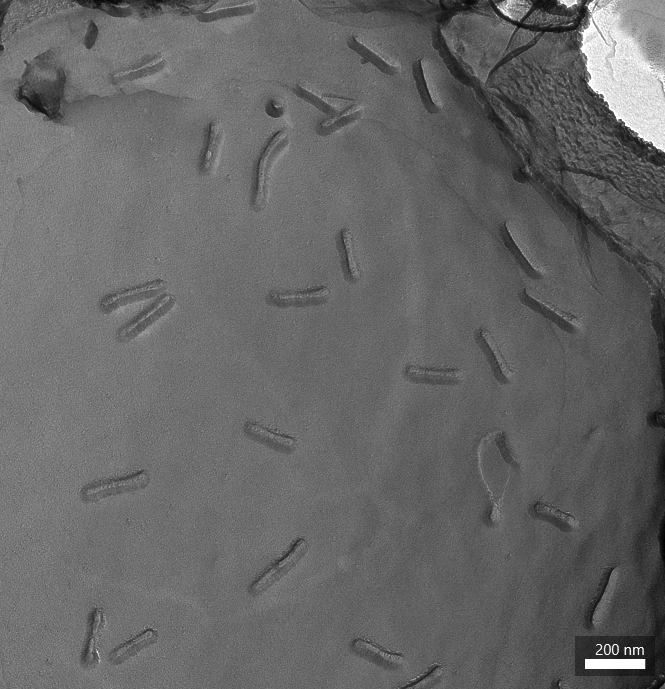

Supplement: Supplementary file 10 — Source Data for Figure 3 [file EMBR-24-e57232-s001.zip › Figure 3/3H/WT_FreezeFracture.tif]

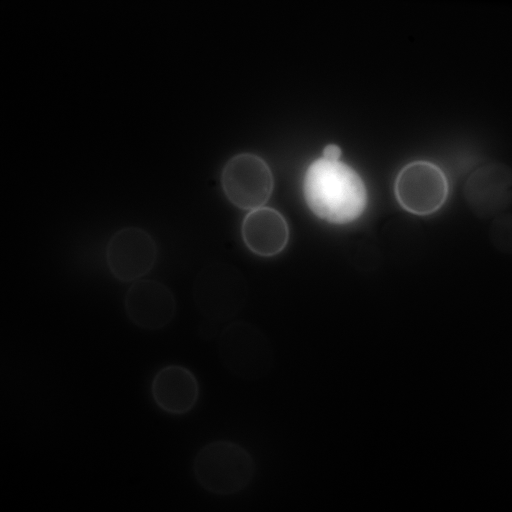

Supplement: Supplementary file 11 — Source Data for Figure 4 [file EMBR-24-e57232-s002.zip › Figure 4/4A/5xKO_2xPH_cell.tif]

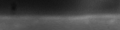

Supplement: Supplementary file 11 — Source Data for Figure 4 [file EMBR-24-e57232-s002.zip › Figure 4/4A/5xKO_2xPH_linear.tif]

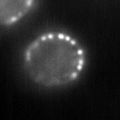

Supplement: Supplementary file 11 — Source Data for Figure 4 [file EMBR-24-e57232-s002.zip › Figure 4/4A/5xKO_A08184g_cell.tif]

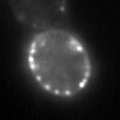

Supplement: Supplementary file 11 — Source Data for Figure 4 [file EMBR-24-e57232-s002.zip › Figure 4/4A/5xKO_Fmp45_cell.tif]

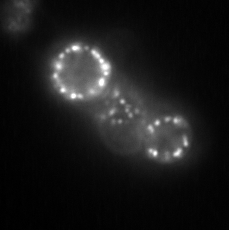

Supplement: Supplementary file 11 — Source Data for Figure 4 [file EMBR-24-e57232-s002.zip › Figure 4/4A/5xKO_Pun1_cell.tif]

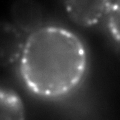

Supplement: Supplementary file 11 — Source Data for Figure 4 [file EMBR-24-e57232-s002.zip › Figure 4/4A/5xKO_Sur7_cell.tif]

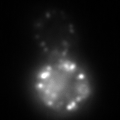

Supplement: Supplementary file 11 — Source Data for Figure 4 [file EMBR-24-e57232-s002.zip › Figure 4/4A/5xKO_Tos7_cell.tif]

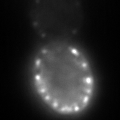

Supplement: Supplementary file 11 — Source Data for Figure 4 [file EMBR-24-e57232-s002.zip › Figure 4/4A/5xKO_Ynl194c_cell.tif]

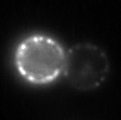

Supplement: Supplementary file 11 — Source Data for Figure 4 [file EMBR-24-e57232-s002.zip › Figure 4/4A/WT_Sur7_cell.tif]

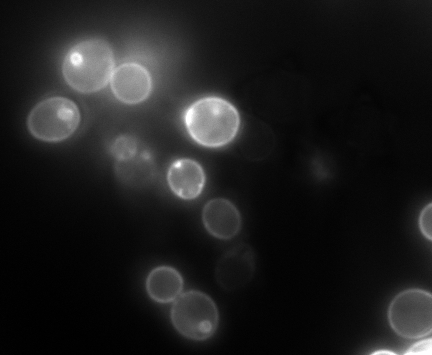

Supplement: Supplementary file 11 — Source Data for Figure 4 [file EMBR-24-e57232-s002.zip › Figure 4/4C/5xKO_2PH_cell.tif]

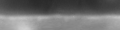

Supplement: Supplementary file 11 — Source Data for Figure 4 [file EMBR-24-e57232-s002.zip › Figure 4/4C/5xKO_2PH_linear.tif]

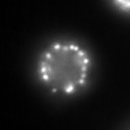

Supplement: Supplementary file 11 — Source Data for Figure 4 [file EMBR-24-e57232-s002.zip › Figure 4/4C/5xKO_Sur7DC_cell.tif]

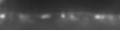

Supplement: Supplementary file 11 — Source Data for Figure 4 [file EMBR-24-e57232-s002.zip › Figure 4/4C/5xKO_Sur7DC_linear.tif]

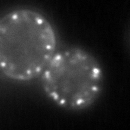

Supplement: Supplementary file 11 — Source Data for Figure 4 [file EMBR-24-e57232-s002.zip › Figure 4/4C/5xKO_Sur7DNDC_cell.tif]

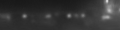

Supplement: Supplementary file 11 — Source Data for Figure 4 [file EMBR-24-e57232-s002.zip › Figure 4/4C/5xKO_Sur7DNDC_linear.tif]

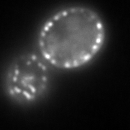

Supplement: Supplementary file 11 — Source Data for Figure 4 [file EMBR-24-e57232-s002.zip › Figure 4/4C/5xKO_Sur7DN_cell.tif]

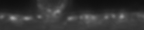

Supplement: Supplementary file 11 — Source Data for Figure 4 [file EMBR-24-e57232-s002.zip › Figure 4/4C/5xKO_Sur7DN_linear.tif]

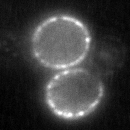

Supplement: Supplementary file 11 — Source Data for Figure 4 [file EMBR-24-e57232-s002.zip › Figure 4/4C/5xKO_Sur7_cell.tif]

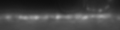

Supplement: Supplementary file 11 — Source Data for Figure 4 [file EMBR-24-e57232-s002.zip › Figure 4/4C/5xKO_Sur7_linear.tif]

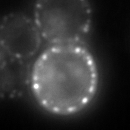

Supplement: Supplementary file 11 — Source Data for Figure 4 [file EMBR-24-e57232-s002.zip › Figure 4/4C/WT_Sur7_cell.tif]

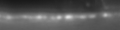

Supplement: Supplementary file 11 — Source Data for Figure 4 [file EMBR-24-e57232-s002.zip › Figure 4/4C/WT_Sur7_linear.tif]

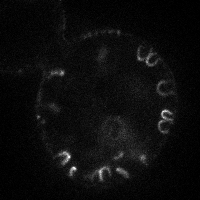

Supplement: Supplementary file 11 — Source Data for Figure 4 [file EMBR-24-e57232-s002.zip › Figure 4/4D/5xKO_cell.tif]

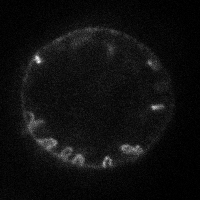

Supplement: Supplementary file 11 — Source Data for Figure 4 [file EMBR-24-e57232-s002.zip › Figure 4/4D/5xKO_empty_cell.tif]

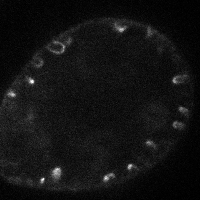

Supplement: Supplementary file 11 — Source Data for Figure 4 [file EMBR-24-e57232-s002.zip › Figure 4/4D/5xKO_Sur7DC_cell.tif]

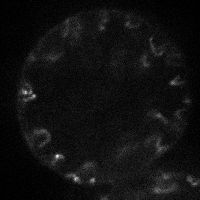

Supplement: Supplementary file 11 — Source Data for Figure 4 [file EMBR-24-e57232-s002.zip › Figure 4/4D/5xKO_Sur7DNDC_cell.tif]

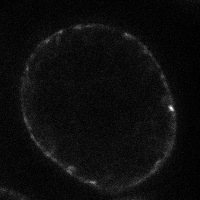

Supplement: Supplementary file 11 — Source Data for Figure 4 [file EMBR-24-e57232-s002.zip › Figure 4/4D/5xKO_Sur7DN_cell.tif]

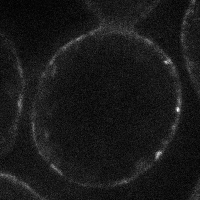

Supplement: Supplementary file 11 — Source Data for Figure 4 [file EMBR-24-e57232-s002.zip › Figure 4/4D/5xKO_Sur7_cell.tif]

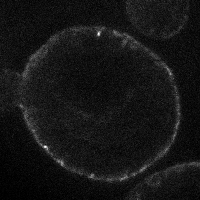

Supplement: Supplementary file 11 — Source Data for Figure 4 [file EMBR-24-e57232-s002.zip › Figure 4/4D/WT_cell.tif]

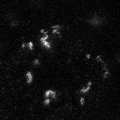

Supplement: Supplementary file 11 — Source Data for Figure 4 [file EMBR-24-e57232-s002.zip › Figure 4/4E/5xKO_Sur7DC_top.tif]

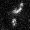

Supplement: Supplementary file 11 — Source Data for Figure 4 [file EMBR-24-e57232-s002.zip › Figure 4/4E/5xKO_Sur7DC_zoom.tif]

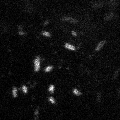

Supplement: Supplementary file 11 — Source Data for Figure 4 [file EMBR-24-e57232-s002.zip › Figure 4/4E/WT_Sur7DC_top.tif]

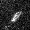

Supplement: Supplementary file 11 — Source Data for Figure 4 [file EMBR-24-e57232-s002.zip › Figure 4/4E/WT_Sur7DC_zoom.tif]

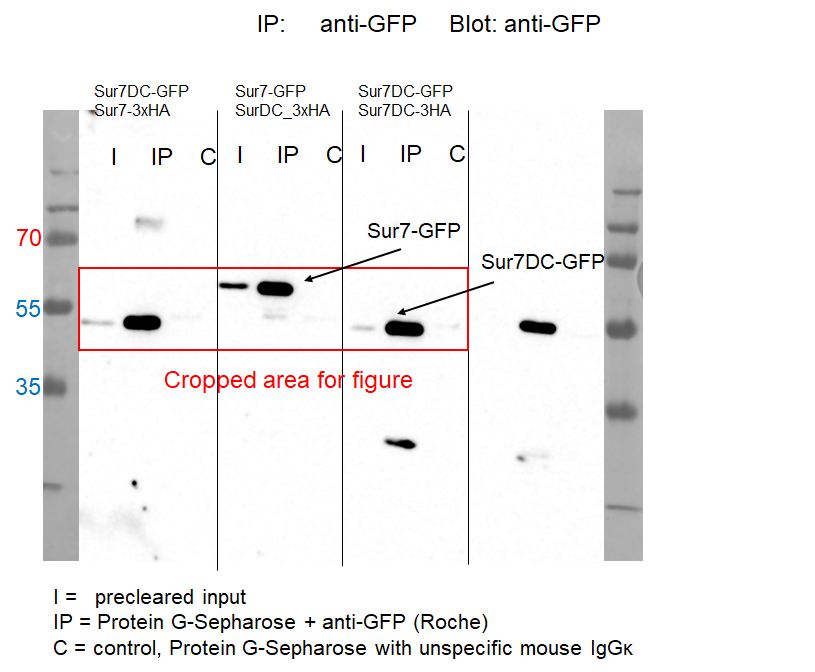

Supplement: Supplementary file 11 — Source Data for Figure 4 [file EMBR-24-e57232-s002.zip › Figure 4/4F/antiGFP.jpg]

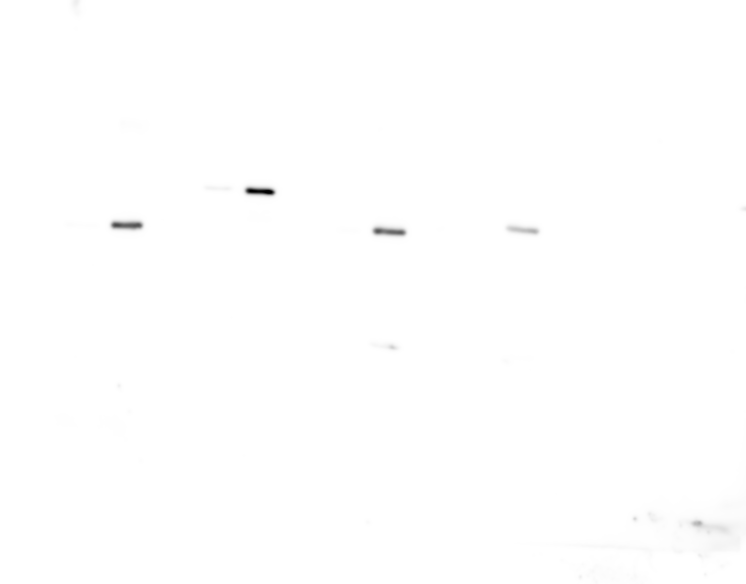

Supplement: Supplementary file 11 — Source Data for Figure 4 [file EMBR-24-e57232-s002.zip › Figure 4/4F/antiGFP.tif]

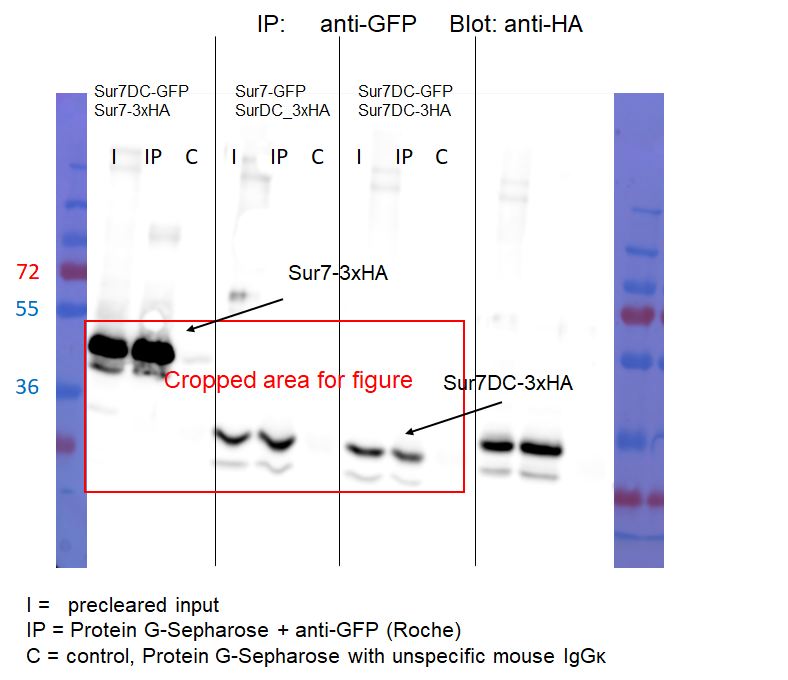

Supplement: Supplementary file 11 — Source Data for Figure 4 [file EMBR-24-e57232-s002.zip › Figure 4/4F/antiHA.jpg]

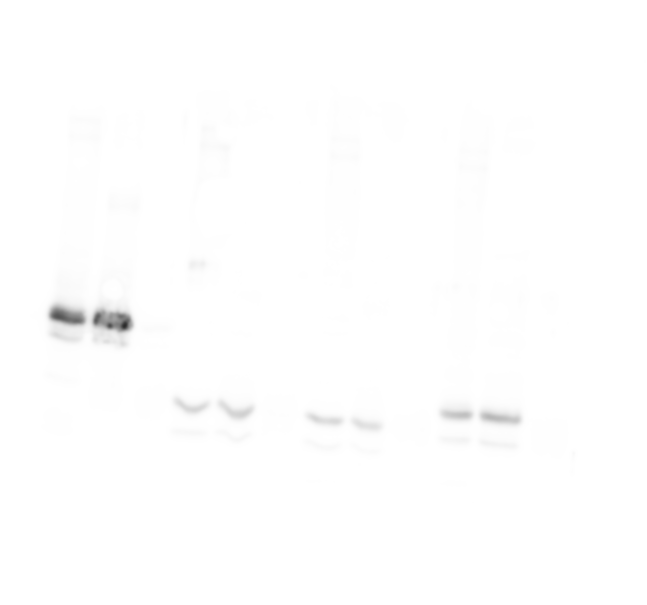

Supplement: Supplementary file 11 — Source Data for Figure 4 [file EMBR-24-e57232-s002.zip › Figure 4/4F/antiHA.tif]

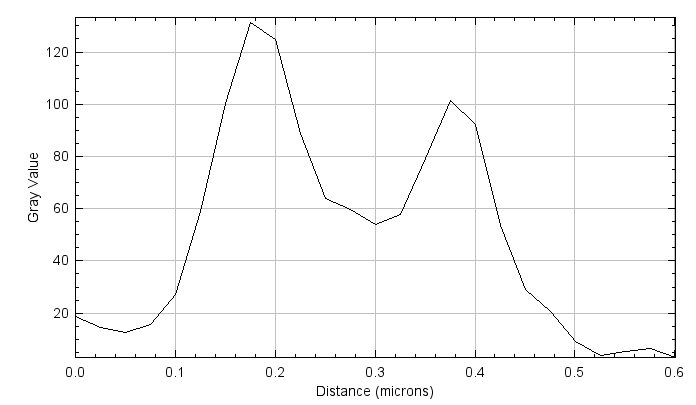

Supplement: Supplementary file 12 — Source Data for Figure 5 [file EMBR-24-e57232-s012.zip › Figure 5/5B/Pil1Halo_Dinp5152_profile.tif]

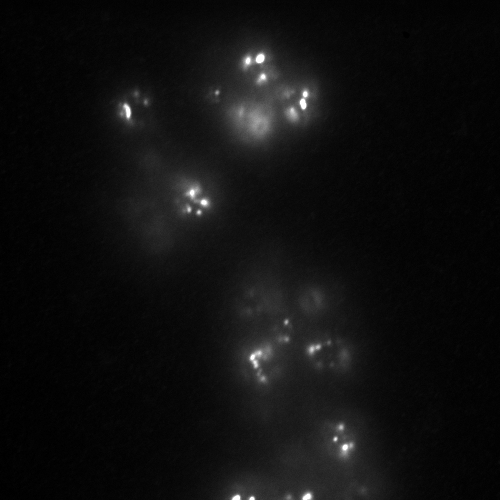

Supplement: Supplementary file 12 — Source Data for Figure 5 [file EMBR-24-e57232-s012.zip › Figure 5/5D/Dinp5152_Sur7mNeGr_Nce102RFP.tif]

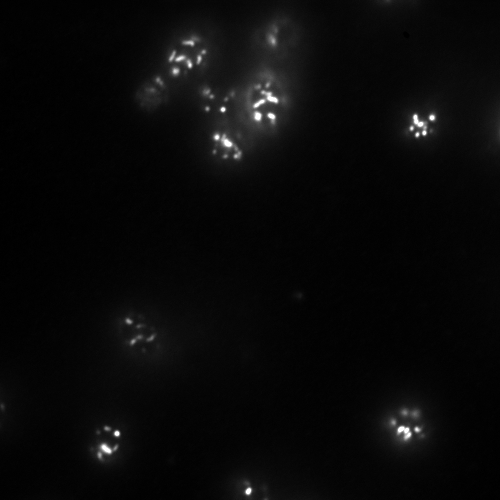

Supplement: Supplementary file 12 — Source Data for Figure 5 [file EMBR-24-e57232-s012.zip › Figure 5/5D/WT_Sur7mNeGr_Nce102RFP.tif]

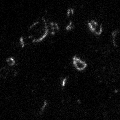

Supplement: Supplementary file 12 — Source Data for Figure 5 [file EMBR-24-e57232-s012.zip › Figure 5/5E/Dinp5152_Sur7.tif]

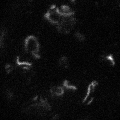

Supplement: Supplementary file 12 — Source Data for Figure 5 [file EMBR-24-e57232-s012.zip › Figure 5/5E/Dinp5152_Sur7OE.tif]

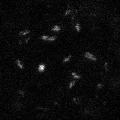

Supplement: Supplementary file 12 — Source Data for Figure 5 [file EMBR-24-e57232-s012.zip › Figure 5/5E/WT_Sur7.tif]

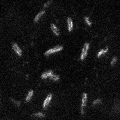

Supplement: Supplementary file 12 — Source Data for Figure 5 [file EMBR-24-e57232-s012.zip › Figure 5/5E/WT_Sur7OE.tif]

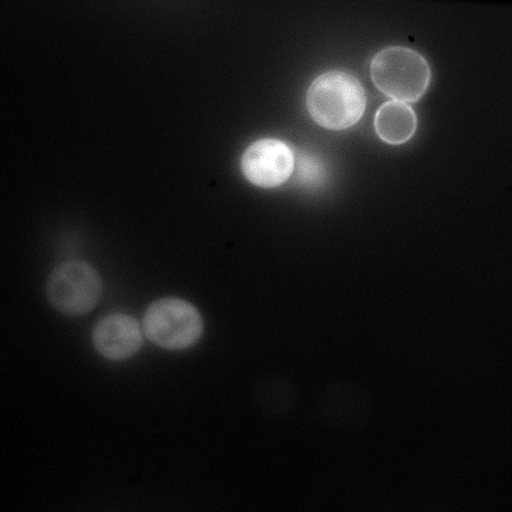

Supplement: Supplementary file 12 — Source Data for Figure 5 [file EMBR-24-e57232-s012.zip › Figure 5/5F/5xKO_PH.tif]

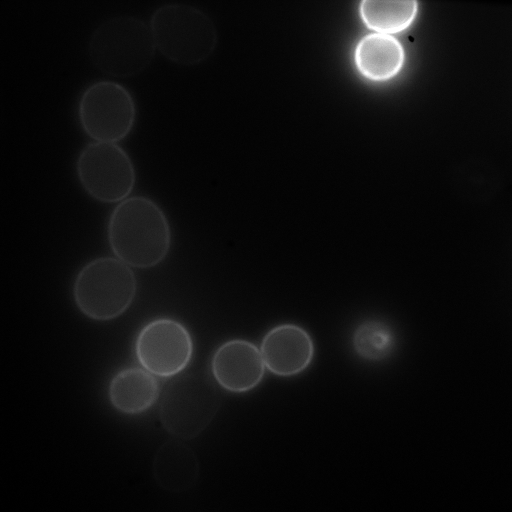

Supplement: Supplementary file 12 — Source Data for Figure 5 [file EMBR-24-e57232-s012.zip › Figure 5/5F/WT_PH.tif]

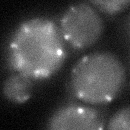

Supplement: Supplementary file 12 — Source Data for Figure 5 [file EMBR-24-e57232-s012.zip › Figure 5/5G/5xKO_Nce102RFP_Inp51GFP.tif]

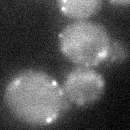

Supplement: Supplementary file 12 — Source Data for Figure 5 [file EMBR-24-e57232-s012.zip › Figure 5/5G/WT_Nce102RFP_Inp51GFP.tif]

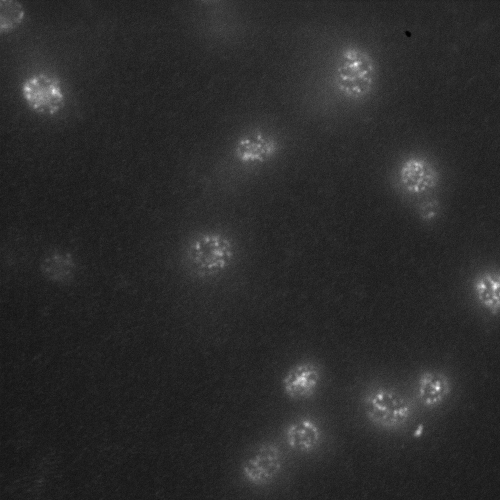

Supplement: Supplementary file 12 — Source Data for Figure 5 [file EMBR-24-e57232-s012.zip › Figure 5/5H/5xKO_GFP-Mss4_Nce102-RFP.tif]

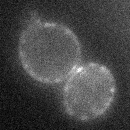

Supplement: Supplementary file 12 — Source Data for Figure 5 [file EMBR-24-e57232-s012.zip › Figure 5/5H/5xKO_Mss4GFP_medial.tif]

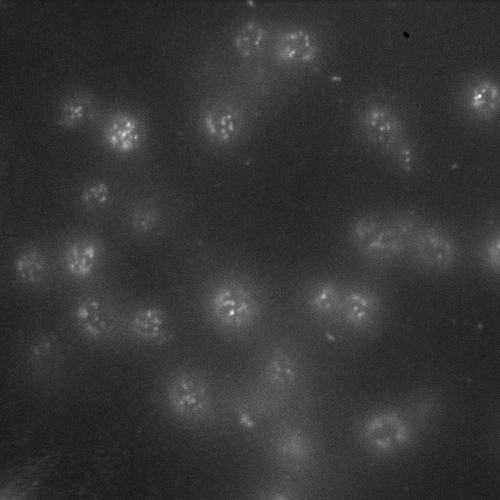

Supplement: Supplementary file 12 — Source Data for Figure 5 [file EMBR-24-e57232-s012.zip › Figure 5/5H/WT_GFP-Mss4_Nce102-RFP.tif]

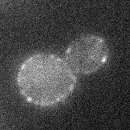

Supplement: Supplementary file 12 — Source Data for Figure 5 [file EMBR-24-e57232-s012.zip › Figure 5/5H/WT_Mss4_medial.tif]

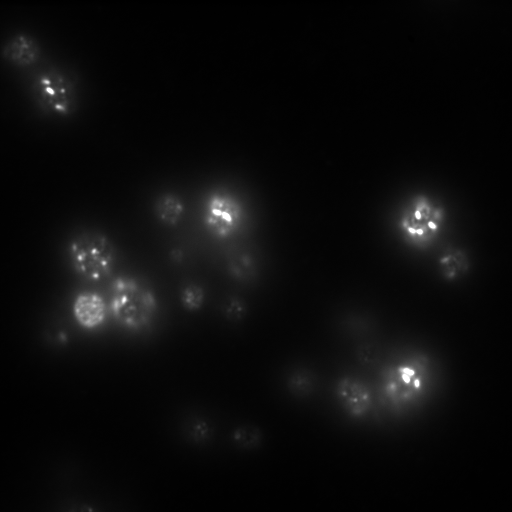

Supplement: Supplementary file 13 — Source Data for Figure 6 [file EMBR-24-e57232-s015.zip › Figure 6/6A/Dinp5152_Nce102-Sur7G_Pil1R_cell.tif]

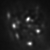

Supplement: Supplementary file 13 — Source Data for Figure 6 [file EMBR-24-e57232-s015.zip › Figure 6/6A/Dinp5152_Nce102-Sur7G_Pil1R_zoom.tif]
