# Supplementary figures and images for: Tetraspanner‐based nanodomains modulate BAR domain‐induced membrane curvature (part 3 of 3)
Source: EMBO Rep. 2023 Oct 30;24(12):e57232. doi: 10.15252/embr.202357232 (PMC10702824; doi:10.15252/embr.202357232)

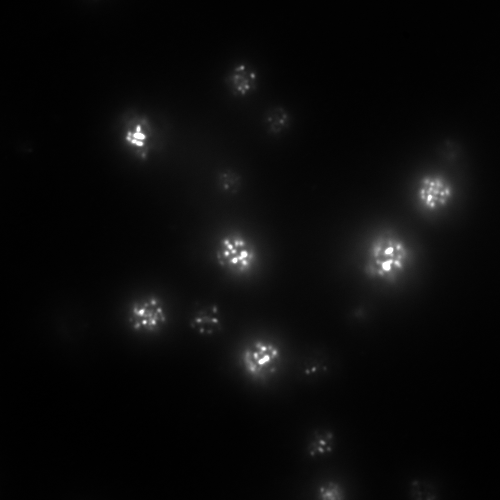

Supplement: Supplementary file 13 — Source Data for Figure 6 [file EMBR-24-e57232-s015.zip › Figure 6/6A/Dinp5152_Nce102-Sur7G_Sur7R_cell.tif]

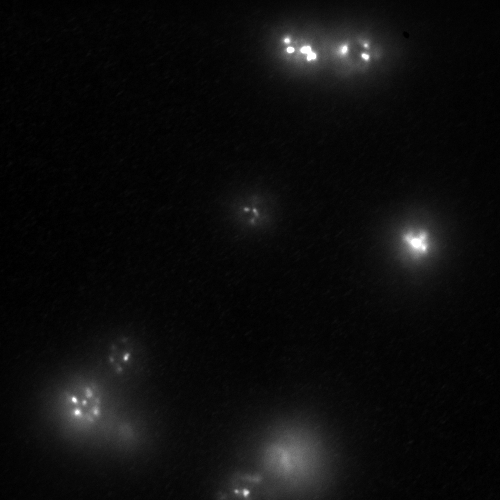

Supplement: Supplementary file 13 — Source Data for Figure 6 [file EMBR-24-e57232-s015.zip › Figure 6/6A/Dinp5152_Nce102G_Sur7R_cell.tif]

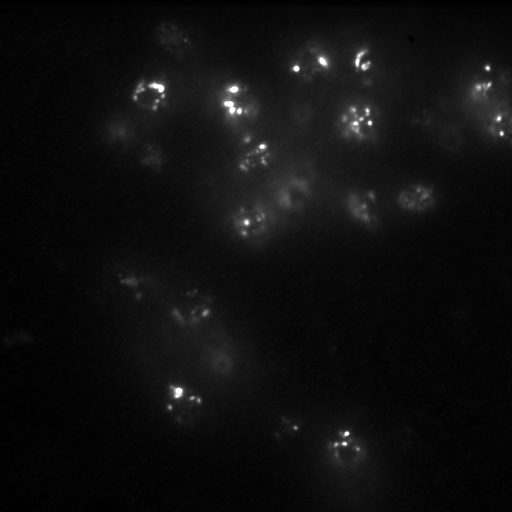

Supplement: Supplementary file 13 — Source Data for Figure 6 [file EMBR-24-e57232-s015.zip › Figure 6/6A/Dinp5152_Sur7G_Pil1R_cell.tif]

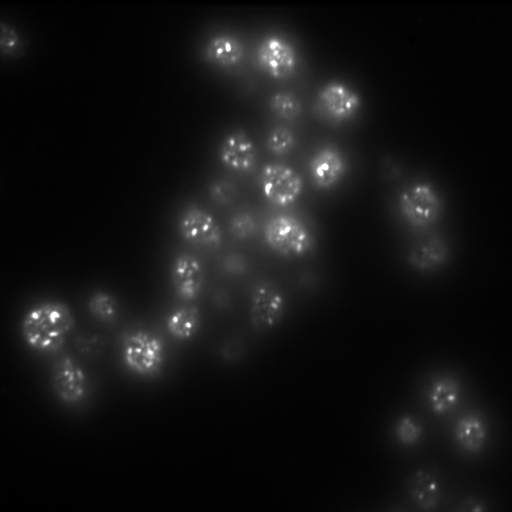

Supplement: Supplementary file 13 — Source Data for Figure 6 [file EMBR-24-e57232-s015.zip › Figure 6/6A/WT_Nce102-Sur7G_Pil1R_cell.tif]

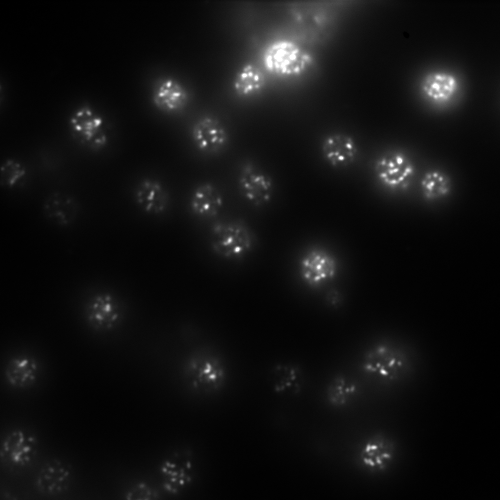

Supplement: Supplementary file 13 — Source Data for Figure 6 [file EMBR-24-e57232-s015.zip › Figure 6/6A/WT_Nce102-Sur7G_Sur7R_cell.tif]

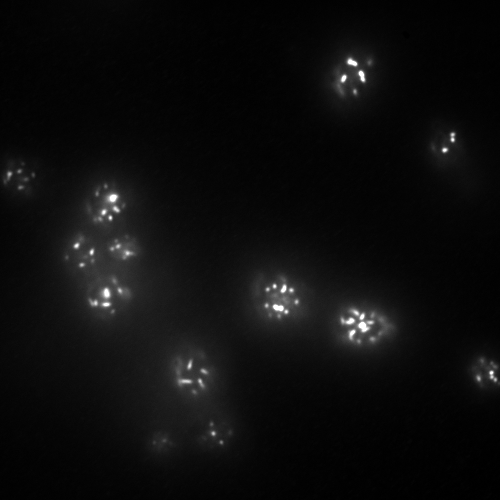

Supplement: Supplementary file 13 — Source Data for Figure 6 [file EMBR-24-e57232-s015.zip › Figure 6/6A/WT_Nce102G_Sur7R_cell.tif]

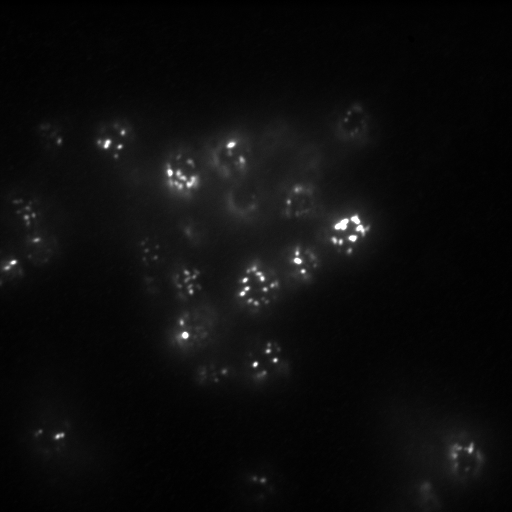

Supplement: Supplementary file 13 — Source Data for Figure 6 [file EMBR-24-e57232-s015.zip › Figure 6/6A/WT_Sur7G_Pil1R_cell.tif]

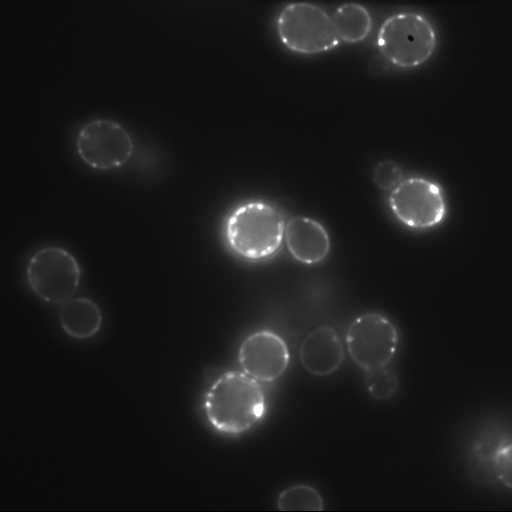

Supplement: Supplementary file 13 — Source Data for Figure 6 [file EMBR-24-e57232-s015.zip › Figure 6/6B/Dinp5152_Nce102-Sur7_cell.tif]

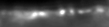

Supplement: Supplementary file 13 — Source Data for Figure 6 [file EMBR-24-e57232-s015.zip › Figure 6/6B/Dinp5152_Nce102-Sur7_linear.tif]

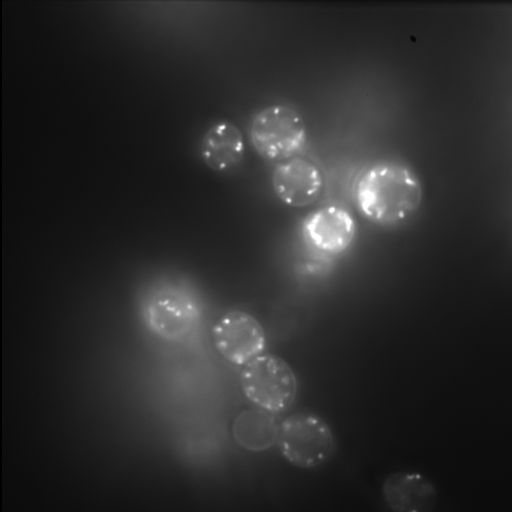

Supplement: Supplementary file 13 — Source Data for Figure 6 [file EMBR-24-e57232-s015.zip › Figure 6/6B/Dinp5152_Nce102_cell.tif]

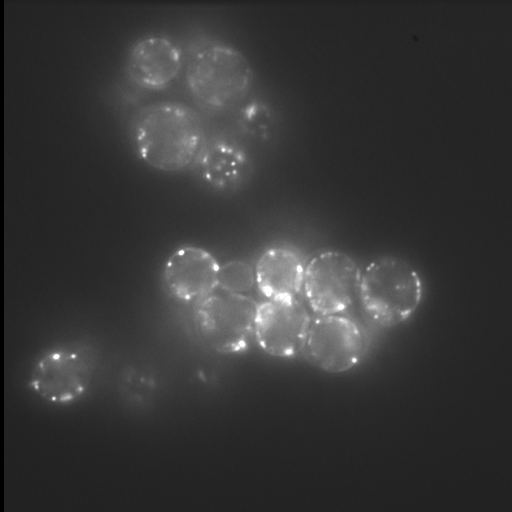

Supplement: Supplementary file 13 — Source Data for Figure 6 [file EMBR-24-e57232-s015.zip › Figure 6/6B/Dinp5152_Sur7_cell.tif]

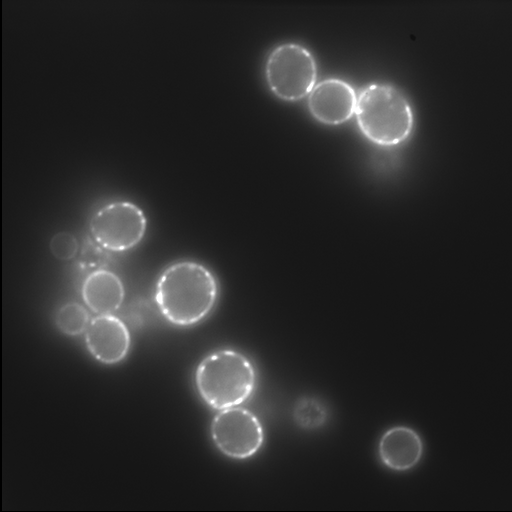

Supplement: Supplementary file 13 — Source Data for Figure 6 [file EMBR-24-e57232-s015.zip › Figure 6/6B/WT_Nce102-Sur7_cell.tif]

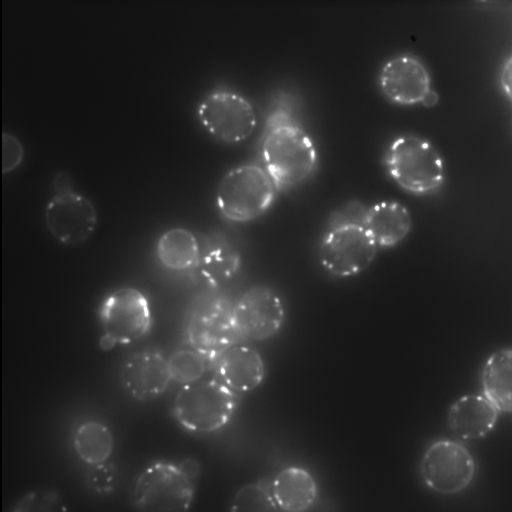

Supplement: Supplementary file 13 — Source Data for Figure 6 [file EMBR-24-e57232-s015.zip › Figure 6/6B/WT_Nce102_cell.tif]

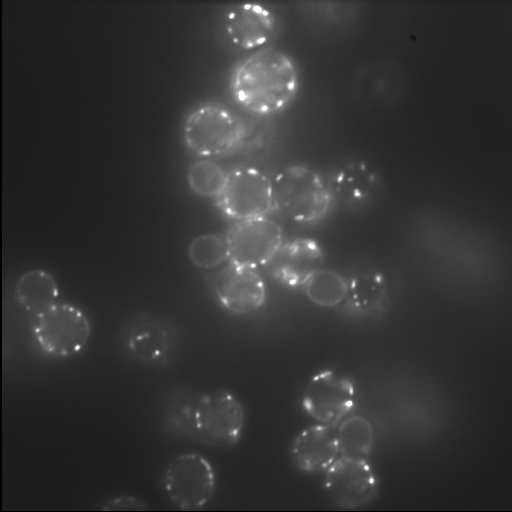

Supplement: Supplementary file 13 — Source Data for Figure 6 [file EMBR-24-e57232-s015.zip › Figure 6/6B/WT_Sur7_cell.tif]

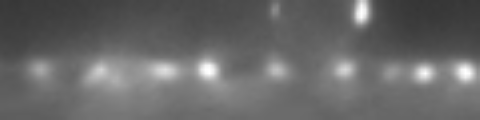

Supplement: Supplementary file 13 — Source Data for Figure 6 [file EMBR-24-e57232-s015.zip › Figure 6/6B/WT_Sur7_linear.tif]

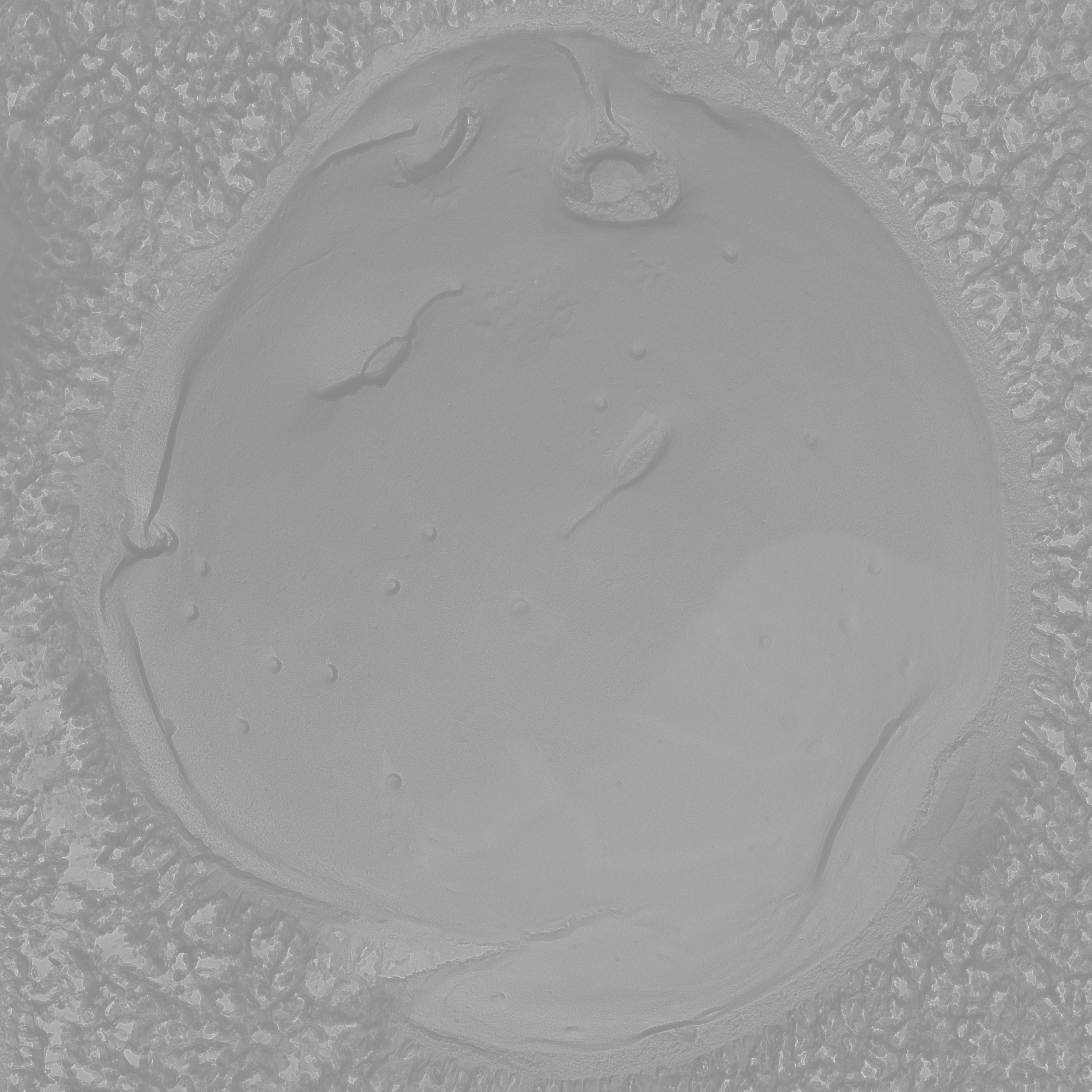

Supplement: Supplementary file 13 — Source Data for Figure 6 [file EMBR-24-e57232-s015.zip › Figure 6/6C/Dinp5152_FreezeFracture.tif]

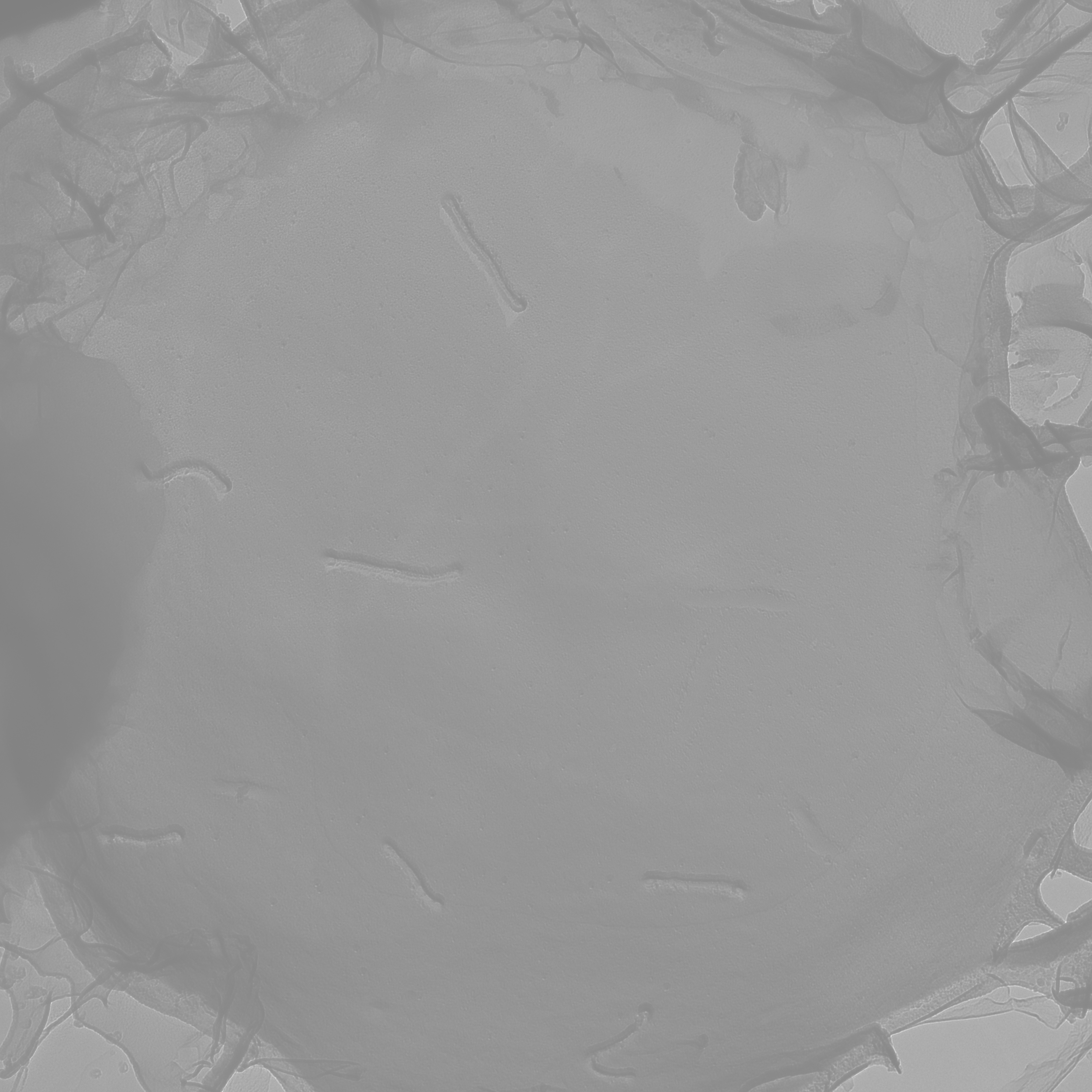

Supplement: Supplementary file 13 — Source Data for Figure 6 [file EMBR-24-e57232-s015.zip › Figure 6/6C/Dinp5152_Nce102-Sur7_FreezeFracture.tif]

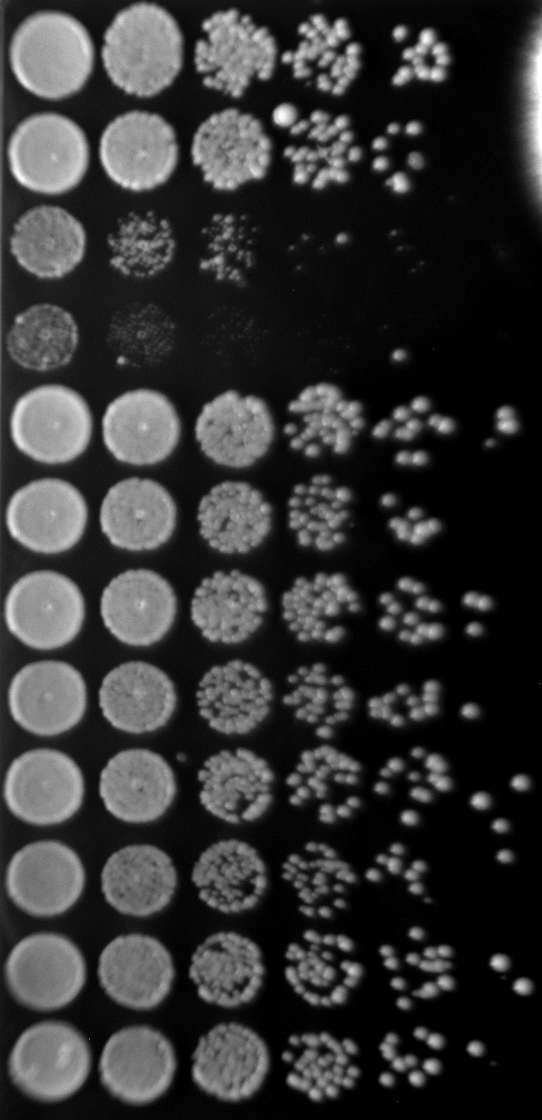

Supplement: Supplementary file 13 — Source Data for Figure 6 [file EMBR-24-e57232-s015.zip › Figure 6/6D/37C.tif]

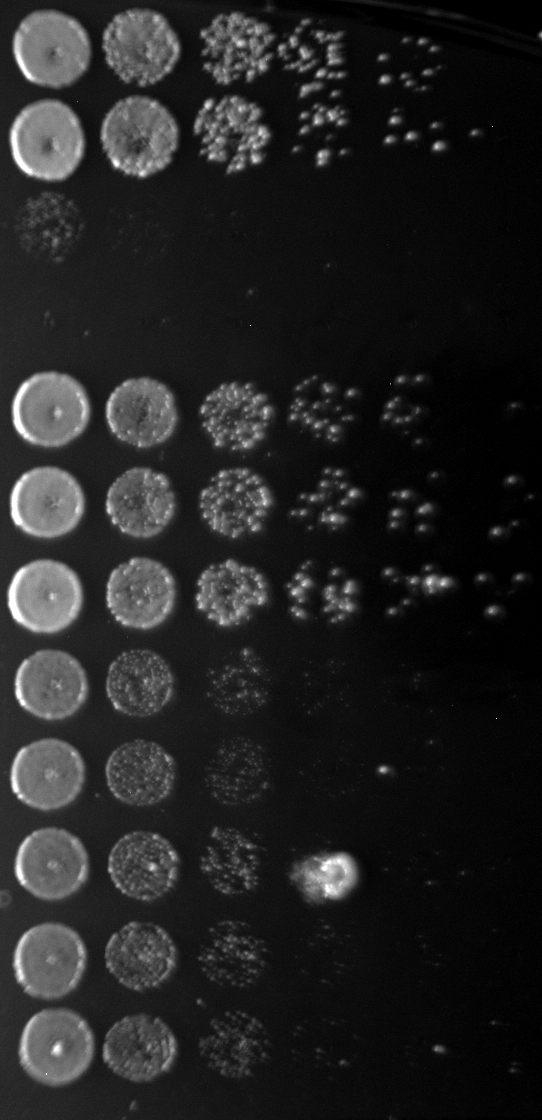

Supplement: Supplementary file 13 — Source Data for Figure 6 [file EMBR-24-e57232-s015.zip › Figure 6/6D/40μgml_CW.tif]

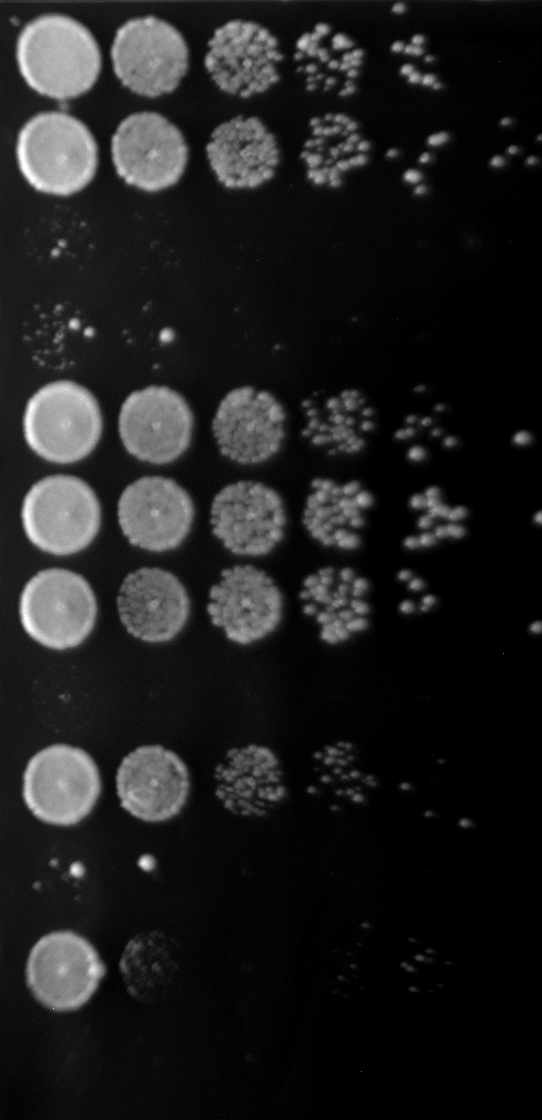

Supplement: Supplementary file 13 — Source Data for Figure 6 [file EMBR-24-e57232-s015.zip › Figure 6/6D/60nm_AbA.tif]

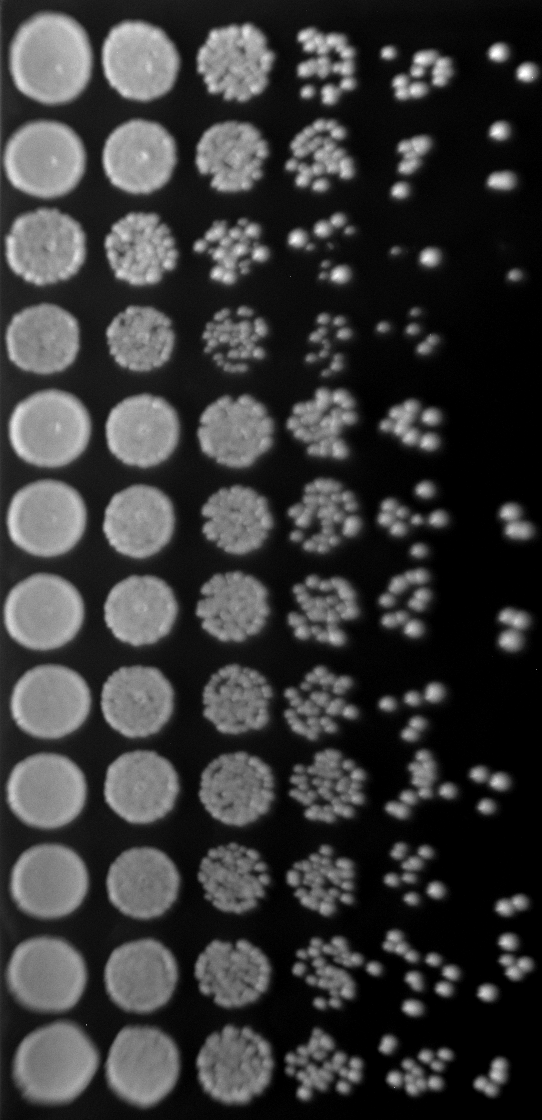

Supplement: Supplementary file 13 — Source Data for Figure 6 [file EMBR-24-e57232-s015.zip › Figure 6/6D/Ctrl.tif]

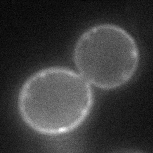

Supplement: Supplementary file 14 — Source Data for Figure 7 [file EMBR-24-e57232-s003.zip › Figure 7/7A/0.5.tif]

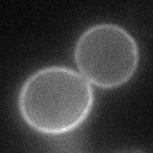

Supplement: Supplementary file 14 — Source Data for Figure 7 [file EMBR-24-e57232-s003.zip › Figure 7/7A/0.tif]

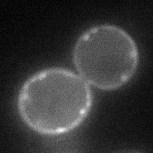

Supplement: Supplementary file 14 — Source Data for Figure 7 [file EMBR-24-e57232-s003.zip › Figure 7/7A/1.tif]

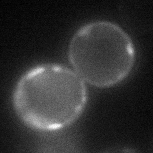

Supplement: Supplementary file 14 — Source Data for Figure 7 [file EMBR-24-e57232-s003.zip › Figure 7/7A/10.tif]

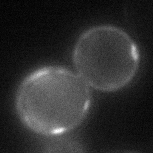

Supplement: Supplementary file 14 — Source Data for Figure 7 [file EMBR-24-e57232-s003.zip › Figure 7/7A/15.tif]

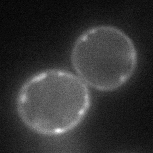

Supplement: Supplementary file 14 — Source Data for Figure 7 [file EMBR-24-e57232-s003.zip › Figure 7/7A/2.tif]

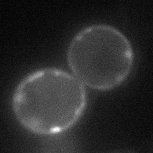

Supplement: Supplementary file 14 — Source Data for Figure 7 [file EMBR-24-e57232-s003.zip › Figure 7/7A/5.tif]

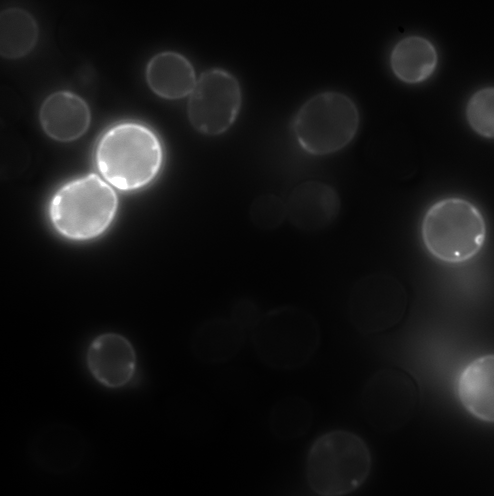

Supplement: Supplementary file 14 — Source Data for Figure 7 [file EMBR-24-e57232-s003.zip › Figure 7/7B/5xKO_Nce102RFP_PHGFP_PalmC.tif]

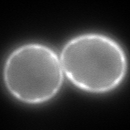

Supplement: Supplementary file 14 — Source Data for Figure 7 [file EMBR-24-e57232-s003.zip › Figure 7/7B/WT_Nce102RFP_PHGFP_PalmC.tif]

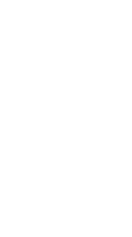

Supplement: Supplementary file 14 — Source Data for Figure 7 [file EMBR-24-e57232-s003.zip › Figure 7/7D/5xKO_2xzoom_0.tif]

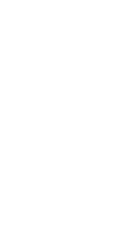

Supplement: Supplementary file 14 — Source Data for Figure 7 [file EMBR-24-e57232-s003.zip › Figure 7/7D/5xKO_2xzoom_100.tif]

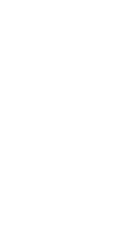

Supplement: Supplementary file 14 — Source Data for Figure 7 [file EMBR-24-e57232-s003.zip › Figure 7/7D/5xKO_2xzoom_200.tif]

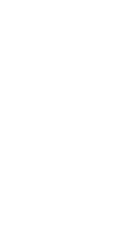

Supplement: Supplementary file 14 — Source Data for Figure 7 [file EMBR-24-e57232-s003.zip › Figure 7/7D/5xKO_2xzoom_series_5s.tif]

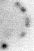

Supplement: Supplementary file 14 — Source Data for Figure 7 [file EMBR-24-e57232-s003.zip › Figure 7/7D/WT_0.tif]

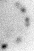

Supplement: Supplementary file 14 — Source Data for Figure 7 [file EMBR-24-e57232-s003.zip › Figure 7/7D/WT_25.tif]

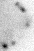

Supplement: Supplementary file 14 — Source Data for Figure 7 [file EMBR-24-e57232-s003.zip › Figure 7/7D/WT_50.tif]

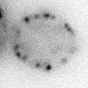

Supplement: Supplementary file 14 — Source Data for Figure 7 [file EMBR-24-e57232-s003.zip › Figure 7/7D/WT_series_5s.tif]

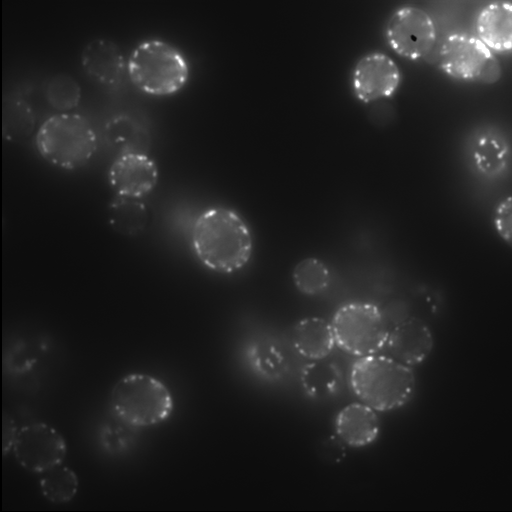

Supplement: Supplementary file 14 — Source Data for Figure 7 [file EMBR-24-e57232-s003.zip › Figure 7/7E/Ctrl_Lsp1mNeGr_Pil1RFP_cell.tif]

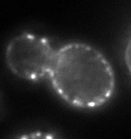

Supplement: Supplementary file 14 — Source Data for Figure 7 [file EMBR-24-e57232-s003.zip › Figure 7/7E/Ctrl_Sur7mNeGr_Pil1RFP_cell.tif]

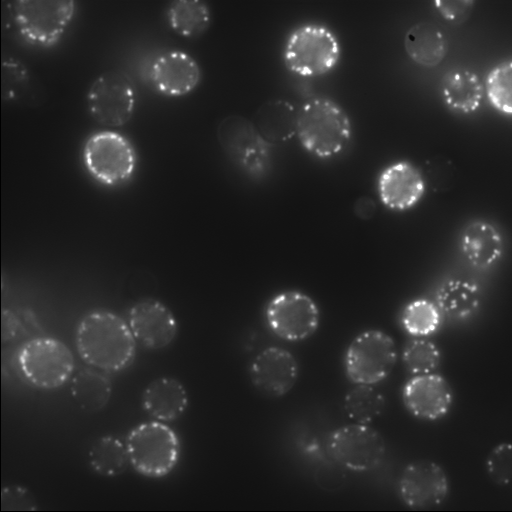

Supplement: Supplementary file 14 — Source Data for Figure 7 [file EMBR-24-e57232-s003.zip › Figure 7/7E/PalmC_Lsp1mNeGr_Pil1RFP_cell.tif]

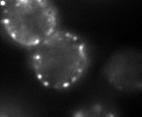

Supplement: Supplementary file 14 — Source Data for Figure 7 [file EMBR-24-e57232-s003.zip › Figure 7/7E/PalmC_Sur7mNeGr_Pil1RFP_cell.tif]

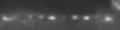

Supplement: Supplementary file 14 — Source Data for Figure 7 [file EMBR-24-e57232-s003.zip › Figure 7/7E/PalmC_Sur7mNeGr_Pil1RFP_linear.tif]

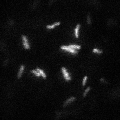

Supplement: Supplementary file 14 — Source Data for Figure 7 [file EMBR-24-e57232-s003.zip › Figure 7/7F/Pil1Halo_Ctrl.tif]

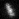

Supplement: Supplementary file 14 — Source Data for Figure 7 [file EMBR-24-e57232-s003.zip › Figure 7/7F/Pil1Halo_Ctrl_zoom.tif]

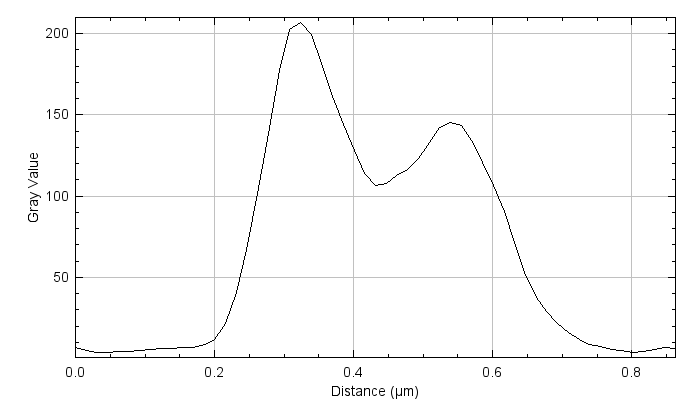

Supplement: Supplementary file 14 — Source Data for Figure 7 [file EMBR-24-e57232-s003.zip › Figure 7/7F/Plot of Pil1Halo_PalmC_profile.tif]

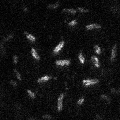

Supplement: Supplementary file 14 — Source Data for Figure 7 [file EMBR-24-e57232-s003.zip › Figure 7/7F/Sur7Halo_Ctrl.tif]

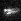

Supplement: Supplementary file 14 — Source Data for Figure 7 [file EMBR-24-e57232-s003.zip › Figure 7/7F/Sur7Halo_Ctrl_zoom.tif]

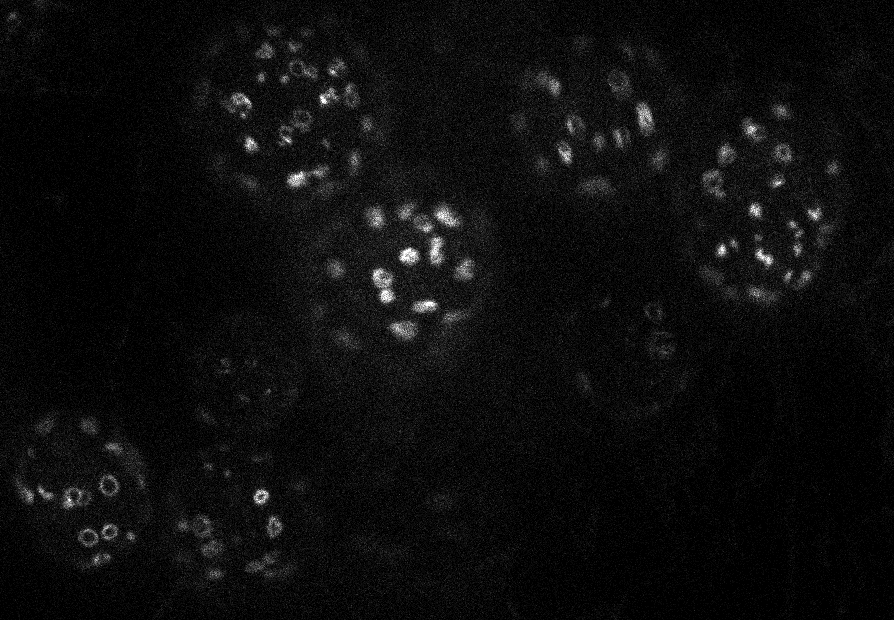

Supplement: Supplementary file 14 — Source Data for Figure 7 [file EMBR-24-e57232-s003.zip › Figure 7/7F/Sur7Halo_PalmC.tif]

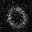

Supplement: Supplementary file 14 — Source Data for Figure 7 [file EMBR-24-e57232-s003.zip › Figure 7/7F/Sur7Halo_PalmC_zoom.tif]
