# Supplementary material for: Tetraspanner‐based nanodomains modulate BAR domain‐induced membrane curvature
Source: EMBO Rep. 2023 Oct 30;24(12):e57232. doi: 10.15252/embr.202357232 (PMC10702824; doi:10.15252/embr.202357232)
Supplement: Supplementary file 15 [file EMBR-24-e57232-s009.pdf]

EMBO reports

# Tetraspanner-based nanodomains modulate BAR domain-induced membrane curvature

Daniel Haase, Christiane Rasch, Ulrike Keller, Yaroslav Tsytsyura, Nataliya Glyvuk, Annegret Elting, Julia Wittmar, Annette Janning, Martin Kahms, Noah Wedlich, Christian Schuberth, Andreas Heuer, Jurgen Klingauf, and Roland Wedlich-Soldner
**DOI: 10.15252/embr.202357232**

*Corresponding author(s): Roland Wedlich-Soldner (wedlich@uni-muenster.de)*



## Review Timeline:

| | |
|---|---|
| Transfer Date: | 23rd Mar 23 |
| Editorial Decision: | 30th Mar 23 |
| Revision Received: | 6th Jul 23 |
| Editorial Decision: | 29th Sep 23 |
| Revision Received: | 2nd Oct 23 |
| Accepted: | 11th Oct 23 |

*Editor: Martina Rembold*


**Transaction Report: This manuscript was transferred to EMBO reports following peer review at The EMBO Journal.**

**Referee #1 Review**
Received: 12th Dec 22

Report for Author:
Comments for Hasse et al.,
This paper describes the role of tetraspanins in the eisosome membrane furrows for a possible role of keeping their shape, presumably by preventing the closure of the open hole. The story is quite interesting, though there is no direct evidence in term of membrane-shaping manipulations by tetraspanin itself. The story is interesting but difficult to follow before memorizing the function of each gene and thus would benefit from rewriting. I would like to make several points for improving the manuscript.

Major points

1. The authors made a significant effort to make the 5-genes knockout of teteraspanins, It would be better to erase the last remaining one, Nce102 on the 5-gene knockout line (5xdelta).

2. The BAR domain protein Pil1/Lsp1 deletion on the 5 genes (5xdelta) or 5xdelta plus Nce102 KO would be interesting to see whether these proteins are the essential building block of the furrow.

3. I am confused about the tunneling phenotype that the authors propose. The BAR domain alone should make just the invaginations, only one end of the tubules at the plasma membrane, but the phenotype of the 5xdelta is the two ends on the plasma membrane, and thus the tunnel is proposed. The tunneling is from the EM sections, but I am not convinced with that because the inside of the tubules is not visible, and thus the EM sections would be the section of the larger cup structure, of which part would have Nce102 with the relatively straight appearance.
Then I suggest the observation of extracellular dye to mark the region connected to the extracellular space.

4. The point 3, where the tunnel is really tunnel connected to the plasma membrane at two ends, will also be clearer with 5xdelta plus Nce102 KO.

5. The lipid enzyme KO would be confirmed by checking the lipids that the cells have by extracting the lipids and running TLC.

Minor point
The absence of band in IP can not guarantee the absence of interaction. And line 133 should be modified.

**Referee #2 Review**
Received: 16th Jan 23

Report for Author:
Haase et al. provides novel evidence concerning the organization and localization of proteins in MCCs/eisosomes using superresolution microscopy and freeze fracture EM. The authors show compelling data that tetraspanner proteins Sur7 and Nce102 show differing localizations in the eisosome, with Sur7 decorating the top edge of eisosome "halfpipe" furrows, and Nce102 localizing to the base. The authors then go on to propose a model suggesting the function of each of these proteins in eisosome formation and stabilization, with Sur7 preventing membrane closure on the top edge, and Nce102 promoting BAR domain-scaffolded curvature on the bottom. Much of this interpretation hinges on a phenotype seen upon knockout of all 5 Sur7 family members (5x knockout), causing tubular hemi-toroidal furrows in the plasma membrane that colocalize with other eisosome components. Since the authors propose that Sur7 acts to prevent membrane closure of eisosomes, they assert that in the 5x knockout condition the upper membrane closes in on itself, leaving a BAR protein-scaffolded tubular membrane. We believe this assertion lacks sufficient evidence, and it is very difficult to rationalize how membrane topology would allow the transition between wild-type eisosomes and these tubular invaginations to be possible. This phenotype has been reported in several other papers, and in particular, Singer-Krüger et al. (Journal of Cell Science, 1998) suggests a different method for the formation of these structures upon Inp51 and Inp52 (Sjl1 and 2) knockout (see Figure 3 of referenced paper). The authors do not present any alternative models for the formation of these invaginations other than their own. Since much of the remaining data presented has been reported previously, we cannot recommend publication of the current version of the manuscript. In addition, we believe the interpretation of the data requires significant reconsideration.

Specific Comments:
Our main comment concerns the interpretation of the 5x knockout data. Much of the model in Figure 10 hinges on the phenotype observed upon deletion of all 5 Sur7 family members, which has previously been observed with EM in several papers (Alvarez et al. 2008, Singer-Krüger et al. 1998). In the latter paper, the tubular structures seem to form as a result of abnormally long plasma membrane invaginations upon deletion of Inp51 and Inp52 (Sjl1 and 2) that bend around back to the plasma membrane. The model the authors have proposed for the formation of their similar observed structures (the transition from figure 10A to 10B) does not make sense to us. We do not understand how membrane topologies available in an eisosome allow for tubular structures to form by the partial closing of the top part of the membrane. The authors should provide detail on how they envision that each leaflet of each membrane interacts with the other to form these complex structures. Apart from this, if there are indeed two distinct mechanisms for formation of these tubular structures (the authors proposed method and the method proposed in Singer-Krüger et al.), the authors have no way of distinguishing them using the methods used in this study. In both the EM and superresolution data of the 5x knockout presented in this manuscript, we can see linear invaginations with only one opening (reminiscent of Figure 3 in Singer-Krüger et al.). We do not believe that the authors have provided sufficient evidence to establish a new model for how the observed invaginations form, which creates a fundamental issue with the interpretation of the data in the manuscript.

(Singer-Krüger et al., 1998 JCS)

In addition, the model proposed in Figure 10 hinges on the assumption that BAR proteins sculpt the membrane into a tubular structure. While there is much evidence that purified yeast BAR proteins can sculpt and tubulate membranes in vitro, as far as we are aware there is no direct in vivo evidence that this property holds true in the high turgor pressure environment of the yeast cytosol.

We suggest that the authors look for distinct phenotypes in erg6Δ cells to support the claim that ergosterol enrichment on the upper edges of eisosomes has a function in localizing Sur7 family members. This claim cannot be supported by only showing colocalization of filipin and Sur7.

The authors should also provide more support for their "distance between peaks" metric. It seems that this metric relies on cells being exactly in the medial focal plane, and that any slight deviation of the focal plane down or up could cause significant differences in the observed distance between peaks depending on the orientation of the eisosome invagination. For example, the data for Pil1 and Sur7 in both figure 1E and figure 5B are noticeably different.

**Referee #3 Review**
Received: 19th Dec 22

Report for Author:
Haase et al. report the super-resolution microscopy-based sub-domain organization / structure of a particular membrane domain of the yeast plasma membrane (PM), the MCC/eisosome, which forms furrow-like invaginations. Their findings identify, for the first time, structural roles of two families of tetraspanners, Sur7 and Nce102, in the assembly of the invagination. Sur7 members are located at an ergosterol-enriched subdomain at the upper edge of the invagination and somehow counteract the tubulation induced by the BAR-domain proteins Pil1 and Lsp1, thereby preventing closure of the furrow into a tubule. Nce102, on the contrary, is localized deeper in the furrow and is required for Pil1 activity, which requires PIP2.
The results are novel and reveal a non-homogeneous organization of the eisosome domain at the sub-domain level. The authors show that this sub-domain organization is critical for the proper formation/assembly of the domain into characteristic furrow-like invaginations, and consequently for the physiological function of MCCs in sphingolipid signalling and cell-wall integrity. Eisosomes are conserved in fungi and important for pathogenicity, so this work will be most interesting for the fungal cell biology and pathogenicity communities. In addition, such a nanoscale organization could give important insight into the organization of similar tetraspan proteins in mammalian membrane domains, like claudins and occludins in tight junctions.

The manuscript is in general well-written and clear, and the results for the subdomain organization are strong and convincing. A general feeling of this reviewer though, reflected also in the comments below, is that as the story progresses the manuscript is less developed (from results describing Figure 5 and below) and the text progressively becomes less accurate and more difficult to follow. I would suggest a revision of this second part of the results section, to make it precise and clear, as is the first part.
In addition, and especially in this second part of the results, sometimes control experiments are missing, or interpretations drawn are not fully supported by the data presented, analysed below:

Major comments.

A first category of comments is about the role of PIP2. Despite the results presented, the role of PIP2 in MCC assembly remains poorly undestood. Some of the proposals below could shed more light on this relationship.

1. In Figure 7F, is it possible to additionally monitor Mss4 localization without overexpression? Because in Figure 7A it is shown that Mss4 overexpression leads to tubulation, suggesting that Sur7 members are already misplaced from Pil1 and Nce102-containing tubulated eisosomes, similarly to what is shown for the Δinp51/52 mutant in Figure 7C. If this is the case, how does deletion of SUR7 members affect the membrane recruitment of Mss4? A potential explanation could be that, given that the effects of Mss4OE and 5xΔ on membrane tubulation are additive, non-overexpressed Mss4 remains normally at the PM in the 5xΔ strain. Alternative explanations are also welcome.

2. Lines 28-29, 98-99 and elsewhere. To which evidence do the authors base their claim that that PIP2 is found at the bottom of the furrows? Apart from previously published results that PIP2 is required for tubulation by Pil1 and Lsp1, I could not find any data within this manuscript. In Figure 7G, no labelling of 2xPH(PLCδ)-GFP to the characteristic Pil1-containing tubules of the 5xΔ strain is evident, suggesting that either these tubules do not contain any PIP2, or that PIP2 is not accessible to 2xPH(PLCδ)-GFP, for example due to binding by Pil1 and Lsp1. Moreover, overexpression of PIP2 affects Sur7 positioning, which is found at the upper part of the invagination. Importantly, Inp51 in Figure 7E seems to mostly localize to the upper part of the invagination (similarly to Sur7) and not close to the Nce102-containing tubules of the 5xΔ strain, yet, no radial distance measurements are provided. The same applies to Mss4OE in Figure 7F: it seems to localize mostly to the upper part of eisosomes. These results potentially suggest that PIP2 biosynthesis and degradation mainly occur at the upper part of the eisosome. I suggest the authors to either provide evidence for an increased PIP2 concentration to the lower part of eisosomes, or rephrase their claims and include a dedicated section in the discussion about the potential localization of PIP2. This discussion could include the arguments above and alternative explanations, considering also the possibility that PIP2 is homogeneous within the whole eisosomal invagination. Additional experiments to address some of the points raised above (e.g. about the subdomain distribution of Mss4 or Inp51) would also be welcome but not essential.

3. If the levels of PIP2 at the PM are reduced in the 5xΔ, how come tubulation by Pil1 (which requires PIP2) is more efficient? Could the results in Figure 7G be, somehow, the outcome of increased tubules within the cytoplasm? The authors could discuss such this.

4. Results in Figure 8 are most interesting and suggest a role of PIP2 in the misplacement of Sur7 upon reduction of PM tension. The authors show that PIP2 (using 2xPH-PLCδ) clustering under these conditions is closely associated (but no high-resolution colocalization is provided) with Nce102, and that Sur7 is misplaced compared to Pil1. Yet, the authors do not directly investigate the spatiotemporal relationship between Sur7 and PIP2. Such an investigation would show whether PIP2 clusters are more associated to Pil1 or to the misplaced Sur7 upon acute reduction of membrane tension. Moreover, the authors could directly investigate whether tubulation is formed under these conditions, for example by determining with STED microscopy the radial localization of Pil1 upon PalmC treatment. In addition, since MCCs are stable and static over long time-periods, it would be interesting to investigate, using time-lapse microscopy, which eisosome components (if any) remain static and which are missplaced after PalmC treatment. In other words, is it Sur7 or Pil1 (or neither?) that remain at the same position after PalmC treatment, and is this somehow related to PIP2 distribution? These results could give important mechanistic insights, potentially also coupled with suggestion 3 (see also major comments 7 and 9).

Some other major comments:

5. Line 237. It is not clear to this reviewer what the authors mean by saying that the "Sur7 C-terminus was not required for strand formation per se". In Figure 4E it is indeed clear that in the wt, Sur7ΔC is normally recruited to eisosomes, but localization at the edges of both strands of the invagination is not as clear as shown for wt Sur7 in Figure 2. Moreover, in the 5xΔ strain, curved invaginations and tubules are mostly evident, as expected since Sur7ΔC cannot rescue membrane tubulation. Consequently, the argument that the c-terminus is not required for strand formation is not clear from the data presented. To strengthen such a statement, the authors could provide micrographs similar to Figure 2, showing that Sur7ΔC is recruited to both strands of the invagination in the wt, or to both strands of curved invaginations in the 5xΔ mutant, along with measurements of the intensity profile. In case the authors intended to say something different, please rephrase.

6. Results presented in Figure 5E and 5F are most interesting, but incomplete. The results indicate that Nce102 has a structural role in MCC assembly that goes beyond its previously described role in regulating Pil1 phosphorylation. The main data supporting this is that Pil1-4A expression rescues defective MCC formation and tubulation in the 6xΔ strain, yet these tubules are shorter when compared to the 5xΔ strain expressing wt Pil1. Please notice that the two strains compared have two differences: expression of Pil1 or Pil1-4A and presence or absence of NCE102. In order to define the contribution of Nce102 and Pil1-4A in the phenotype described, an important control is missing. A 5xΔ strain expressing Pil1-4A-Halo instead of Pil1-Halo. Importantly, the authors already show that, in the absence of Nce102, the distance between Pil1-4A and Sur7 is shorter than the one of Pil1 and Sur7 in the few remaining eisosomes (Figure 5B). This could potentially suggest that Pil1-4A is somehow less efficient that wt Pil1 for membrane tubulation in the absence of Nce102. Alternatively, in the absence of Nce102 (and due to unknown reasons) when Pil1-4A is expressed an increased number of shallower MCCs could be formed instead of a smaller number of normal-depth eisosomes. An additional control would be a Pil1-4A expressing wt strain. These controls could clarify whether the shorter tubes observed in 6xΔ Pil1-4A are due to the absence of Nce102 or defective Pil1-4A tubulating activity, or a combination of both.

7. Lines 92-93, 377-379, 403-404. The arguments presented by the authors that Sur7 members "counteract the forces exerted by BAR domain proteins at the furrow base" are not strong. This reviewer agrees that Sur7 members seem to somehow modulate/diminish the tubulating function of the BAR domain proteins, but the underlying molecular mechanism has not been elucidated. The mechanisms could, thus, be others not related to "counteracting the forces", like for example a direct or indirect regulation of Pil1 and Lsp1 function, or an anchoring role (via contacts with the cell wall? See also major comment 9 and suggestion 3) of the invaginations to the plasma membrane. The authors could provide additional evidence in support of their claims, or rephrase the text to include alternative explanations for their results.

8. The idea of fusing Sur7 to Nce102 in an attempt to tether Sur7 to MCCs and rescue eisosome tubulation in the Δinp51/52 strain is excellent, and the results very promising. Yet, a few important controls are missing, since the results presented could have alternative an interpretation: The fusion, instead of tethering Sur7 to MCCs, could misplace Nce102 too out of MCCs. In the experiments presented, this cannot be ruled out since the MCC localization of the chimeric protein under all conditions has not been examined. More specifically:
a) In Figure 9A the authors measure distance from the plasma membrane (and not from MCCs as usually in other figures, but that is not feasible here indeed) of Sur7, Nce102, and of the chimeric protein. Data show that the latter is closer to the plasma membrane, and the authors interpret this as a rescue of the tubulation phenotypes. Yet, since there is no marker of the upper or lower parts of eisosomes, it is possible that tubulation is not rescued and it is only the chimera that is found closer to the plasma membrane, either in the upper part of eisosomes or even outside eisosomes (especially for the Δinp51/52 strain, as shown for Sur7 in Figures 7C and 9C). I propose the authors to repeat the experiment with a non-fluorescently-tagged Nce102-Sur7 chimera and check the distance of Pil1 (as a marker of tubule formation) from the plasma membrane under the same conditions, for determining membrane tubulation.
b) The same alternative explanation could be valid for Figure 9C. The fact that the Nce102-Sur7 chimera colocalizes with Sur7 in the Δinp51/52 strain does not necessarily mean that this colocalization occurs in eisosomes. The degree of colocalization of the chimeric protein with Pil1 (or Nce102) in both the wt and the Δinp51/52 strains should be provided.

9. Line 381-382. The data presented by the authors are in agreement with Sur7 members forming parallel strands along the longitudinal axis at the upper edges of MCC/eisosome furrows, and this reviewer agrees that this is a very likely organization. Yet, the high-resolution molecular structure of these strands is missing and alternative modes of organization could be envisaged. For example, are these single strands or many parallel strands? Are the Sur7 localized to the curved membrane (at the edge of the plasma membrane and the invagination), to the inner upper part of the invagination, or to the neighboring, flat part of the plasma membrane (outside to the invagination, but in close contact with it), or combinations of the previous? Such types of organization could potentially suggest mechanistic insights into how Sur7 members, via their C-termini, inhibit Pil1 tubulation, e.g. via anchoring to the cell wall (See also major comment 7 and suggestion 3). In the absence of experimental evidence or data from the literature (about the topology of other tetraspanners as well?), I invite the authors to present a model that is as broad as possible.

10. In figure 9D, it is shown that the Nce102-Sur7 chimeric protein cannot rescue hypersensitivity to CW of the 5xΔ mutant. In this growth test, expression of Sur7 alone is not shown, which would be expected to rescue the phenotype. If this is the case, would this mean that the chimeric protein rescues only the Nce102-dependent phenotypes (sphingolipid signaling) and not Sur7-

phenotypes? It would be nice to have such a control in the growth test. Moreover it would be nice to know whether the increased resistance of the 5xΔ to PHS could be reversed by expression of the Nce102-Sur7 chimera.

Minor comments

1. Line 72. This reviewer agrees that the MCCs/eisosomes are characteristic, stable invaginations of the yeast plasma membrane, but the statement that the yeast membrane is otherwise flat could be misleading. In fact, there are also sites of endocytosis, which correspond to smaller and dynamic, transiently-formed invaginations with different size. The sentence could be rephrased to exclude misconceptions.

2. Line 95. Can it be excluded from current and previous evidence that Nce102 somehow structurally supports the formation of deeper invaginations, without promoting BAR domain-induced curvature?

3. Lines 108-109, please clarify that A08184g was heterologously expressed in S. cerevisiae and the results are not obtained in K. lactis cells.

4. Please correct the positioning of the namings in EV1C, so that everything is visible.

5. Line 346. "found" could be replaced by "verified" or "confirmed".

6. The radial distance of Pil1 and Sur7 in the micrographs of Figure 5A is not as clear as in Figure 1, although measurements are similar. The authors could show a more representative micrograph here.

7. Figure 8D. micrographs for the radial distance of Pil1 and Lsp1 are missing.

8. Line 425-426. Do these results refer to conditions of the Pil1-4A-expressing strains? Please specify.

9. Line 831. Quantifications refer to F and not E.

Suggestions

1. A previous publication has analyzed the periprotein lipidome of several eisosomal proteins, including Sur7 (van 't Klooster et al., 2020, eLife). The authors have reported that the periprotein lipidome of Sur7 is not enriched in ergosterol, data that seem to not agree with the data presented by the authors in the current manuscript. The authors could try to include this previous work in the discussion and speculate a bit about the reasons of potential differences.

2. Regarding the effect of AbA on the accumulation of Sur7 in MCCs, it has previously been reported that depletion of complex sphingolipids leads to eisosome disassembly (Fröhlich et al. 2009). Given that Sur7 strand partitioning is not affected, could the reduced MCC partitioning of Sur7 be due to deformation of eisosomes? Have the authors tried to do the same experiment in a Pil1-4A expressing strain, in which MCCs are resistance to complex sphingolipid depletion-induced disassembly (Fröhlich et al., 2009)? This should be easy, but I understand that it is not critical, since the authors do not claim an important role of sphingolipids for MCC partitioning of Sur7.

3. Young et al. 2002 have reported a change in typical PM distribution of Sur7 upon digestion of the cell wall. This result was a matter of debate in subsequent publications (Malinska et al, 2004, Spira et al., 2012). In the current manuscript, the authors find a very interesting but poorly understood relationship between PM tension, PIP2 distribution and Sur7 MCC partitioning, which directs the formation of static and stable MCC furrows at the plasma membrane. A potential mechanism via which Sur7 proteins would counteract the tubulation of Pil1 could be via anchoring to the cell wall. It would be interesting to test this idea in light of the current findings. Some ideas would be to check PIP2 distribution, Sur7 and Pil1 colocalization in protoplasts, and whether Pil1-containing tubules are formed in protoplasts. Yet, this is only a suggestion.

4. The relationship of sterols and Sur7 seems most interesting but not analyzed in detail in the manuscript. The authors could potentially want to check if sterols are required for the proper MCC localization of Sur7 members, e.g. by depleting ergosterol using a conditional sterol biosynthesis mutant. Additionally, the authors could determine the localization of fillipin under conditions of PIP2 overexpression: Will it still segregate with Pil1-eisosomes, or with the Sur7 outside MCCs? This reviewer understands well that this could be another story though.

## 1st Editorial Decision 30th Mar 2023

Dear Roland,

Thank you for the transfer of your research manuscript to EMBO reports. As discussed, I would like to invite you to revise your manuscript for potential publication in EMBO reports along the lines we discussed during our virtual meeting last week. Foremost, it will be important to address the concerns regarding the tunnelling phenotype you observe.

Please address all referee concerns in a complete point-by-point response. Acceptance of the manuscript will depend on a positive outcome of a second round of review. It is EMBO reports policy to allow a single round of revision only and acceptance or rejection of the manuscript will therefore depend on the completeness of your responses included in the next, final version of the manuscript.

We realize that it is difficult to revise to a specific deadline. In the interest of protecting the conceptual advance provided by the work, we recommend a revision within 3 months (June 30). Please discuss the revision progress ahead of this time with me if you require more time to complete the revisions.

*****IMPORTANT NOTE:
We perform an initial quality control of all revised manuscripts before re-review. Your manuscript will FAIL this control and the handling will be DELAYED if the following APPLIES:

1) A data availability section providing access to data deposited in public databases is missing. If you have not deposited any data, please add a sentence to the data availability section that explains that.

2) Your manuscript contains statistics and error bars based on n=2. Please use scatter blots in these cases. No statistics should be calculated if n=2.

When submitting your revised manuscript, please carefully review the instructions that follow below. Failure to include requested items will delay the evaluation of your revision.*****

When submitting your revised manuscript, we will require:

1) a .docx formatted version of the manuscript text (including legends for main figures, EV figures and tables). Please make sure that the changes are highlighted to be clearly visible.

2) individual production quality figure files as .eps, .tif, .jpg (one file per figure).
Please download our Figure Preparation Guidelines (figure preparation pdf) from our Author Guidelines pages https://www.embopress.org/page/journal/14693178/authorguide for more info on how to prepare your figures.

3) a .docx formatted letter INCLUDING the reviewers' reports and your detailed point-by-point responses to their comments. As part of the EMBO Press transparent editorial process, the point-by-point response is part of the Review Process File (RPF), which will be published alongside your paper.

4) a complete author checklist, which you can download from our author guidelines (<https://www.embopress.org/page/journal/14693178/authorguide>). Please insert information in the checklist that is also reflected in the manuscript. The completed author checklist will also be part of the RPF.

5) Please note that all corresponding authors are required to supply an ORCID ID for their name upon submission of a revised manuscript (<https://orcid.org/>). Please find instructions on how to link your ORCID ID to your account in our manuscript tracking system in our Author guidelines (<https://www.embopress.org/page/journal/14693178/authorguide#authorshipguidelines>)

6) We replaced Supplementary Information with Expanded View (EV) Figures and Tables that are collapsible/expandable online. A maximum of 5 EV Figures can be typeset. EV Figures should be cited as 'Figure EV1, Figure EV2" etc... in the text and their respective legends should be included in the main text after the legends of regular figures.

- For the figures that you do NOT wish to display as Expanded View figures, they should be bundled together with their legends in a single PDF file called *Appendix*, which should start with a short Table of Content. Appendix figures should be referred to in the main text as: "Appendix Figure S1, Appendix Figure S2" etc. See detailed instructions regarding expanded view here: <https://www.embopress.org/page/journal/14693178/authorguide#expandedview>

- Additional Tables/Datasets should be labeled and referred to as Table EV1, Dataset EV1, etc. Legends have to be provided in

a separate tab in case of .xls files. Alternatively, the legend can be supplied as a separate text file (README) and zipped together with the Table/Dataset file.

7) Please note that a Data Availability section at the end of Materials and Methods is now mandatory. In case you have no data that requires deposition in a public database, please state so instead of refereeing to the database.
See also < https://www.embopress.org/page/journal/14693178/authorguide#dataavailability>). Please note that the Data Availability Section is restricted to new primary data that are part of this study.

8) At EMBO Press we ask authors to provide source data for the main figures. Our source data coordinator will contact you to discuss which figure panels we would need source data for and will also provide you with helpful tips on how to upload and organize the files.

Additional information on source data and instruction on how to label the files are available <https://www.embopress.org/page/journal/14693178/authorguide#sourcedata>.

9) The journal requires a statement specifying whether or not authors have competing interests (defined as all potential or actual interests that could be perceived to influence the presentation or interpretation of an article). In case of competing interests, this must be specified in your disclosure statement. Further information: https://www.embopress.org/competing-interests

10) Figure legends and data quantification:
The following points must be specified in each figure legend:

- the name of the statistical test used to generate error bars and P values,
- the number (n) of independent experiments (please specify technical or biological replicates) underlying each data point,
- the nature of the bars and error bars (s.d., s.e.m.)

- If the data are obtained from n {less than or equal to} 5, show the individual data points in addition to the SD or SEM.
- If the data are obtained from n {less than or equal to} 2, use scatter blots showing the individual data points.

Discussion of statistical methodology can be reported in the materials and methods section, but figure legends should contain a basic description of n, P and the test applied.

See also the guidelines for figure legend preparation:
https://www.embopress.org/page/journal/14693178/authorguide#figureformat

- Please also include scale bars in all microscopy images.

11) Our journal encourages inclusion of *data citations in the reference list* to directly cite datasets that were re-used and obtained from public databases. Data citations in the article text are distinct from normal bibliographical citations and should directly link to the database records from which the data can be accessed. In the main text, data citations are formatted as follows: "Data ref: Smith et al, 2001" or "Data ref: NCBI Sequence Read Archive PRJNA342805, 2017". In the Reference list, data citations must be labeled with "[DATASET]". A data reference must provide the database name, accession number/identifiers and a resolvable link to the landing page from which the data can be accessed at the end of the reference. Further instructions are available at <https://www.embopress.org/page/journal/14693178/authorguide#referencesformat>.

12) As part of the EMBO publication's Transparent Editorial Process, EMBO reports publishes online a Review Process File to accompany accepted manuscripts. This File will be published in conjunction with your paper and will include the referee reports, your point-by-point response and all pertinent correspondence relating to the manuscript.

You are able to opt out of this by letting the editorial office know (emboreports@embo.org). If you do opt out, the Review Process File link will point to the following statement: "No Review Process File is available with this article, as the authors have chosen not to make the review process public in this case."

We would also welcome the submission of cover suggestions, or motifs to be used by our Graphics Illustrator in designing a cover.

I look forward to seeing a revised form of your manuscript when it is ready and please let me know if you have questions or comments regarding the revision.

Kind regards,

Martina

Martina Rembold, PhD
Senior Editor
EMBO reports

Dear Fiona,

Below is a point by point reply to all issues that were raised. Original comments are in black, replies in blue, links to corresponding changes in the revised manuscript are indicated in each reply. We have marked large modification in the manuscript in red.

The main changes to the manuscript are:

- We focused our manuscript with now 3 main parts:
  a) The sub-domain organization of MCC/eisosomes and parallel strand-localization of tetraspanners.
  b) The closure of furrow into half-toroidal tubes in the absence of Sur7 tetraspanners and the role of the Sur7 C-terminus.
  c) The formation of equivalent tubes and the loss of Sur7 localization upon overproduction of PIP2 with the rescue of this effect and all accompanying phenotypes by synthetic anchoring of Sur7 to MCC/eisosomes.
  Additional aspects that were previously included but were not relevant to the central points (role of ergosterol, function of Nce102) were removed or toned down to focus our arguments. The key statements are now that Sur7 tetraspanners oppose BAR-mediated curvature and thereby stabilize furrows – and that Sur7 and PIP2 show mutual interactions to modulate PM topography.

- We added new STED and freeze fracture EM data, improved quantifications and structural predictions to further support the formation of half-toroidal tubes in the absence of Sur7.

- We modified the discussion to focus on the key changes in PM topography. Most importantly, we teamed up with the group of Andreas Heuer to run simulations of membrane systems with either BAR domains alone or BAR domains + tetraspanners. We also discuss properties of the yeast system that could contribute to the observed changes in PM topography

We think that with these changes our manuscript has been greatly improved and want to thank the reviewers for their thoughtful and detailed comments. We hope that the manuscript is now acceptable for publication at EMBO reports.

Roland Wedlich-Söldner

**Referee #1:**

This paper describes the role of tetraspanins in the eisosome membrane furrows for a possible role of keeping their shape, presumably by preventing the closure of the open hole. The story is quite interesting, though there is no direct evidence in term of membrane-shaping manipulations by tetraspanin itself. The story is interesting but difficult to follow before memorizing the function of each gene and thus would benefit from rewriting.

We thank the reviewer for his interest in our results. As this also refers to several comments below, we want to make clear that our results concern a class of tetraspanners (Sur7 family) that is distinct from the classical mammalian tetraspanins. We agree that none of our results proves the direct shaping of membranes by Sur7 tetraspanners. The required in vitro experiments to show this would be beyond the scope of this manuscript but are clearly a focus of future work on this topic. However, we think that our in vivo findings using super resolution microscopy and genetic manipulations provide very strong evidence in support of our mechanistic model. To further strengthen our conclusions we have now added structural predictions (PPM 3.0, Lomize et al, 2022) that show a unique preference of Sur7 tetraspanners for highly positively curved membranes (Fig. 2E, p6 lines 175-178). We have also added new freeze fracture EM images indicating the formation of half-toroid tubes in strains with $PIP_2$ overproduction (Fig. 6C, p9 line 318). Finally, we appreciate the difficulties of following our story and have therefore substantially reorganized the text to provide more focus on our main story line (see general comments). We hope that the manuscript is now more accessible to scientists outside the yeast community. In particular, we have removed sections on the role of lipids beyond $PIP_2$ and on the role of Nce102, as neither provided information on the key mechanisms for PM shaping.

**Major points**

1. The authors made a significant effort to make the 5-genes knockout of teteraspanins, It would be better to erase the last remaining one, Nce102 on the 5-gene knockout line (5xdelta).

The 6x KO was and is actually included in the manuscript (Fig. 3F), but as Sur7 and Nce102 have very distinct roles in MCC/eisosome formation this strain is difficult to interpret. In particular, the general loss of MCC/eisosomes upon deletion of Nce102 makes further quantifications difficult. Importantly, to avoid confusion with the different tetraspanner families and gene names we decided to remove most of the results obtained on the Nce102 mutant and only include some of those data as control experiments. For better clarity we now exclusively focus on the role of Sur7 tetraspanners and the interaction with $PIP_2$.

2. The BAR domain protein Pil1/Lsp1 deletion on the 5 genes (5xdelta) or 5xdelta plus Nce102 KO would be interesting to see whether these proteins are the essential building block of the furrow.

The two 6x KO strains (5xΔ + Δpil1 and 5xΔ + Δnce102) are included in the manuscript (Fig. 3F). Deletion of either severely disrupts MCC/eisosome formation, which makes further analysis of membrane topography in combined knock outs difficult but clearly shows that the BAR protein Pil1 is essential for formation of both, furrow and half-toroidal tube.

**3.** I am confused about the tunneling phenotype that the authors propose. The BAR domain alone should make just the invaginations, only one end of the tubules at the plasma membrane, but the phenotype of the 5xdelta is the two ends on the plasma membrane, and thus the tunnel is proposed. The tunneling is from the EM sections, but I am not convinced with that because the

inside of the tubules is not visible, and thus the EM sections would be the section of the larger cup structure, of which part would have Nce102 with the relatively straight appearance. Then I suggest the observation of extracellular dye to mark the region connected to the extracellular space.

The formation of membrane tubes that remain connected to the PM at both ends (half-toroidal topography) is indeed a central finding and a concern that was also raised by reviewer 2. We now provide additional evidence for the presence of half-toroidal tubes in the 5xΔ as well as Δinp51/52 cells by freeze fracture EM and STED (Fig. 3G, 3H, 5B, 6C). We have attempted to label the inside of tubes using extracellular dyes but were not successful with this. This is likely due to physical obstruction by the yeast cell wall, which is closely attached to the PM in contrast to bacteria where the cell wall leaves some periplasmic space. Importantly, the distinction of single tip-growing tubes from half-toroid tubes (see also reply to reviewer 2) would not be possible with this approach, as the tube lumen would be connected to the outside in either conformation. We have also included an extensive discussion on how tube closure and formation of half-toroid tubes at the yeast PM could occur (p12-13).

4. The point 3, where the tunnel is really tunnel connected to the plasma membrane at two ends, will also be clearer with 5xdelta plus Nce102 KO.

We had included this experiment (Fig 3F). However, as removal of Nc102 leads to loss of most invaginations, the effect on tube formation could not be analyzed in this strain.

5. The lipid enzyme KO would be confirmed by checking the lipids that the cells have by extracting the lipids and running TLC.

We did perform shot gun lipidomics for the 5xΔ and various phospholipid mutants (removal of PE, PS or PC) and these showed the expected changes in whole-cell lipid composition (no changes for 5xΔ, removal of specific phospholipid group in the other mutants). Unfortunately, PIP variants are not part of standard lipid MS. However, lipid changes in the Δ*inp51/52* or Mss4 OE strains were reported previously and we now include those citations.

**Minor point**

The absence of band in IP cannot guarantee the absence of interaction. And line 133 should be modified.

We now modified this statement to “we found no evidence for strong physical interactions” (line 136).

**Referee #2:**

Haase et al. provides novel evidence concerning the organization and localization of proteins in MCCs/eisosomes using super resolution microscopy and freeze fracture EM. The authors show compelling data that tetraspanner proteins Sur7 and Nce102 show differing localizations in the eisosome, with Sur7 decorating the top edge of eisosome "halfpipe" furrows, and Nce102 localizing to the base. The authors then go on to propose a model suggesting the function of each of these proteins in eisosome formation and stabilization, with Sur7 preventing membrane closure on the top edge, and Nce102 promoting BAR domain-scaffolded curvature on the bottom. Much of this interpretation hinges on a phenotype seen upon knockout of all 5 Sur7 family members (5x knockout), causing tubular hemi-toroidal furrows in the plasma membrane that colocalize with other eisosome components. Since the authors propose that Sur7 acts to prevent membrane closure of eisosomes, they assert that in the 5x knockout condition the upper membrane closes in on itself, leaving a BAR protein-scaffolded tubular membrane. We believe this assertion lacks sufficient evidence, and it is very difficult to rationalize how membrane topology would allow the transition between wild-type eisosomes and these tubular invaginations to be possible. This phenotype has been reported in several other papers, and in particular, Singer-Krüger et al. (Journal of Cell Science, 1998) suggests a different method for the formation of these structures upon Inp51 and Inp52 (Sjl1 and 2) knockout (see Figure 3 of referenced paper). The authors do not present any alternative models for the formation of these invaginations other than their own.

We thank the reviewer for raising this very important point. The half-toroidal tubes at the PM of the 5xΔ indeed represent a very unusual topology and we had not put enough emphasis on this fact in our original manuscript. We have addressed this in our revision in two ways:

1) Experimental support for MCC/eisosomal origin of toroidal tubes: We have performed additional experiments showing that the circular structures in freeze fracture images also occur in the Δ*inp51/51* mutant and that they are rescued by expression of the Nce102-Sur7 chimera that forces localization of Sur7 to MCC/eisosomes (Fig 6C). We also now provide STED images of the cell surface that clearly show the increased end intensities expected for the toroidal appearance in 5xΔ cells (Fig 3G), Δinp51/52 cells (Fig 5B) and upon PalmC treatment (Fig 7F). We have also emphasized our previous findings that the distance between tube ends corresponds to the length of MCC/eisosomes and is increased by overexpression of the Seg1 scaffold (p6, line 202-209). We have attempted to visualize the tubular structures in serial TEM sections but the sub-optimal contrast in our samples unfortunately did not allow clear identification of lipid bilayers and thus of smaller (<50 nm) invaginations (contrast needs to be high to identify cross sections through tubes). Instead, we found many large cup-like invaginations that likely correspond to the vacuole-like structures reported in Singer-Krüger et al., 1998. These invagination contained cell wall material, were labeled by FM4-64 but did not contain Pil1 or Nce102 (Fig EV4). We therefore think that the mutants exhibiting half-toroidal Pil1derived invaginations also show additional cell wall-filled large PM invaginations that are not directly linked to MCC/eisosomes. The link between those different types of invaginations is an interesting question for future studies.
2) We now provide an extensive discussion of the evidence supporting our model and the unique properties of the yeast system that might support tube formation (p12-13). We also explicitly refer to previous observations in Singer-Krüger et al. 1998 and Stefan et al.

2002 in multiple instances and explain that the larger invaginations seen in 5xΔ cells might be formed by alternative mechanisms (p7). Importantly, we found large invaginations in serial EM sections of inp51/52 KO cells that spanned >300 nm in depth and were reminiscent of the cups or "vacuole-like" structures seen in fluorescence images of Fig. 3 in Singer-Krüger et al. In contrast, Nce102 or Pil1 positive structures in top-view STED images (Fig. 3G, 5B, 7F) were always linear, less than 50 nm wide and had increased intensities at both end, which perfectly fits the expected patterns for half-toroidal tubes but is incompatible with larger cup-like structures. We therefore think that the cups seen in EM are not connected to MCC/eisosomes. In addition, tube-like structures with only one end at the PM in medial STED sections could represent views of rotated toroidal tubes, as we exclusively found linear or curved Nce102/Pil1 positive structures in top view images. Finally, the tubular nature of invaginations in 5xΔ cells became very clear in the time series taken after PalmC treatment (Fig 7D, Movie S1), where whole tubes or individual tube ends were frequently laterally displaced along the surface.

Since much of the remaining data presented has been reported previously, we cannot recommend publication of the current version of the manuscript. In addition, we believe the interpretation of the data requires significant reconsideration.

We agree that our mechanistic model needed additional support and better discussion. But we disagree that the remaining data in our manuscript has been reported before. Two key additional findings are:

- The sub-domain organization of MCC/eisosomes – characterized by the two marker-tetraspanners Sur7 and Nce102 – was indeed suggested before but solid evidence for this was so far lacking.
- The rescue of the PIP2 overproduction effects on PM topology and overall cell growth by synthetic anchoring of Sur7 to MCC/eisosomes is surprising and indicates that major consequence of PIP2 unbalance in the PM is actually the displacement of Sur7.

In general, the quality and diversity of our imaging approaches provide a comprehensive view of the enigmatic fungal MCC/eisosome structures and a strong foundation for future work on the physiological role of MCC/eisosomes. We think that our study will therefore influence a wide range of research directions, such as on lipid homeostasis, cell wall biogenesis, cells stress response and nutrient transporter physiology. Finally, the opposing roles of BAR domains and tetraspanners in controlling membrane curvature provides a novel and widely applicable concept in membrane organization, irrespective of the underlying molecular mechanism.

Specific Comments:

Our main comment concerns the interpretation of the 5x knockout data. Much of the model in Figure 10 hinges on the phenotype observed upon deletion of all 5 Sur7 family members, which has previously been observed with EM in several papers (Alvarez et al. 2008, Singer-Krüger et al. 1998). In the latter paper, the tubular structures seem to form as a result of abnormally long plasma membrane invaginations upon deletion of Inp51 and Inp52 (Sjl1 and 2) that bend around back to the plasma membrane. The model the authors have proposed for the formation of their

similar observed structures (the transition from figure 10A to 10B) does not make sense to us. We do not understand how membrane topologies available in an eisosome allow for tubular structures to form by the partial closing of the top part of the membrane. The authors should provide detail on how they envision that each leaflet of each membrane interacts with the other to form these complex structures.

A major phenotype described in Singer-Krüger, 1998 is the formation of large cups (called "vacuolar-like invagination" in the publication) that are very likely not linked to MCC/eisosomes and do not contain Pil1 or Nce102, as these markers exclusively localize to thin tubes at the cell periphery in STED images (Fig. 3G, EV4D). The shown "infolding" in Fig. 3g of Singer-Krüger, 1998 is very similar to the large invaginations we saw in serial sections of 5xΔ cells (Fig. EV4C) and likely represents a section of a larger lamella that delineates such a cup. Importantly, the study by Singer-Krüger et al. focused on the Δinp51/52 strain and did not follow any MCC/eisosomes markers.

As detailed above we have now added several experiments to further support our suggested model for half-toroidal tube formation: 1) We now include freeze fracture EM images of circular tube ends in the *inp51/52* KO and show that this phenotype can be rescued by artificial tethering of Sur7 to Nce102 in MCC/eisosomes (Fig 6C). 2) We have added linear STED profiles for tubes in 5xΔ, Δinp51/52 and PalmC-treated cells that show the increased intensity at ends that indicates the half-toroidal shape (Fig 3G 5B, 7F). 3) We better emphasize the end to end distance of tubes that correlates with the length of MCC/eisosome at different levels of expression for the Seg1 scaffold (Fig 3B, EV3B, C). 4) We now include structural predictions showing that Sur7 tetraspanners prefer to localize in membranes with very high positive curvature. 5) We performed Monte Carlo simulations to explore the curvature effects of varying numbers of BAR domains and the addition of tetraspanners that stabilize positive curvature (Fig 8B-F). 6) Time series of Pil1 tubes in 5xKO cells treated with PalmC show lateral displacement of entire tubes or of individual tube ends along the cell periphery, consistent with the proposed half-toroidal morphology (Fig 7D, Movie S1).

Apart from this, if there are indeed two distinct mechanisms for formation of these tubular structures (the authors proposed method and the method proposed in Singer-Krüger et al.), the authors have no way of distinguishing them using the methods used in this study. In both the EM and superresolution data of the 5x knockout presented in this manuscript, we can see linear invaginations with only one opening (reminiscent of Figure 3 in Singer-Krüger et al.). We do not believe that the authors have provided sufficient evidence to establish a new model for how the observed invaginations form, which creates a fundamental issue with the interpretation of the data in the manuscript. (Singer-Krüger et al., 1998 JCS)

We indeed observe a variety of diversely-shaped PM invaginations in both the 5xKO and *inp51/52* KO cells. Without in vitro reconstitution (beyond the scope of this manuscript) we cannot be certain that the proposed formation of half-toroid tubes through pre-patterned BAR-domains is the only mechanism involved. We have therefore added images of different invagination types found in serial TEM images (Fig EV4), in freeze fracture preparations (Fig 3H) and STED (Fig 3G). As suggested by the reviewer we discuss alternative mechanisms for tube formation and for larger cup-like invaginations. We note that half-toroidal tubes are likely covered by a coat of BAR domain proteins and that MCC/eisosomes exclude typical markers of

endo- or exocytosis (Busto *et al*, 2018). We therefore find it unlikely that a tip growth-fusion mechanism can account for the prevalent toroidal topography. In addition, we find a remarkably conserved distance between the tube endings at the cell surface that corresponds to the expected length of MCC/eisosomes (this distance is increased by overexpression of the Seg1 scaffold). Such a correlation of tube and MCC/eisosome length is hard to explain by tube growth and fusion, which should lead to a more random distribution of tube ends. Finally, together with Andreas Heuer we performed Monte Carlo simulations of planar particle arrays to support our mechanistic model of PM deformation from a linear stretch of pre-patterned BAR domains (supposedly by the Seg1 scaffold). The observed membrane deformations recapitulate the proposed evolution from flat PM to half-toroidal tube (Fig. 8B-F and Methods). Importantly, we do not propose a transition from furrow to toroidal tube but rather that Sur7 tetraspanners block the evolution of invaginations such that stable furrows are formed with lateral edges that are optimal for the presence of Sur7, in keeping with its strong predicted curvature preference (PPM3.0 prediction, Fig. 8B-F).

In addition, the model proposed in Figure 10 hinges on the assumption that BAR proteins sculpt the membrane into tubular structures. While there is evidence that purified yeast BAR proteins can sculpt and tubulate membranes in vitro, as far as we are aware there is no direct in vivo evidence that this property holds true in the high turgor pressure environment of the yeast cytosol.

The biochemical proof for sculpting of membranes by the Lsp1/Pil1 BAR domains has indeed been obtained in vitro with artificial lipid bilayers (Karotki *et al*, 2011, Zhao *et al*, 2013). This is one reason we think that the localization of Pil1 and Nce102 within the half-toroidal tubes in our STED images provides important novel evidence for the equivalent in vivo function of the BAR domains. Remarkably, the diameter of tube ends closely resembles the diameter of in vitro-formed membrane tubes using the Lsp1 BAR domain. That Pil1 and Lsp1 are responsible for the formation of MCC/eisosome furrows - and can do this despite the high turgor pressure - is well documented. The exceptional force needed to bend the yeast PM might also be the reason that Pil1 is assembled onto a polymeric Seg1 scaffold, rather than directly binding to PIP2-enriched membrane surfaces. In addition, it has also been shown that Pil1 can bend membranes in mammalian cells (Olivera-Couto, Aguilar, 2011 MBoC).

We suggest that the authors look for distinct phenotypes in erg6Δ cells to support the claim that ergosterol enrichment on the upper edges of eisosomes has a function in localizing Sur7 family members. This claim cannot be supported by only showing colocalization of filipin and Sur7.

We did not intend to claim a direct role for ergosterol in recruiting Sur7 to MCC/eisosome edges. We mainly wanted to provide a reference radial position for typical PM markers that have previously been localized to MCC/eisosomes, including the filipin dye presumably specific for ergosterol. While a role for ergosterol, and possibly asymmetrically distributed sterols, in driving or supporting local membrane topography is very attractive, this will have to be addressed in a future study. We have now further toned down our claims on lipid function beyond and removed the localization profiles of filipin in 5xΔ cells as this did not directly contribute to the main story.

The authors should also provide more support for their "distance between peaks" metric. It seems that this metric relies on cells being exactly in the medial focal plane, and that any slight deviation of the focal plane down or up could cause significant differences in the observed distance between peaks depending on the orientation of the eisosome invagination. For example, the data for Pil1 and Sur7 in both figure 1E and figure 5B are noticeably different.

We now provide a more detailed description of radial distance measurements in the methods section. Briefly, we found that close to the medial focus plane our measurements were quite insensitive to slight focus shifts up or down. We have extensively validated our approach using various positive and negative controls and found remarkably consistent distances between all marker pairs measured (with measurements by different experimenters, on different days or with different strains). We were however very grateful for this comment: While revisiting our data we found that in our analysis routine the mathematical sign of our distance measurements was accidentally omitted, which mainly affected pairs of markers with small or no distance. With the correct operations control, values of colocalizing markers now all distribute around 0 (new Fig. 1F, 6B, 7E).

**Referee #3:**

Haase et al. report the super-resolution microscopy-based sub-domain organization / structure of a particular membrane domain of the yeast plasma membrane (PM), the MCC/eisosome, which forms furrow-like invaginations. Their findings identify, for the first time, structural roles of two families of tetraspanners, Sur7 and Nce102, in the assembly of the invagination. Sur7 members are located at an ergosterol-enriched subdomain at the upper edge of the invagination and somehow counteract the tubulation induced by the BAR-domain proteins Pil1 and Lsp1, thereby preventing closure of the furrow into a tubule. Nce102, on the contrary, is localized deeper in the furrow and is required for Pil1 activity, which requires PIP2.

The results are novel and reveal a non-homogeneous organization of the eisosome domain at the sub-domain level. The authors show that this sub-domain organization is critical for the proper formation/assembly of the domain into characteristic furrow-like invaginations, and consequently for the physiological function of MCCs in sphingolipid signalling and cell-wall integrity. Eisosomes are conserved in fungi and important for pathogenicity, so this work will be most interesting for the fungal cell biology and pathogenicity communities. In addition, such a nanoscale organization could give important insight into the organization of similar tetraspan proteins in mammalian membrane domains, like claudins and occludins in tight junctions.

The manuscript is in general well-written and clear, and the results for the subdomain organization are strong and convincing. A general feeling of this reviewer though, reflected also in the comments below, is that as the story progresses the manuscript is less developed (from results describing Figure 5 and below) and the text progressively becomes less accurate and more difficult to follow. I would suggest a revision of this second part of the results section, to make it precise and clear, as is the first part.

In addition, and especially in this second part of the results, sometimes control experiments are missing, or interpretations drawn are not fully supported by the data presented, analysed below:

**Major comments.**

A first category of comments is about the role of PIP2. Despite the results presented, the role of PIP2 in MCC assembly remains poorly understood. Some of the proposals below could shed more light on this relationship:

In Figure 7F, is it possible to additionally monitor Mss4 localization without overexpression?

We have now replaced the overexpression data with results showing that endogenous Mss4 localizes to PM clusters that are segregated from MCC/eisosome (Fig. 5H). These clusters are dynamic (Ling et al., 2012, EmboJ) and did not localize to the half-toroidal tubes labeled by Nce102 in 5xΔ cells (Fig. 5H).

Surprisingly, as shown in our original figure, overexpressed Mss4 was absent from the PM in 5xΔ cells. A possible explanation for this finding is that Mss4 recruitment to the PM requires PI(4)P (Ling et al., 2012, EmboJ) and that this substrate is depleted when Mss4 is overexpressed and Inp51 function is reduced due to the altered MCC/eisosome organization (tubes instead of furrows, Fröhlich et al., 2012, MBoC). In addition, Mss4 recruitment is inhibited by the PH-domain protein Opy1 (Ling et al., 2012, EmboJ) and overexpression of Mss4 could increase this inhibitory interaction. Importantly, the amount of accessible PIP2 (by the PLCδ PH domain) in

the PM is clearly reduced upon deletion of Sur7 (Fig. 5F). As the Mss4 overexpression results were difficult to interpret, we decided to keep them out of the manuscript and only report the localization of endogenous Mss4, which is not affected by Sur7 deletion at all.

Because in Figure 7A it is shown that Mss4 overexpression leads to tubulation, suggesting that Sur7 members are already misplaced from Pil1 and Nce102-containing tubulated eisosomes, similarly to what is shown for the Δinp51/52 mutant in Figure 7C. If this is the case, how does deletion of SUR7 members affect the membrane recruitment of Mss4? A potential explanation could be that, given that the effects of Mss4OE and 5xΔ on membrane tubulation are additive, non-overexpressed Mss4 remains normally at the PM in the 5xΔ strain. Alternative explanations are also welcome.

We indeed now show that endogenously tagged Mss4 remains at the PM in 5xΔ cells (Fig. 5H). In addition we show that in contrast to the close overlap of Pil1 and Inp51 in MCC/eisosomes of WT cells, Inp51 no longer localizes to Pil1-covered tubes of 5xΔ cells (Fig. 5G), possibly leading to uncontrolled phosphatase activity. The consequence of this would be excess PIP2 within tubes (and increased BAR domain recruitment) and decreased PIP2 levels in the rest of the PM (possibly leading to displacement of Sur7).

2. Lines 28-29, 98-99 and elsewhere. To which evidence do the authors base their claim that that PIP2 is found at the bottom of the furrows? Apart from previously published results that PIP2 is required for tubulation by Pil1 and Lsp1, I could not find any data within this manuscript. In Figure 7G, no labelling of 2xPH(PLCδ)-GFP to the characteristic Pil1-containing tubules of the 5xΔ strain is evident, suggesting that either these tubules do not contain any PIP2, or that PIP2 is not accessible to 2xPH(PLCδ)-GFP, for example due to binding by Pil1 and Lsp1. Moreover, overexpression of PIP2 affects Sur7 positioning, which is found at the upper part of the invagination. Importantly, Inp51 in Figure 7E seems to mostly localize to the upper part of the invagination (similarly to Sur7) and not close to the Nce102-containing tubules of the 5xΔ strain, yet, no radial distance measurements are provided. The same applies to Mss4OE in Figure 7F: it seems to localize mostly to the upper part of eisosomes. These results potentially suggest that PIP2 biosynthesis and degradation mainly occur at the upper part of the eisosome. I suggest the authors to either provide evidence for an increased PIP2 concentration to the lower part of eisosomes, or rephrase their claims and include a dedicated section in the discussion about the potential localization of PIP2. This discussion could include the arguments above and alternative explanations, considering also the possibility that PIP2 is homogeneous within the whole eisosomal invagination. Additional experiments to address some of the points raised above (e.g. about the subdomain distribution of Mss4 or Inp51) would also be welcome but not essential.

We thank the reviewer for this very important point. We indeed think that PIP2 is critical for Pil1/Lsp1-mediated bending of the PM in MCC/eisosomes or tubes but that the BAR domains sequester the lipid from cytosolic binding probes such as the PH domain (shown for Lsp1 by Zhao et al. 2013). We now include results showing that endogenous Mss4 is not localized to MCC/eisosomes and remains at the PM in 5xΔ cells (Fig 5H). We also found that Inp51 localizes to the bottom of furrows together with Nce102 and Pil1, consistent with previously shown biochemical interactions (Fröhlich et al., 2012, MBoC). In 5xΔ cells Inp51 was still present in dynamic clusters at the PM, but now found preferentially at the upper edge of tubes marked by

Nce102 (Fig. 5G). We cannot currently completely distinguish between local effects of Inp51 in regulating Pil1 function and PM topography and more general homeostatic effects on PIP levels and the physical properties of the PM. We have adapted our discussion to better cover these aspects (p14).

3. If the levels of PIP2 at the PM are reduced in the 5xΔ, how come tubulation by Pil1 (which requires PIP2) is more efficient? Could the results in Figure 7G be, somehow, the outcome of increased tubules within the cytoplasm? The authors could discuss such this.

As described above we find that Inp51 is uncoupled from Pil1 in the 5xΔ mutant and no longer localizes to the Pil1-decorated tubes (Fig 5G). If Inp51 activity remains unaffected this would lead to a reduced activity and increased PIP2 levels within tubes (explaining the increased activity of Pil1) and simultaneously higher activity outside of tubes and the reduction of PIP2 within the rest of the PM. We have now included these aspects in our discussion (p14).

4. Results in Figure 8 are most interesting and suggest a role of PIP2 in the misplacement of Sur7 upon reduction of PM tension. The authors show that PIP2 (using 2xPH-PLCδ) clustering under these conditions is closely associated (but no high-resolution colocalization is provided) with Nce102, and that Sur7 is misplaced compared to Pil1. Yet, the authors do not directly investigate the spatiotemporal relationship between Sur7 and PIP2. Such an investigation would show whether PIP2 clusters are more associated to Pil1 or to the misplaced Sur7 upon acute reduction of membrane tension.

We were unfortunately not successful in visualizing PIP2 via STED due to the rapid motion of the probe during the STED scanning period. Instead, we have now included image series (Fig 7D, Movie S1) demonstrating rapid lateral displacement of entire MCC/eisosomes or PM-linked tubes within seconds of PalmC addition. This indicates a large-scale reorganization likely through disruption of extensive gel domains within the PM. In contrast, PIP2 clusters formed rapidly after addition of PalmC but did not move laterally. Unfortunately, we could not determine whether the initiation of mobility for furrows or tubes was linked to local PIP2 accumulation - and possibly uncoupling of Sur7 strands from furrows - but this would definitely be an exciting challenge for future studies.

Moreover, the authors could directly investigate whether tubulation is formed under these conditions, for example by determining with STED microscopy the radial localization of Pil1 upon PalmC treatment.

As suggested, we have now performed STED microscopy of Pil1 after PalmC treatment for 10 min. Compared to the typical short and straight furrows of control cells, we observed bent structures at the surface of cell and increased intensities at the ends of linear structures that were reminiscent of the half-toroidal tubes seen in 5xΔ cells (Fig. 7F). In addition, the radial distance between Pil1 and Sur7 was significantly increased in PalmC treated cell, albeit to a lesser extent than seen in 5xΔ or Δinp51/51 cells (Fig. 7E).

In addition, since MCCs are stable and static over long time-periods, it would be interesting to investigate, using time-lapse microscopy, which eisosome components (if any) remain static and

which are missplaced after PalmC treatment. In other words, is it Sur7 or Pil1 (or neither?) that remain at the same position after PalmC treatment, and is this somehow related to PIP2 distribution? These results could give important mechanistic insights, potentially also coupled with suggestion 3 (see also major comments 7 and 9).

To address this point we have now followed the effects of PalmC treatment using live cell epifluorescence microscopy. We found that within seconds of PalmC addition individual MCC/eisosomes started to rapidly move across the entire cell periphery (Fig 7D). Considering the typical stability of MCC/eisosomes over several hours this was a very surprising finding and indicated that PM "softening" with PalmC released MCC/eisosomes from some sort of anchor. We found similar movement of entire Nce102-labeled tubes or single tube ends in 5xΔ or Δinp51/52 cells (Fig. 7D), consistent with our proposed model of half-toroidal tubes. We were not able to obtain similar movies using Sur7-GFP due to practical issues with much lower signal levels and prevalent bleaching. However, the displacement of Sur7 from MCC/eisosome edges – and potentially the resulting tube formation – is likely a result of the large scale reorganization of the PM upon PalmC addition.

**Some other major comments:**

5. Line 237. It is not clear to this reviewer what the authors mean by saying that the "Sur7 C-terminus was not required for strand formation per se". In Figure 4E it is indeed clear that in the wt, Sur7ΔC is normally recruited to eisosomes, but localization at the edges of both strands of the invagination is not as clear as shown for wt Sur7 in Figure 2.

We now included a zoomed image of Sur7ΔC localizing to double strands in the wt background (Fig 4E).

Moreover, in the 5xΔ strain, curved invaginations and tubules are mostly evident, as expected since Sur7ΔC cannot rescue membrane tubulation. Consequently, the argument that the c-terminus is not required for strand formation is not clear from the data presented. To strengthen such a statement, the authors could provide micrographs similar to Figure 2, showing that Sur7ΔC is recruited to both strands of the invagination in the wt, or to both strands of curved invaginations in the 5xΔ mutant, along with measurements of the intensity profile. In case the authors intended to say something different, please rephrase.

We mainly intended to state that the deletion of the C-terminus did not abolish correct recruitment to MCC/eisosomes and that we still observed elongated structures that indicated oligomerization of Sur7ΔC on the PM. We expect the curved Sur7ΔC structures to only partially colocalize with the tips of the half-toroidal PM tubes. We have now rephrased this part (p8, line 264-265).

6. Results presented in Figure 5E and 5F are most interesting, but incomplete. The results indicate that Nce102 has a structural role in MCC assembly that goes beyond its previously described role in regulating Pil1 phosphorylation. The main data supporting this is that Pil1-4A expression rescues defective MCC formation and tubulation in the 6xΔ strain, yet these tubules are shorter when compared to the 5xΔ strain expressing wt Pil1. Please notice that the two strains compared have two differences: expression of Pil1 or Pil1-4A and presence or absence of NCE102. In order to define the contribution of Nce102 and Pil1-4A in the phenotype described,

an important control is missing. A 5xΔ strain expressing Pil1-4A-Halo instead of Pil1-Halo. Importantly, the authors already show that, in the absence of Nce102, the distance between Pil1-4A and Sur7 is shorter than the one of Pil1 and Sur7 in the few remaining eisosomes (Figure 5B). This could potentially suggest that Pil1-4A is somehow less efficient that wt Pil1 for membrane tubulation in the absence of Nce102. Alternatively, in the absence of Nce102 (and due to unknown reasons) when Pil1-4A is expressed an increased number of shallower MCCs could be formed instead of a smaller number of normal-depth eisosomes. An additional control would be a Pil1-4A expressing wt strain. These controls could clarify whether the shorter tubes observed in 6xΔ Pil1-4A are due to the absence of Nce102 or defective Pil1-4A tubulating activity, or a combination of both.

We indeed think that Nce102 plays a structural role beyond its function in Pil1 phosphorylation. However, considering the extensive revision and additional data now included, we also felt that this dataset was rather distracting from our main focus on the interactions of Sur7, PIP2 and Pil1. We therefore decided to completely remove experiments on the function of Nce102. We only kept control experiments with Nce102 that show that it is exclusively located at the base of MCC/eisosomes (Fig. 1F, 2A), and that it is not required for furrow or double strand formation per se (Fig. 3F). For future studies on the exact role of Nce102 we have generate the control strains indicated by the reviewer but have not yet performed the required STED imaging due to time constraints.

7. Lines 92-93, 377-379, 403-404. The arguments presented by the authors that Sur7 members "counteract the forces exerted by BAR domain proteins at the furrow base" are not strong. This reviewer agrees that Sur7 members seem to somehow modulate/diminish the tubulating function of the BAR domain proteins, but the underlying molecular mechanism has not been elucidated. The mechanisms could, thus, be others not related to "counteracting the forces", like for example a direct or indirect regulation of Pil1 and Lsp1 function, or an anchoring role (via contacts with the cell wall? See also major comment 9 and suggestion 3) of the invaginations to the plasma membrane. The authors could provide additional evidence in support of their claims, or rephrase the text to include alternative explanations for their results.

We absolutely agree with the reviewer and did not intend to claim that Sur7 acts on the PM through direct mechanical forces. We rather think that a combination of different but highly localized molecular mechanisms including cell wall anchorage, membrane curvature, lipid composition and protein oligomerization finally result in the “counteracting forces” that establish the local PM topography. The formation of large protein oligomers indicate that these complexes might directly influence membrane curvature but this is clearly not unambiguously shown by our in vivo data. We have now replaced the term “counteract” by “modulate” to make this clearer (for example title, abstract and line 92).

8. The idea of fusing Sur7 to Nce102 in an attempt to tether Sur7 to MCCs and rescue eisosome tubulation in the Δinp51/52 strain is excellent, and the results very promising. Yet, a few important controls are missing, since the results presented could have an alternative interpretation: The fusion, instead of tethering Sur7 to MCCs, could misplace Nce102 too out of MCCs. In the experiments presented, this cannot be ruled out since the MCC localization of the chimeric protein under all conditions has not been examined. More specifically:

a) In Figure 9A the authors measure distance from the plasma membrane (and not from MCCs as usually in other figures, but that is not feasible here indeed) of Sur7, Nce102, and of the chimeric protein. Data show that the latter is closer to the plasma membrane, and the authors interpret this as a rescue of the tubulation phenotypes. Yet, since there is no marker of the upper or lower parts of eisosomes, it is possible that tubulation is not rescued and it is only the chimera that is found closer to the plasma membrane, either in the upper part of eisosomes or even outside eisosomes (especially for the Δinp51/52 strain, as shown for Sur7 in Figures 7C and 9C). I propose the authors to repeat the experiment with a non-fluorescently-tagged Nce102-Sur7 chimera and check the distance of Pil1 (as a marker of tubule formation) from the plasma membrane under the same conditions, for determining membrane tubulation.

We thank the reviewer for this suggestion. To address the possible displacement of Nce102 (instead of Sur7) we have now performed the radial and lateral localization experiments using Pil1-RFP as reference instead of Cellmask Orange (the latter was now completely removed to make assays more comparable). Pil1 clearly defines the base of either MCC/eisosome furrows or toroidal tubes. As can be seen in Fig. 6B endogenous Nce102 and Pil1 remain closely associated, even in the tubes of Δinp51/52 cells. In contrast, both Sur7 and the Nce102-Sur7 chimera exhibit the typical distance of 50-100 nm from Pil1 seen in furrows (in wt and in rescued Δinp51/52 cells). Note the much larger distance of several 100 nm between Sur7 and Pil1 in the tubes of mutant cells. We have also added a new panel showing the ends of half toroidal tubes in freeze fracture images of Δinp51/52 cells and the rescue of this effect by the Nce102-Sur7 chimera (Fig. 6C)

b) The same alternative explanation could be valid for Figure 9C. The fact that the Nce102-Sur7 chimera colocalizes with Sur7 in the Δinp51/52 strain does not necessarily mean that this colocalization occurs in eisosomes. The degree of colocalization of the chimeric protein with Pil1 (or Nce102) in both the wt and the Δinp51/52 strains should be provided.

We now provide the comparison asked by the reviewer (Fig. 6A). It can be clearly seen that Nce102 and the chimera always colocalize with Pil1, while Sur7 is laterally displaced in the in Δinp51/52 cells.

9. Line 381-382. The data presented by the authors are in agreement with Sur7 members forming parallel strands along the longitudinal axis at the upper edges of MCC/eisosome furrows, and this reviewer agrees that this is a very likely organization. Yet, the high-resolution molecular structure of these strands is missing and alternative modes of organization could be envisaged. For example, are these single strands or many parallel strands? Are the Sur7 localized to the curved membrane (at the edge of the plasma membrane and the invagination), to the inner upper part of the invagination, or to the neighboring, flat part of the plasma membrane (outside to the invagination, but in close contact with it), or combinations of the previous? Such types of organization could potentially suggest mechanistic insights into how Sur7 members, via their C-termini, inhibit Pil1 tubulation, e.g. via anchoring to the cell wall (See also major comment 7 and suggestion 3). In the absence of experimental evidence or data from the literature (about the topology of other tetraspanners as well?), I invite the authors to present a model that is as broad as possible.

These are very good suggestions and we modified our discussion to illustrate the different mechanistic options (line 450-462). We find it unlikely that the C-terminus acts through

interaction with the cell wall as it is oriented towards the cytosolic side. One possibility is that the C-terminus influences the interaction (affinity, orientation) within the oligomeric strands. While self-interaction of Sur7ΔC was still detectable in co-IP experiments (Fig 4F) we cannot know whether this interaction is direct or weaker than for the full length protein. In vitro experiments with purified components should be able to address this point in the future. Regarding the exact position of the Sur7 tetraspanners: we have now added structural predictions using the PPM3.0 server that show a preference of Sur7 for very high positive curvature, while Nce102 prefers flat membranes (Fig 2E).

10. In figure 9D, it is shown that the Nce102-Sur7 chimeric protein cannot rescue hypersensitivity to CW of the 5xΔ mutant. In this growth test, expression of Sur7 alone is not shown, which would be expected to rescue the phenotype. If this is the case, would this mean that the chimeric protein rescues only the Nce102-dependent phenotypes (sphingolipid signaling) and not Sur7-phenotypes? It would be nice to have such a control in the growth test. Moreover it would be nice to know whether the increased resistance of the 5xΔ to PHS could be reversed by expression of the Nce102-Sur7 chimera.

We thank the reviewer for this observation. We now reorganized the manuscript to better focus on the physiological implications of our findings. In the main figure (now Fig 6D) we only show the results from the rescue of the inp51/52 deletion and also added results with heat stress showing a similar effect. All growth reductions upon stress are rescued by expression of the chimera. The growth assays with the Sur7 KO are now in Fig. EV5C. We show that while AbA sensitivity is increased if at least 3 of the 5 Sur7 family members are removed, CW sensitivity specifically depends on Tos7, which was also recently reported (Zhu et al., 2020, Fungal Genet Biol 144: 103467). As the Nce102-Sur7 chimera cannot substitute for the absence of Tos7, the failed rescue is therefore expected. To avoid unnecessary confusion in our arguments we decided to remove all chimera-rescue experiments with the 5xΔ strain.

**Minor comments**

1. Line 72. This reviewer agrees that the MCCs/eisosomes are characteristic, stable invaginations of the yeast plasma membrane, but the statement that the yeast membrane is otherwise flat could be misleading. In fact, there are also sites of endocytosis, which correspond to smaller and dynamic, transiently-formed invaginations with different size. The sentence could be rephrased to exclude misconceptions.

We removed the questionable part, the sentence now reads "characteristic half-pipe shaped furrow with that marks this domain as a unique topographic environment within the yeast PM"

2. Line 95. Can it be excluded from current and previous evidence that Nce102 somehow structurally supports the formation of deeper invaginations, without promoting BAR domain-induced curvature?

We currently have no mechanistic evidence for the actual function of Nce102 but find it highly unlikely that it acts by directly interfering with Pil1 phosphorylation. It is much more likely that the high concentration of Nce102 changes local PM properties and indirectly supports assembly of Pil1 coats or the establishment of specific curvature. As we cannot pinpoint a specific function for Nce102, we have now removed most data on Nce102 deletion from the manuscript and

instead focus exclusively on the role of Sur7 tetraspanners and their interaction with PIP2 and Pil1.

3. Lines 108-109, please clarify that A08184g was heterologously expressed in S. cerevisiae and the results are not obtained in K. lactis cells.

This was amended.

4. Please correct the positioning of the namings in EV1C, so that everything is visible.

This was now corrected

5. Line 346. "found" could be replaced by "verified" or "confirmed".

We replaced it with “could identify” (line 311)

6. The radial distance of Pil1 and Sur7 in the micrographs of Figure 5A is not as clear as in Figure 1, although measurements are similar. The authors could show a more representative micrograph here.

Data on ΔNce102 cells was mostly removed from the new manuscript.

7. Figure 8D. micrographs for the radial distance of Pil1 and Lsp1 are missing.

This was now corrected (Fig. 7E)

8. Line 425-426. Do these results refer to conditions of the Pil1-4A-expressing strains? Please specify.

Results on Nce102 deletion and Pil4A were removed from the current manuscript to better focus the story. See above for more details.

9. Line 831. Quantifications refer to F and not E.

This was corrected.

**Suggestions**

1. A previous publication has analyzed the periprotein lipidome of several eisosomal proteins, including Sur7 (van 't Klooster et al., 2020, eLife). The authors have reported that the periprotein lipidome of Sur7 is not enriched in ergosterol, data that seem to not agree with the data presented by the authors in the current manuscript. The authors could try to include this previous work in the discussion and speculate a bit about the reasons of potential differences.

The cited study did indeed find that several yeast PM proteins extracted into SMALPs (small lipid particles generated by using styrene-maleic-acid as limiting polymers) exhibited very comparable lipid contents and did not show sterol or sphingolipid enrichment. One possible explanation for their findings (which are in contradiction to many studies on lipid distribution in the yeast PM) is that the bilayer asymmetry characteristic for the fungal PM is not maintained during the SMALP preparation and that the lipid composition of the purified particles therefore no longer corresponds to the natural environment. Without achieving realistic asymmetry as well as SL and sterol contents in their preparations we think that interpretations will be very difficult. We have now toned down all lipid-related interpretations in the manuscript except for the effects of PIP2, in order to better focus our story.

2. Regarding the effect of AbA on the accumulation of Sur7 in MCCs, it has previously been reported that depletion of complex sphingolipids leads to eisosome disassembly (Fröhlich et al. 2009). Given that Sur7 strand partitioning is not affected, could the reduced MCC partitioning of Sur7 be due to deformation of eisosomes? Have the authors tried to do the same experiment in a Pil1-4A expressing strain, in which MCCs are resistance to complex sphingolipid depletion-induced disassembly (Fröhlich et al., 2009)? This should be easy, but I understand that it is not critical, since the authors do not claim an important role of sphingolipids for MCC partitioning of Sur7.

The exact role of SL in MCC/eisosome organization is an interesting question but difficult to assess. We limited our microscopic analysis to cells that were treated with AbA for only 1h. This treatment leads to a block of complex SL synthesis but does not deplete the cellular SL pool. We have characterized the effects on the whole cell lipidome and mainly found a shift from MIP2C to IPC with few secondary adaptations. The accumulation of IPC is likely toxic to cells, which is the primary effect of shorter AbA treatment. Downstream effects of longer AbA treatments with actual SL depletion will include lipid metabolism adaptations and are therefore difficult to interpret and we have so tried to avoid such setups.

3. Young et al. 2002 have reported a change in typical PM distribution of Sur7 upon digestion of the cell wall. This result was a matter of debate in subsequent publications (Malinska et al, 2004, Spira et al., 2012). In the current manuscript, the authors find a very interesting but poorly understood relationship between PM tension, PIP2 distribution and Sur7 MCC partitioning, which directs the formation of static and stable MCC furrows at the plasma membrane. A potential mechanism via which Sur7 proteins would counteract the tubulation of Pil1 could be via anchoring to the cell wall. It would be interesting to test this idea in light of the current findings. Some ideas would be to check PIP2 distribution, Sur7 and Pil1 colocalization in protoplasts, and whether Pil1-containing tubules are formed in protoplasts. Yet, this is only a suggestion.

This is a very interesting suggestion and we have indeed observed large changes in MCC/eisosome morphology/distribution upon removal of CW. A major phenotype we observe is that in protoplasts individual MCC/eisosome furrows align end to end and form connected rows of furrows where the two Sur7 strands fuse to continuous structure but the Pil1 intensities are periodic. This is a very interesting direction to pursue but not related to the main points we want to make in the current story. We would therefore prefer to keep this data out of the manuscript and expand on it in a future study. Importantly, MCC/eisosome furrows in protoplast do not turn into tubes, arguing against an essential role of the CW for preventing tubulation. However, this does not preclude a supporting role.

4. The relationship of sterols and Sur7 seems most interesting but not analyzed in detail in the manuscript. The authors could potentially want to check if sterols are required for the proper MCC localization of Sur7 members, e.g. by depleting ergosterol using a conditional sterol biosynthesis mutant. Additionally, the authors could determine the localization of filipin under conditions of PIP2 overexpression: Will it still segregate with Pil1-eisosomes, or with the Sur7 outside MCCs? This reviewer understands well that this could be another story though.

We agree that the localization of filipin signal at the upper edge of MCC/eisosome furrows is very interesting and we definitely plan to study this further in the future. However, we have so far not found any effects of ergosterol depletion (different mutants) on Sur7 localization and did

not want to imply a key role of sterols in curvature generation. We have now only reported the localization of fillipin to the upper edge of WT MCC/eisosomes and removed all data on sterol distribution in 5xΔ cells as we cannot make any claims about actual function of sterols in this context and did not want to distract from our main focus on the interactions of PIP2 and Sur7 tetraspanners.

Dear Roland,

Thank you for the submission of your revised manuscript to EMBO reports. I am sorry for the delay in handling your manuscript. Unfortunately former referee #2 was not available anymore, but we have received the reports from referee #1 and former referee #3 (now #2). I have already informed you about the reports and appreciate your point-by-point response to these. As discussed, the remaining concerns from referee #2 can be addressed by clarification. The reports are again copied below my signature.

From the editorial side, there are also a few things that we need before we can proceed with the official acceptance of your study.

- Please reduce the number of keywords to 5.

- Please provide a 'Disclosure and competing interests statement'. For more information see https://www.embopress.org/page/journal/14693178/authorguide#conflictsofinterest

- Please remove the Author Contributions from the manuscript file and make sure that the author contributions in our online submission system are correct and up-to-date. The information you specified in the system will be automatically retrieved and typeset into the article. You can enter additional information in the free text box provided, if you wish.

- Per our editorial policies, all statements need to be supported by data. In this respect, we note two cases of 'data not shown on page 12 and page 15. Please either supply the data you refer to or remove/rephrase the statement.

- Table EV1 should be uploaded as Dataset and named Dataset EV1. Please provide a short legend in the first tab and correct the callouts in the manuscript.

- Movies: the legends should be removed from the manuscript file. Each legend (provided as a README.txt file) should be zipped with its corresponding video and the zip files uploaded separately.

- The Acknowledgments should go after the Data Availability section

- When you describe in the figure legends how many cells or eisosomes you measured, please also include information from how many independent experiments these were derived.

- I attach to this email a related manuscript file with comments by our data editors. Please address all comments and upload a revised file with tracked changes with your final manuscript submission.

- We already discussed about the reuse of an image from Figure 2D in Fig. EV3B (Seg1 OE) and of an image from Fig. 5E in Fig. EV2C (WT control). As discussed please either replace these images with alternative ones or state the duplication clearly in the respective figure legends.

- Source data:
1) I did a spot check on the source data for Fig 2A and noticed that I could only open the images in Fiji. When I opened them in either Preview or Adobe Photoshop they appeared grey. The same was true for the STED image from Fig 2B or the images from 2C etc. I wonder what the reason for this is, since they are all .tif images?

2) In the source data checklist you refer to S# figures which should be updated to the correct EV figure number. We also need the source data for the EV figures, which is currently still missing.

- Finally, EMBO Reports papers are accompanied online by A) a short (1-2 sentences) summary of the findings and their significance, B) 2-3 bullet points highlighting key results and C) a synopsis image that is 550x300-600 pixels large (width x height) in PNG for JPG format. You can either show a model or key data in the synopsis image. Please note that the size is rather small and that text needs to be readable at the final size. Please send us this information along with the revised manuscript.

- On a different note, I would like to alert you that EMBO Press offers a new format for a video-synopsis of work published with us, which essentially is a short, author-generated film explaining the core findings in hand drawings, and, as we believe, can be very useful to increase visibility of the work. This has proven to offer a nice opportunity for exposure i.p. for the first author(s) of the study. Please see the following link for representative examples and their integration into the article web page:

https://www.embopress.org/video_synopses
https://www.embopress.org/doi/full/10.15252/embj.2019103932

Please let me know, should you be interested to engage in commissioning a similar video synopsis for your work. According operation instructions are available and intuitive.

We look forward to seeing a final version of your manuscript as soon as possible.

With kind regards,

Martina

Martina Rembold, PhD
Senior Editor
EMBO reports

***********************

Referee #1:

Haase and collaborators have done an excellent job in addressing all my comments, and the comments of the other reviewers. I find that the revised version of the manuscript is greatly improved. The new data on the role of PIP2 in eisosome assembly and the recruitment of Sur7 proteins, and especially the simulations for the formation of invaginations, provide a convincing mechanistic insight into how the subdomain organisation of eisosomes could be regulated by and control PIPI2 levels. The latter is particularly important, since it suggests a mechanism by which eisosomes could be regulated in response to changes in membrane tension. Importantly, the authors additionally provide convincing evidence that the toroidal tubes formed in the absence of the Sur7 or upon increase in PIP2 levels are different from the previously-reported large, cell-walll filled invaginations. I, consequently, suggest immediate acceptance of the manuscript for publication.

minor correction at line 239: correct to "Nce102-positive".

Referee #2:

Haase et al. showed the importance of Sur7 family tetraspanner proteins in MCC/eisosome formation. It is also significant that they showed the relationship between Sur7 and PIP2 in MCC/eisosome. The data are presented using TIRFM, STED, and freeze fracture EM, and are examined from multiple perspectives, including genetic analysis using various deletion mutants, pharmacological analysis, and analysis using simulations.
Although the half-toroidal tubes structure the authors proposed has not been completely demonstrated, it is a reasonable model structure that could be assumed. Further analyzes are expected to reveal the details of this structure. The manuscript is well-written, and the data is convincing. However, some of the figures are not easily understood, and some explanations are lacking. Below are comments, including suggestions.

・Although Sur7 ΔN and ΔC are used in the experiment of figure4, the authors do not show the domain structure of Sur7 ΔN and ΔC. It is better to show the domain structure of ΔN and ΔC in figure4B.

・It is better to describe the discussion of Sur7 not being able to localize properly when the amount of PIP2 is increased (or decreased).

・The authors assume that Nce102 localizes to the lower part of the furrow and has no curvature preference, but Sur7 localizes to the upper furrow edges and has a positive curvature preference. The authors do not provide the explanation of why the phenotype was rescued by expressing Nce102-Sur7 chimera, it would be better to describe the explanation. Also, what about PIP2 levels?

・In Figure 3H freeze fracture EM, it would be better to show the distribution of the structures in wild type sample. The authors describe that the structures of 5xΔ and Δinp51/52 mutants are similar in fluorescence-based experiments, but the population of structures of 5xΔ (Figure 3H) and Δinp51/52 (Figure 6C) in freeze fracture EM seem to differ significantly. How do the authors explain this point?

・The effects of PIP2 levels and other phospholipids have been examined, but what about ergosterol levels in deficient mutants involved in furrow formation, such as 5xΔmutants?

・

We thank both reviewers for their positive evaluation. Below are brief replies (in blue) to the remaining comments:

**Referee #1**

Haase and collaborators have done an excellent job in addressing all my comments, and the comments of the other reviewers. I find that the revised version of the manuscript is greatly improved. The new data on the role of PIP2 in eisosome assembly and the recruitment of Sur7 proteins, and especially the simulations for the formation of invaginations, provide a convincing mechanistic insight into how the subdomain organisation of eisosomes could be regulated by and control PIPI2 levels. The latter is particularly important, since it suggests a mechanism by which eisosomes could be regulated in response to changes in membrane tension. Importantly, the authors additionally provide convincing evidence that the toroidal tubes formed in the absence of the Sur7 or upon increase in PIP2 levels are different from the previously-reported large, cell-wall filled invaginations. I, consequently, suggest immediate acceptance of the manuscript for publication.

minor correction at line 239: correct to "Nce102-positive".

done

**Referee #2**

Haase et al. showed the importance of Sur7 family tetraspanner proteins in MCC/eisosome formation. It is also significant that they showed the relationship between Sur7 and PIP2 in MCC/eisosome. The data are presented using TIRFM, STED, and freeze fracture EM, and are examined from multiple perspectives, including genetic analysis using various deletion mutants, pharmacological analysis, and analysis using simulations.

Although the half-toroidal tubes structure the authors proposed has not been completely demonstrated, it is a reasonable model structure that could be assumed. Further analyzes are expected to reveal the details of this structure. The manuscript is well-written, and the data is convincing. However, some of the figures are not easily understood, and some explanations are lacking. Below are comments, including suggestions.

- Although Sur7 ΔN and ΔC are used in the experiment of figure4, the authors do not show the domain structure of Sur7 ΔN and ΔC. It is better to show the domain structure of ΔN and ΔC in figure4B.

We have now added a short link in the text to the relevant regions of the schematics: line 259/260: "Sur7 variants lacking N- and/or C-terminal regions (light grey intracellular ends in Fig 4B)"

- It is better to describe the discussion of Sur7 not being able to localize properly when the amount of PIP2 is increased (or decreased).

It's not completely clear to us, what the reviewer wants us to change: The discussion currently contains a dedicated paragraph that details the effects on Sur7 distribution that we observe in cells with increased PIP2 levels (lines 498-503 in attached Main text).

- The authors assume that Nce102 localizes to the lower part of the furrow and has no curvature preference, but Sur7 localizes to the upper furrow edges and has a positive curvature preference. The authors do not provide the explanation of why the phenotype was rescued by expressing Nce102-Sur7 chimera, it would be better to describe the explanation. Also, what about PIP2 levels?

While we find clear radial separation of the intensity maxima for Nce102 and Sur7, the low optical resolution does not allow us to exclude overlap between both proteins. In particular, we would expect Nce102 to be in the flatter stretches of the furrow that directly touch the Sur7-domain. Of note, the preference for flat membranes is also consistent with the presence of significant Nce102 levels outside of MCC/eisosomes (see Fig 1C, higher network factor for Nce102).

The chimera is expressed under the Nce102 promoter (endogenous integration), which likely leads to 2-3x overexpression compared to endogenous Sur7 levels. Also, the radial peak of the chimera is close to that expected for Sur7 (Fig 6B), indicating that sufficient Nce102-Sur7 molecules are localized at furrow edges and can therefore fulfil the Sur7 function in preventing closure of furrows.

We were not able to detect PIP2 in our lipidomics analysis due to technical reasons. However, the increased levels of PIP2 in Δinp51/52 cells have been reported before. Unfortunately, detection of PIP2 via the PLCδ PH domain is not accurate, as PIP2 molecules in MCC/eisosomes are likely sequestered by the BAR domains of Pil1 and Lsp1.

- In Figure 3H freeze fracture EM, it would be better to show the distribution of the structures in wild type sample. The authors describe that the structures of 5xΔ and Δinp51/52 mutants are similar in fluorescence-based experiments, but the population of structures of 5xΔ (Figure 3H) and Δinp51/52 (Figure 6C) in freeze fracture EM seem to differ significantly. How do the authors explain this point?

In our analysis wt cells exclusively showed the typical furrows and never circular structures or tubules. Δinp51/52 in addition exhibit large invaginations that we do not observe in 5xΔ cells. These may correspond to the previously reported large invaginations that are frequently filled with cell wall (electron translucent) material. We want to stress that the membrane phenotypes in the two mutants is clearly different and even after rescue with the chimera we can still observe some large invaginations (Fig 6C, 3.3%)

- The effects of PIP2 levels and other phospholipids have been examined, but what about ergosterol levels in deficient mutants involved in furrow formation, such as 5xΔmutants?

Overall ergosterol levels in 5xKO cells were unchanged compared to wt as determined by shot gun lipidomics. The local concentration or distribution of ergosterol in furrows and tubes might very well be altered. However, we currently have no good labeling approach to address this question as it is still controversial what e.g. filipin is exactly staining in living yeast cells. Furthermore, the D4H peptide expressed in the cytosol of cells did not localize to either MCC/eisosomes or tubes in exponentially growing cells, indicating that better tools will have to be developed to clarify the role or ergosterol.

## 2nd Revision - Editorial Decision 11th Oct 2023

Prof. Roland Wedlich-Soldner
University of Muenster
Institute of Cell Dynamics and Imaging
Von-Esmarch-Str. 56
Muenster, NRW 48149
Germany

Dear Roland,

I am very pleased to accept your manuscript for publication in the next available issue of EMBO reports. Thank you for your contribution to our journal.

At the end of this email I include important information about how to proceed. Please ensure that you take the time to read the information and complete and return the necessary forms to allow us to publish your manuscript as quickly as possible.

As part of the EMBO publication's Transparent Editorial Process, EMBO reports publishes online a Review Process File to accompany accepted manuscripts. As you are aware, this File will be published in conjunction with your paper and will include the referee reports, your point-by-point response and all pertinent correspondence relating to the manuscript.

If you do NOT want this File to be published, please inform the editorial office within 2 days, if you have not done so already, otherwise the File will be published by default [contact: emboreports@embo.org]. If you do opt out, the Review Process File link will point to the following statement: "No Review Process File is available with this article, as the authors have chosen not to make the review process public in this case."

Thank you again for your contribution to EMBO reports and congratulations on a successful publication. Please consider us again in the future for your most exciting work.

Kind regards,

Martina

Martina Rembold, PhD
Senior Editor
EMBO reports

************************************************************************************

THINGS TO DO NOW:

Please note that you will be contacted by Wiley Author Services to complete licensing and payment information. The required 'Page Charges Authorization Form' is available here: https://www.embopress.org/pb-assets/embo-site/er_apc.pdf - please download and complete the form and return to embopressproduction@wiley.com

EMBO Press participates in many Publish and Read agreements that allow authors to publish Open Access with reduced/no publication charges. Check your eligibility: https://authorservices.wiley.com/author-resources/Journal-Authors/open-access/affiliation-policies-payments/index.html

You will receive proofs by e-mail approximately 2-3 weeks after all relevant files have been sent to our Production Office; you should return your corrections within 2 days of receiving the proofs.

Please inform us if there is likely to be any difficulty in reaching you at the above address at that time. Failure to meet our deadlines may result in a delay of publication, or publication without your corrections.

All further communications concerning your paper should quote reference number EMBOR-2023-57232V3 and be addressed to emboreports@wiley.com.

Should you be planning a Press Release on your article, please get in contact with emboreports@wiley.com as early as possible, in order to coordinate publication and release dates.

## EMBO Press Author Checklist


| | |
|---|---|
| Corresponding Author Name: Roland Wedlich-Söldner | |
| Journal Submitted to: EMBO Reports | |
| Manuscript Number: EMBOR-2023-57232-T | |


**USEFUL LINKS FOR COMPLETING THIS FORM**
The EMBO Journal - Author Guidelines
EMBO Reports - Author Guidelines
Molecular Systems Biology - Author Guidelines
EMBO Molecular Medicine - Author Guidelines

**Reporting Checklist for Life Science Articles (updated January**
This checklist is adapted from Materials Design Analysis Reporting (MDAR) Checklist for Authors. MDAR establishes a minimum set of requirements in transparent reporting in the life sciences (see Statement of Task: 10.31222/osf.io/9sm4x). Please follow the journal's guidelines in preparing your
**Please note that a copy of this checklist will be published alongside your article.**

**Abridged guidelines for figures**
**1. Data**
The data shown in figures should satisfy the following conditions:
- the data were obtained and processed according to the field's best practice and are presented to reflect the results of the experiments in an accurate and unbiased manner.
- ideally, figure panels should include only measurements that are directly comparable to each other and obtained with the same assay.
- plots include clearly labeled error bars for independent experiments and sample sizes. Unless justified, error bars should not be shown for technical
- if n<5, the individual data points from each experiment should be plotted. Any statistical test employed should be justified.
- Source Data should be included to report the data underlying figures according to the guidelines set out in the authorship guidelines on Data

**2. Captions**
Each figure caption should contain the following information, for each panel where they are relevant:
- a specification of the experimental system investigated (eg cell line, species name).
- the assay(s) and method(s) used to carry out the reported observations and measurements.
- an explicit mention of the biological and chemical entity(ies) that are being measured.
- an explicit mention of the biological and chemical entity(ies) that are altered/varied/perturbed in a controlled manner.
- the exact sample size (n) for each experimental group/condition, given as a number, not a range;
- a description of the sample collection allowing the reader to understand whether the samples represent technical or biological replicates (including how many animals, litters, cultures, etc.).
- a statement of how many times the experiment shown was independently replicated in the laboratory.
- definitions of statistical methods and measures:
  - common tests, such as t-test (please specify whether paired vs. unpaired), simple χ2 tests, Wilcoxon and Mann-Whitney tests, can be unambiguously identified by name only, but more complex techniques should be described in the methods section;
  - are tests one-sided or two-sided?
  - are there adjustments for multiple comparisons?
  - exact statistical test results, e.g., P values = x but not P values < x;
  - definition of 'center values' as median or average;
  - definition of error bars as s.d. or s.e.m.

**Please complete ALL of the questions below.**
**Select "Not Applicable" only when the requested information is not relevant for your study.**

**Materials**

| Newly Created Materials | Information included in the manuscript? | In which section is the information available? (Reagents and Tools Table, Materials and Methods, Figures, Data Availability Section) |
|---|---|---|
| New materials and reagents need to be available; do any restrictions apply? | Yes | Material and Methods, Table S1 |

| Antibodies | Information included in the manuscript? | In which section is the information available? (Reagents and Tools Table, Materials and Methods, Figures, Data Availability Section) |
|---|---|---|
| For **antibodies** provide the following information:<br>- Commercial antibodies: RRID (if possible) or supplier name, catalogue number and or/clone number<br>- Non-commercial: RRID or citation | Yes | Material and Methods |

| DNA and RNA sequences | Information included in the manuscript? | In which section is the information available? (Reagents and Tools Table, Materials and Methods, Figures, Data Availability Section) |
|---|---|---|
| **Short novel DNA or RNA including primers, probes**: provide the sequences. | Yes | Material and Methods, Table S1 |

| Cell materials | Information included in the manuscript? | In which section is the information available? (Reagents and Tools Table, Materials and Methods, Figures, Data Availability Section) |
|---|---|---|
| **Cell lines:** Provide species information, strain. Provide accession number in repository **OR** supplier name, catalog number, clone number, and/**OR** RRID. | Not Applicable | |
| **Primary cultures:** Provide species, strain, sex of origin, genetic modification status. | Not Applicable | |
| Report if the cell lines were recently **authenticated** (e.g., by STR profiling) and tested for mycoplasma contamination. | Not Applicable | |

| Experimental animals | Information included in the manuscript? | In which section is the information available? (Reagents and Tools Table, Materials and Methods, Figures, Data Availability Section) |
|---|---|---|
| **Laboratory animals or Model organisms:** Provide species, strain, sex, age, genetic modification status. Provide accession number in repository **OR** supplier name, catalog number, clone number, **OR** RRID. | Not Applicable | |
| **Animal observed in or captured from the field:** Provide species, sex, and age where possible. | Not Applicable | |
| Please detail **housing and husbandry conditions**. | Not Applicable | |

| Plants and microbes | Information included in the manuscript? | In which section is the information available? (Reagents and Tools Table, Materials and Methods, Figures, Data Availability Section) |
|---|---|---|
| **Plants:** provide species and strain, ecotype and cultivar where relevant, unique accession number if available, and source (including location for collected wild specimens). | Not Applicable | |
| **Microbes:** provide species and strain, unique accession number if available, and source. | Yes | Material and Methods |

| Human research participants | Information included in the manuscript? | In which section is the information available? (Reagents and Tools Table, Materials and Methods, Figures, Data Availability Section) |
|---|---|---|
| If collected and within the bounds of privacy constraints report on age, sex and gender or ethnicity for all study participants. | Not Applicable | |

| Core facilities | Information included in the manuscript? | In which section is the information available? (Reagents and Tools Table, Materials and Methods, Figures, Data Availability Section) |
|---|---|---|
| If your work benefited from core facilities, was their service mentioned in the acknowledgments section? | Yes | Acknowledgment |

**Design**

| Study protocol | Information included in the manuscript? | In which section is the information available? (Reagents and Tools Table, Materials and Methods, Figures, Data Availability Section) |
|---|---|---|

| | | |
|---|---|---|
| If study protocol has been **pre-registered, provide DOI in the manuscript**. For clinical trials, provide the trial registration number **OR** cite DOI. | Not Applicable | |
| Report the **clinical trial registration number** (at ClinicalTrials.gov or equivalent), where applicable. | Not Applicable | |

| Laboratory protocol | Information included in the manuscript? | In which section is the information available? (Reagents and Tools Table, Materials and Methods, Figures, Data Availability Section) |
|---|---|---|
| Provide DOI OR other citation details if **external detailed step-by-step protocols** are available. | Not Applicable | |

| Experimental study design and statistics | Information included in the manuscript? | In which section is the information available? (Reagents and Tools Table, Materials and Methods, Figures, Data Availability Section) |
|---|---|---|
| Include a statement about **sample size** estimate even if no statistical methods were used. | Yes | Results, Material and Methods |
| Were any steps taken to minimize the effects of subjective bias when allocating animals/samples to treatment (e.g. **randomization procedure**)? If yes, have they been described? | Not Applicable | |
| Include a statement about **blinding** even if no blinding was done. | Not Applicable | |
| Describe **inclusion/exclusion criteria** if samples or animals were excluded from the analysis. Were the criteria pre-established?<br><br>If sample or data points were omitted from analysis, report if this was due to attrition or intentional exclusion and provide justification. | Not Applicable | |
| For every figure, are **statistical tests** justified as appropriate? Do the data meet the assumptions of the tests (e.g., normal distribution)? Describe any methods used to assess it. Is there an estimate of variation within each group of data? Is the variance similar between the groups that are being statistically compared? | Yes | Material and Methods |

| Sample definition and in-laboratory replication | Information included in the manuscript? | In which section is the information available? (Reagents and Tools Table, Materials and Methods, Figures, Data Availability Section) |
|---|---|---|
| In the figure legends: state number of times the experiment was **replicated** in laboratory. | Yes | Figure Legends, Material and Methods |
| In the figure legends: define whether data describe **technical or biological replicates**. | Yes | Figure Legends, Material and Methods |

## Ethics

| Ethics | Information included in the manuscript? | In which section is the information available? (Reagents and Tools Table, Materials and Methods, Figures, Data Availability Section) |
|---|---|---|
| Studies involving **human participants**: State details of **authority granting ethics approval** (IRB or equivalent committee(s), provide reference number for approval. | Not Applicable | |
| Studies involving **human participants**: Include a statement confirming that **informed consent** was obtained from all subjects and that the experiments conformed to the principles set out in the WMA Declaration of Helsinki and the Department of Health and Human Services Belmont Report. | Not Applicable | |
| Studies involving **human participants:** For publication of **patient photos**, include a statement confirming that consent to publish was obtained. | Not Applicable | |
| Studies involving experimental **animals**: State details of **authority granting ethics approval** (IRB or equivalent committee(s), provide reference number for approval. Include a statement of compliance with ethical regulations. | Not Applicable | |
| Studies involving **specimen and field samples:** State if relevant **permits** obtained, provide details of authority approving study; if none were required, explain why. | Not Applicable | |

| Dual Use Research of Concern (DURC) | Information included in the manuscript? | In which section is the information available? (Reagents and Tools Table, Materials and Methods, Figures, Data Availability Section) |
|---|---|---|
| Could your study fall under dual use research restrictions? Please check biosecurity documents and list of **select agents and toxins** (CDC): https://www.selectagents.gov/sat/list.htm | Not Applicable | |
| If you used a select agent, is the security level of the lab appropriate and reported in the manuscript? | Not Applicable | |
| If a study is subject to dual use research of concern regulations, is the name of the **authority granting approval and reference number** for the regulatory approval provided in the manuscript? | Not Applicable | |

## Reporting

The MDAR framework recommends adoption of discipline-specific guidelines, established and endorsed through community initiatives. Journals have their own policy about requiring specific guidelines and recommendations to complement MDAR.

| Adherence to community standards | Information included in the manuscript? | In which section is the information available? (Reagents and Tools Table, Materials and Methods, Figures, Data Availability Section) |
|---|---|---|
| State if relevant guidelines or checklists (e.g., **ICMJE, MIBBI, ARRIVE, PRISMA**) have been followed or provided. | Not Applicable | |
| For **tumor marker prognostic studies,** we recommend that you follow the **REMARK** reporting guidelines (see link list at top right). See author guidelines, under 'Reporting Guidelines'. Please confirm you have followed these guidelines. | Not Applicable | |
| For **phase II and III randomized controlled trials**, please refer to the **CONSORT** flow diagram (see link list at top right) and submit the CONSORT checklist (see link list at top right) with your submission. See author guidelines, under 'Reporting Guidelines'. Please confirm you have submitted this list. | Not Applicable | |

## Data Availability

| Data availability | Information included in the manuscript? | In which section is the information available? (Reagents and Tools Table, Materials and Methods, Figures, Data Availability Section) |
|---|---|---|
| Have **primary datasets** been deposited according to the journal's guidelines (see 'Data Deposition' section) and the respective accession numbers provided in the Data Availability Section? | Not Applicable | |
| Were **human clinical and genomic datasets** deposited in a public access-controlled repository in accordance to ethical obligations to the patients and to the applicable consent agreement? | Not Applicable | |
| Are **computational models** that are central and integral to a study available without restrictions in a machine-readable form? Were the relevant accession numbers or links provided? | Yes | Material and Methods |
| If publicly available data were reused, provide the respective **data citations in the reference list**. | Not Applicable | |
